# Reductant‐Free Cross‐Electrophile Synthesis of Di(hetero)arylmethanes by Palladium‐Catalyzed Desulfinative C−C Coupling

**DOI:** 10.1002/anie.202116775

**Published:** 2022-03-14

**Authors:** Janette McKnight, Andre Shavnya, Neal W. Sach, David C. Blakemore, Ian B. Moses, Michael C. Willis

**Affiliations:** ^1^ Department of Chemistry University of Oxford Chemistry Research Laboratory 12 Mansfield Road Oxford OX1 3TA UK; ^2^ Medicine Design, Pfizer Inc. Eastern Point Road Groton CT 06340 USA; ^3^ Medicine Design, La Jolla Laboratories, Pfizer Inc. 10770 Science Center Drive San Diego CA 92121 USA; ^4^ Chemical Research and Development, Pfizer Ltd. Discovery Park, Ramsgate Rd Sandwich CT13 9ND UK

**Keywords:** Cross-Coupling, Diarylmethane, Palladium Catalysis, Reductive Coupling, Sulfinate

## Abstract

An efficient Pd‐catalyzed one‐pot desulfinative cross‐coupling to access medicinally relevant di(hetero)arylmethanes is reported. The method is reductant‐free, and involves a sulfinate transfer reagent and a Pd‐catalyst mediating the union of two electrophilic coupling partners; a (hetero)aryl halide and a benzyl halide. We establish for the first time that benzyl sulfinates, generated in situ, undergo efficient Pd‐catalyzed desulfinative cross‐coupling with (hetero)aryl halides to generate di(hetero)arylmethanes. The reaction can be extended to benzylic pseudohalides derived from benzyl alcohols. The reactions are straightforward to perform and scalable, and all reaction components are commercially available.

## Graphical abstract

Benzylic halides and heteroaryl halides are directly coupled using Pd‐catalysis and a simple sulfinate transfer reagent. The reactions proceed by formation of an intermediate benzylic sulfinate, formed in situ, and deliver di(hetero)arylmethane products. The reactions use only commercial components, and no stoichiometric metal reductant is needed.

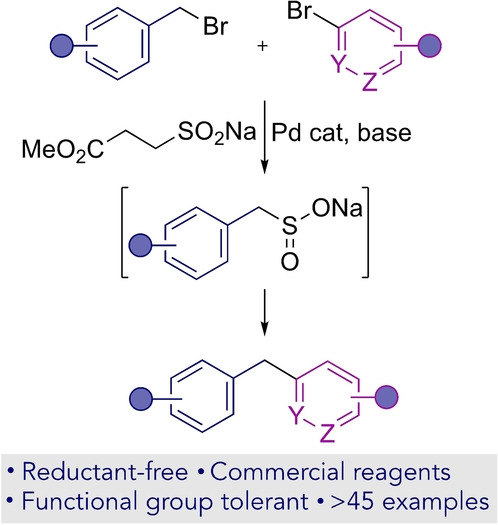

Diarylmethanes are prevalent motifs in pharmaceutically relevant molecules, host‐guest chemistry, and materials science (Figure 1).^[1]^ The drive to increase the 3D character of drug molecules^[2]^ has led to interest in developing methods for the synthesis of more conformationally flexible molecules such as diarylmethanes. Unsurprisingly, the synthesis of diarylmethanes has become a popular research area.^[3]^ Traditional approaches include vicarious nucleophilic substitution (VNS),^[4]^ Friedel–Crafts alkylation,^[5]^ which has recently been advanced,^[6]^ and transition‐metal‐catalyzed cross‐coupling of benzyl metallic reagents (Figure 2A).^[7]^ Due to use of organometallic species, these routes are often not amenable to complex functionality. Alternative methods, which often display low selectivity, include acid^[8]^ or base‐promoted benzylation,^[9]^ radical‐radical cross‐coupling reactions,^[10]^ and C−H activation approaches.^[11]^ Suzuki–Miyaura type cross‐couplings^[12]^ suffer from the use of unstable benzylic boronate reagents and narrow scopes. Notably, there have been significant advances in reductive coupling methods (Figure 2B).^[13]^ However, despite the success of these procedures, all reductive approaches, by design, require the use of formal reductants, often metals, which are usually employed in excess. This requirement can limit functional group compatibility, and in turn make application of these methods to medicinal chemistry‐relevant molecules, challenging.

**Figure 1:**
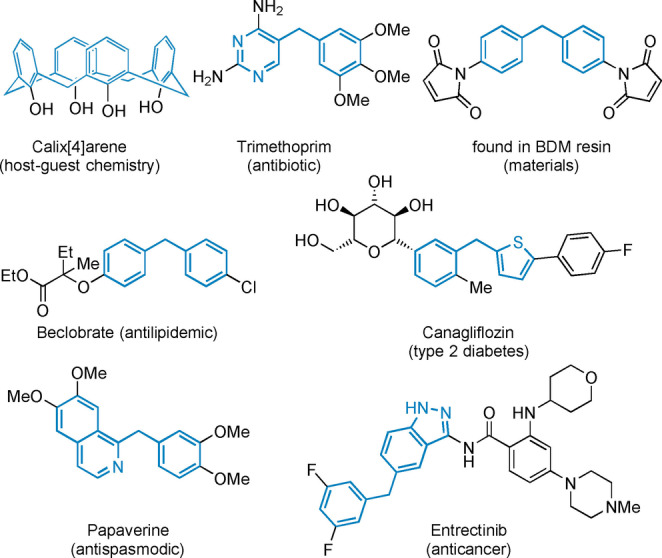
Notable di(hetero)arylmethanes in host–guest chemistry, materials, and pharmaceuticals.

**Figure 2:**
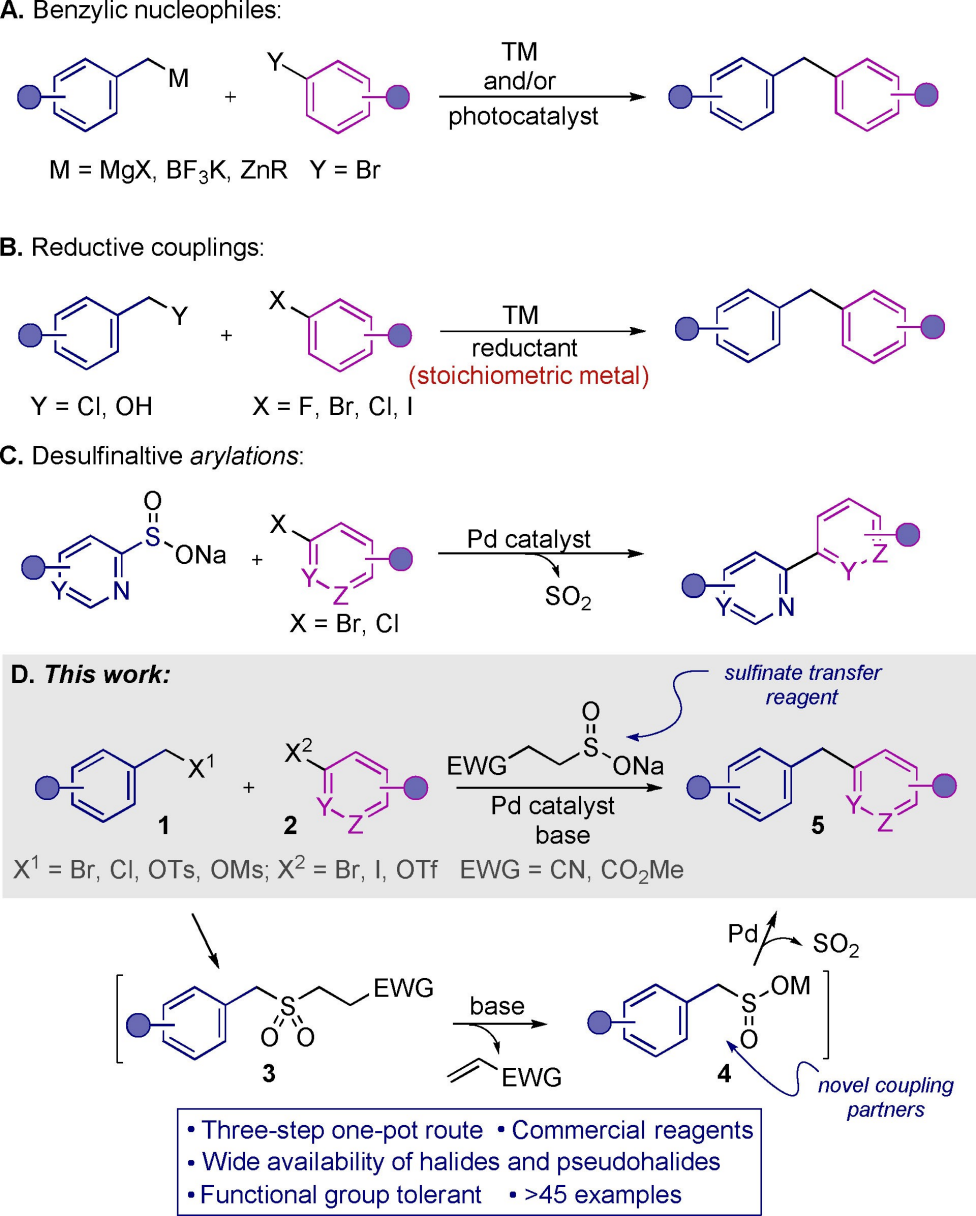
Previous synthesis of diarylmethanes and this work.

Aryl and alkyl sulfinates are often bench stable solids; they are routinely used as nucleophiles in the synthesis of sulfones, sulfonamides and other sulfonyl‐derivatives,^[14]^ and are generally attractive reagents for organic synthesis.^[15]^ Our laboratory has recently demonstrated the utility of (hetero)aryl sulfinates as nucleophilic partners in palladium‐catalyzed desulfinative cross‐coupling reactions for the synthesis of a broad range of bi(hetero)aryls (Figure 2C).^[16]^ Carbocyclic aryl sulfinates have also been employed in the Pd‐catalyzed synthesis of diarylmethanes.^[17]^

Seduced by both the attractive features of sulfinate reactivity, and the importance of bi(hetero)arylmethanes, we conceived of a route to these important targets that would complement existing methods (Figure 2D). Our approach combines a benzylic halide, a heteroaryl halide, and a sulfinate transfer reagent. The sequence commences with S‐alkylation of the sulfinate transfer reagent with the benzylic halide to form a base‐labile sulfone (**1**→**3**); base then releases the benzylic sulfinate (**3**→**4**), which then enters the Pd^0^‐catalyzed desulfinative C−C coupling reaction (**4**→**5**). All of these steps take place in a one‐pot, single‐stage operation. Importantly, no metal reductant is needed.

To validate this approach, it was important to determine that benzyl sulfinates could undergo efficient Pd‐catalyzed desulfinative cross‐coupling with (hetero)aryl halides. The required sulfinates were prepared in two steps from the corresponding halides and a sulfinate transfer reagent,^[18]^ in this case a β‐nitrile sulfinate (Scheme 1). Base‐assisted β‐elimination of acrylonitrile from the intermediate sulfones (**3**) provided the benzyl sulfinates (**4**) in good yields. This approach was not amenable to secondary benzylic halides, as although the intermediate sulfones could be formed efficiently, the elimination reactions to generate sulfinates resulted in complex mixtures of products.

**Scheme 1:**
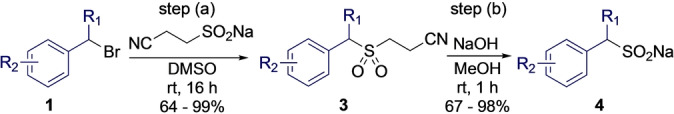
Synthesis of benzyl sulfinates. Reaction conditions: step a) benzyl bromide (1.0 equiv), β‐nitrile sulfinate (1.2 equiv), DMSO (0.34 M), rt, 16 h; step b) NaOH (0.98 equiv), MeOH (0.2 M), rt, 1–2 h.

Benzylic sulfinates proved to be efficient coupling partners in reactions with aryl halides. The full optimization is provided in the Supporting Information, but key observations were that the ligand P(^*t*^Bu)_2_Me.HBF_4_ was optimal; as shown from our study of desulfinative‐arylation, carbonate bases were essential,[^16a^, ^19^] with K_2_CO_3_ being selected; DMSO was the best performing solvent, and temperatures of 110–120 °C were needed to achieve high yields. A small scoping study coupling benzyl sulfinates with a quinoline, pyrimidine, and three pyridyl halides is shown in Scheme 2.

**Scheme 2:**
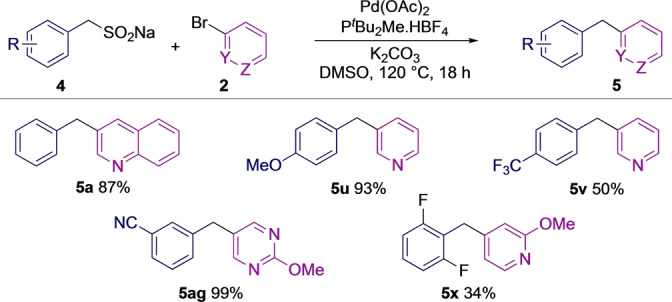
The coupling of benzyl sulfinates to heteroaryl halides.

Although the desulfinative cross‐coupling of benzyl sulfinates was generally a high yielding process (Scheme 2), these coupling partners posed several challenges; unlike (hetero)aryl sulfinates and alkyl sulfinates, which are often bench stable solids,[^15a^, ^16c^] we found that many benzylic sulfinates decomposed readily in reaction media at elevated temperatures (50 °C), or within a week when stored at room temperature. This was most notable for sulfinates bearing electron‐withdrawing groups. Furthermore, unless formed in high yield, sulfinates are often contaminated with inorganic salts that are difficult to remove, and which can lead to reduced yields in coupling reactions. In terms of wider synthetic routes and late‐stage functionalization, sulfinate functionality is not suitable for multistep elaboration. Our laboratory has previously overcome similar issues surrounding pyridine sulfinates by using latent heteroaryl nucleophiles, where the sulfinate is unmasked in situ for the coupling reaction to follow.[^16b^, ^20^] Related approaches have also been used with dienyl sulfinates.^[21]^ Applying a similar approach to the preparation of diarylmethanes led to our proposed route, and in particular, to a sequence in which the benzyl sulfinate would be formed in situ, as would the precursor sulfone (see Figure 2D).

The successful use of the benzylic sulfinates set the stage for the proposed three‐component one‐pot transformation, and pleasingly, early investigations were successful. We undertook a round of optimization, and the final conditions are shown in Table 1 (key deviations from optimal are noted). Lowering the catalyst loading, equivalents of benzyl bromide, or β‐nitrile sulfinate, led to reduced yields (entries 1–3). As in our previous work on desulfinative couplings,[^16a^, ^16b^, ^16c^, ^16d^, ^16e^] elevated reaction temperatures were crucial for high yields (entry 4). Alternative sulfinate reagents were tested, including two derivatives of rongalite (sodium hydroxymethanesulfinate dihydrate, entries 5 and 6), although both performed poorly. Control reactions showed that all reaction components were necessary for product formation (entry 7). As the β‐nitrile sulfinate reagent requires a three‐step synthesis, the opportunity to use a commercial reagent was appealing. Accordingly, we evaluated the SMOPS reagent (sodium 1‐methyl 3‐sulfinopropanoate) and, pleasingly, found no significant difference in performance (entry 8). SMOPS was therefore selected for further development. Benzyl alcohol‐derived electrophiles were explored, with benzylmethyl carbonate providing the coupled product in only 22 % yield (not shown).^[22]^ However, both benzyl tosylate and mesylate, were shown to couple efficiently (entries 9 and 10).^[23]^

**Table 1:** Optimization of the one‐pot cross‐coupling reactions.

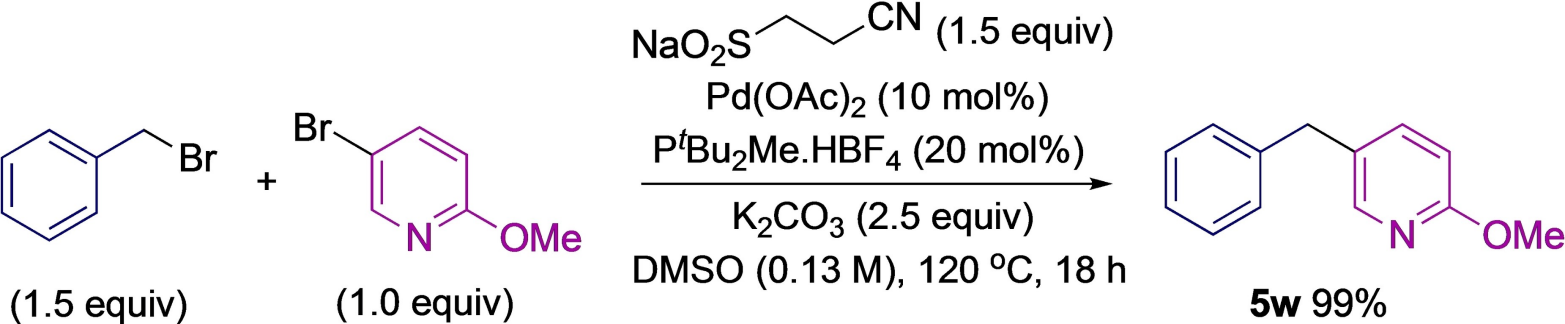		
		
Entry	Variation from conditions above	Yield [%]^[a]^
1	5 mol% Pd(OAc)_2_, β‐nitrile sulfinate (1.65 equiv)	82
2	7.5 mol% Pd(OAc)_2_, β‐nitrile sulfinate (1.65 equiv)	84
3	BnBr (1.0 equiv), β‐nitrile sulfinate (1.1 equiv)	70
4	110 °C	72
5	Rongacyl (**A**) instead of β‐nitrile sulfinate	40
6	TBSOCH_2_SO_2_Na (**B**) and CsF instead of β‐nitrile sulfinate	14
7	No sulfinate reagent	0
8	SMOPS instead of β‐nitrile sulfinate	99
9	BnOTs used instead of BnBr, SMOPS used	98^[b]^
10	BnOMs used instead of BnBr, SMOPS used	70^[b]^
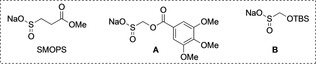		

Reactions were performed on 0.2 mmol scale in a sealed microwave vial under an inert atmosphere. [a] HPLC yields using *p*‐tolyl ether as an internal standard. [b] Yield from cross‐coupling to 3‐bromoquinoline.

With reagents and conditions optimized, we next explored the scope of the process (Scheme 3). In the evaluation of the benzylic coupling partner, reactions of 3‐bromoquinoline with benzyl bromides bearing electron‐donating (**5** **b**–**d**), electron‐withdrawing (**5** **e**), sterically demanding (**5** **b**) and acidic functional groups (**5** **f**), performed well. Bromo (**5** **g**, **h**), chloro (**5** **i**), and fluoro‐substituents (**5** **j**, **k**) were all tolerated. A number of heterocyclic benzylic halides, including thiophene (**5** **l**), isoxazole (**5** **m**), functionalized quinoline (**5** **n**), and benzothiazole (**5** **o**) were also used with success. The use of related pyridyl substrates was challenging, as although the corresponding SMOPS‐derived sulfones could be formed, the resultant sulfinate intermediates were unstable and decomposition resulted; pyridine **5** **p** is an illustrative example. While the thiophene (**5** **l**) yield is low, this is a notable example, as the corresponding isolated sulfinate was unproductive when used in a direct coupling reaction and was unstable to storage. This was a common trait of several heteroaromatic benzylic sulfinates. A cinnamyl bromide delivered an efficient reaction (**5** **q**), but an isomeric mixture of alkenes was obtained. Finally, other medicinally relevant functional groups, such as sulfone (**5** **r**), pentafluorosulfanyl (**5** **s**) and trifluoromethyl (**5** **t**) could be incorporated.

**Scheme 3:**
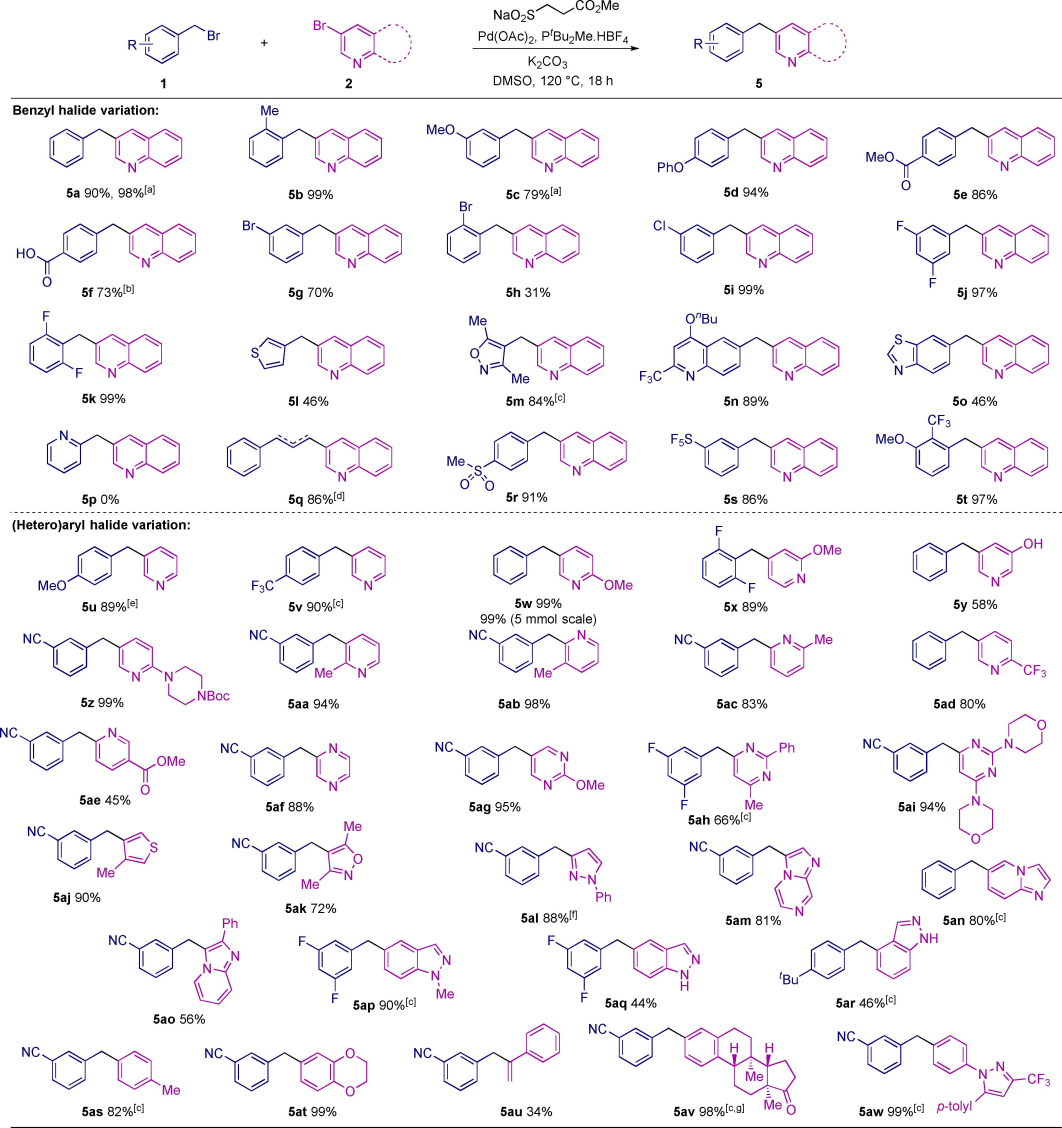
The one‐pot desulfinative cross‐coupling of benzyl halides to (hetero)aryl halides. Reaction conditions: benzyl bromide (0.3 mmol, 1.5 equiv), X‐(Het)Aryl (0.2 mmol, 1.0 equiv), Pd(OAc)_2_ (10 mol %), P^*t*^Bu_2_Me.HBF_4_ (20 mol %), SMOPS (0.3 mmol, 1.5 equiv), K_2_CO_3_ (0.5 mmol, 2.5 equiv), DMSO (0.13 M, 1.5 mL), 120 °C, 18 h. [a] Benzyl tosylate used. [b] Isolated as the ethyl ester. [c] β‐nitrile sulfinate used in place of SMOPS. [d] Alkene isomers 1 : 1 ratio, combined yield of 86 %. [e] 4‐Methoxybenzyl chloride used. [f] Heteroaryl iodide employed. [g] Aryl triflate employed.

Next, we explored the scope of the (hetero)aryl coupling partner, and in general, a broad range of heterocycles could be used. 3‐Bromopyridine (**5** **u**, **v**) as well as derivatives bearing electron‐donating (**5** **w**–**ac**), electron‐withdrawing (**5** **ad**, **ae**), and sterically demanding (**5** **aa**–**ac**) groups could all be employed. A free hydroxyl group was tolerated (**5** **y**). High yields were achieved for reactions with other important heterocyclic motifs, such as pyrazine (**5** **af**), pyrimidines (**5** **ag**–**ai**), thiophene (**5** **aj**), isoxazole (**5** **ak**), as well as a protected pyrazole (**5** **al**). Imidazolepyrazine (**5** **am**) and imidazolepyridine (**5** **an**) examples were also successful. Several indazole analogs (**5** **ap**–**ar**) were prepared, including N−H examples. Indazole **5** **aq** is notable, as it maps directly onto the heterocyclic core of the anticancer compound Entrectinib (see Figure 1). Carbocyclic aryl halides could be used (**5** **as**,**at**), as could α‐bromostyrene, although in lower yield (**5** **au**). An aryl triflate derived from an estrone fragment (**5** **av**), and a celecoxib motif (**5** **aw**) were also successful. Importantly, the reaction could be performed on 5 mmol/gram scale using a simple round bottom flask and condenser, rather than in a sealed vial, and pyridine **5** **w** was obtained in excellent yield in this way (99 %, 986 mg).

To further highlight the benefit of using the three‐component one‐pot method, control experiments comparing benzyl sulfinate, sulfone, and halide starting materials were performed (Scheme 4). A trifluoromethylbenzyl group was selected for these reactions, and all variants were coupled with 3‐bromopyridine. The sulfinate reagent delivered the coupled product **5** **ax** in 50 % yield (eq. 1); the β‐nitrile sulfone was more successful, providing the diarylmethane in 99 % yield (eq. 2), while the cross‐electrophile process, starting from the aryl halide, afforded the coupled product in 90 % yield (eq. 3). These reactions highlight the challenges associated with using benzylic sulfinate reagents. This particular sulfinate was shown to decompose within a week when stored on the bench at room temperature, and so it is likely that this decomposition was accelerated at the elevated temperatures of the coupling reaction. The sulfone reaction demonstrates the benefit of releasing the sulfinate in situ when the sulfinate has stability issues. Finally, although the one‐pot conditions were slightly less efficient, the three‐component process remains superior due to eliminating the need to prepare and purify intermediate reagents.

**Scheme 4:**
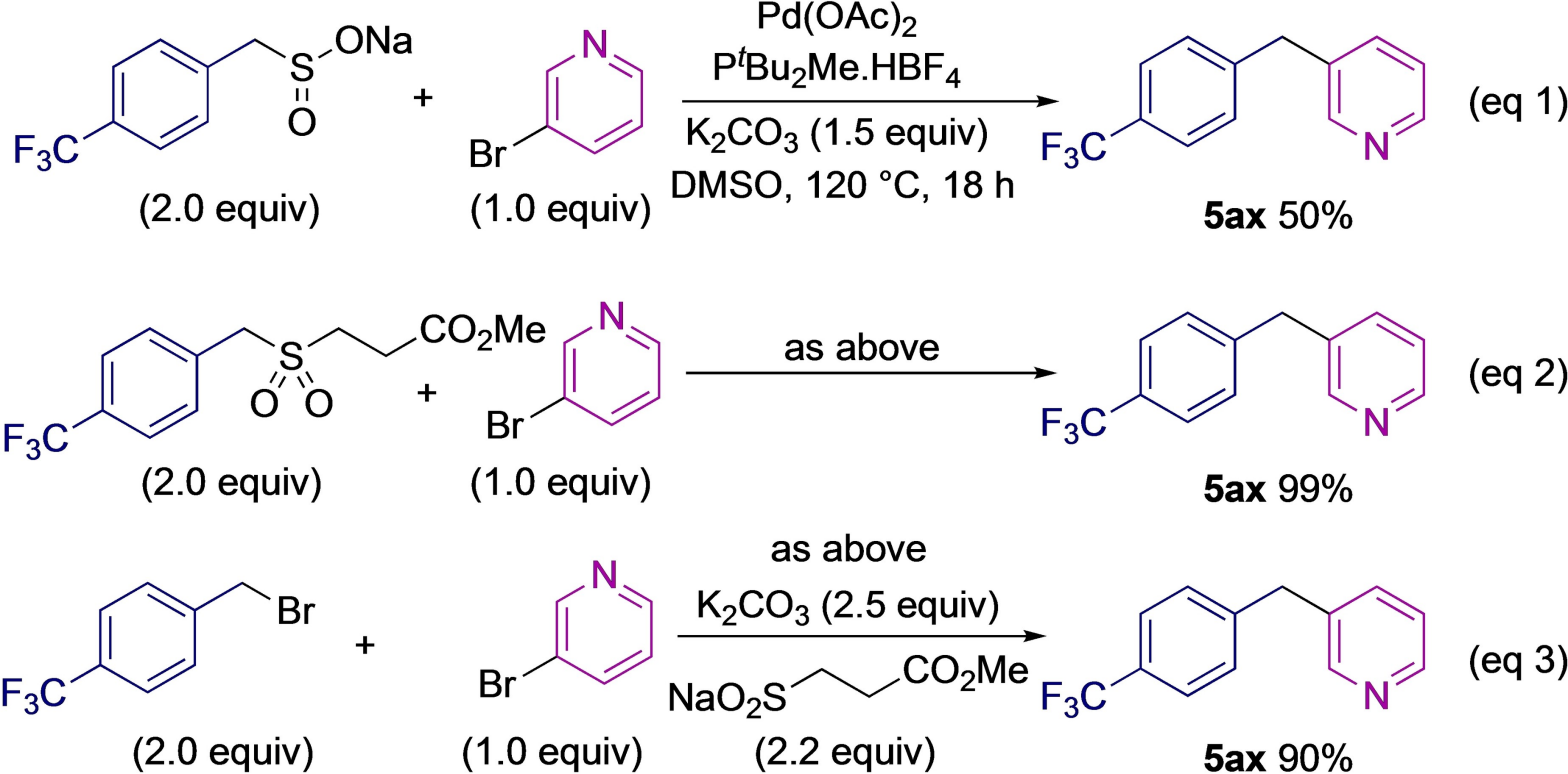
Comparison of reactions with a sulfinate, sulfone or the ‘one‐pot’ components.

In summary, an operationally simple, one‐pot Pd‐catalyzed desulfinative cross‐coupling method of broad scope has been developed for the synthesis of pharmaceutically important di(hetero)arylmethanes. The reactions join (hetero)aryl halides (and triflates) and benzyl halides. Numerous functionalities are tolerated on either coupling partner, and the reaction can be extended from benzyl halides to benzyl sulfonates. The chemistry can be scaled, while retaining excellent yields. All reaction components are readily available, including a highly diverse commercial pool. For these reasons, we envisage this methodology to be useful for synthetic chemists in fields who search for reliable and versatile methods to rapidly access di(hetero)arylmethane scaffolds.

## Conflict of interest

The authors declare no conflict of interest.

## Supporting information

As a service to our authors and readers, this journal provides supporting information supplied by the authors. Such materials are peer reviewed and may be re‐organized for online delivery, but are not copy‐edited or typeset. Technical support issues arising from supporting information (other than missing files) should be addressed to the authors.

Supporting Information

## Data Availability

The data that support the findings of this study are available in the supplementary material of this article.
