# Supplementary material for: Reductant‐Free Cross‐Electrophile Synthesis of Di(hetero)arylmethanes by Palladium‐Catalyzed Desulfinative C−C Coupling
Source: Angew Chem Int Ed Engl. 2022 Mar 14;61(19):e202116775. doi: 10.1002/anie.202116775 (PMC9314995; doi:10.1002/anie.202116775)
Supplement: Supplementary file 1 — Supporting Information [file ANIE-61-0-s001.pdf]

Angewandte Chemie
Eine Zeitschrift der Gesellschaft Deutscher Chemiker

Supporting Information

Reductant-Free Cross-Electrophile Synthesis of Di(hetero)arylmethanes by Palladium-Catalyzed Desulfinative C–C Coupling

*J. McKnight, A. Shavnya, N. W. Sach, D. C. Blakemore, I. B. Moses, M. C. Willis**

Table of Contents

1. **General Information** S2
2. **Benzyl sulfinate synthesis** S3
3. **Benzyl sulfinate desulfinative cross-coupling optimisation** S3
3.1 Screening sodium benzyl sulfinate S4
3.2 Ligand screen examples S5
3.3 Solvent screen examples S5
3.4 Control reactions S6
4. **One-pot optimisation** S7
4.1 Control reactions S8
4.2 Alternative sulfinate reagents S9
4.3 Lowering the amount of sulfinate reagent S9
4.4 Pseudohalides subjected to the cross-coupling conditions S10
5. **HPLC calibration curves** S11
5.1 General method S11
5.2 Monitoring the one-pot reaction profile S11
6. **Synthesis of starting materials** S17
6.1 Sulfinate reagent synthesis S17
6.2 Sulfone synthesis S18
6.2.1 General Procedure A S18
6.2.2 General Procedure B S18
6.3 Sulfinate synthesis S20
6.3.1 General Procedure C S20
6.3.2 General Procedure D S20
6.4 Benzyl tosylate synthesis S22
7.0 **Desulfinative cross-coupling products** S23
7.1 General procedure E S23
7.2 General procedure F S23
8.0 **^{1}H, ^{13}C and ^{19}F NMR Spectra** S41
8.1 Starting material spectra S41
8.2 Di(hetero)arylmethane spectra S56
References S117

1. General Information

Reactions were performed with continuous magnetic stirring under an atmosphere of nitrogen with anhydrous solvents unless otherwise stated using standard Schlenk techniques. Anhydrous DMSO was purchased as anhydrous from Sigma-Aldrich. All glassware was oven dried at >100 °C, and was allowed to cool to room temperature under a positive nitrogen pressure.

Reagents, ligands and catalysts were purchased from Acros Organics, Alfa Aesar Ltd, Fluorochem Ltd, Insight Biotechnology Ltd, Sigma-Aldrich, Strem Chemicals Inc., and were used as supplied unless stated otherwise. Palladium acetate was purchased from Strem Chemicals Inc. and di-*tert*-butyl(methyl)phosphonium tetrafluoroborate was purchased from Insight Biotechnology Ltd.

The reagent '*Rongacyl*' was prepared according to the literature procedure.[1] The reagents 2-bromopyridine, 2-chloropyridine, 3-bromopyridine and 3-chloropyridine were distilled prior to use *via* short path distillation with a Kugelrohr.

Reactions were followed by thin layer chromatography (TLC) when practical. TLC was performed on Merck aluminium silica gel 60 F_{254} precoated plates. Plates were visualised under an ultraviolet lamp (λ_{max} = 254 nm or 365 nm) and/or by staining with potassium permanganate solution. Flash column chromatography (FCC) was carried out on Merck silica gel 60 (230 – 400 mesh) and the solvent system used reported in parenthesis. The compound to be purified was loaded as an oil or pre-absorbed onto celite. Petrol refers to the fraction of light petroleum ether boiling in the range of 40 – 60 °C. HPLC samples were analyzed using an Ascentis® Express C18 10 cm x 4.6 mm, 2.7 µm column (cat# 53827-U), with an eluent system combining acetonitrile and water. Collection wavelengths were 210 nm, 230, 254 nm, and 280 nm. Data was processed using Chem Station for LC systems Rev.B.04.01.SP1.

^{1}H NMR spectra were recorded on a Bruker AVIII400 (400 MHz) spectrometer using the residual solvent as an internal reference, ^{13}C NMR spectra were recorded on a Bruker AVIII400 (101 MHz) using the residual solvent as an internal reference and ^{19}F NMR spectra were obtained on a Bruker AVIII400 (377 MHz) spectrometer using $CFCl_3$ as an external reference. Both ^{13}C and ^{19}F NMR spectra were recorded proton decoupled. Acquisitions were carried out at room temperature unless otherwise stated. Chemical shifts (δ_H, δ_C and δ_F) are quoted in parts per million (ppm) with the multiplicities of the spectra reported as the following: *s*, singlet; *d*, doublet; *t*, triplet; *q*, quartet; *p*, quintet; *m*, multiplet; *app*., apparent; *br*, broad; *dd*, doublet of doublets *et cetera*. Coupling constants (*J*) are given in Hertz (Hz) and rounded to the nearest 0.5 Hz.

High resolution mass spectra (HRMS) were recorded by the mass spectrometry service at the Chemistry Research Laboratory, University of Oxford, using a Bruker Daltonic µTOF spectrometer (ESI). Samples for HRMS were prepared as a <1 mg/mL solution in MeOH (HRMS-ESI). *m/z* values are reported in Daltons (Da) and high resolution values are calculated to four decimal places from the molecular formula, with all found values in a tolerance of 5 ppm.

Infrared spectra were recorded using a Bruker Tensor 27 Fourier Transform spectrometer using a diamond ATR module. Absorption maximum, ν_{max}, are given in wavenumbers (cm^{-1}) and are assigned as broad (*br*) or strong (*s*) when appropriate.

Melting points are recorded in degrees Celsius (°C) using a Stuart Scientific Melting Point Apparatus SMP1 and are reported uncorrected.

Optical rotations were measured on a Schmidt Haensch UniPol L2000 polarimeter at 589 nm, 25 °C. $[\alpha]_D^{25}$ is expressed in deg cm^3 g^{-1} dm^{-1} and *c* is expressed in g 100 cm^{-3}.

Systematic names in accordance with the guidelines specified by the International Union of Pure and Applied Chemistry (IUPAC) were generated using the computer program ChemDraw.

2. Benzyl sulfinate synthesis

The βECN group could be used as a readily accessible reagent to synthesise benzyl sulfinates (Table S1). Entries 3 and 4 show that this group can be directly installed in excellent yields via a S_N2 reaction with benzyl bromide at room temperature.

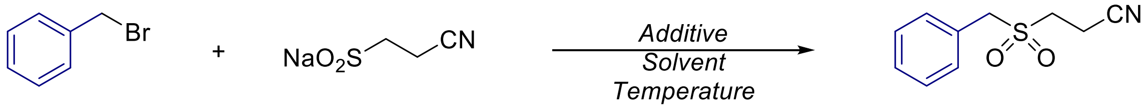

Table S1. Optimisation of benzyl sulfinate synthesis.

Entry	Additive	Solvent	Temperature /°C	HPLC Yield /%
1	K_2CO_3[a]	DMSO	40	20
2	TBACl[b]	DMAc	75	87
3	-	DMSO	21	91
4	-	DMAc	21	89

Reaction conditions: Benzyl bromide (0.34 mmol, 1.0 equiv.), sodium 2-cyanoethane-1-sulfinate (0.41 mmol, 1.2 equiv.), solvent (1.0 mL) [a] K_2CO_3 (1.0 equiv.). [b] TBACl (0.3 equiv.).

The following sulfone, prepared from a secondary benzylic bromide, could be isolated, but the conversion to the corresponding sulfinates was unsuccessful:

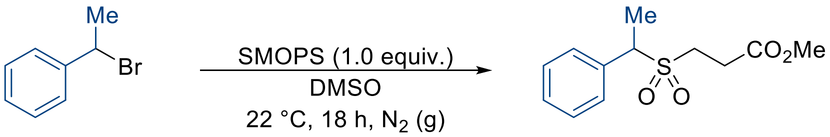

87%, 0.3 mmol scale
96%, 1.2 mmol scale

3. Benzyl sulfinate desulfinative cross-coupling optimisation

3.1 Screening sodium benzyl sulfinate

With pure benzyl sulfinate reagent in hand, an initial screen was performed with 6-bromoquinoline as the coupling partner on a range of palladium and nickel catalyst systems (Table S2).

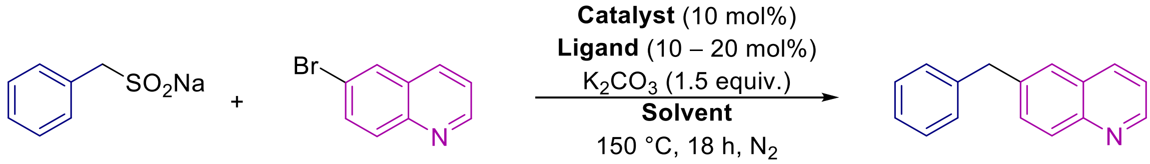

Table S2. Initial desulfinylative cross-coupling screen between benzyl sulfinate with 6-bromoquinoline.

Entry	Catalyst	Ligand/Additive	Solvent	Yield /%[a]
1	$Pd(OAc)_2$	PCy_3[b]	Dioxane	69
2	$NiBr_2$.Glyme	BPhen[c]	DMSO	22
3	$PdCl_2$	PCy_3[b]	Dioxane	62
4	$NiCl_2(PPh_3)_2$	-	Dioxane	Trace
5	$NiCl_2(PCy_3)_2$	-	Dioxane	Trace
6	$NiCl_2$(1,3-dppp)	-	Dioxane	Trace
7	$NiBr_2$.Glyme	PPh_3[b]	DMSO	Trace
8	$Pd(OAc)_2$	$P(^tBu)_2Me$[b]	Dioxane	69
9	$Pd(OAc)_2$	BPhen[c]	Dioxane	Trace
10	$PdCl_2$	PCy_3[b]	Dioxane	61
11	$PdCl_2$	BPhen[c]	Dioxane	Trace
12	$PdCl_2$	Cu_2O[b]	1:1 H_2O/ Dioxane	Trace[d]
13	$PdCl_2$	-	1:1 H_2O/ Dioxane	Trace[d]
14	[(TMEDA)Ni(o-tolyl)Cl]	PCy_3[b]	Dioxane	0

[a] Isolated yields. [b] 20 mol% used. [c] 10 mol% used. [d] Reactions were conducted at 100 °C.

3.2 Ligand screen examples

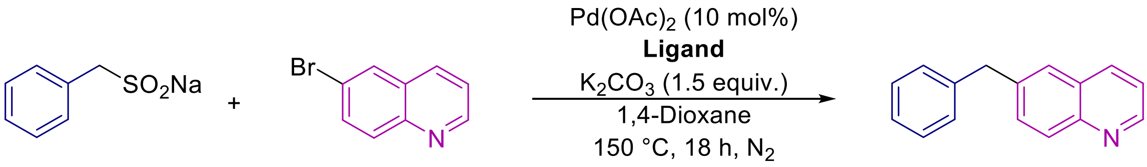

Table S3. Ligand screen.

Entry	Ligand	Yield /% (150 °C)	Yield /% (120 °C)
1	PCy_3[a]	69	49
2	P(^{t}Bu)$_2$Me.HBF$_4$[a]	86	76
3	P(^{t}Bu)$_3$.HBF$_4$[a]	7	-
4	PPh_3[a]	21	-
5	XantPhos[b]	12	-
6	Dppe[b]	33	-
7	p-OMe-Dppe[b]	35	-
8	PCy_2Et[a]	-	60

Reaction conditions: benzyl sulfinate (0.2 mmol, 1.0 equiv.), 6-bromoquinoline (0.2 mmol, 1.0 equiv.), $Pd(OAc)_2$ (10 mol%), ligand (10 – 20 mol%), K_2CO_3 (0.3 mmol, 1.5 equiv.), Dioxane (0.1 M, 2 mL). [a] 20 mol% used. [b] 10 mol% used.

3.3 Solvent screen examples

Polar aprotic solvents were superior to the non-polar solvents presumably due to the increased solubility of the sulfinate salt and inorganic base (Table S4). Although there is not much difference in yield for entries 1 – 4, less complex reaction profiles (by HPLC) were observed for entries 1 – 3. Sulfolane gave a comparative yield to the other dipolar aprotics, which illustrates that if the reaction was scaled up, there would be a suitable alternative solvent for industrial processes (Entry 3). However, sulfolane was less desirable to use on small scale due to having a melting point around room temperature; needle blockage was often an issue.

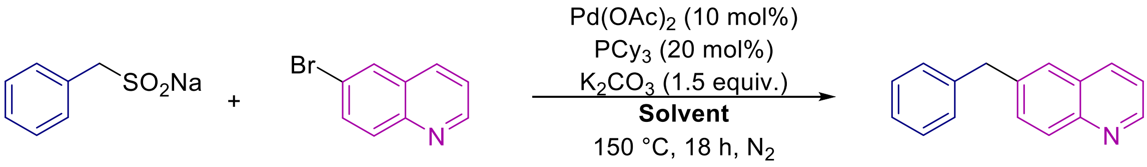

Table S4. Ligand screen.

Entry	Solvent	Yield /%
1	Dioxane	69
2	DMSO	76
3	Sulfolane	77[a]
4	DMF	75
5	Toluene	0
6	Anisole	0
7	DMA	69

Reaction conditions: benzyl sulfinate (0.2 mmol, 1.0 equiv.), 6-bromoquinoline (0.2 mmol, 1.0 equiv.), $Pd(OAc)_2$ (10 mol%), PCy_3 (20 mol%), K_2CO_3 (0.3 mmol, 1.5 equiv.), solvent (0.1 M). [a] Reaction conducted at 120 °C.

Minimal difference in yield was observed when either two or three equivalents of sulfinates were used (Entries 3 and 4, Table S5), so it was preferable to use two equivalents. There was a dramatic increase in yield when the solvent was swapped to DMSO for this particular substrate. Increasing the equivalents of base resulted in a yield reduction (Entry 5). Running the reaction at higher concentration makes the work up easier, although little change in yield is observed (Entry 6). Heating the reaction to 150 °C gave no further improvement in yield (Entry 7).

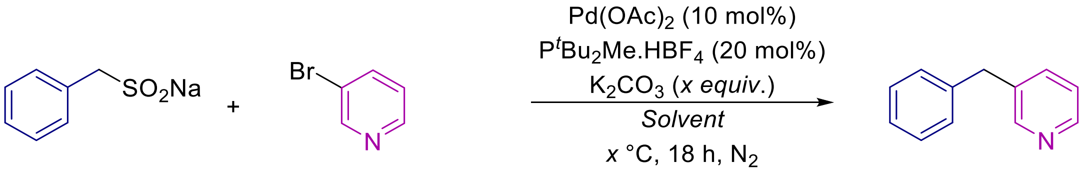

Table S5. The effects of altering the stoichiometry of the reagents on reaction performance.

Entry	Solvent	Sulfinate /equiv.	K_2CO_3 /equiv.	Temperature /°C	Solvent volume /mL	Yield /%
1	Dioxane	1	1.5	150	2	28
2	DMSO	1	1.5	120	2	63
3	DMSO	2	1.5	120	2	81
4	DMSO	3	1.5	120	2	89
5	DMSO	2	2	120	2	20
6	DMSO	2	1.5	120	1	76
7	DMSO	2	1.5	150	2	82

[a] Reaction conditions: benzyl sulfinate (0.4 mmol, 2.0 equiv.), 3-bromopyridine (0.2 mmol, 1.0 equiv.), $Pd(OAc)_2$ (10 mol%), $P^tBu_2Me.HBF_4$ (20 mol%), K_2CO_3 (0.3 mmol, 1.5 equiv.), DMSO (0.2 M, 1 mL).

3.4 Control reactions

Control experiments for the reaction of benzyl sulfinate with 6-bromoquinoline showed that all the reaction components are essential for full conversion to the desired product (Table S6).

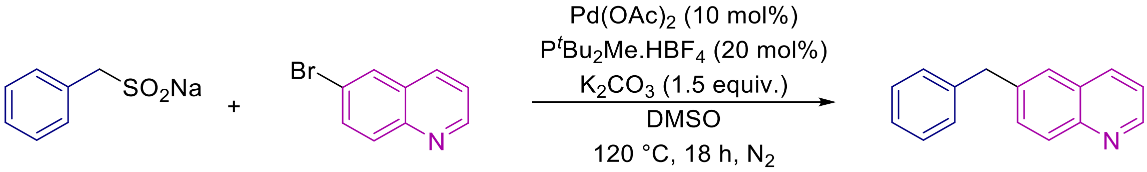

Table S6. Control experiments.

Entry	$Pd(OAc)_2$	$P^tBu_2Me.HBF_4$	K_2CO_3	Yield /%
1	Yes	Yes	Yes	99
2	Yes	Yes	-	6
3	-	-	Yes	0
4	-	-	-	0

[a] Reaction conditions: benzyl sulfinate (0.3 mmol, 1.5 equiv.), 6-bromoquinoline (0.2 mmol, 1.0 equiv.), $Pd(OAc)_2$ (10 mol%), $P^tBu_2Me.HBF_4$ (20 mol%), K_2CO_3 (0.3 mmol, 1.5 equiv.), DMSO (0.2 M, 1 mL)

Control experiments for the reaction of benzyl sulfinate with 3-bromopyridine were employed using freshly dried K_2CO_3 and anhydrous DMSO from an unopened bottle (Table S7). Clearly it is not necessarily water in the reaction that causes reduction in yield (Entry 1) but rather is due to the air content of the reaction mixture (Entry 2). Interestingly, Cs_2CO_3 (freshly dried) lead to a steep reduction in yield (Entry 3).

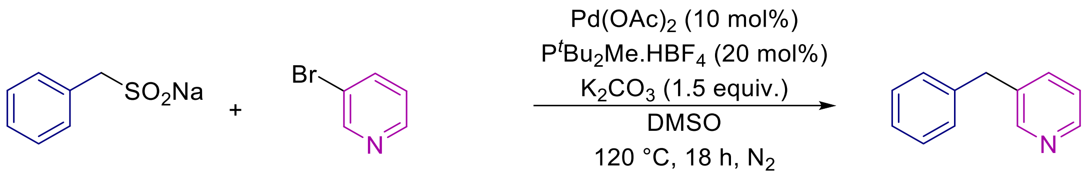

Table S7. Control experiments.

Entry	Deviation	Yield /%
1	Degassed H_2O (1.0 equiv.)	93
2	Air atm.	54
3	Cs_2CO_3 used instead of K_2CO_3	31

[a] Reaction conditions: benzyl sulfinate (0.4 mmol, 2.0 equiv.), 3-bromopyridine (0.2 mmol, 1.0 equiv.), $Pd(OAc)_2$ (10 mol%), $P^tBu_2Me.HBF_4$ (20 mol%), K_2CO_3 (0.3 mmol, 1.5 equiv.), DMSO (0.2 M, 1 mL)

4. One-pot optimisation

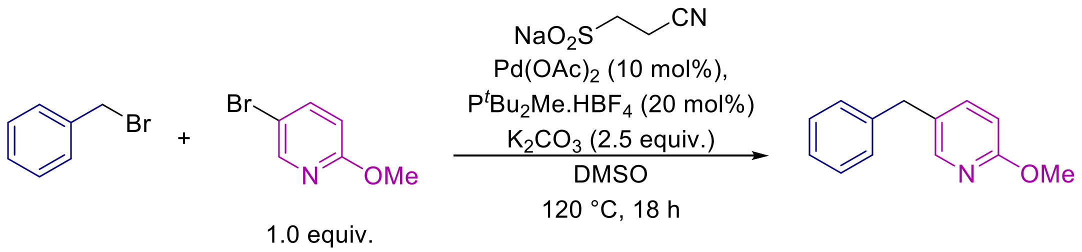

Table S8. One-pot optimisation.

Entry	BnBr equiv.	Sulfinate equiv.	DMSO conc. /M	HPLC yield /%[d]
1	2.0	2.2	0.2	78
2	1.0	1.1	0.2	70
3	1.5	1.65	0.2	90
4	2.0	2.2	0.13	96
5	1.5	1.65	0.13	94
6	1.5	1.65	0.13	82[a]
7	1.5	1.65	0.13	84[b]
8	1.5	1.5	0.13	99
9	1.5	1.5	0.13	76[c]
10	1.5	1.5	0.13	72[d]

[a] 5 mol% $Pd(OAc)_2$. [b] 7.5 mol% $Pd(OAc)_2$. [c] Performed in an round-bottom flask fitted with a condenser rather than in a sealed vial. [d] At 110 °C. [d] HPLC yields using *p*-tolyl ether as an internal standard.

A screen of some solvents listed on green solvent guides[2] gave some promise, but none were as successful as DMSO (Table S9).

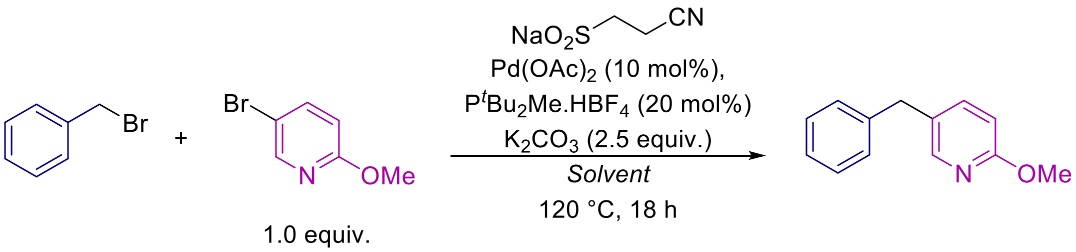

Table S9. Green solvent screen.

Entry[a]	Solvent	HPLC Yield /%[b]
1	Anisole	55
2	Sulfolane	43
3	Diethyl carbonate	30
4	1-Octanol	0

[a] Reactions conditions: benzyl bromide (0.3 mmol, 1.5 equiv.), heteroarylhalide (0.2 mmol, 1.0 equiv.), sulfinate reagent (0.3 mmol, 1.5 equiv.), $Pd(OAc)_2$ (10 mol%), $P^tBu_2Me.HBF_4$ (20 mol%), K_2CO_3 (0.5 mmol, 2.5 equiv.), solvent (0.13 M, 1.5 mL). [b] HPLC yields using *p*-tolyl ether as an internal standard.

4.1 Control reactions

Control tests behaved as expected with all the reaction components shown to be necessary to achieve good yields of cross-coupled products for the one-pot procedure (Table S10).

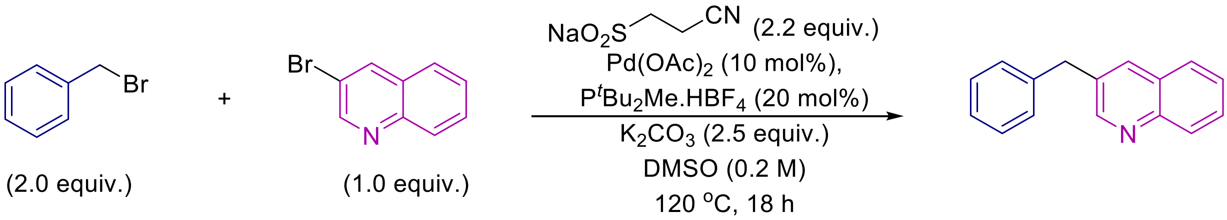

Table S10. Control reactions showing that all reagents are necessary for efficient cross-coupling.

Entry[a]	βECN sulfinate reagent	[Pd] and Ligand	K_2CO_3	Isolated yield /%
1	Yes	Yes	Yes	78
2	No	Yes	Yes	0
3	Yes	No	Yes	Trace[b]
4	Yes	Yes	No	0

[a] Reaction conditions: Benzyl bromide (2.0 equiv.), 3-bromoquinoline (1.0 equiv., 0.2 mmol), βECN sulfinate (2.2 equiv.), $Pd(OAc)_2$ (10 mol%), $P^tBu_2Me.HBF_4$ (20 mol%), K_2CO_3 (2.5 equiv.), DMSO (0.2 M), 120 °C, 18 h. [b] Trace product peak found in the mass spectra.

4.2 Alternative sulfinate reagents

Other sulfinate reagents were tested, including two derived from the very cheap and readily available sulfinate called rongalite (Table S11). While there appeared to be no considerable yield difference between using the βECN or SMOPS sulfinate reagents, due to the commercial availability of SMOPS, we decided to select this reagent.

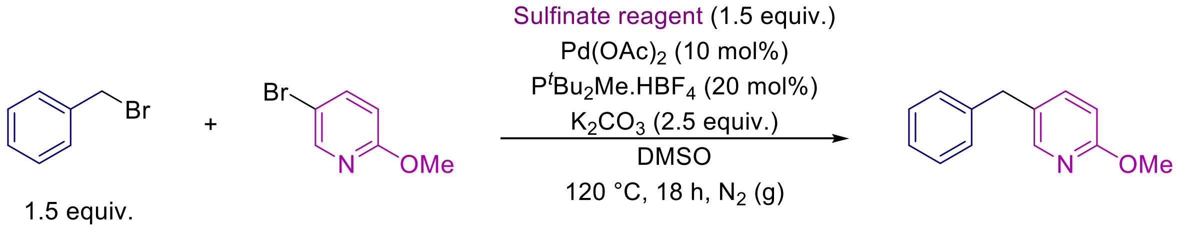

Table S11. Sulfinate reagent selection.

Entry[a]	Sulfinate reagent	K_2CO_3 equiv.	Additive	Yield /%[b]
1	Rongacyl	2.5	-	40
2	$OTBSCH_2SO_2Na$	1.5	CsF (2.0 equiv.)	14
3	βECN	2.5	-	99
4	SMOPS	2.5	-	99

[a] Reactions conditions: benzyl bromide (0.3 mmol, 1.5 equiv.), (heteroaryl)bromide (0.2 mmol, 1.0 equiv.), sulfinate reagent (0.3 mmol, 1.5 equiv.), $Pd(OAc)_2$ (10 mol%), $P^tBu_2Me.HBF_4$ (20 mol%), K_2CO_3 (0.5 mmol, 2.5 equiv.), solvent (0.13 M, 1.5 mL). [b] HPLC yields using *p*-tolyl ether as an internal standard.

4.3 Lowering the amount of sulfinate reagent

Excess of the benzyl bromide and SMOPS reagents was critical for high yielding cross-coupling (Table S12). It is important to note that if the BnX coupling partner is expensive/in scarce supply, the equivalents can be lowered slightly without having a detrimental impact; there is little difference in yield for 1.4 or 1.5 equivalents (Entries 4 and 5).

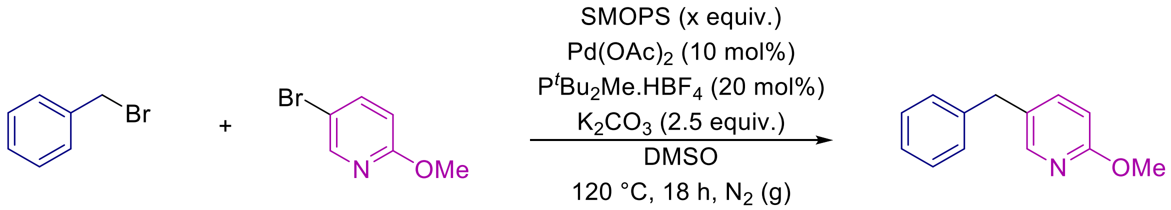

Table S12. Stoichiometry optimisation.

Entry	Benzyl bromide /equiv.	*SMOPS* /equiv.	HPLC Yield /%[b]
1	1.1	1.1	77
2	1.2	1.2	86
3	1.3	1.3	89
4	1.4	1.4	98
5	1.5	1.5	99

[a] Reactions conditions: benzyl bromide (0.3 mmol, 1.5 equiv.), (heteroaryl)bromide (0.2 mmol, 1.0 equiv.), SMOPS sulfinate reagent (x equiv.), $Pd(OAc)_2$ (10 mol%), $P^tBu_2Me.HBF_4$ (20 mol%), K_2CO_3 (0.5 mmol, 2.5 equiv.), DMSO (0.13 M, 1.5 mL). [b] HPLC yields using *p*-tolyl ether as an internal standard.

4.4 Pseudohalides subjected to the cross-coupling conditions

Altering the halide on the heteroaryl coupling partner from a bromide to a chloride did not have a drastic effect on yield, however tosylate and triflate groups do not seem to be well tolerated under the reaction conditions (Table S13).

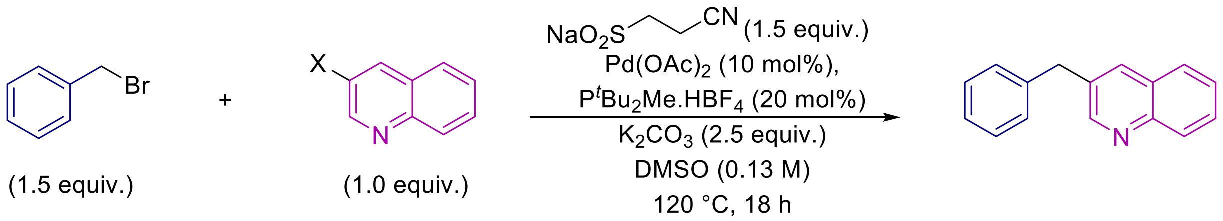

Table S13. The effect of altering the halide on the heteroaryl coupling partner.

Entry[a]	(Pseudo)halide	HPLC Yield /%[b]
1	Br	99
2	Cl	86
3	OTs	48
4	OTf	19

[a] Reactions conditions: benzyl bromide (0.3 mmol, 1.5 equiv.), (heteroaryl)pseudohalide (0.2 mmol, 1.0 equiv.), βECN sulfinate reagent (0.3 mmol, 1.5 equiv.), $Pd(OAc)_2$ (10 mol%), $P^tBu_2Me.HBF_4$ (20 mol%), K_2CO_3 (0.5 mmol, 2.5 equiv.), DMSO (0.13 M, 1.5 mL). [b] HPLC yields using *p*-tolyl ether as an internal standard.

Due to the wide availability and affordability of benzyl alcohols, we investigated whether these derivatives could be employed in place of benzyl bromides (Table S14). Unsurprisingly, coupling benzyl alcohol directly led to no desired product (Entry 1). Commonly used derivatives are benzyl carbonate coupling partners,[3] but when subjected to our reaction conditions the desired product was synthesised in poor yield (Entry 2). Both benzyl tosylates and mesylates were shown to couple successfully under the desulfinative one-pot reaction conditions (Entries 3 – 4). Although often suffering from instability on storage and synthesis in liquid media due to facile hydrolysis,[4] they show alcohol derived reagents can be utilized. We envisage that quick consumption of the tosylate, in the initial S_N2 sulfone formation step, avoids decomposition, as high cross-coupling yields are still achieved.

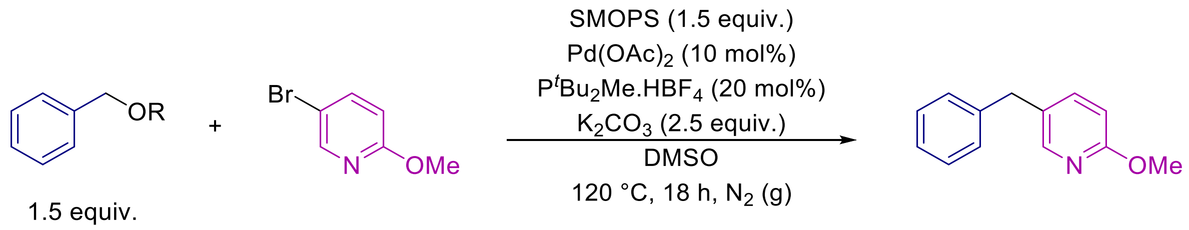

Table S14. Investigation into the utility of various benzyl alcohol derivatives.

Entry[a]	R group	Yield /%
1	H	0
2	COOMe	22
3	Ms	70[b]
4	Ts	98[b]

[a] Reactions conditions: benzyl alcohol derivative (0.3 mmol, 1.5 equiv.), (heteroaryl)bromide (0.2 mmol, 1.0 equiv.), SMOPS sulfinate reagent (0.3 mmol, 1.5 equiv.), $Pd(OAc)_2$ (10 mol%), $P^tBu_2Me.HBF_4$ (20 mol%), K_2CO_3 (0.5 mmol, 2.5 equiv.), DMSO (0.13 M, 1.5 mL). [b] Yield from cross-coupling to 3-bromoquinoline.

5. HPLC calibration curves

5.1 General method

A 0.04 M stock solution of the substrate in DMSO and a 0.04 M stock solution of *p*-tolyl ether (used as the internal standard (I.S.)) in DMSO were prepared. Using a 100 µL glass syringe, six different ratios of these solutions were mixed in six different vials which were topped up with 250 µL DMSO (Table 15). Then, 50 µL of each final solution was placed into a HPLC vial and topped up with MeOH.

Table S15.

Volume substrate solution /µL	Volume internal standard solution /µL	Substrate/internal standard ratio
10	100	0.1
20	80	0.25
40	60	0.67
60	40	1.5
80	20	4
100	10	10

A linear relationship between the obtained substrate and internal standard HPLC peak areas and the substrate/I.S. ratio was calculated using Microsoft Excel. An equation of the type $y = mx + c$ can be generated from a plot of this data, and the calibration curve can thus be used to determine the mols of substrate. Once the mols, and subsequent yield was determined for each wavelength, the average of the three was taken.

5.2 Monitoring the one-pot reaction profile

Calibration curves were made for the βECN sulfone, SMOPS sulfone, potassium benzyl sulfinate salt and 5-benzyl-2-methoxypyridine as described in the general method (Scheme 2). The formation and consumption of each, during the one-pot desulfinative cross-coupling process, was monitored by HPLC at regular time intervals.

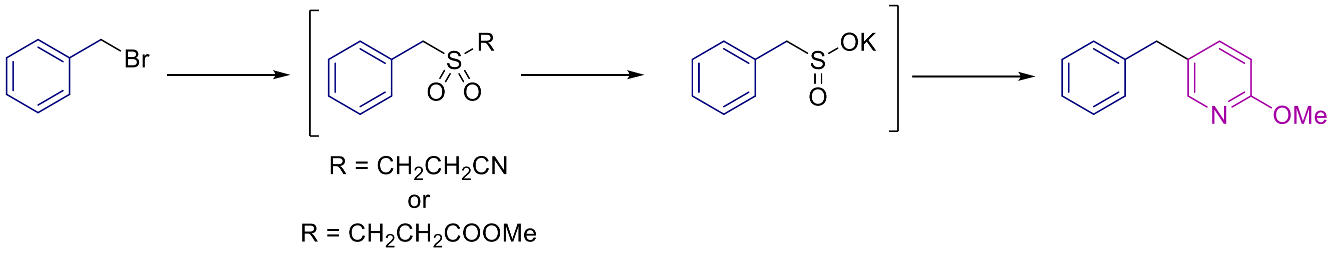

Scheme 2. Calibration curves were made for each intermediate and the final product of the reaction and their respective formation and consumption was monitored and quantified. Reactions conditions: benzyl bromide (0.3 mmol, 1.5 equiv.), 5-benzyl-2-methoxypyridine (0.2 mmol, 1.0 equiv.), sulfinate reagent (0.3 mmol, 1.5 equiv.), $Pd(OAc)_2$ (10 mol%), $P^tBu_2Me.HBF_4$ (20 mol%), K_2CO_3 (0.5 mmol, 2.5 equiv.), solvent (0.13 M, 1.5 mL).

3-(Benzylsulfonyl)propanenitrile

Table S16. Calibration curve data for 3-(benzylsulfonyl)propanenitrile.

Substrate/internal standard ratio	Substrate peak area/I.S. peak area at 254 nm	Substrate peak area/I.S. peak area at 210 nm	Substrate peak area/I.S. peak area at 230 nm
0.1	0.024	0.048	0.008
0.25	0.029	0.122	0.022
0.67	0.076	0.341	0.070
1.5	0.136	0.627	0.130
4	0.367	1.727	0.329
10	0.916	4.189	0.832

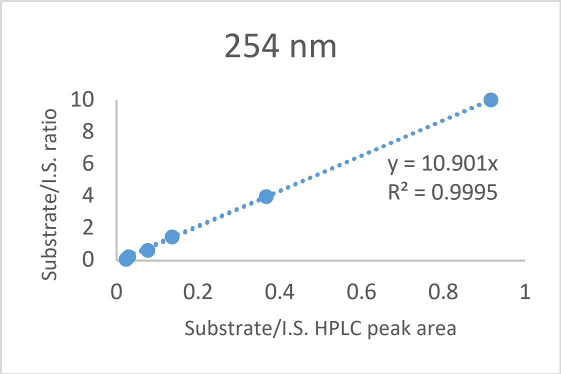

$$\frac{Area_{Substrate}}{Area_{I.S.}} \times mols_{I.S.} \times 10.901 = mols_{Substrate}$$

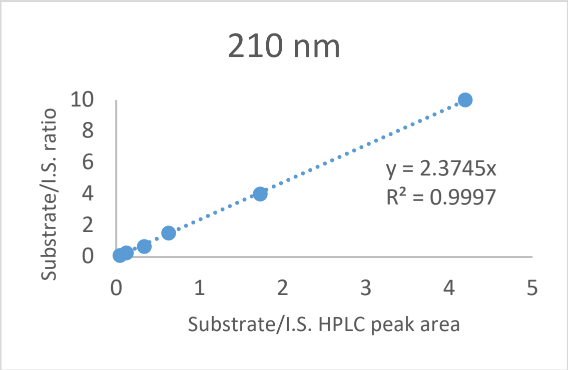

$$\frac{Area_{Substrate}}{Area_{I.S.}} \times mols_{I.S.} \times 2.3745 = mols_{Substrate}$$

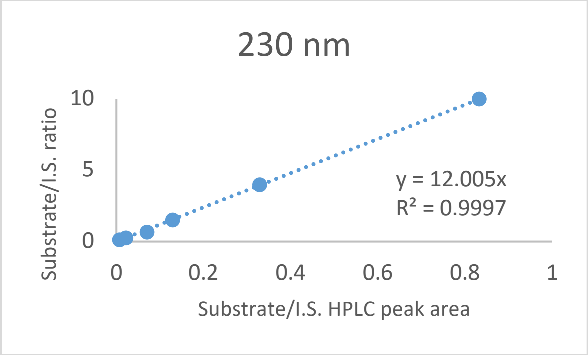

$$\frac{Area_{Substrate}}{Area_{I.S.}} \times mols_{I.S.} \times 12.005 = mols_{Substrate}$$

Methyl 3-(benzylsulfonyl)propanoate

Table S17. Calibration curve data for methyl 3-(benzylsulfonyl)propanoate.

Substrate/internal standard ratio	Substrate peak area/I.S. peak area at 254 nm	Substrate peak area/I.S. peak area at 210 nm	Substrate peak area/I.S. peak area at 230 nm
0.1	0.024	0.045	0.010
0.25	0.026	0.108	0.022
0.67	0.065	0.282	0.054
1.5	0.145	0.634	0.125
4	0.376	1.693	0.331
10	0.954	4.112	0.829

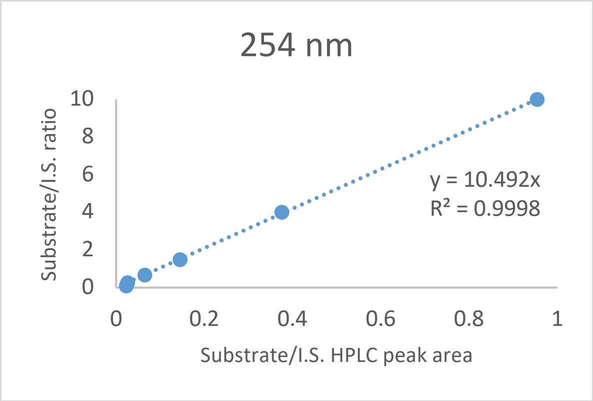

$$\frac{Area_{Sulfone}}{Area_{I.S.}} \times mols_{I.S.} \times 10.492 = mols_{Sulfone}$$

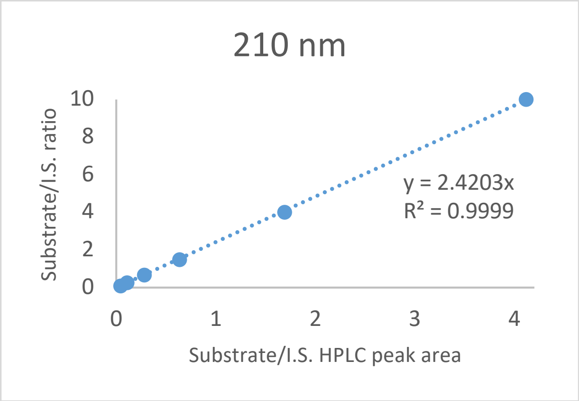

$$\frac{Area_{Sulfone}}{Area_{I.S.}} \times mols_{I.S.} \times 2.4203 = mols_{Sulfone}$$

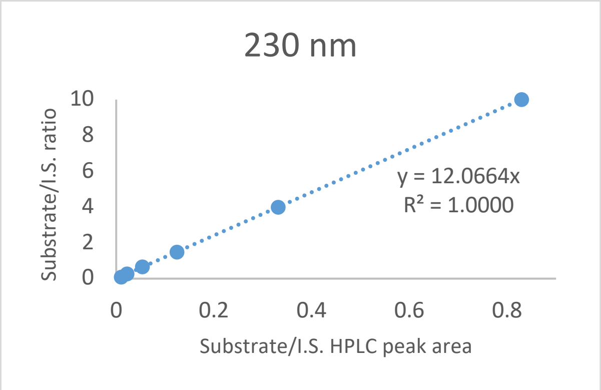

$$\frac{Area_{Sulfone}}{Area_{I.S.}} \times mols_{I.S.} \times 12.066 = mols_{Sulfone}$$

Potassium benzyl sulfinate salt

Table S18. Calibration curve data for potassium benzyl sulfinate salt.

Substrate/internal standard ratio	Substrate peak area/I.S. peak area at 254 nm	Substrate peak area/I.S. peak area at 210 nm	Substrate peak area/I.S. peak area at 230 nm
0.1	0.044	0.021	0.022
0.25	0.116	0.053	0.054
0.67	0.346	0.160	0.169
1.5	0.845	0.384	0.400
4	2.237	1.013	1.062
10	5.434	2.442	2.600

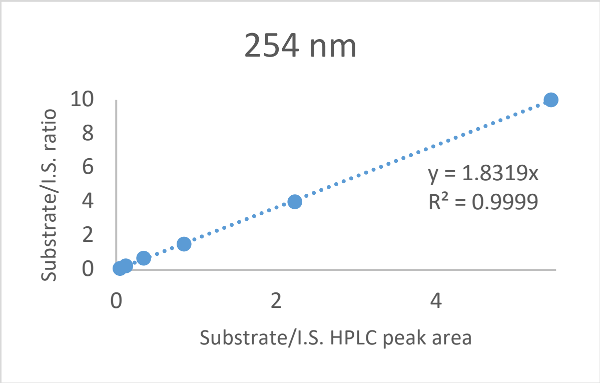

$$\frac{Area_{Sulfinate}}{Area_{I.S.}} \times mols_{I.S.} \times 1.8319 = mols_{Sulfinate}$$

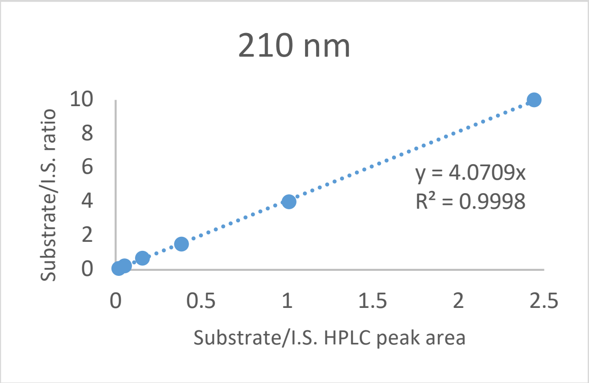

$$\frac{Area_{Sulfinate}}{Area_{I.S.}} \times mols_{I.S.} \times 4.0709 = mols_{Sulfinate}$$

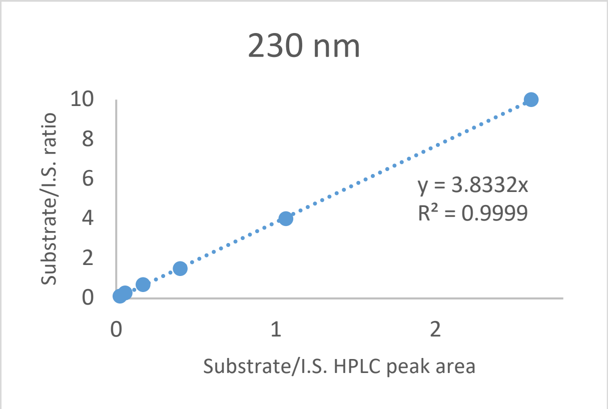

$$\frac{Area_{Sulfinate}}{Area_{I.S.}} \times mols_{I.S.} \times 3.8332 = mols_{Sulfinate}$$

5-Benzyl-2-methoxypyridine

Table S19. Calibration curve data for 5-benzyl-2-methoxypyridine.

Substrate/internal standard ratio	Substrate peak area/I.S. peak area at 254 nm	Substrate peak area/I.S. peak area at 210 nm	Substrate peak area/I.S. peak area at 230 nm
0.1	0.036	0.052	0.032
0.25	0.113	0.143	0.126
0.67	0.317	0.394	0.349
1.5	0.669	0.843	0.741
4	1.914	2.389	2.148
10	4.846	6.242	5.627

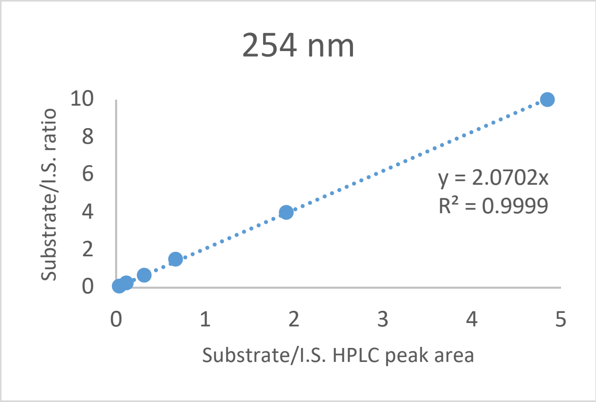

$$\frac{Area_{Product}}{Area_{I.S.}} \times mols_{I.S.} \times 2.0702 = mols_{Product}$$

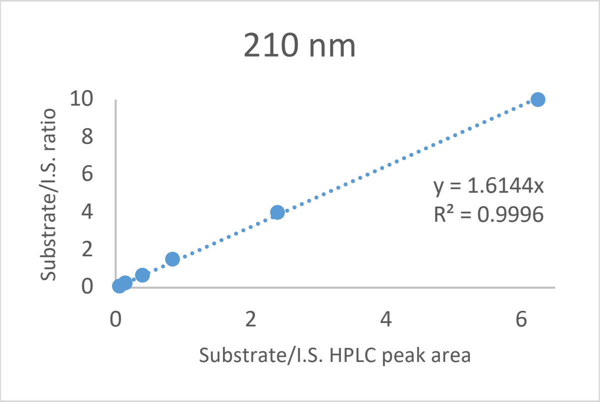

$$\frac{Area_{Product}}{Area_{I.S.}} \times mols_{I.S.} \times 1.6144 = mols_{Product}$$

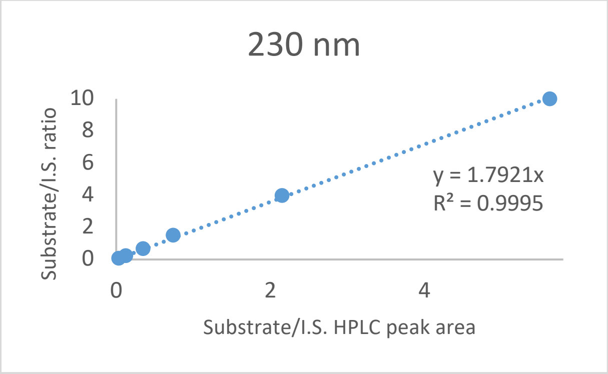

$$\frac{Area_{Product}}{Area_{I.S.}} \times mols_{I.S.} \times 1.7921 = mols_{Product}$$

SUPPORTING INFORMATION

In the βECN reaction, the generation of the sulfone intermediate *via* an S_N2 reaction must have occurred within 5 minutes at 120 °C, as this was already in high yield when the first sample was taken (Figure 1). After 1 hour, the sulfone was almost completely consumed, illustrating that a slow release mechanism cannot be at play. The product had reached an 80% yield after only 2 hours, with quantitative yield observed after 24 hours.

In the SMOPS reaction, alike to the βECN, the formation and consumption of the sulfone intermediate was fast, but the sulfinate consumption seemed to be much quicker. After only 1.5 hours, the product had already reached an 88% yield and quantitative yield was observed after 3 hours.

At least for this particular substrate, using SMOPS in place of the βECN sulfinate is beneficial not only due to it’s commercial availability, but also due to the quicker reaction time.

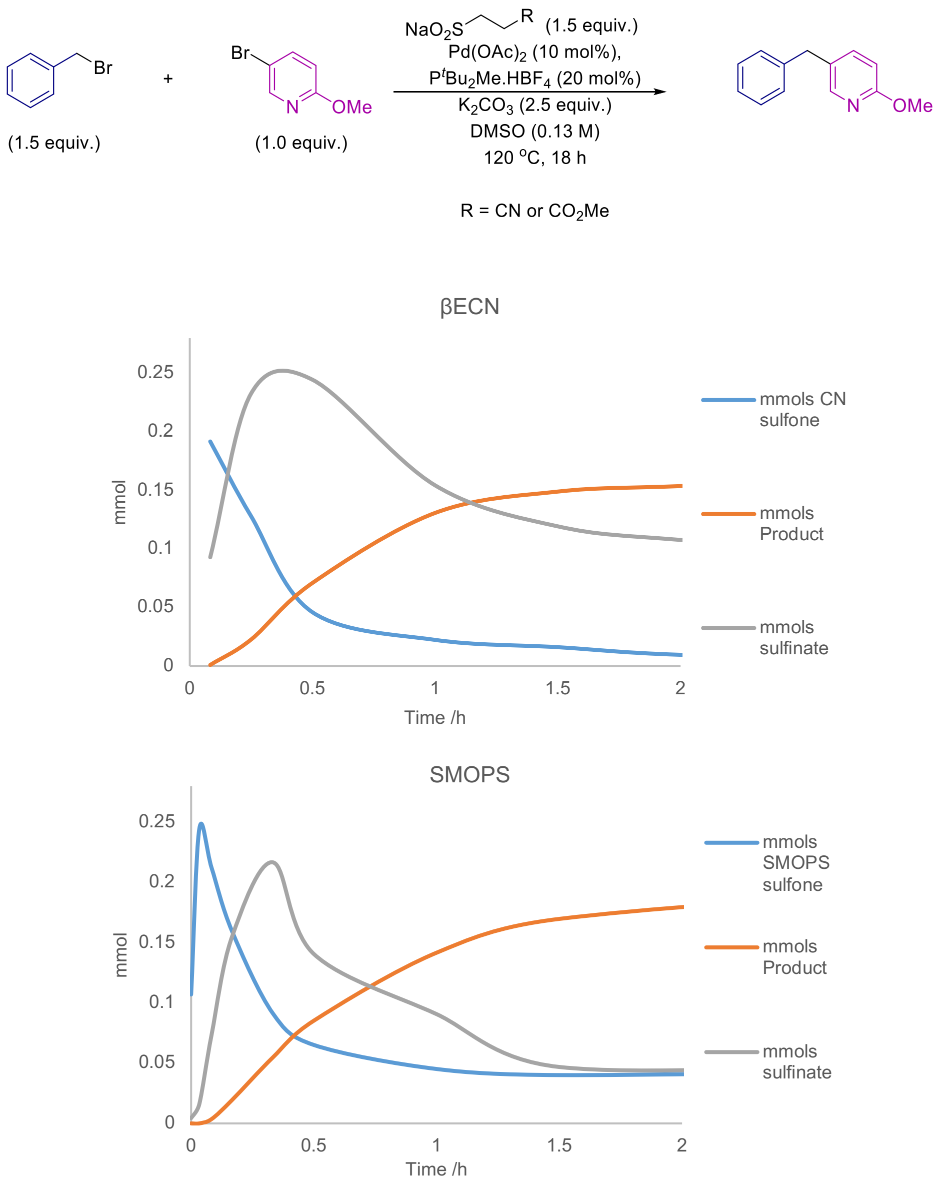

Figure 1. Comparison study between the desulfinative cross-coupling utilising either the βECN or SMOPS sulfinate reagent.

6. Synthesis of starting materials

6.1 Sulfinate reagent synthesis

Sodium ((3,4,5-trimethoxybenzoyl)oxy)methanesulfinate (Rongacyl) (S1)

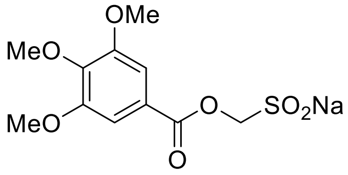

Procedure according to A. Shavnya et al.[1]

Sodium hydroxymethylsulfinate dihydrate (Rongalite®) (9.25 g, 60.00 mmol, 1.4 equiv.) was dissolved in aqueous NaOH (52 mL, 1.0 M, 1.2 equiv.) and the clear solution was kept without stirring at room temperature for 90 min. Aqueous NaBr (25 mL, 3.9 M) was added and the resulting solution was cooled to 0 °C under stirring, before addition of 3,4,5-trimethoxybenzoyl chloride (10.0 g, 43.35 mmol, 1.0 equiv.) was added in one portion. The heavy slurry was warmed under stirring to room temperature over 1 hour and was stirred for 3 further hours at this temperature (a thin slurry formed). NaBr (32.5 g) was added and the mixture was stirred for 1 hour. The solid was filtered off and washed with aqueous NaBr (2 × 40 mL, 3.9 M) and EtOAc (2 × 40 mL), then dried on the filter and then under vacuum (< 1 mbar) for 18 hours to obtain the title compound as a white solid (9.10 g, 67%).

^{1}H NMR (400 MHz, DMSO-d$_6$) δ 7.24 (s, 2H, 2 × Ar-*H*), 4.12 (s, 2H, OC*H*$_2$-SO$_2$Na), 3.83 (s, 6H, 2 × ArOC*H*$_3$), 3.73 (s, 3H, ArOC*H*$_3$); **^{13}C NMR** (101 MHz, DMSO-d$_6$) δ 164.8, 152.8, 141.7, 124.9, 106.6, 85.5, 60.2, 56.1; **HRMS** (ESI, m/z) calculated for $C_{11}H_{13}O_7S$, [M-Na]$^-$ is 289.0387, found 289.0387.

Data is in accordance with the literature.[1]

Sodium 2-cyanoethane-1-sulfinate (β-nitrile sulfinate) (S2)

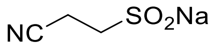

A modified procedure from Wang et al.[5]

CAUTION: Exothermic and Evolution of $H_{2\,(g)}$.

To a 250 mL round-bottom flask, fitted with a $CaCl_2$ drying tube and stirrer bar, anhydrous methanol (80 mL) was added. The vessel was placed in an ice-water bath before hexane washed sodium (0.92 g, 40.00 mmol, 1.0 equiv.) was added, in four equal sized pieces, in one portion. The reaction was stirred until all the sodium had dissolved to give a clear, colourless solution. The 3,3'-sulfonyldipropionitrile (6.89 g, 40.00 mmol, 1.0 equiv.) was added with quick removal and replacement of the drying tube from the round-bottom flask. The ice-water bath was removed, and the mixture left to stir for 2 hours. The excess methanol was removed under vacuum and the residue was dried by forming an azeotrope with toluene. The crude residue was triturated with EtOAc (100 mL) and stirred for 2 hours before filtration. The solid was washed with EtOAc (200 mL) and Et_2O (200 mL). The title product was afforded as a white crystalline powder (5.32 g, 94% yield).

^{1}H NMR (400 MHz, MeOD) δ 2.69 (td, J = 7.5, 0.5 Hz, 2H, C*H*$_2$), 2.48 – 2.40 (m, 2H, C*H*$_2$); **^{13}C NMR** (101 MHz, MeOD) δ 120.7, 56.0, 10.1; **MP** (EtOAc) 175 °C; **IR** (neat)/ν_{max} 3391, 2160, 2030, 1658, 1417, 1026, 1009, 749; **HRMS** (ESI, m/z) calculated for $C_3H_4O_2NSNa$, [M-Na]$^-$ is 117.9968, found 117.9967.

6.2 Sulfone synthesis

6.2.1 General Procedure A

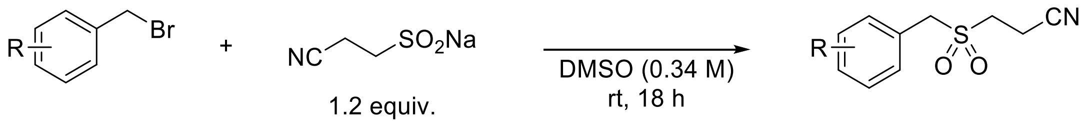

A mixture of the benzyl bromide (3.00 mmol, 1.0 equiv.), sodium 2-cyanoethane-1-sulfinate (0.508 g, 3.60 mmol, 1.2 equiv.) and DMSO (8.8 mL, 0.34 M) was stirred at room temperature for 18 hours. The reaction mixture was transferred to a 100 mL separating funnel in EtOAc (20 mL). The organic layer was washed with aqueous LiCl solution (5 mol%, 3 × 10 mL). The organic layer was dried over $MgSO_4$ and then concentrated under reduced pressure. The crude residue was then purified by column chromatography (EtOAc in petrol) to yield the sulfone product.

6.2.2 General Procedure B

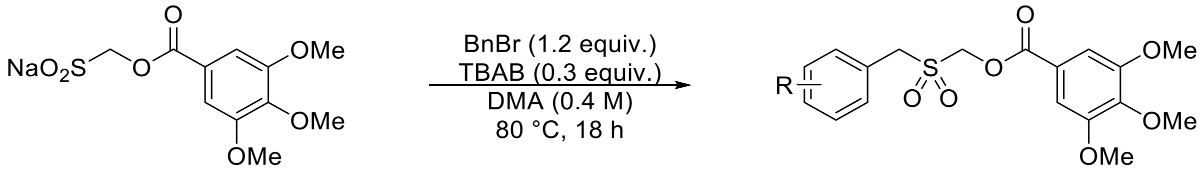

Modified from a procedure by A. Shavnya et al.[1]

A mixture of Rongacyl (1.69 g, 5.40 mmol, 1.0 equiv.), alkyl halide (6.50 mmol, 1.2 equiv.), TBAB (0.522 g, 1.62 mmol, 0.3 equiv.), and DMAc (13.5 mL, 0.4 M) was stirred at 80 °C for 18 hours. The reaction was cooled to room temperature, quenched with half-saturated $NaHCO_3$ (20 mL) and extracted with EtOAc (3 × 20 mL). The combined organic extracts were dried over $MgSO_4$, concentrated under reduced pressure and the crude residue was purified by column chromatography (EtOAc in petrol) to yield the sulfone product.

3-(Benzylsulfonyl)propanenitrile (3a)

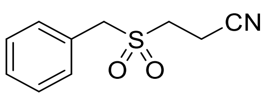

General procedure A was followed using benzyl bromide (357 µL, 3.00 mmol, 1.0 equiv.), β-nitrile sulfinate (0.508 g, 3.60 mmol, 1.2 equiv.) and DMSO (8.8 mL, 0.34 M). The crude residue was purified by column chromatography (30 – 60% EtOAc in petrol) to give the product as a white crystalline solid (601.9 mg, 96% yield).

^{1}H NMR (400 MHz, $CDCl_3$) δ 7.44 (s, 5H, 5 × Ar-*H*), 4.35 (s, 2H, ArC*H*$_2$), 3.13 (t, *J* = 7.5 Hz, 2H, C*H*$_2$CH$_2$CN), 2.78 (t, *J* = 7.5 Hz, 2H, CH$_2$C*H*$_2$CN); **^{13}C NMR** (101 MHz, $CDCl_3$) δ 130.8, 129.7, 129.5, 127.1, 116.7, 60.6, 46.2, 11.3; **MP** (CH_2Cl_2) 110 °C; **HRMS** (ESI, *m/z*) calculated for $C_{10}H_{11}O_2NS$, $[M+Na]^+$ is 232.0403, found 232.0404.

The data is in accordance with the literature.[6]

((4-Methoxybenzyl)sulfonyl)methyl 3,4,5-trimethoxybenzoate (3b)

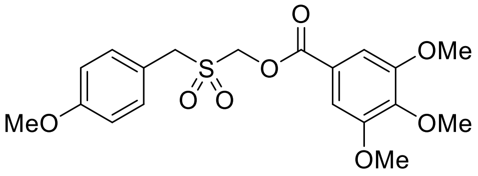

General procedure B was followed, using 4-methoxybenzyl bromide (0.94 mL, 6.50 mmol, 1.2 equiv.). The crude residue was purified by column chromatography (15 – 70% EtOAc in Petrol) to give the title product as a white solid (727.3 mg, 33% yield).

^{1}H NMR (400 MHz, $CDCl_3$) δ δ 7.39 – 7.36 (m, 2H, 2 × Ar-*H*), 7.33 (s, 2H, Ar-*H*), 6.92 – 6.89 (m, 2H, Ar-*H*), 5.17 (s, 2H, SO_2CH_2), 4.30 (s, 2H, $ArCH_2$), 3.94 (s, 3H, $ArOCH_3$), 3.93 (s, 6H, $Ar(OCH_3)_2$), 3.79 (s, 3H, $ArOCH_3$); **^{13}C NMR** (101 MHz, $CDCl_3$) δ 164.4, 160.5, 153.3, 143.6, 132.2, 122.6, 118.7, 114.7, 107.7, 73.1, 61.2, 58.4, 56.5, 55.4; **MP** (EtOH) 100 – 101 °C; **IR** (neat)/ν_{max} 2981, 2839, 1732, 1588, 1341, 1126; **HRMS** (ESI, *m/z*) calculated for $C_{19}H_{22}O_8SNa$, $[M+Na]^+$ is 433.0928, found 433.0925.

3-((4-(Trifluoromethyl)benzyl)sulfonyl)propanenitrile (3c)

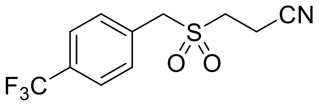

General procedure A was followed using 4-(trifluoromethyl)benzyl bromide (478 mg, 2.00 mmol, 1.0 equiv.), β-nitrile sulfinate (336 mg, 2.40 mmol, 1.2 equiv.) and DMSO (6 mL, 0.34 M). The crude residue was purified by column chromatography (30 – 60% EtOAc in petrol) to give the product as a white solid (511.3 mg, 92% yield).

^{1}H NMR (400 MHz, DMSO-d_6) δ 7.80 (d, *J* = 8.0 Hz, 2H, 2 × Ar-*H*), 7.64 (d, *J* = 8.0 Hz, 2H, 2 × Ar-*H*), 4.74 (s, 2H, $ArCH_2$), 3.51 (t, *J* = 7.0 Hz, 2H, CH_2CH_2CN), 3.03 (t, *J* = 7.0 Hz, 2H, CH_2CH_2CN); **^{13}C NMR** (101 MHz, DMSO-d_6) δ 132.7, 132.0, 129.1 (q, *J* = 32.0 Hz), 125.5 (q, *J* = 4.0 Hz), 124.1 (q, *J* = 273.5 Hz), 118.2, 57.2, 46.6, 10.6; **^{19}F NMR** (377 MHz, DMSO-d_6) δ -61.20; **MP** ($CDCl_3$) 104 °C; **IR** (neat)/ν_{max} 3052, 2938, 1420, 1338, 1135, 1124; **HRMS** (ESI, *m/z*) calculated for $C_{11}H_9O_2NF_3S$ $[M-H]^-$ is 276.0312 (100%), 277.0344 (10%) found 276.0306 (100%), 277.0338 (10%).

3-(((2-Cyanoethyl)sulfonyl)methyl)benzonitrile (3d)

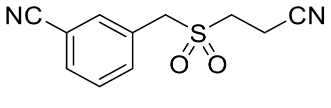

General procedure A was followed using 3-(bromomethyl)benzonitrile (294 mg, 1.50 mmol, 1.00 equiv.), β-nitrile sulfinate (254 mg, 1.80 mmol, 1.2 equiv.) and DMSO (4.4 mL, 0.34 M) to give the product as a white solid (324.6 mg, 92% yield).

^{1}H NMR (400 MHz, $CDCl_3$) δ 7.79 – 7.70 (m, 3H, 3 × Ar-*H*), 7.58 (td, *J* = 7.5, 0.7 Hz, 1H, Ar-*H*), 4.40 (s, 2H, $ArCH_2$), 3.20 (t, *J* = 7.5 Hz, 2H, CH_2CH_2CN), 2.93 (t, *J* = 7.5 Hz, 2H, CH_2CH_2CN); **^{13}C NMR** (101 MHz, $CDCl_3$) δ 135.3, 134.3, 133.3, 130.3, 128.5, 117.9, 116.6, 113.9, 59.5, 47.1, 11.7; **MP** (CH_2Cl_2) 118 °C; **IR** (neat)/ν_{max} 3065, 2922, 2233, 1420, 1318, 1124, 1029; **HRMS** (ESI, *m/z*) calculated for $C_{11}H_{10}O_2N_2S$, $[M+Na]^+$ is 257.0355, found 257.0357.

6.3 Sulfinate synthesis

6.3.1 General Procedure C

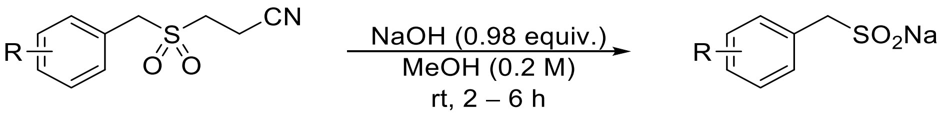

The sulfone made from general procedure A (1.0 equiv.), NaOH (0.98 equiv.) and methanol (0.2 M) were added to a round-bottom flask equipped with a stirrer bar. The reaction was stirred at room temperature until complete consumption of the sulfone (2 – 6 hours), through monitoring by TLC. The solvents were removed under reduced pressure, and the residue was dried by forming an azeotrope with toluene to yield the benzyl sulfinate as a sodium salt.

6.3.2 General Procedure D

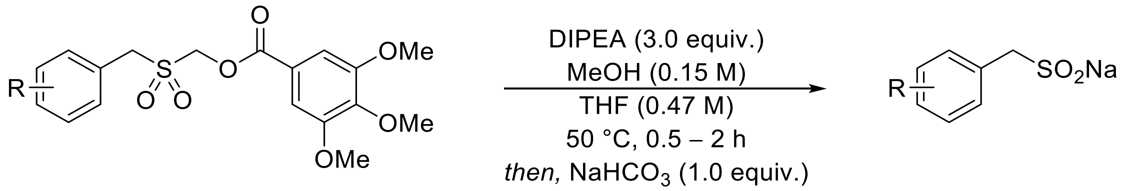

Modified from a procedure by A. Shavnya et al.[24]
The sulfone made from general procedure B (1.18 mmol, 1.0 equiv.), DIPEA (0.64 mL, 3.54 mmol, 3.0 equiv.), MeOH (7.6 mL, 0.16 M), and THF (2.5 mL, 0.47 M) were combined in a reaction flask. The initial suspension was stirred at 50 °C for 0.5 – 2 h until the starting material appeared to be consumed from monitoring by TLC; the reaction mixture turned to a clear solution. The reaction was cooled to room temperature, treated with aq. $NaHCO_3$ (1.18 mL, 1 M, 1.0 equiv.), concentrated, and the residue was dried by forming an azeotrope with toluene. MTBE (5 mL) was added to the crude residue, the mixture sonicated, before solid being allowed to settle to the bottom of the vessel. The organic layer was then removed by pipette to extract the methyl 3,4,5-trimethoxybenzoate by-product. This process was repeated four times. The final residue was dried under vacuum to yield the purified benzyl sulfinate as a sodium salt.

Sodium benzylsulfinate (4a)

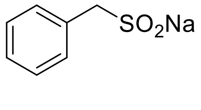

General procedure C was followed using 3-(benzylsulfonyl)propanenitrile (167 mg, 0.80 mmol, 1.0 equiv.), NaOH (30.4 mg, 0.76 mmol, 0.95 equiv.) and methanol (4 mL, 0.2 M) to give the product as a fine white powder (71.2 mg, 81% yield).

^{1}H NMR (400 MHz, MeOD) δ 7.29 – 7.25 (m, 4H, 4 × Ar-*H*), 7.24 – 7.17 (m, 1H, Ar-*H*), 3.51 (s, 2H, ArC*H*$_2$); **^{13}C NMR** (101 MHz, MeOD) δ 134.7, 131.0, 129.2, 127.7, 70.9; **MP** (MTBE) 250 °C (decomp.); **IR** (neat)/ν_{max} 3030, 1601, 1454, 1337, 1187, 1131, 1027; **HRMS** (ESI, m/z) calculated for $C_7H_7O_2S$, $[M-Na]^-$ is 155.0172, found 155.0171.

The data is in accordance with the literature.[7]

Sodium (4-methoxy)benzylsulfinate (4b)

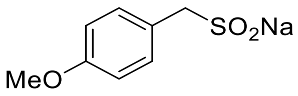

General procedure D was followed using ((4-methoxybenzyl)sulfonyl)methyl 3,4,5-trimethoxybenzoate (488 mg, 1.18 mmol, 1.0 equiv.) to give the title product as a fine white powder (202.9 mg, 83% yield).

^{1}H NMR (400 MHz, MeOD) δ 7.22 – 7.16 (m, 2H, 2 × Ar-*H*), 6.87 – 6.82 (m, 2H, 2 × Ar-*H*), 3.76 (s, 3H, ArOC*H*$_3$), 3.45 (s, 2H, ArC*H*$_2$); **^{13}C NMR** (101 MHz, MeOD) δ 160.2, 132.0, 126.8, 114.7, 69.9, 55.6; **MP** (MTBE) 265 °C (decomp.); **IR** (neat)/ν_{max} 2981, 1611, 1511, 1249, 1013, 973; **HRMS** (ESI) calculated for $C_8H_9O_3S$, [M-Na]$^-$ is 185.0278, found 185.0275.

The data is in accordance with the literature.[8]

Sodium 4-(trifluoromethyl)benzylsulfinate (4c)

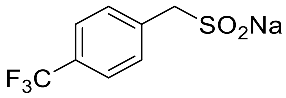

General procedure C was followed using 3-((4-(trifluoromethyl)benzyl)sulfonyl)propanenitrile (368 mg, 1.33 mmol, 1.0 equiv.), NaOH (52 mg, 0.98 mmol, 0.98 equiv.) and methanol (6.7 mL, 0.2 M) to give the product as a fine white powder (264.9 mg, 81% yield).

^{1}H NMR (400 MHz, MeOD) δ 7.51 (dd, *J* = 55.5, 8.0 Hz, 4H, 4 × Ar-*H*), 3.59 (s, 2H, ArC*H*$_2$); **^{13}C NMR** (101 MHz, MeOD) δ 139.3, 131.5, 129.7 (q, *J* = 33.5 Hz), 126.0 (q, *J* = 3.5 Hz), 124.6 (q, *J* = 124.5 Hz), 70.2; **^{19}F NMR** (377 MHz, CD_3OD) δ -63.89; **MP** (MTBE) 210 – 215 °C (decomp.); **IR** (neat)/ν_{max} 2161, 2024, 1327, 1118, 1068, 966; **HRMS** (ESI, *m/z*) calculated for $C_8H_6O_2F_3S$, [M-Na]$^-$ is 223.0046, found 223.0044.

Sodium (3-cyanophenyl)methanesulfinate (4d)

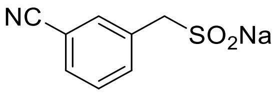

General procedure C was followed using 3-(((2-cyanoethyl)sulfonyl)methyl)benzonitrile (281 mg, 1.20 mmol, 1.0 equiv.), sodium hydroxide (47.2 mg, 1.18 mmol, 0.98 equiv.) and MeOH (6 mL, 0.2 M), to give the title product as a white powder (201.3 mg, 84% yield).

^{1}H NMR (400 MHz, MeOD) δ 7.64 – 7.60 (m, 1H, Ar-*H*), 7.59 – 7.54 (m, 2H, 2 × Ar-*H*), 7.47 (t, *J* = 7.5 Hz, 1H, Ar-*H*), 3.55 (s, 2H, ArC*H*$_2$); **^{13}C NMR** (101 MHz, MeOD) δ 136.5, 135.8, 134.5, 131.2, 130.2, 119.9, 113.1, 69.4; **MP** (MTBE) 220 °C (Decomp); **IR** (neat)/ν_{max} 2230, 1976, 1625, 1215, 1178, 1153, 1035, 1009; **HRMS** (ESI, *m/z*) calculated for $C_8H_6O_2NSNa$, [M-Na]$^-$ is 180.0125, found 180.0123.

6.4 Benzyl tosylate synthesis

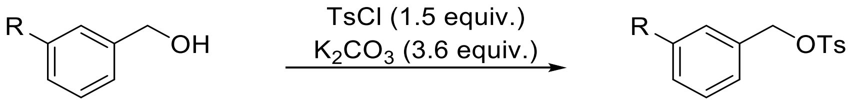

A mortar was charged with K_2CO_3 (4.97 g, 36.00 mmol, 3.6 equiv.), the benzyl alcohol (10.00 mmol, 1.0 equiv.) and tosyl chloride (2.86 g, 15.00 mmol, 1.5 equiv.). The reagents were ground together vigorously with a pestle for 5 minutes. The consistency of the reaction mixture changed. KOH (2.81 g, 50.00 mmol, 5.0 equiv.) was added and vigorous grinding occurred for a further 3 minutes. Et_2O (100 mL) was added, the mixture was stirred before vacuum filtration through a Buchner funnel. The solid residue was transferred to a vial before drying under vacuum (< 1 mbar) until constant mass was achieved. The resulting white solid tosylate was used directly in the cross-coupling reactions without any further purification.

Note: The tosylates were stored under nitrogen and in the freezer to prevent decomposition. *(At ambient temperature, the white solid darkens within an hour and becomes amorphous overnight)*

Benzyl 4-methylbenzenesulfonate (S3)

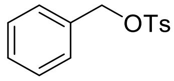

The general procedure was followed using benzyl alcohol (1.03 mL, 10.00 mmol, 1.0 equiv.). The title product was given as a white crystalline solid (2.06 g, 78% yield).

^{1}H NMR (400 MHz, $CDCl_3$) δ 7.76 – 7.71 (m, 2H, 2 × Ar-*H*), 7.29 – 7.22 (m, 5H, 5 × Ar-*H*), 7.21 – 7.17 (m, 2H, 2 × Ar-*H*), 4.99 (s, 2H, ArC*H*$_2$), 2.38 (s, 3H, Ar-C*H*$_3$); **^{13}C NMR** (101 MHz, $CDCl_3$) δ 144.9, 133.4, 133.4, 129.9, 129.1, 128.8, 128.6, 128.1, 72.0, 21.7; **MP** (Et_2O) 48 – 50 °C.

The data is consistent with the literature.[9]

3-Methoxybenzyl 4-methylbenzenesulfonate (S4)

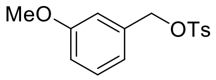

The general procedure was followed using 3-methoxybenzyl alcohol (1.24 mL, 10.00 mmol, 1.0 equiv.). The title product was given as a white crystalline solid (1.17 g, 40% yield).

^{1}H NMR (400 MHz, $CDCl_3$) δ 7.86 – 7.78 (m, 2H, 2 × Ar-*H*), 7.39 – 7.32 (m, 2H, 2 × Ar-*H*), 7.28 – 7.22 (m, 1H, Ar-*H*), 6.91 – 6.82 (m, 2H, 2 × Ar-*H*), 6.79 (dd, *J* = 2.5, 1.5 Hz, 1H, Ar-*H*), 5.05 (s, 2H, ArC*H*$_2$), 3.79 (s, 3H, OC*H*$_3$), 2.47 (s, 3H, Ar-C*H*$_3$); **^{13}C NMR** (101 MHz, $CDCl_3$) δ 159.9, 144.9, 134.9, 133.4, 130.0, 129.8, 128.1, 120.8, 114.9, 113.8, 71.9, 55.4, 21.8; **MP** (Et_2O) 68 - 70 °C; **IR** (neat)/ν_{max} 2957, 1440, 1316, 1265, 1169, 1094, 929.

The data is consistent with the literature.[9]

7.0 Desulfinative cross-coupling products

7.1 General procedure E

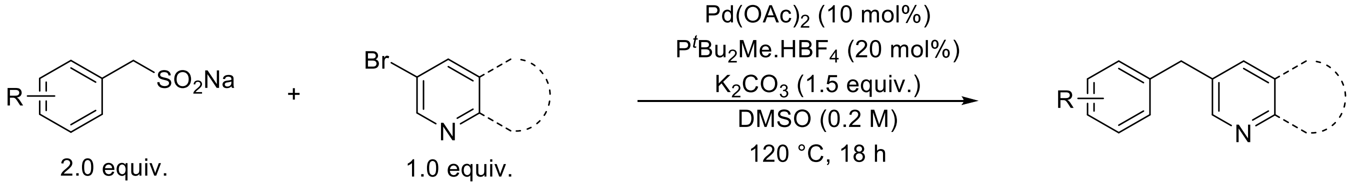

To a 10 mL microwave vial equipped with a stirrer bar was added, the sodium benzyl sulfinate (0.40 mmol, 2.0 equiv.), *if solid*, the (hetero)aryl bromide (0.20 mmol, 1.0 equiv.), potassium carbonate (41.5 mg, 0.30 mmol, 1.5 equiv.), $Pd(OAc)_2$ (4.5 mg, 10 mol%) and $P^tBu_2Me\cdot HBF_4$ (9.9 mg, 20 mol%). The vial was sealed with a microwave vial cap, evacuated under vacuum (<1 mbar) and back-filled with nitrogen gas. This cycle was repeated a further two times. Addition of, *if liquid*, the heteroaryl bromide (0.20 mmol, 1.0 equiv.), and DMSO (1.0 mL, 0.2 M) followed. The reaction was heated to 120 °C for 18 h before being cooled to room temperature. The reaction mixture was filtered through a pad of celite, rinsed thoroughly with EtOAc (50 mL) and the solvents were removed under reduced pressure. If the product was highly polar, 15 mL EtOAc was added to the crude residue and this was then washed with 5% aqueous LiCl solution (3 × 8 mL) to draw the DMSO into the aqueous layer. The aqueous washings were discarded. The organic layer was dried over $MgSO_4$, before removal of the solvents under reduced pressure. The resulting crude material was purified by silica gel chromatography to give the desired cross-coupled product.

7.2 General procedure F

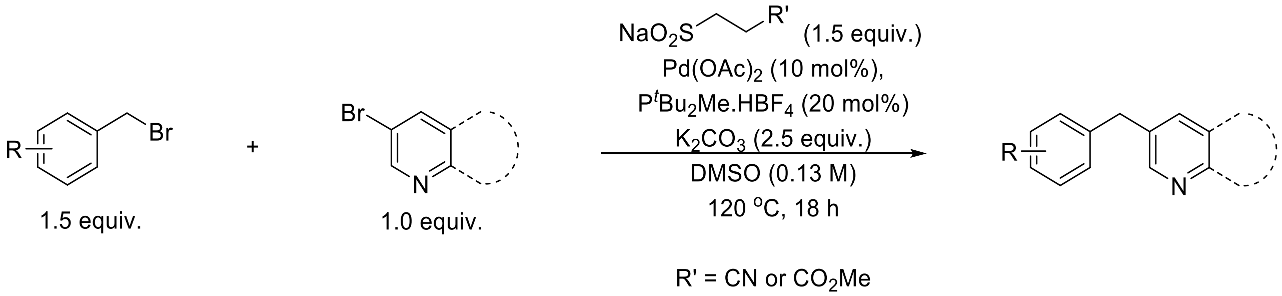

To a 10 mL microwave vial equipped with a stirrer bar was added, *if solid*, the benzyl bromide (0.30 mmol, 1.5 equiv.), *if solid*, the (hetero)aryl bromide (0.20 mmol, 1.0 equiv.), SMOPS (52.2 mg, 0.30 mmol, 1.5 equiv.) or β-nitrile sulfinate (42.3 mg, 0.30 mmol, 1.5 equiv.), potassium carbonate (69.1 mg, 0.50 mmol, 2.5 equiv.), $Pd(OAc)_2$ (4.5 mg, 10 mol%) and $P^tBu_2Me\cdot HBF_4$ (9.9 mg, 20 mol%). The vial was sealed with a microwave vial cap, evacuated under vacuum (<1 mbar) and back-filled with nitrogen gas. This cycle was repeated a further two times. Addition of, *if liquid*, the heteroaryl bromide (0.20 mmol, 1.0 equiv.), *if liquid*, the benzyl bromide (0.30 mmol, 1.5 equiv.) and DMSO (1.5 mL, 0.13 M) followed. The reaction was heated to 120 °C for 18 h before being cooled to room temperature. The reaction mixture was filtered through a pad of celite, rinsed thoroughly with EtOAc (50 mL) and the solvents were removed under reduced pressure. If the product was highly polar, 15 mL EtOAc was added to the crude residue and this was then washed with 5% aqueous LiCl solution (3 × 8 mL) to draw the DMSO into the aqueous layer. The aqueous washings were discarded. The organic layer was dried over $MgSO_4$, before removal of the solvents under reduced pressure. The resulting crude material was purified by silica gel chromatography to give the desired cross-coupled product.

3-Benzylquinoline (5a)

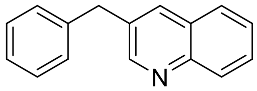

General procedure E was followed using sodium benzyl sulfinate (71.3 mg, 0.40 mmol, 2.0 equiv.) and 3-bromoquinoline (27 µL, 0.20 mmol, 1.0 equiv.). The crude material was purified by column chromatography (10 – 30% EtOAc in petrol) to give the product as an off-white solid (38.1 mg, 87%).

General procedure F was followed using benzyl bromide (36 µL, 0.30 mmol, 1.5 equiv.), 3-bromoquinoline (27 µL, 0.20 mmol, 1.0 equiv.) and SMOPS (52.2 mg, 0.30 mmol, 1.5 equiv.). The crude material was purified by column chromatography (10 – 30% EtOAc in petrol) to give the product as an off-white solid (39.5 mg, 90%).

^{1}H NMR (400 MHz, $CDCl_3$) δ 8.83 (d, *J* = 2.5 Hz, 1H, Het-*H*), 8.09 (dt, *J* = 8.5, 1.0 Hz, 1H, Het-*H*), 7.88 (dd, *J* = 2.5, 1.0 Hz, 1H, Het-*H*), 7.74 (dd, *J* = 8.5, 1.5 Hz, 1H, Het-*H*), 7.66 (ddd, *J* = 8.5, 7.0, 1.5 Hz, 1H, Het-*H*), 7.54 – 7.48 (m, 1H, Het-*H*), 7.36 – 7.29 (m, 2H, 2 × Ar-*H*), 7.25 (td, *J* = 7.0, 2.0 Hz, 3H, 3 × Ar-*H*), 4.17 (s, 2H, Ar-C*H*$_2$-Het); **^{13}C NMR** (101 MHz, $CDCl_3$) δ 152.2, 147.0, 139.8, 134.9, 134.0, 129.3, 129.1, 129.0, 128.9, 128.2, 127.6, 126.8, 126.7, 39.4; **MP** (CH_2Cl_2) 48 °C; **HRMS (ESI)** calculated for $C_{16}H_{14}N$, $[M+H]^+$ is 220.1121, found 220.1121.

The data is consistent with the literature.[10]

3-(2-Methylbenzyl)quinoline (5b)

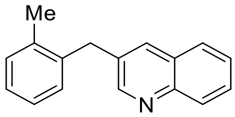

General procedure F was followed using 2-methyl benzyl bromide (40 µL 0.30 mmol, 1.5 equiv.), 3-bromoquinoline (27 µL, 0.20 mmol, 1.0 equiv.) and SMOPS (52.2 mg, 0.30 mmol, 1.5 equiv.). The crude material was purified by column chromatography (10 – 50% EtOAc in petrol) to give the product as a yellow oil (46.1 mg, 99%).

^{1}H NMR (400 MHz, $CDCl_3$) 8.82 (d, *J* = 2.5 Hz, 1H, Het-*H*), 8.08 (d, *J* = 8.5 Hz, 1H, Het-*H*), 7.77 – 7.73 (m, 1H, Het-*H*), 7.70 (dd, *J* = 8.0, 1.5 Hz, 1H, Het-*H*), 7.66 (ddd, *J* = 8.5, 7.0, 1.5 Hz, 1H, Het-*H*), 7.50 (ddd, *J* = 8.0, 7.0, 1.5 Hz, 1H, Het-*H*), 7.23 – 7.13 (m, 4H, 4 × Ar-*H*), 4.17 (s, 2H, Ar-C*H*$_2$-Het), 2.28 (s, 3H, Ar-C*H*$_3$).; **^{13}C NMR** (101 MHz, $CDCl_3$) δ 152.2, 147.0, 137.7, 136.7, 134.7, 133.3, 130.7, 130.1, 129.3, 128.9, 128.3, 127.6, 127.1, 126.8, 126.4, 37.0, 19.8; **IR** (neat)/ν_{max} 3063, 3022, 2923, 1661, 1494; **HRMS (ESI)** calculated for $C_{17}H_{16}N$, $[M+H]^+$ is 234.1277, found 234.1278.

3-(3-Methoxybenzyl)quinoline (5c)

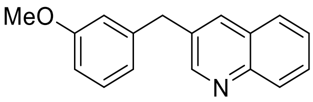

General procedure F was followed, using 3-methoxybenzyl tosylate (87.7 mg, 0.30 mmol, 1.5 equiv.), 3-bromoquinoline (27 µL, 0.20 mmol, 1.0 equiv.), and SMOPS (52.2 mg, 0.30 mmol, 1.5 equiv.). The crude material was purified by column chromatography (10 – 40% EtOAc in petrol) gave the title product as a colourless oil (39.2 mg, 79% yield).

^{1}H NMR (400 MHz, $CDCl_3$) δ 8.73 (d, *J* = 2.0 Hz, 1H, Het-*H*), 8.00 (d, *J* = 8.5 Hz, 1H, Het-*H*), 7.80 (d, *J* = 2.0 Hz, 1H, Het-*H*), 7.64 (dd, *J* = 8.5, 1.5 Hz, 1H, Het-*H*), 7.57 (ddd, *J* = 8.5, 7.5, 1.5 Hz, 1H, Het-*H*), 7.42 (t, *J* = 7.5 Hz, 1H, Het-*H*), 7.15 (t, *J* = 7.5 Hz, 1H, Ar-*H*), 6.75 – 6.67 (m, 3H, 3 × Ar-*H*), 4.04 (s, 2H, Ar-C*H*$_2$-Het), 3.68 (s, 3H, Ar-OC*H*$_3$); **^{13}C NMR** (101 MHz, $CDCl_3$) δ 160.0, 152.1, 147.0, 141.3, 135.0, 133.8, 129.9, 129.2, 129.0, 128.2, 127.6, 126.8, 121.5, 115.0, 111.9, 55.3, 39.4; **HRMS (ESI)** calculated for $C_{17}H_{16}ON$, $[M+H]^+$ is 250.1226 (100%), 251.1260 (18%), found 250.1227 (100%), 251.1261 (18%).

The data is consistent with the literature.[11]

3-(4-Phenoxybenzyl)quinoline (5d)

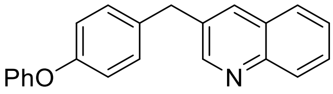

General procedure F was followed, using 4-phenoxybenzyl bromide (78.9 mg, 0.30 mmol, 1.5 equiv.), 3-bromoquinoline (27 µL, 0.20 mmol, 1.0 equiv.), and SMOPS (52.2 mg, 0.30 mmol, 1.5 equiv.). The crude material was purified by column chromatography (20 – 30% EtOAc in petrol) to give the title product as a white solid (58.5 mg, 94% yield).

^{1}H NMR (400 MHz, $CDCl_3$) δ 8.82 (d, *J* = 2.0 Hz, 1H, Het-*H*), 8.11 (dq, *J* = 8.5, 1.0 Hz, 1H, Het-*H*), 7.91 (dd, *J* = 2.0, 1.0 Hz, 1H, Het-*H*), 7.76 (dd, *J* = 8.0, 1.5 Hz, 1H, Het-*H*), 7.68 (ddd, *J* = 8.5, 7.0, 1.5 Hz, 1H, Het-*H*), 7.53 (ddd, *J* = 8.0, 7.0, 1.0 Hz, 1H, Het-*H*), 7.35 – 7.30 (m, 2H, 2 × Ar-*H*), 7.21 – 7.17 (m, 2H, 2 × Ar-*H*), 7.12 – 7.07 (m, 1H, Ar-*H*), 7.02 – 6.99 (m, 2H, 2 × Ar-*H*), 6.99 – 6.95 (m, 2H, 2 × Ar-*H*), 4.15 (s, 2H, Ar-C*H*$_2$-Het); **^{13}C NMR** (101 MHz, $CDCl_3$) δ 157.4, 156.0, 152.0, 146.9, 135.1, 134.6, 134.1, 130.3, 129.9, 129.2, 129.1, 128.3, 127.6, 126.9, 123.3, 119.4, 118.9, 38.7; **MP** (CH_2Cl_2) 63 – 65 °C; **IR (neat)/*v*$_{max}$** 3036, 1589, 1505, 1488, 1239; **HRMS (ESI)** calculated for $C_{22}H_{18}ON$, $[M+H]^+$ is 312.1383, found 312.1383.

Methyl 4-(quinolin-3-ylmethyl)benzoate (5e)

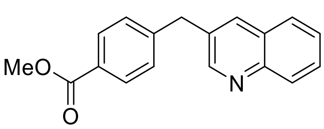

General procedure F was followed, using methyl 4-(bromomethyl)benzoate (68.7 mg, 0.30 mmol, 1.5 equiv.), 3-bromoquinoline (27 µL, 0.20 mmol, 1.0 equiv.), and SMOPS (52.2 mg, 0.30 mmol, 1.5 equiv.). The crude material was purified by column chromatography (15 – 30% EtOAc in petrol) to give the title product as a white crystalline solid (47.5 mg, 86% yield).

^{1}H NMR (400 MHz, $CDCl_3$) δ 8.80 (d, *J* =. 2.5 Hz, 1H, Het-*H*), 8.10 (dq, *J* = 8.5, 1.0 Hz, 1H, Het-*H*), 8.01 – 7.97 (m, 2H, 2 × Ar-*H*), 7.88 (dd, *J* = 2.5, 1.0 Hz, 1H, Het-*H*), 7.74 (dd, *J* = 8.5, 1.5 Hz, 1H, Het-*H*), 7.68 (ddd, *J* = 8.5, 7.0, 1.5 Hz, 1H, Het-*H*), 7.53 (ddd, *J* = 8.5, 7.0, 1.5 Hz, 1H, Het-*H*), 7.32 – 7.28 (m, 2H, 2 × Ar-*H*), 4.22 (s, 2H, Ar-C*H*$_2$-Het), 3.90 (s, 3H, OC*H*$_3$); **^{13}C NMR** (101 MHz, $CDCl_3$) δ 167.0, 151.9, 147.0, 145.0, 135.3, 133.0, 130.2, 129.3, 129.2, 129.1, 128.8, 128.2, 127.6, 127.0, 52.2, 39.3; **MP** (CH_2Cl_2) 84 – 86 °C; **IR** (neat)/*v*$_{max}$ 3000, 2950, 1718, 1610, 1435, 1280, 1109; **HRMS (ESI)** calculated for $C_{18}H_{16}O_2N$, $[M+H]^+$ is 278.1176, found 278.1176.

Ethyl 4-(quinolin-3-ylmethyl)benzoate (5f)

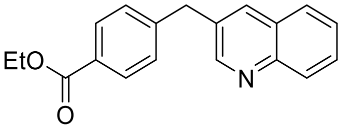

To a microwave vial equipped with a magnetic stirrer bar was added 4-(bromomethyl)benzoic acid (64.5 mg, 0.30 mmol, 1.5 equiv.), SMOPS (52.2 mg, 0.30 mmol, 1.5 equiv.), $Pd(OAc)_2$ (4.5 mg, 10 mol%), $P(^tBu)_2Me{\cdot}HBF_4$ ligand (9.9 mg, 20 mol%) and K_2CO_3 (69.1 mg, 0.50 mmol, 2.5 equiv.). The vessel was sealed with a microwave vial cap, evacuated under vacuum (<1 mbar) and back filled with nitrogen gas 3 times. Addition of 3-bromoquinoline (27 µL, 0.20 mmol, 1.0 equiv.) and DMSO (1.5 mL) followed. The reaction was heated to 120 °C for 18 hours before being cooled to room temperature. A mixture of ethyl iodide (80 µL, 1.00 mmol, 5.0 equiv.) and Cs_2CO_3 (97.7 mg, 0.30 mmol, 1.5 equiv.) in DMSO (1 mL) was added dropwise to the crude cross-coupling reaction. The resulting mixture was heated to 80 °C, and stirred for a further 2 hours before cooling to room temperature. The reaction mixture was transferred to a separating funnel and EtOAc (20 mL) was added. The organic layer was washed with 20% $NaHCO_3$ aqueous solution (3 × 20 mL), then 5% LiCl aqueous solution (3 × 10 mL), before drying over $MgSO_4$. The solvents were removed under vacuum. The crude material was purified by column chromatography (5 – 40% EtOAc in pentane) to give the title product as a pale yellow oil (42.3 mg, 73% yield).

^{1}H NMR (400 MHz, $CDCl_3$) 8.80 (d, *J* = 2.5 Hz, 1H, Het-*H*), 8.10 (dq, *J* = 8.5, 1.0 Hz, 1H, Het-*H*), 8.02 – 7.98 (m, 2H, 2 × Ar-*H*), 7.87 (dd, *J* = 2.5, 1.0 Hz, 1H, Het-*H*), 7.74 (dd, *J* = 8.0, 1.5 Hz, 1H, Het-*H*), 7.68 (ddd, *J* = 8.5, 7.0, 1.5 Hz, 1H, Het-*H*), 7.53 (ddd, *J* = 8.0, 7.0, 1.0 Hz, 1H, Het-*H*), 7.32 – 7.28 (m, 2H, 2 × Ar-*H*), 4.36 (q, *J* = 7.0 Hz, 2H, OC*H*$_2$CH$_3$), 4.22 (s, 2H, Ar-C*H*$_2$-Het), 1.38 (t, *J* = 7.0 Hz, 3H, OCH$_2$C*H*$_3$); **^{13}C NMR** (101 MHz, $CDCl_3$) δ 166.5, 151.8, 146.9, 144.9, 135.3, 133.1, 130.2, 129.3, 129.2, 129.1, 129.1, 128.2, 127.6, 127.0, 61.1, 39.3, 14.5; **IR** (neat)/*v*$_{max}$ 3064, 1714, 1610, 1496, 1416, 1367, 1276, 1178, 1106; **HRMS (ESI)** calculated for $C_{19}H_{18}O_2N$, $[M+H]^+$ is 292.1332 (100%), 293.1366 (22%), found 292.1331 (100%), 293.1365 (22%).

3-(3-Bromobenzyl)quinoline (5g)

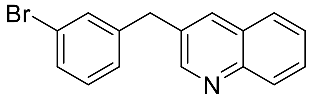

General procedure F was followed, using 3-bromobenzyl bromide (74.9 mg, 0.30 mmol, 1.5 equiv.), 3-bromoquinoline (27 µL, 0.20 mmol, 1.0 equiv.), and SMOPS (52.2 mg, 0.30 mmol, 1.5 equiv.). The crude material was purified by column chromatography (10 – 40% EtOAc in petrol) to give the title product as a colourless oil (41.8 mg, 70% yield).

^{1}H NMR (400 MHz, $CDCl_3$) δ 8.79 (d, *J* = 2.5 Hz, 1H, Het-*H*), 8.10 (dd, *J* = 8.5, 1.0 Hz, 1H, 1H, Het-*H*), 7.88 (dd, *J* = 2.5, 1.0 Hz, 1H, 1H, Het-*H*), 7.77 – 7.74 (m, 1H, 1H, Het-*H*), 7.71 – 7.65 (m, 1H, 1H, Het-*H*), 7.56 – 7.51 (m, 1H, 1H, Het-*H*), 7.40 – 7.35 (m, 2H, Ar-*H*), 7.21 – 7.13 (m, 2H, Ar-*H*), 4.13 (s, 2H, Ar-C*H*$_2$-Het); **^{13}C NMR** (101 MHz, $CDCl_3$) δ 151.9, 147.0, 142.1, 135.2, 133.1, 132.1, 130.4, 129.9, 129.28, 129.26, 128.2, 127.7, 127.6, 127.0, 123.0, 39.0; **HRMS (ESI)** calculated for $C_{16}H_{13}NBr^{79}$ [M+H]$^+$ is 298.0226 (100%), 299.0260 (17%) and for $C_{16}H_{13}NBr^{81}$ [M+H]$^+$ is 300.0205 (97%), 301.0239 (17%), found 298.0227 (100%), 299.0261 (15%), 300.0205 (95%), 301.0239 (15%).

The data is consistent with the literature.[11]

3-(2-Bromobenzyl)quinoline (5h)

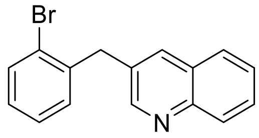

General procedure F was followed, using 2-bromobenzyl bromide (74.9 mg, 0.30 mmol, 1.5 equiv.), 3-bromoquinoline (27 µL, 0.20 mmol, 1.0 equiv.), and SMOPS (52.2 mg, 0.30 mmol, 1.5 equiv.). The crude material was purified by column chromatography (2 – 20% EtOAc in petrol) to give the title product as a yellow oil (18.3 mg, 31% yield).

^{1}H NMR (400 MHz, $CDCl_3$) δ 8.85 (d, *J* = 2.0 Hz, 1H, Het-*H*), 8.08 (dq, *J* = 8.5, 1.0 Hz, 1H, Het-*H*), 7.85 (dd, *J* = 2.0, 1.0 Hz, 1H, Het-*H*), 7.73 (dd, *J* = 8.5, 1.5 Hz, 1H, Het-*H*), 7.67 (ddd, *J* = 8.5, 7.0, 1.5 Hz, 1H, Het-*H*), 7.61 (dd, *J* = 8.0, 1.5 Hz, 1H, Ar-*H*), 7.51 (ddd, *J* = 8.0, 7.0, 1.5 Hz, 1H, Het-*H*), 7.30 – 7.26 (m, 1H, Ar-*H*), 7.22 (dd, *J* = 7.5, 2.0 Hz, 1H, Ar-*H*), 7.14 (td, *J* = 7.5, 2.0 Hz, 1H, Ar-*H*), 4.30 (s, 2H, Ar-C*H*$_2$-Het); **^{13}C NMR** (101 MHz, $CDCl_3$) δ 152.2, 147.1, 139.2, 135.1, 133.3, 132.5, 131.3, 129.4, 129.1, 128.6, 128.2, 127.9, 127.6, 126.8, 125.0, 39.4; **IR (neat)/ν_{max}** 3057, 1605, 1496, 1471, 1439, 1025; **HRMS (ESI)** calculated for $C_{26}H_{28}ON$, [M+H]$^+$ is 298.0226 (100%), 299.0260 (17%), 300.0205 (95%), 301.0239 (17%), found 298.0224 (100%), 299.0261 (18%), 300.0205 (100%), 301.0236 (18%).

3-(3-Chlorobenzyl)quinoline (5i)

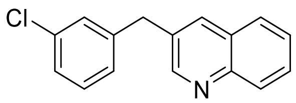

General procedure F was followed, using 3-chlorobenzyl bromide (39 µL, 0.30 mmol, 1.5 equiv.), 3-bromoquinoline (27 µL, 0.20 mmol, 1.0 equiv.), and SMOPS (52.2 mg, 0.30 mmol, 1.5 equiv.). The crude material was purified by column chromatography (40 – 70% EtOAc in petrol) to give the title product as a colourless oil (50.2 mg, 99% yield).

^{1}H NMR (400 MHz, $CDCl_3$) δ 8.79 (d, *J* = 2.5 Hz, 1H, Het-*H*), 8.10 (dq, *J* = 8.5, 1.0 Hz, 1H, Het-*H*), 7.88 (dd, *J* = 2.5, 1.0 Hz, 1H, Het-*H*), 7.75 (dd, *J* = 8.0, 1.5 Hz, 1H, Het-*H*), 7.67 (ddd, *J* = 8.5, 7.0, 1.5 Hz, 1H, Het-*H*), 7.52 (ddd, *J* = 8.0, 7.0, 1.5 Hz, 1H, Het-*H*), 7.26 – 7.23 (m, 1H, Ar-*H*), 7.22 – 7.20 (m, 2H, 2 × Ar-*H*), 7.10 (dt, *J* = 7.0, 2.0 Hz, 1H, Ar-*H*), 4.13 (s, 2H, Ar-C*H*$_2$-Het); **^{13}C NMR** (101 MHz, $CDCl_3$) δ 151.9, 147.1, 141.8, 135.2, 134.7, 133.1, 130.1, 129.3, 129.24, 129.18, 129.1, 127.6, 127.6, 126.99, 126.97 39.0; **IR** (neat)/ν_{max} 3063, 2981, 1595, 1572, 1495, 1475, 1430, 1378; **HRMS (ESI)** calculated for $C_{16}H_{13}NCl^{35}$ [M+H]$^+$ is 254.0731 (100%), 255.0765 (17%) and for $C_{16}H_{13}NCl^{37}$ [M+H]$^+$ is 256.0702 (32%), 257.0735 (6%), found 254.0732 (100%), 255.0765 (17%), 256.0703 (35%), 257.0735 (7%).

3-(3,5-Difluorobenzyl)quinoline (5j)

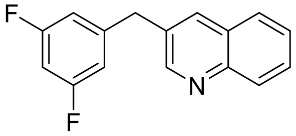

General procedure F was followed, using 3,5-difluorobenzyl bromide (39 µL, 0.30 mmol, 1.5 equiv.), 3-bromoquinoline (27 µL, 0.20 mmol, 1.0 equiv.), and SMOPS (52.2 mg, 0.30 mmol, 1.5 equiv.). The crude material was purified by column chromatography (5 – 30% EtOAc in petrol) to give the title product as a white solid (49.7 mg, 97% yield).

^{1}H NMR (400 MHz, $CDCl_3$) δ 8.78 (d, *J* = 2.0 Hz, 1H, Het-*H*), 8.11 (dt, *J* = 8.5, 1.0 Hz, 1H, Het-*H*), 7.91 (dd, *J* = 2.0, 1.0 Hz, 1H, Het-*H*), 7.77 (dd, *J* = 8.0, 1.5 Hz, 1H, Het-*H*), 7.70 (ddd, *J* = 8.5, 7.0, 1.5 Hz, 1H, Het-*H*), 7.55 (ddd, *J* = 8.0, 7.0, 1.5 Hz, 1H, Het-*H*), 6.74 (dt, *J* = 7.0, 2.0 Hz, 2H, 2 × Ar-*H*), 6.69 (tt, *J* = 8.5, 2.0 Hz, 1H, Ar-*H*), 4.14 (s, 2H, Ar-CH_2-Het); **^{13}C NMR** (101 MHz, $CDCl_3$) δ 163.4 (dd, *J* = 249.0, 13.0 Hz), 151.6, 147.0, 143.6 (t, *J* = 9.0 Hz), 135.4, 132.4, 129.5, 129.3, 128.2, 127.6, 127.2, 111.9 (dd, *J* = 18.0, 6.5 Hz), 102.4 (t, *J* = 25.5 Hz), 39.0 (t, *J* = 2.0 Hz); **^{19}F NMR** (377 MHz, $CDCl_3$) δ -109.53; **MP** (CH_2Cl_2) 68 – 70 °C; **IR** (neat)/v_{max} 3061, 2922, 1625, 1595, 1460, 1322, 1117; **HRMS (ESI)** calculated for $C_{16}H_{12}F_2N$, $[M+H]^+$ is 256.0932 (100%), 257.0966 (17%), 258.0999 (1%), found 256.0933 (100%), 257.0966 (20%), 258.1000 (1%).

3-(2,6-Difluorobenzyl)quinoline (5k)

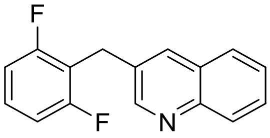

General procedure F was followed, using 2,6-difluorobenzyl bromide (62.1 mg, 0.30 mmol, 1.5 equiv.), 3-bromoquinoline (27 µL, 0.20 mmol, 1.0 equiv.), and SMOPS (52.2 mg, 0.30 mmol, 1.5 equiv.). The crude material was purified by column chromatography (5 – 20% EtOAc in petrol) to give the title product as a white crystalline solid (50.4 mg, 99% yield).

^{1}H NMR (400 MHz, $CDCl_3$) δ 8.89 (d, *J* = 2.5 Hz, 1H, Het-*H*), 8.08 (dd, *J* = 8.5, 1.0 Hz, 1H, Het-*H*), 7.95 (dd, *J* = 2.5, 1.0 Hz, 1H, Het-*H*), 7.74 (dd, *J* = 8.5, 1.5 Hz, 1H, Het-*H*), 7.66 (ddd, *J* = 8.5, 7.0, 1.5 Hz, 1H, Het-*H*), 7.51 (ddd, *J* = 8.0, 7.0, 1.2 Hz, 1H, Het-*H*), 7.20 (tt, *J* = 8.5, 6.5 Hz, 1H, Ar-*H*), 6.91 (t, *J* = 8.0 Hz, 2H, 2 × Ar-*H*), 4.20 (s, 2H, Ar-CH_2-Het); **^{13}C NMR** (101 MHz, $CDCl_3$) δ 161.4 (dd, J = 247.5, 8.5 Hz), 151.6, 146.9, 134.8, 132.1, 129.2, 128.7 (t, *J* = 10.0 Hz), 128.2, 127.61, 127.56, 126.9, 115.8 (t, *J* = 20.0 Hz), 111.6 (dd, *J* = 18.5, 6.5 Hz), 25.9 (t, *J* = 3.0 Hz); **^{19}F NMR** (377 MHz, $CDCl_3$) δ -114.85; **MP** (CH_2Cl_2) 88 – 90 °C; **IR** (neat)/v_{max} 3036, 2941, 1623, 1591, 1498, 1468, 1261; **HRMS (ESI)** calculated for $C_{16}H_{12}NF_2$, $[M+H]^+$ is 256.0932, found 256.0933.

3-(Thiophen-3-ylmethyl)quinoline (5l)

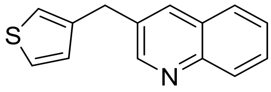

General procedure F was followed, using 3-(bromomethyl)thiophene (33 µL, 0.30 mmol, 1.5 equiv.), 3-bromoquinoline (27 µL, 0.20 mmol, 1.0 equiv.), and SMOPS (52.2 mg, 0.30 mmol, 1.5 equiv.). The crude residue was purified by column chromatography (10 – 30% EtOAc in toluene) to give the product as a yellow oil (20.7 mg, 46%, 90% NMR purity).

^{1}H NMR (400 MHz, DMSO-d_6) δ 8.84 (d, *J* = 2.5 Hz, 1H, Het-*H*), 8.15 (dd, *J* = 2.5, 1.0 Hz, 1H, Het-*H*), 7.99 (dd, *J* = 8.5, 1.0 Hz, 1H, Het-*H*), 7.91 (dd, *J* = 8.5, 1.5 Hz, 1H, Het-*H*), 7.73 – 7.68 (m, 1H, Het-*H*), 7.60 – 7.56 (m, 1H, Het-*H*), 7.49 (dd, *J* = 5.0, 3.0 Hz, 1H, Het-*H*), 7.29 – 7.27 (m, 1H, Het-*H*), 7.04 (dd, *J* = 5.0, 1.5 Hz, 1H), 4.17 (s, 2H, Het-CH_2-Het); **^{13}C NMR** (101 MHz, DMSO-d_6) δ 151.9, 146.3, 140.5, 134.2, 133.9, 128.8, 128.6, 128.4, 127.69, 127.66, 126.7, 126.6, 121.9, 32.9; **IR** (neat)/v_{max} 3053, 2918, 2850, 1496, 1265; **HRMS (ESI)** calculated for $C_{14}H_{12}SN$, $[M+H]^+$ is 226.0685 (100%), 227.0718 (15%), found 226.0686 (100%), 227.0720 (15%).

The data is consistent with the literature.[12]

3,5-Dimethyl-4-(quinolin-3-ylmethyl)isoxazole (5m)

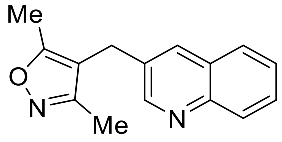

General procedure F was followed, using 4-chloromethyl-3,5-dimethylisoxazole (37 µL, 0.30 mmol, 1.5 equiv.), 3-bromoquinoline (27 µL, 0.20 mmol, 1.0 equiv.), and β-nitrile sulfinate (42.3 mg, 0.30 mmol, 1.5 equiv.). The crude residue was purified by column chromatography (10 – 50 % EtOAc in petrol) to give the product as a yellow solid (39.9 mg, 84%).

^{1}H NMR (400 MHz, $CDCl_3$) δ 8.79 (s, 1H, Het-*H*), 8.10 (dd, *J* = 8.5, 1.5 Hz, 1H, Het-*H*), 7.74 (dd, *J* = 8.0, 1.5 Hz, 2H, 2 × Het-*H*), 7.69 (ddd, *J* = 8.5, 7.0, 1.5 Hz, 1H, Het-*H*), 7.54 (ddd, *J* = 8.0, 7.0, 1.5 Hz, 1H, Het-*H*), 3.87 (s, 2H, Het-C*H₂*-Het), 2.36 (s, 3H, Het-C*H₃*), 2.12 (s, 3H, Het-C*H₃*); **^{13}C NMR** (101 MHz, $CDCl_3$) δ 166.1, 159.9, 151.2, 146.7, 134.2, 131.5, 129.5, 129.2, 128.1, 127.6, 127.3, 111.2, 25.8, 11.3, 10.5; **MP** (CH_2Cl_2) 98 – 100 °C; **IR (neat)/*v_{max}*** 3027, 2970, 1640, 1571, 1452, 1423, 1381, 1260, 983; **HRMS (ESI)** calculated for $C_{15}H_{15}N_2O$, $[M+H]^+$ is 239.1179 (100%), 240.1213 (17%), found 239.1179 (100%), 240.1212 (17%).

4-Butoxy-6-(quinolin-3-ylmethyl)-2-(trifluoromethyl)quinoline (5n)

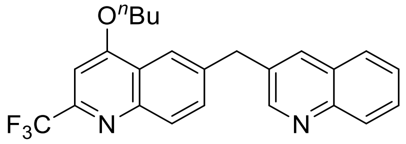

General procedure F was followed, using 6-(bromomethyl)-4-butoxy-2-(trifluoromethyl)quinoline (95 mg, 0.26 mmol, 1.3 equiv.), 3-bromoquinoline (27 µL, 0.20 mmol, 1.0 equiv.), and SMOPS (52.2 mg, 0.30 mmol, 1.5 equiv.). The crude residue was purified by column chromatography (20 – 30 % EtOAc in petrol) to give the product as an off-white solid (73.1 mg, 89%).

^{1}H NMR (400 MHz, $CDCl_3$) δ 8.87 (d, *J* = 2.0 Hz, 1H, Het-*H*), 8.14 (d, *J* = 8.5 Hz, 1H, Het-*H*), 8.11 (d, *J* = 2.0 Hz, 1H, Het-*H*), 8.09 (d, *J* = 8.5 Hz, 1H, Het-*H*), 7.94 (*app* s, 1H, Het-*H*), 7.75 (dd, *J* = 8.0, 1.5 Hz, 1H, Het-*H*), 7.70 (ddd, *J* = 8.5, 7.0, 1.5 Hz, 1H, Het-*H*), 7.63 (dd, *J* = 8.5, 2.0 Hz, 1H, Het-*H*), 7.55 (ddd, *J* = 8.0, 7.0, 1.5 Hz, 1H, Het-*H*), 7.04 (s, 1H, Het-*H*), 4.40 (s, 2H, Het-C*H₂*-Het), 4.26 (t, *J* = 6.5 Hz, 2H, -O-C*H₂*-CH₂-), 1.97 – 1.90 (m, 2H, -O-CH₂-C*H₂*-), 1.56 (h, *J* = 7.5 Hz, 2H, -CH₂-C*H₂*-CH₃), 1.00 (t, *J* = 7.5 Hz, 3H, C*H₃*); **^{13}C NMR** (101 MHz, $CDCl_3$) δ 163.1, 151.9, 148.9 (q, *J* = 34.0 Hz), 147.3, 147.0, 139.2, 135.3, 133.2, 132.4, 130.3, 129.3, 129.2, 128.2, 127.6, 127.0, 122.0, 121.8 (q, *J* = 275.5 Hz), 121.5, 97.1 (q, *J* = 2.0 Hz), 69.1, 39.5, 30.9, 19.4, 13.9; **^{19}F NMR** (377 MHz, $CDCl_3$) δ -67.71; **MP** (CH_2Cl_2) 81 – 84 °C; **IR** (neat)/*v_{max}* 3062, 1591, 1575, 1413, 1280, 1255, 1135; **HRMS (ESI)** calculated for $C_{24}H_{22}ON_2F_3$, $[M+H]^+$ is 411.1679, found 411.1679.

6-(Quinolin-3-ylmethyl)benzo[d]thiazole (5o)

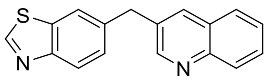

General procedure F was followed, using 6-(bromomethyl)benzo[d]thiazole (68.4 mg, 0.30 mmol, 1.5 equiv.), 3-bromoquinoline (27 µL, 0.20 mmol, 1.0 equiv.), and SMOPS (52.2 mg, 0.30 mmol, 1.5 equiv.). The crude material was purified by column chromatography (10 – 40% EtOAc in petrol) to give the title product as a white solid (25.2 mg, 46% yield).

^{1}H NMR (400 MHz, $CDCl_3$) δ 8.95 (s, 1H, Het-*H*), 8.85 (d, *J* = 2.5 Hz, 1H, Het-*H*), 8.13 – 8.07 (m, 2H, 2 × Het-*H*), 7.92 (dd, *J* = 2.5, 1.0 Hz, 1H, Het-*H*), 7.78 (dd, *J* = 1.5, 1.0 Hz, 1H, Het-*H*), 7.75 (dd, *J* = 8.5, 1.5 Hz, 1H, Het-*H*), 7.68 (ddd, *J* = 8.5, 7.0, 1.5 Hz, 1H, Het-*H*), 7.53 (ddd, *J* = 8.5, 7.0, 1.5 Hz, 1H, Het-*H*), 7.41 (dd, *J* = 8.5, 1.5 Hz, 1H, Het-*H*), 4.33 (s, 2H, Het-C*H₂*-Het); **^{13}C NMR** (101 MHz, $CDCl_3$) δ 153.9, 152.3, 151.9, 146.9, 137.6, 135.3, 134.5, 133.5, 129.3, 129.2, 128.2, 127.7, 127.6, 127.1, 123.9, 121.9, 39.3; **MP** (CH_2Cl_2) 68 – 70 °C; **IR** (neat)/*v_{max}* 3056, 1472, 1441, 1293, 1269, 1125, 900; **HRMS (ESI)** calculated for $C_{17}H_{13}N_2S$, $[M+H]^+$ is 277.0794, found 277.0796.

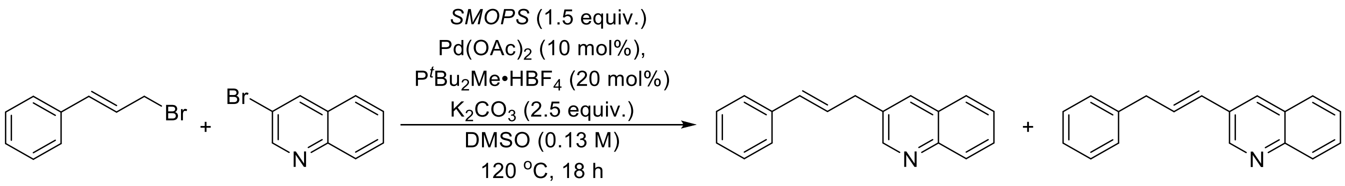

General procedure F was followed, using 3-bromo-1-phenyl-1-propene (44 µL, 0.30 mmol, 1.5 equiv.), 3-bromoquinoline (27 µL, 0.20 mmol, 1.0 equiv.) and SMOPS (52.2 mg, 0.30 mmol, 1.5 equiv.). The crude residue was purified by column chromatography (0 – 20% EtOAc in petrol) to give a mixture of two positional isomers as a yellow oil (42.4 mg, 86%). For analysis, the isomers were separated by preparative thin-layer chromatography (15% EtOAc in petrol) to give (*E*)-3-(3-phenylprop-1-en-1-yl)quinoline (17.2 mg, 35% yield) and 3-cinnamylquinoline (14.2 mg, 29% yield), both as yellow oils.

(*E*)-3-(3-Phenylprop-1-en-1-yl)quinoline (5q')

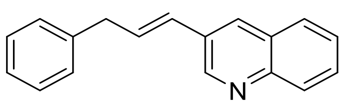

^{1}H NMR (400 MHz, $CDCl_3$) δ 8.98 (d, *J* = 2.5 Hz, 1H, Het-*H*), 8.08 (d, *J* = 8.5 Hz, 1H, Het-*H*), 8.02 (d, *J* = 2.5 Hz, 1H, Het-*H*), 7.77 (dd, *J* = 8.0, 1.5 Hz, 1H, Het-*H*), 7.66 (ddd, *J* = 8.5, 7.0, 1.5 Hz, 1H, Het-*H*), 7.52 (ddd, *J* = 8.0, 7.0, 1.5 Hz, 1H, Het-*H*), 7.39 – 7.32 (m, 2H, 2 × Ar-*H*), 7.31 – 7.23 (m, 3H, 3 × Ar-*H*), 6.67 – 6.54 (m, 2H, 2 × C*H*), 3.64 (d, *J* = 5.0 Hz, 2H, Ar-C*H*₂-R); **^{13}C NMR** (101 MHz, $CDCl_3$) δ 149.3, 147.2, 139.6, 132.3, 132.2, 130.5, 129.2, 128.9, 128.80, 128.78, 128.2, 127.90, 127.85, 127.1, 126.6, 39.7; **IR** (neat)/ν_{max} 3061, 2850, 1602, 1570, 1494, 1453, 966; **HRMS (ESI)** calculated for $C_{18}H_{16}N$, $[M+H]^+$ is 246.1277 (100%), 247.1311 (20%), found 246.1278 (100%), 247.1312 (22%).

3-Cinnamylquinoline (5q'')

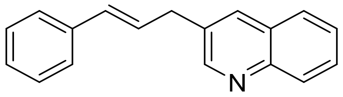

^{1}H NMR (400 MHz, $CDCl_3$) δ 8.77 (s, 1H), 8.04 (d, *J* = 8.0 Hz, 1H, Het-*H*), 7.92 (s, 1H, Het-*H*), 7.71 (d, *J* = 8.0 Hz, 1H, Het-*H*), 7.61 (ddd, *J* = 8.5, 7.0, 1.5 Hz, 1H, Het-*H*), 7.46 (ddd, *J* = 8.5, 7.0, 1.5 Hz, 1H, Het-*H*), 7.32 – 7.28 (m, 2H, 2 × Ar-*H*), 7.27 – 7.21 (m, 2H, 2 × Ar-*H*), 7.19 – 7.13 (m, 1H, Ar-*H*), 6.45 (dt, *J* = 16.0, 1.5 Hz, 1H, C*H*), 6.33 (dt, *J* = 16.0, 6.5 Hz, 1H, C*H*), 3.67 (d, *J* = 6.5 Hz, 2H, Ar-C*H*₂-R); **^{13}C NMR** (101 MHz, $CDCl_3$) δ 152.0, 146.9, 137.2, 135.0, 133.0, 132.4, 129.2, 129.1, 128.7, 128.3, 127.7, 127.60, 127.56, 126.9, 126.4, 36.7; **IR (neat)/ν_{max}** 3059, 1599, 1571, 1496, 1449, 966; **HRMS (ESI)** calculated for $C_{18}H_{16}N$, $[M+H]^+$ is 246.1277 (100%), 247.1311 (20%), found 246.1278 (100%), 247.1312 (22%).

The data is consistent with the literature.[13]

3-(4-(Methylsulfonyl)benzyl)quinoline (5r)

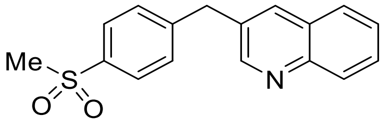

General procedure F was followed, using 4-(methylsulfonyl)benzyl bromide (79.5 mg, 0.30 mmol, 1.5 equiv.), 3-bromoquinoline (27 µL, 0.20 mmol, 1.0 equiv.), and SMOPS (52.2 mg, 0.30 mmol, 1.5 equiv.). The crude material was purified by column chromatography (20 – 45% EtOAc in petrol) to give the title product as a white solid (54.3 mg, 91% yield).

^{1}H NMR (400 MHz, $CDCl_3$) δ 8.78 (d, *J* = 2.5 Hz, 1H, Het-*H*), 8.09 (dq, *J* = 8.5, 1.0 Hz, 1H, Het-*H*), 7.91 – 7.86 (m, 3H, Het-*H*, 2 × Ar-*H*), 7.76 (dd, *J* = 8.0, 1.5 Hz, 1H, Het-*H*), 7.69 (ddd, *J* = 8.5, 7.0, 1.5 Hz, 1H, Het-*H*), 7.54 (ddd, *J* = 8.0, 7.0, 1.2 Hz, 1H, Het-*H*), 7.45 – 7.40 (m, 2H, 2 × Ar-*H*), 4.25 (s, 2H, Ar-C*H*$_2$-Het), 3.03 (s, 3H, Ar-SO_2-C*H*$_3$); **^{13}C NMR** (101 MHz, $CDCl_3$) δ 151.7, 147.1, 146.3, 139.1, 135.4, 132.3, 130.0, 129.5, 129.3, 128.1, 128.0, 127.6, 127.2, 44.7, 39.2; **HRMS (ESI)** calculated for $C_{17}H_{16}O_2NS$, $[M+H]^+$ is 298.0896, found 298.0896.

The data is consistent with the literature.[14]

3-(3-(Pentafluorothio)benzyl)quinoline (5s)

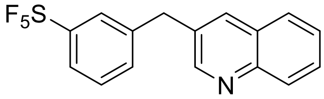

General procedure F was followed, using 3-(pentafluorothio)benzyl bromide (89.1 mg, 0.30 mmol, 1.5 equiv.), 3-bromoquinoline (27 µL, 0.20 mmol, 1.0 equiv.), and SMOPS (52.2 mg, 0.30 mmol, 1.5 equiv.). The crude material was purified by column chromatography (10 – 30% EtOAc in petrol) to give the title product as a colourless oil (59.7 mg, 86% yield).

^{1}H NMR (400 MHz, $CDCl_3$) δ 8.80 (d, *J* = 2.5 Hz, 1H, Het-*H*), 8.11 (dq, *J* = 8.5, 1.0 Hz, 1H, Het-*H*), 7.89 (dd, *J* = 2.5, 1.0 Hz, 1H, Het-*H*), 7.76 (dd, *J* = 8.5, 1.5 Hz, 1H, Het-*H*), 7.70 (ddd, *J* = 8.5, 7.0, 1.5 Hz, 1H, Het-*H*), 7.67 – 7.62 (m, 2H, 2 × Ar-*H*), 7.54 (ddd, *J* = 8.5, 7.0, 1.5 Hz, 1H, Het-*H*), 7.41 (app. t, *J* = 7.5 Hz, 1H, Ar-*H*), 7.35 (d, *J* = 7.5 Hz, 1H, Ar-*H*), 4.23 (s, 2H, Ar-C*H*$_2$-Het); **^{13}C NMR** (101 MHz, $CDCl_3$) δ 154.4 (quint, *J* = 16.5 Hz), 151.7, 147.2, 141.0, 135.3, 132.5, 132.2, 129.4, 129.3, 129.3, 128.2, 127.6, 127.1, 126.5 (quint, *J* = 4.5 Hz), 124.2 (quint, *J* = 4.5 Hz), 39.2; **^{19}F NMR** (377 MHz, $CDCl_3$) δ 85.37 – 83.38 (m, 1F), 62.82 (d, *J* = 150.5 Hz, 4F); **IR** (neat)/ν_{max} 2981, 2361, 1736, 1496; **HRMS (ESI)** calculated for $C_{16}H_{13}SF_5N$, $[M+H]^+$ is 346.0683, found 346.0682.

3-(3-Methoxy-2-(trifluoromethyl)benzyl)quinoline (5t)

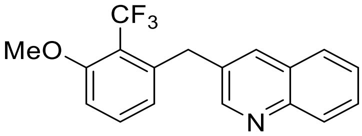

General procedure F was followed, using 3-methoxy-2-(trifluoromethyl)benzyl bromide (80.7 mg, 0.30 mmol, 1.5 equiv.), 3-bromoquinoline (27 µL, 0.20 mmol, 1.0 equiv.), and SMOPS (52.2 mg, 0.30 mmol, 1.5 equiv.). The crude material was purified by column chromatography (15 – 30% EtOAc in petrol) to give the title product as a white solid (61.5 mg, 97% yield).

^{1}H NMR (400 MHz, $CDCl_3$) δ 8.77 (d, *J* = 2.5 Hz, 1H, Het-*H*), 8.09 (dq, *J* = 8.5, 1.0 Hz, 1H, Het-*H*), 7.75 – 7.68 (m, 2H, 2 × Het-*H*), 7.66 (ddd, *J* = 8.5, 7.0, 1.0 Hz, 1H, Het-*H*), 7.50 (ddd, *J* = 8.5, 7.0, 1.0 Hz, 1H, Het-*H*), 7.43 (t, *J* = 8.0 Hz, 1H, Het-*H*), 6.97 (d, *J* = 8.0 Hz, 1H, Ar-*H*), 6.84 (d, *J* = 8.0 Hz, 1H, Ar-*H*), 4.37 (s, 2H, Ar-C*H*$_2$-Het), 3.90 (s, 3H, Ar-OC*H*$_3$); **^{13}C NMR** (101 MHz, $CDCl_3$) δ 159.3 (q, *J* = 2.5 Hz), 151.7, 146.8, 139.3 (q, *J* = 1.5 Hz), 134.7, 133.4, 132.8, 129.2, 129.1, 128.2, 127.6, 126.9, 124.9 (q, *J* = 276.5 Hz), 124.8, 117.9 (q, *J* = 29.5 Hz), 111.4, 56.5, 37.6 (q, *J* = 4.5 Hz); **^{19}F NMR** (377 MHz, $CDCl_3$) δ -54.54; **MP** (CH_2Cl_2) 74 – 78 °C; **IR** (neat)/ν_{max} 3032, 2843, 1476, 1271, 1120; **HRMS (ESI)** calculated for $C_{18}H_{15}ONF_3$, $[M+H]^+$ is 318.1100, found 318.1101.

3-(4-Methoxybenzyl)pyridine (5u)

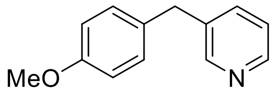

General procedure E was followed using sodium 4-methoxybenzyl sulfinate (83.3 mg, 0.40 mmol, 2.0 equiv.) and 3-bromopyridine (20 µL 0.20 mmol, 1.0 equiv.). The crude material was purified by column chromatography (10 – 50% EtOAc in petrol) to give the product as a yellow oil (37.1 mg, 93%).

General procedure F was followed, using 4-methoxybenzyl chloride (41 µL, 0.30 mmol, 1.5 equiv.), 3-bromopyridine (20 µL, 0.20 mmol, 1.0 equiv.) and SMOPS (52.2 mg, 0.30 mmol, 1.5 equiv.). Purification by column chromatography (10 – 50% EtOAc in petrol) gave the title product as a yellow oil (35.4 mg, 89% yield).

^{1}H NMR (400 MHz, $CDCl_3$) δ 8.53 – 8.41 (m, 2H, 2 × Het-*H*), 7.48 – 7.43 (m, 1H, Het-*H*), 7.20 (dd, *J* = 7.8, 4.8 Hz, 1H, Het-*H*), 7.12 – 7.05 (m, 2H, 2 × Het-*H*), 6.86 – 6.82 (m, 2H, 2 × Het-*H*), 3.92 (s, 2H, Ar-CH_2-Het), 3.78 (s, 3H, Ar-OCH_3); **^{13}C NMR** (101 MHz, $CDCl_3$) δ 158.4, 150.0, 147.5, 137.1, 136.5, 131.9, 129.9, 123.6, 114.2, 55.4, 38.3; **HRMS (ESI)** calculated for $C_{13}H_{14}ON$, $[M+H]^+$ is 200.1070 (100%), 201.1104 (15%), found 200.1073 (100%), 201.1108 (15%)

The data is consistent with the literature.[15]

3-(4-(Trifluoromethyl)benzyl)pyridine (5v)

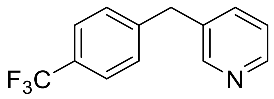

General procedure E was followed using sodium 4-(trifluoromethyl)benzyl sulfinate (98.5 mg, 0.40 mmol, 2.0 equiv.) and 3-bromopyridine (20 µL 0.20 mmol, 1.0 equiv.). The crude material was purified by column chromatography (10 – 40% EtOAc in petrol) to give the product as a yellow oil (23.7 mg, 50%).

General procedure F was followed, using 4-(trifluoromethyl)benzyl bromide (71.7 mg, 0.30 mmol, 2.0 equiv.), 3-bromopyridine (14 µL, 0.15 mmol, 1.0 equiv.) and β-nitrile sulfinate (46.2 mg, 0.30 mmol, 2.2 equiv.). The crude material was purified by column chromatography (10 – 40% EtOAc in petrol) to give the product as a yellow oil (32.1 mg, 90%).

^{1}H NMR (400 MHz, $CDCl_3$) δ 8.53 – 8.45 (m, 2H), 7.56 (d, *J* = 8.0 Hz, 2H), 7.45 (dt, *J* = 8.0, 2.0 Hz, 1H), 7.29 (d, *J* = 8.0 Hz, 2H), 7.22 (ddd, *J* = 8.0, 5.0, 1.0 Hz, 1H), 4.03 (s, 2H, Ar-CH_2-Het); **^{13}C NMR** (101 MHz, $CDCl_3$) δ 150.3, 148.2, 144.0, 136.4, 135.6, 129.3, 129.1 (q, *J* = 32.4 Hz), 125.8 (q, *J* = 4.0 Hz), 124.1 (q, *J* = 271.8 Hz), 123.7, 38.9; **^{19}F NMR** (377 MHz, $CDCl_3$) δ -62.46; **IR** (neat)/v_{max} 3030, 2922, 1619, 1576, 1325, 1019; **HRMS (ESI)** calculated for $C_{13}H_{10}F_3N$, $[M+H]^+$ is 238.0838 (100%), 239.0872 (15%), found 238.0838 (100%), 239.0871 (15%).

The data is consistent with the literature.[3b]

5-Benzyl-2-methoxypyridine (5w)

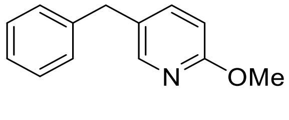

General procedure F was followed using benzyl bromide (36 µL, 0.30 mmol, 1.5 equiv.), 2-methoxy-5-bromopyridine (26 µL, 0.20 mmol, 1.0 equiv.) and SMOPS (52.2 mg, 0.30 mmol, 1.5 equiv.). The crude material was purified by column chromatography (10 – 40% Et_2O in petrol) to give the product as a colourless oil (39.4 mg, 99% yield).

5 mmol scale up
To a 250 mL two-necked round bottom flask equipped with a magnetic stirrer bar was added SMOPS (1.31 g, 7.5 mmol, 1.5 equiv.), $Pd(OAc)_2$ (112 mg, 10 mol%), $P(^tBu)_2Me{\cdot}HBF_4$ ligand (248 mg, 20 mol%) and K_2CO_3 (1.73 g, 12.5 mmol, 2.5 equiv.). One neck of the round bottom flask was fitted with a condenser and the other was fitted with a suba seal. The reaction vessel was evacuated under vacuum (<1 mbar) and back filled with nitrogen gas. This cycle was repeated two further times. Addition of 5-Bromo-2-methoxypyridine (0.65 mL, 5.0 mmol, 1.0 equiv.), benzyl bromide (0.89 mL, 7.5 mmol, 1.5 equiv.) and anhydrous DMSO (38 mL) followed. The reaction mixture was heated to 120 °C for 18 h before cooling to room temperature. The reaction mixture was filtered through celite, and washed

with EtOAc. The EtOAc washings were then transferred to a 500 mL separating funnel, and the organics were washed with 5% aqueous LiCl solution until the DMSO was fully extracted from the organic layer. The organic layer was dried over $MgSO_4$, and the solvents removed under vacuum. The crude residue was purified by column chromatography (2 – 10% EtOAc in petrol) to give the title product as a clear oil (986.3 mg, 99% yield).

^{1}H NMR (400 MHz, $CDCl_3$) δ 8.03 (dd, *J* = 2.5, 1.0 Hz, 1H, Het-*H*), 7.37 (dd, *J* = 8.5, 2.5 Hz, 1H, Het-*H*), 7.32 – 7.26 (m, 2H, 2 × Ar-*H*), 7.23 – 7.18 (m, 1H, Ar-*H*), 7.18 – 7.15 (m, 2H, 2 × Ar-*H*), 6.67 (dd, *J* = 8.5, 1.0 Hz, 1H, Het-*H*), 3.92 (s, 3H, Het-*OCH$_3$*), 3.90 (s, 2H, Ar-*CH$_2$*-Het); **^{13}C NMR** (101 MHz, $CDCl_3$) δ 163.0, 146.4, 140.7, 139.6, 129.3, 128.9, 128.7, 126.4, 110.9, 53.5, 38.3; **HRMS (ESI)** calculated for $C_{13}H_{14}ON$, $[M+H]^+$ is 200.1070 (100%), 201.1104 (15%), found 200.1071 (100%), 201.1106 (15%).

The data is consistent with the literature.[16]

4-(2,6-Difluorobenzyl)-2-methoxypyridine (5x)

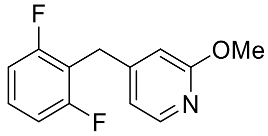

General procedure E was followed using sodium 2,6-difluorobenzyl sulfinate (85.7 mg, 0.40 mmol, 2.0 equiv.) and 4-bromo-2-methoxypyridine (25 µL, 0.20 mmol, 1.0 equiv.). The crude material was purified by column chromatography (10% EtOAc in petrol) to give the product as a colourless oil (16.0 mg, 34%).

General procedure F was followed, using 2,6-difluorobenzyl bromide (62.1 mg, 0.30 mmol, 1.5 equiv.), 4-bromo-2-methoxypyridine (25 µL, 0.20 mmol, 1.0 equiv.) and SMOPS (52.2 mg, 0.30 mmol, 1.5 equiv.). The crude material was purified by column chromatography (10% EtOAc in petrol) to give the title product as a colourless oil (41.8 mg, 89% yield).

^{1}H NMR (400 MHz, $CDCl_3$) δ 8.04 (dd, *J* = 5.5, 0.5 Hz, 1H, Het-*H*), 7.20 (tt, *J* = 8.5, 6.5 Hz, 1H, Ar-*H*), 6.89 (dd, *J* = 8.5, 7.5 Hz, 2H, 2 × Ar-*H*), 6.77 (dt, *J* = 5.5, 0.5 Hz, 1H, Het-*H*), 6.58 (dt, *J* = 1.5, 0.5 Hz, 1H, Het-*H*), 3.95 (s, 2H, Ar-*CH$_2$*-Het), 3.90 (s, 3H, Het-*OCH$_3$*); **^{13}C NMR** (101 MHz, $CDCl_3$) δ 164.7, 161.5 (dd, *J* = 247.5, 8.5 Hz), 150.9, 146.9, 128.7 (t, *J* = 10.0 Hz), 117.3, 115.0 (t, *J* = 20.0 Hz), 111.5 (dd, *J* = 19.0, 6.5 Hz), 110.4, 53.5, 27.6 (t, *J* = 3.0 Hz); **^{19}F NMR** (377 MHz, $CDCl_3$) δ -114.69; **IR** (neat)/ν_{max} 2980, 1611, 1593, 1470, 1451, 1398, 1237, 1152; **HRMS (ESI)** calculated for $C_{13}H_{12}F_2ON$, $[M+H]^+$ is 236.0881 (100%), 237.0915 (15%), found 236.0881 (100%), 237.0916 (15%).

5-Benzylpyridin-3-ol (5y)

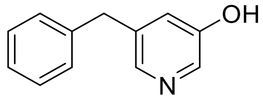

General procedure F was followed, using benzyl bromide (36 µL, 0.30 mmol, 1.5 equiv.), 3-bromo-5-hydroxypyridine (34.8 mg, 0.20 mmol, 1.0 equiv.), and SMOPS (52.2 mg, 0.30 mmol, 1.5 equiv.). After filtration through celite, the filtrate was washed with 5% aqueous LiCl solution until the DMSO was fully removed from the organic phase. The crude material was purified by column chromatography (50 – 80% EtOAc in petrol) to give the title product as a yellow solid (21.4 mg, 58% yield).

^{1}H NMR (400 MHz, $CDCl_3$) δ 10.38 (s, 1H, Het-O*H*), 8.08 (d, *J* = 2.5 Hz, 1H, Het-*H*), 7.92 (d, *J* = 2.0 Hz, 1H, Het-*H*), 7.31 – 7.26 (m, 2H, 2 × Ar-*H*), 7.24 – 7.19 (m, 1H, Ar-*H*), 7.18 – 7.14 (m, 2H, 2 × Ar-*H*), 7.11 (t, *J* = 2.5 Hz, 1H, Het-*H*), 3.93 (s, 2H, Ar-C*H$_2$*-Het); **^{13}C NMR** (101 MHz, $CDCl_3$) δ 155.4, 139.4, 139.2, 139.1, 134.1, 129.0, 128.9, 126.8, 125.8, 39.0; **MP** (CH_2Cl_2) 104 – 106 °C; **IR** (neat)/ν_{max} 3028, 2840, 2642, 1585, 1495, 1441, 1303, 1178; **HRMS (ESI)** calculated for $C_{12}H_{12}ON$, $[M+H]^+$ is 186.0913 (100%), 187.0947 (12%), found 186.0914 (100%), 187.0948 (15%).

Tert-butyl 4-(5-(3-cyanobenzyl)pyridin-2-yl)piperazine-1-carboxylate (5z)

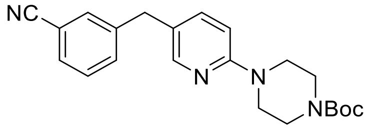

General procedure F was followed, using 3-(bromomethyl)benzonitrile (58.8 mg, 0.30 mmol, 1.5 equiv.), *tert*-butyl 4-(5-bromopyridin-2-yl)piperazine-1-carboxylate (68.4 mg, 0.20 mmol, 1.0 equiv.) and SMOPS (52.0 mg, 0.30 mmol, 1.5 equiv.). The crude residue was purified by column chromatography (20 – 60% EtOAc in petrol) to give the title product as a white solid (75.3 mg, 99% yield).

^{1}H NMR (400 MHz, $CDCl_3$) δ 8.05 – 8.01 (m, 1H, Het-*H*), 7.48 (dt, *J* = 7.0, 2.0 Hz, 1H, Ar-*H*), 7.44 – 7.34 (m, 3H, 2 × Ar-*H*, Het-*H*), 7.26 (dd, *J* = 8.5, 2.5 Hz, 1H, Ar-*H*), 6.60 (dd, *J* = 8.5, 1.0 Hz, 1H, Het-*H*), 3.87 (s, 2H, Ar-C*H*$_2$-Ar), 3.51 (h, *J* = 5.0, 4.5 Hz, 8H, 8 × Alk-*H*), 1.47 (s, 9H, 3 × C*H*$_3$); **^{13}C NMR** (101 MHz, $CDCl_3$) δ 158.5, 154.9, 148.9, 142.5, 138.2, 133.3, 132.3, 130.22, 130.21, 129.5, 124.4, 118.9, 112.8, 107.7, 80.1, 45.43, 45.42, 37.7, 28.6; **MP** ($CDCl_3$) 118 – 119 °C; **IR** (neat)/ν_{max} 2977, 2229, 1692, 1607, 1494, 1405, 1241, 1167; **HRMS (ESI)** calculated for $C_{22}H_{27}O_2N_4$, $[M+H]^+$ is 379.2129 (100%), 380.2161 (25%), found 379.2125 (100%), 380.2160 (25%).

3-((2-Methylpyridin-3-yl)methyl)benzonitrile (5aa)

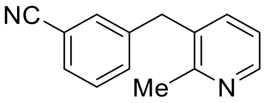

General procedure F was followed, using 3-(bromomethyl)benzonitrile (58.8 mg, 0.30 mmol, 1.5 equiv.), 2-bromo-3-methylpyridine (22 μL, 0.20 mmol, 1.0 equiv.) and SMOPS (52.2 mg, 0.30 mmol, 1.5 equiv.). The crude material was purified by column chromatography (30 – 70% EtOAc in petrol) to give the title product as an off-white solid (39.2 mg, 94% yield).

^{1}H NMR (400 MHz, $CDCl_3$) δ 8.43 (dd, *J* = 5.0, 1.5 Hz, 1H), 7.53 (dt, *J* = 7.5, 1.5 Hz, 1H), 7.43 – 7.33 (m, 4H), 7.14 (dd, *J* = 7.5, 5.0 Hz, 1H), 4.02 (s, 2H), 2.47 (s, 3H); **^{13}C NMR** (101 MHz, $CDCl_3$) δ 157.2, 147.6, 140.7, 137.7, 133.3, 132.8, 132.2, 130.4, 129.6, 121.8, 118.8, 112.9, 38.4, 22.6; **MP** (CH_2Cl_2) 82 – 84 °C; **IR** (neat)/ν_{max} 2993, 2852, 2229, 1734, 1583, 1483; **HRMS (ESI)** calculated for $C_{14}H_{13}N_2$, $[M+H]^+$ is 209.1073 (100%), 210.1107 (15%), found 209.1074 (100%), 210.1108 (15%).

3-((3-Methylpyridin-2-yl)methyl)benzonitrile (5ab)

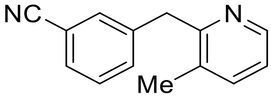

General procedure F was followed, using 3-(bromomethyl)benzonitrile (58.8 mg, 0.30 mmol, 1.5 equiv.), 2-bromo-3-methylpyridine (22 μL, 0.20 mmol, 1.0 equiv.) and SMOPS (52.2 mg, 0.30 mmol, 1.5 equiv.). The crude material was purified by column chromatography (25 – 65% EtOAc in petrol) to give the title product as a colourless oil (40.7 mg, 98% yield).

^{1}H NMR (400 MHz, $CDCl_3$) δ 8.42 (dd, *J* = 5.0, 1.5 Hz, 1H, Het-*H*), 7.49 – 7.43 (m, 4H, 3 × Ar-*H*, Het-*H*), 7.36 (dd, *J* = 8.5, 7.5 Hz, 1H, Ar-*H*), 7.13 (dd, *J* = 7.5, 5.0 Hz, 1H, Het-*H*), 4.20 (s, 2H, Ar-C*H*$_2$-Het), 2.25 (s, 3H, Het-C*H*$_3$); **^{13}C NMR** (101 MHz, $CDCl_3$) δ 157.3, 147.1, 140.6, 138.6, 133.4, 132.3, 131.8, 130.1, 129.3, 122.3, 119.0, 112.6, 41.4, 19.0; **IR** (neat)/ν_{max} 3053, 2229, 1736, 1574, 1466, 1450, 1106; **HRMS (ESI)** calculated for $C_{14}H_{12}N_2$, $[M+H]^+$ is 209.1073 (100%), 210.1107 (15%), found 209.1074 (100%), 210.1108 (15%).

3-((6-Methylpyridin-2-yl)methyl)benzonitrile (5ac)

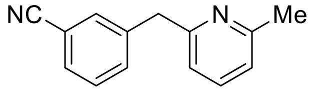

General procedure F was followed, using 3-(bromomethyl)benzonitrile (58.8 mg, 0.30 mmol, 1.5 equiv.), 2-bromo-6-methylpyridine (23 µL, 0.20 mmol, 1.0 equiv.) and SMOPS (52.2 mg, 0.30 mmol, 1.5 equiv.). The crude material was purified by column chromatography (20 – 45% Et_2O in pentane) to give the title product as a yellow solid (34.7 mg, 83% yield).

^{1}H NMR (400 MHz, $CDCl_3$) δ 7.53 – 7.47 (m, 4H, 4 × Ar-*H*), 7.38 (t, *J* = 7.5 Hz, 1H, Het-*H*), 7.02 (d, *J* = 7.5 Hz, 1H, Het-*H*), 6.88 (d, *J* = 7.5 Hz, 1H, Het-*H*), 4.13 (s, 2H, Ar-*CH_2*-Het), 2.54 (s, 3H, Het-*CH_3*); **^{13}C NMR** (101 MHz, $CDCl_3$) δ 158.7, 158.5, 141.3, 137.1, 133.7, 132.6, 130.2, 129.3, 121.4, 120.2, 119.0, 112.6, 44.1, 24.7; **MP** (CH_2Cl_2) 59 - 60 °C; **IR** (neat)/ν_{max} 3064, 2229, 1592, 1576, 1483, 1456; **HRMS (ESI)** calculated for $C_{14}H_{13}N_2$, $[M+H]^+$ is 209.1073 (100%), 210.1107 (15%), found 209.1074 (100%), 210.1109 (15%).

5-Benzyl-2-(trifluoromethyl)pyridine (5ad)

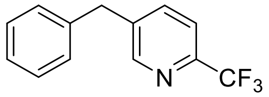

General procedure F was followed, using benzyl bromide (36 µL, 0.30 mmol, 1.5 equiv.), 5-bromo-2-(trifluoromethyl)pyridine (45.2 mg, 0.20 mmol, 1.0 equiv.) and SMOPS (52.2 mg, 0.30 mmol, 1.5 equiv.). The crude material was purified by column chromatography (2 – 10% EtOAc in petrol) to give the title product as a colourless oil (38.0 mg, 80% yield).

^{1}H NMR (400 MHz, $CDCl_3$) δ 8.62 (d, *J* = 2.0 Hz, 1H, Het-*H*), 7.63 (dd, *J* = 8.0, 2.0 Hz, 1H, Het-*H*), 7.59 (dd, *J* = 8.0, 1.0 Hz, 1H, Het-*H*), 7.36 – 7.30 (m, 2H, 2 × Ar-*H*), 7.28 – 7.24 (m, 1H, Ar-*H*), 7.20 – 7.16 (m, 2H, 2 × Ar-*H*), 4.06 (s, 2H, Ar-*CH_2*-Het); **^{13}C NMR** (101 MHz, $CDCl_3$) 150.5, 146.9 (d, *J* = 34.5 Hz), 140.1, 138.8, 137.6, 129.1, 129.0, 127.0, 121.8 (q, *J* = 273.2 Hz), 120.4 (q, *J* = 2.5 Hz), 39.0; **^{19}F NMR** (377 MHz, $CDCl_3$) δ -67.73; **IR** (neat)/ν_{max} 3029, 2924, 1496, 1336, 1176, 1130, 1085, 1028; **HRMS (ESI)** calculated for $C_{13}H_{11}F_3N$, $[M+H]^+$ is 238.0838 (100%), 239.0872 (15%), found 238.0838 (100%), 239.0872 (17%).

The data is consistent with the literature.[17]

Methyl 6-(3-cyanobenzyl)nicotinate (5ae)

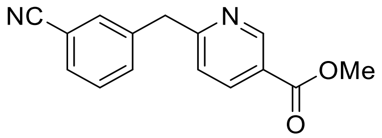

General procedure F was followed, using 3-(bromomethyl)benzonitrile (58.8 mg, 0.30 mmol, 1.5 equiv.), 6-chloropyridine-3-carboxylate (34.3 mg, 0.20 mmol, 1.0 equiv.) and SMOPS (52.2 mg, 0.30 mmol, 1.5 equiv.). The crude material was purified by column chromatography (10 – 40% EtOAc in petrol) to give the title product as a colourless oil (22.9 mg, 45% yield).

^{1}H NMR (400 MHz, $CDCl_3$) δ 9.16 (d, *J* = 2.0 Hz, 1H, Het-*H*), 8.24 (dd, *J* = 8.0, 2.0 Hz, 1H, Het-*H*), 7.59 – 7.50 (m, 3H, Het-*H*, 2 × Ar-*H*), 7.42 (t, *J* = 8.0 Hz, 1H, Ar-*H*), 7.22 (d, *J* = 8.0 Hz, 1H, Ar-*H*), 4.24 (s, 2H, Ar-*CH_2*-Het), 3.94 (s, 3H, Het-COO*CH_3*); **^{13}C NMR** (101 MHz, $CDCl_3$) δ 165.6, 163.6, 150.8, 140.1, 138.4, 133.8, 132.7, 130.7, 129.7, 124.6, 123.1, 118.8, 113.0, 52.6, 44.0; **IR** (neat)/ν_{max} 2953, 2229, 1722, 1596, 1434, 1292, 1195, 1117, 1026; **HRMS (ESI)** calculated for $C_{15}H_{13}O_2N_2$, $[M+H]^+$ is 253.0972 (100%), 254.1005 (17%), found 253.0973 (100%), 254.1006 (17%).

3-(Pyrazin-2-ylmethyl)benzonitrile (5af)

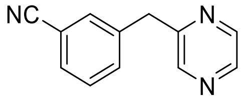

General procedure F was followed, using 3-(bromomethyl)benzonitrile (58.8 mg, 0.30 mmol, 1.5 equiv.), 2-chloropyrazine (18 μL, 0.20 mmol, 1.0 equiv.) and SMOPS (52.2 mg, 0.30 mmol, 1.5 equiv.). The crude material was purified by column chromatography (25 – 65% EtOAc in petrol) to give the title product as a yellow oil (34.2 mg, 88% yield).

^{1}H NMR (400 MHz, $CDCl_3$) δ 8.54 – 8.44 (m, 3H, 3 × Het-*H*), 7.61 – 7.56 (m, 1H, Ar-*H*), 7.56 – 7.50 (m, 2H, 2 × Ar-*H*), 7.42 (t, *J* = 7.5 Hz, 1H, Ar-*H*), 4.19 (s, 2H, Ar-C*H*$_2$-Het); **^{13}C NMR** (101 MHz, $CDCl_3$) δ 155.1, 144.7, 144.5, 143.1, 139.7, 133.7, 132.7, 130.7, 129.7, 118.8, 113.0, 41.3; **IR** (neat)/ν_{max} 3058, 2229, 1734, 1474, 1434, 1403, 1057, 1018; **HRMS (ESI)** calculated for $C_{12}H_9N_3$, $[M+H]^+$ is 196.0877 (100%), 197.0903 (12%), found 196.0871 (100%), 197.0905 (15%).

3-((2-Methoxypyrimidin-5-yl)methyl)benzonitrile (5ag)

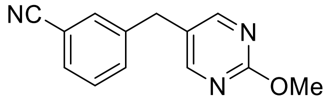

General procedure E was followed using sodium (3-cyanophenyl)methanesulfinate (81.3 mg, 0.40 mmol, 2.0 equiv.) and 5-bromo-2-methoxypyrimidine (37.8 mg, 0.20 mmol, 1.0 equiv.). The crude material was purified by column chromatography (30 – 70% EtOAc in petrol) to give the product as an off-white solid (44.6 mg, 99%).

General procedure F was followed, using 3-(bromomethyl)benzonitrile (58.8 mg, 0.30 mmol, 1.5 equiv.), 5-bromo-2-methoxypyrimidine (37.8 mg, 0.20 mmol, 1.0 equiv.) and SMOPS (52.2 mg, 0.30 mmol, 1.5 equiv.). The crude material was purified by column chromatography (30 – 70% EtOAc in petrol) to give the title product as an off-white solid (42.9 mg, 95% yield).

^{1}H NMR (400 MHz, $CDCl_3$) δ 8.34 (s, 2H, 2 × Het-*H*), 7.54 (dt, *J* = 7.0, 1.5 Hz, 1H, Ar-*H*), 7.46 (ddt, *J* = 2.5, 1.5, 1.0 Hz, 1H, Ar-*H*), 7.44 – 7.38 (m, 2H, 2 × Ar-*H*), 4.00 (s, 3H, Het-OC*H*$_3$), 3.94 (s, 2H, Ar-C*H*$_2$-Het); **^{13}C NMR** (101 MHz, $CDCl_3$) δ 164.9, 159.4, 140.8, 133.2, 132.2, 130.7, 129.8, 126.0, 118.6, 113.2, 55.1, 35.2; **MP** ($CDCl_3$) 96 – 98 °C; **IR** (neat)/ν_{max} 2926, 2228, 2160, 1596, 1477, 1452, 1322, 1291, 1048; **HRMS (ESI)** calculated for $C_{13}H_{12}N_3O$, $[M+H]^+$ is 226.0975 (100%), 227.1009 (15%), found 226.0975 (100%), 227.1010 (15%).

4-(3,5-Difluorobenzyl)-6-methyl-2-phenylpyrimidine (5ah)

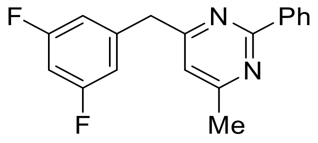

General procedure F was followed, using 3,5-difluorobenzyl bromide (39 μL, 0.30 mmol, 1.5 equiv.), 4-chloro-6-methyl-2-phenyl-pyrimidine (40.9 mg, 0.20 mmol, 1.0 equiv.) and β-nitrile sulfinate (42.3 mg, 0.30 mmol, 1.5 equiv.). The crude residue was purified by column chromatography (5 – 50% EtOAc in petrol) to give the product as a white crystalline solid (39.4 mg, 66% yield).

^{1}H NMR (400 MHz, $CDCl_3$) δ 8.49 – 8.44 (m, 2H, 2 × Ar-*H*), 7.48 (dd, *J* = 5.0, 2.0 Hz, 3H, 3 × Ar-*H*), 6.87 (dt, *J* = 6.5, 2.0 Hz, 2H, 2 × Ar-*H*), 6.84 (s, 1H, Het-*H*), 6.71 (tt, *J* = 9.0, 2.0 Hz, 1H, Ar-*H*), 4.08 (s, 2H, Ar-C*H*$_2$-Het), 2.54 (s, 3H, Het-C*H*$_3$); **^{13}C NMR** (101 MHz, $CDCl_3$) δ 167.9, 167.6, 164.4, 163.2 (dd, *J* = 248.5, 13.0 Hz), 141.8 (t, *J* = 9.0 Hz), 137.8, 130.7, 128.6, 128.5, 117.6, 112.3 (dd, *J* = 19.0, 6.5 Hz), 102.5 (t, *J* = 25.5 Hz), 43.9 (t, *J* = 2.0 Hz), 24.5; **^{19}F NMR** (377 MHz, $CDCl_3$) δ -109.80; **MP** (CH_2Cl_2) 78 – 80 °C; **IR** (neat)/ν_{max} 3065, 1626, 1592, 1573, 1543, 1460, 1371, 1322, 1118; **HRMS (ESI)** calculated for $C_{18}H_{15}F_2N_2$, $[M+H]^+$ is 297.1198 (100%), 298.1231 (20%), found 297.1197 (100%), 298.1230 (20%).

3-((2,6-Dimorpholinopyrimidin-4-yl)methyl)benzonitrile (5ai)

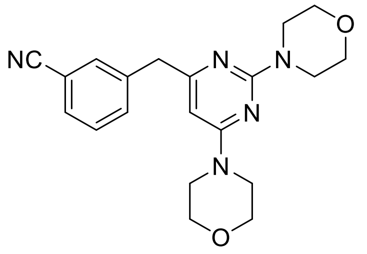

General procedure F was followed, using 3-(bromomethyl)benzonitrile (58.8 mg, 0.30 mmol, 1.5 equiv.), 4,4'-(6-chloropyrimidine-2,4-diyl)dimorpholine (56.9 mg, 0.20 mmol, 1.0 equiv.) and SMOPS (52.2 mg, 0.30 mmol, 1.5 equiv.). The crude material was purified by column chromatography (20 – 60% EtOAc in petrol) to give the title product as an off-white solid (68.6 mg, 94% yield).

^{1}H NMR (400 MHz, $CDCl_3$) δ 7.57 (s, 1H, Ar-*H*), 7.53 (d, *J* = 8.0 Hz, 1H, Ar-*H*), 7.49 (dt, *J* = 8.0, 1.5 Hz, 1H, Ar-*H*), 7.37 (t, *J* = 8.0 Hz, 1H, Ar-*H*), 5.67 (s, 1H, Het-*H*), 3.79 (s, 2H, Ar-C*H$_2$*-Het), 3.76 – 3.72 (m, 12H, 12 × Alk-*H*), 3.51 (t, *J* = 5.0 Hz, 4H, 4 × Alk-*H*); **^{13}C NMR** (101 MHz, $CDCl_3$) δ 167.2, 163.6, 161.7, 140.6, 133.8, 132.8, 130.2, 129.1, 119.1, 112.4, 91.7, 67.0, 66.6, 44.4, 44.3, 44.0; **MP** (CH_2Cl_2) 96 – 100 °C; **IR** (neat)/ν_{max} 2894, 2229, 1734, 1573, 1468, 1440, 1413, 1365, 1264, 1244, 1192, 1116; **HRMS (ESI)** calculated for $C_{20}H_{24}O_2N_5$, $[M+H]^+$ is 366.1925 (100%), 367.1957 (22%), found 366.1919 (100%), 367.1950 (25%).

3-((4-Methylthiophen-3-yl)methyl)benzonitrile (5aj)

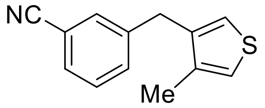

General procedure F was followed, using 3-(bromomethyl)benzonitrile (58.8 mg, 0.30 mmol, 1.5 equiv.), 3-bromo-4-methylthiophene (22 µL, 0.20 mmol, 1.0 equiv.) and SMOPS (52.2 mg, 0.30 mmol, 1.5 equiv.). The crude material was purified by column chromatography (5 – 35% EtOAc in petrol) to give the title product as a pale yellow oil (38.4 mg, 90% yield).

^{1}H NMR (400 MHz, $CDCl_3$) δ 7.51 (dt, *J* = 6.5, 2.0 Hz, 1H, Ar-*H*), 7.45 – 7.39 (m, 3H, 3 × Ar-*H*), 6.96 (dd, *J* = 3.0, 1.0 Hz, 1H, Het-*H*), 6.85 (d, *J* = 3.0 Hz, 1H, Het-*H*), 3.92 (s, 2H, Ar-C*H$_2$*-Het), 2.09 (d, *J* = 1.0 Hz, 3H, Het-C*H$_3$*); **^{13}C NMR** (101 MHz, $CDCl_3$) δ 141.7, 138.9, 136.8, 133.3, 132.3, 130.1, 129.3, 122.9, 122.2, 119.0, 112.6, 35.0, 14.6; **IR** (neat)/ν_{max} 3097, 2920, 2229, 1483, 1444, 1431; **HRMS (ESI)** calculated for $C_{13}H_{12}NS$, $[M+H]^+$ is 214.0685 (100%), 215.0718 (15%), found 214.0688 (100%), 215.0719 (10%).

3-((3,5-Dimethylisoxazol-4-yl)methyl)benzonitrile (5ak)

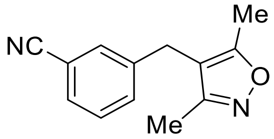

General procedure F was followed, using 3-(bromomethyl)benzonitrile (58.8 mg, 0.30 mmol, 1.5 equiv.), 4-bromo-3,5-dimethylisoxazole (24 µL, 0.20 mmol, 1.0 equiv.) and SMOPS (52.2 mg, 0.30 mmol, 1.5 equiv.). The crude material was purified by column chromatography (20 – 45% EtOAc in petrol) to give the title product as a colourless oil (30.6 mg, 72% yield).

^{1}H NMR (400 MHz, $CDCl_3$) δ 7.54 – 7.50 (m, 1H, Ar-*H*), 7.43 – 7.38 (m, 2H, 2 × Ar-*H*), 7.35 – 7.31 (m, 1H, Ar-*H*), 3.71 (s, 2H, Ar-*CH$_2$*-Het), 2.32 (s, 3H, Het-*CH$_3$*), 2.07 (s, 3H, Het-*CH$_3$*); **^{13}C NMR** (101 MHz, $CDCl_3$) δ 166.1, 159.7, 140.5, 132.6, 131.6, 130.5, 129.7, 118.7, 113.0, 111.2, 29.8, 27.9, 11.2, 10.4; **IR** (neat)/ν_{max} 2956, 2849, 2231, 1734, 1640, 1483, 1381, 1261, 1232, 1195, 1095; **HRMS (ESI)** calculated for $C_{13}H_{13}ON_2$, $[M+H]^+$ is 213.1022 (100%), 214.1056 (15%), found 213.1024 (30%), 214.0897 (100%).

3-((1-Phenyl-1H-pyrazol-3-yl)methyl)benzonitrile (5al)

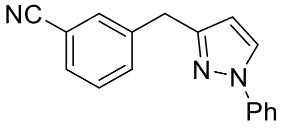

General procedure F was followed, using 3-(bromomethyl)benzonitrile (58.8 mg, 0.30 mmol, 1.5 equiv.), 3-iodo-1-phenyl-1H-pyrazole (31 µL, 0.20 mmol, 1.0 equiv.) and SMOPS (52.2 mg, 0.30 mmol, 1.5 equiv.). The crude material was purified by column chromatography (15 – 30% EtOAc in petrol) to give the title product as a white solid (45.8 mg, 88% yield).

^{1}H NMR (400 MHz, DMSO) δ 8.41 (d, J = 2.5 Hz, 1H, Het-*H*), 7.83 – 7.74 (m, 3H, 3 × Ar-*H*), 7.71 – 7.61 (m, 2H, 2 × Ar-*H*), 7.53 (dd, J = 7.5, 0.5 Hz, 1H, Ar-*H*), 7.51 – 7.44 (m, 2H, 2 × Ar-*H*), 7.31 – 7.23 (m, 1H, Ar-*H*), 6.38 (d, J = 2.5 Hz, 1H, Het-*H*), 4.07 (s, 2H, Ar-C*H$_2$*-Het); **^{13}C NMR** (101 MHz, $CDCl_3$) δ 152.2, 141.3, 140.1, 133.5, 132.5, 130.2, 129.6, 129.4, 128.1, 126.6, 119.3, 119.1, 112.6, 107.2, 34.4; **MP** (CH_2Cl_2) 64 – 66 °C; **IR** (neat)/ν_{max} 2921, 2229, 1737, 1599, 1483, 1389, 1047; **HRMS (ESI)** calculated for $C_{17}H_{13}N_3$, $[M+H]^+$ is 260.1182 (100%), 261.1216 (20%), found 260.1183 (100%), 261.1217 (20%).

3-(Imidazo[1,2-a]pyrazin-3-ylmethyl)benzonitrile (5am)

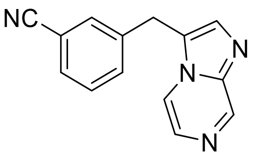

General procedure F was followed, using 3-(bromomethyl)benzonitrile (58.8 mg, 0.30 mmol, 1.5 equiv.), 3-bromoimidazo[1,2-a]pyrazine (39.6 mg, 0.20 mmol, 1.0 equiv.) and SMOPS (52.2 mg, 0.30 mmol, 1.5 equiv.). The crude material was purified by column chromatography (80 – 100% EtOAc in petrol) to give the title product as an orange solid (38.1 mg, 81% yield).

^{1}H NMR (400 MHz, $CDCl_3$) δ 9.11 (d, *J* = 1.5 Hz, 1H, Het-*H*), 7.86 (d, *J* = 5.0 Hz, 1H, Het-*H*), 7.69 (dd, *J* = 5.0, 1.5 Hz, 1H, Het-*H*), 7.64 (s, 1H, Het-*H*), 7.58 (dt, *J* = 7.5, 1.5 Hz, 1H, Ar-*H*), 7.50 – 7.48 (m, 1H, Ar-*H*), 7.45 (t, *J* = 7.5 Hz, 1H, Ar-*H*), 7.40 (dt, *J* = 7.5, 1.5 Hz, 1H, Ar-*H*), 4.32 (s, 2H, Ar-C*H$_2$*-Het); **^{13}C NMR** (101 MHz, $CDCl_3$) δ 144.4, 141.5, 137.4, 135.2, 132.8, 131.9, 131.3, 130.1, 129.8, 122.9, 118.4, 116.1, 113.4, 29.6; **MP** (CH_2Cl_2) 148 – 150 °C; **IR** (neat)/ν_{max} 2954, 2229, 1734, 1558, 1522, 1486, 1379, 1341, 1146; **HRMS (ESI)** calculated for $C_{14}H_{10}N_4$, $[M+H]^+$ is 235.0978 (100%), 236.1012 (15%), found 235.0978 (100%), 236.1012 (15%).

6-Benzylimidazo[1,2-a]pyridine (5an)

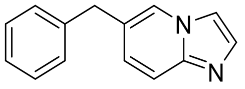

General procedure F was followed, using benzyl bromide (36 µL, 0.30 mmol, 1.5 equiv.), 6-bromoimidazo[1,2-α]pyridine (39.4 mg, 0.20 mmol, 1.0 equiv.) and β-nitrile sulfinate (46.6 mg, 0.33 mmol, 1.65 equiv.). Due to the polar nature of the product, the crude material was dissolved into EtOAc (50 mL) and washed with 5% aqueous LiCl solution (3 × 10 mL) until full removal of DMSO from the organic layer was achieved. The organic layer was dried over $MgSO_4$ and the solvents removed under vacuum. The residue was purified by column chromatography (10 – 60% EtOAc in petrol) to give the product as an orange oil (33.3 mg, 80% yield).

^{1}H NMR (400 MHz, $CDCl_3$) δ 7.87 (dq, *J* = 2.0, 1.0 Hz, 1H, Het-*H*), 7.59 (d, *J* = 1.0 Hz, 1H, Het-*H*), 7.54 (d, *J* = 9.0 Hz, 1H, Het-*H*), 7.49 (t, *J* = 1.0 Hz, 1H, Het-*H*), 7.33 (tt, *J* = 6.8, 1.0 Hz, 2H, 2 × Ar-*H*), 7.26 – 7.23 (m, 1H, Ar-*H*), 7.23 – 7.18 (m, 2H, 2 × Ar-*H*), 7.02 (dd, *J* = 9.0, 2.0 Hz, 1H, Het-*H*), 3.94 (s, 2H, Ar-*CH$_2$*-Het); **^{13}C NMR** (101 MHz, $CDCl_3$) δ 144.7, 139.4, 133.5, 129.0, 128.9, 127.0, 126.8, 125.8, 124.1, 117.6, 112.5, 38.7; **IR** (neat)/ν_{max} 2918, 1733, 1532, 1494, 1479, 1454, 1312, 1148; **HRMS (ESI)** calculated for $C_{14}H_{12}N_2$, $[M+H]^+$ is 209.1073, found 209.1074.

3-((2-Phenylimidazo[1,2-a]pyridin-3-yl)methyl)benzonitrile (5ao)

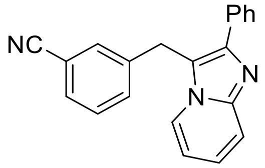

General procedure F was followed, using 3-(bromomethyl)benzonitrile (58.8 mg, 0.30 mmol, 1.5 equiv.), 3-bromo-2-phenylimidazo[1,2-a]pyridine (54.6 mg, 0.20 mmol, 1.0 equiv.) and SMOPS (52.2 mg, 0.30 mmol, 1.5 equiv.). The crude material was purified by column chromatography (40 – 100% EtOAc in petrol) to give the title product as an orange oil (34.4 mg, 56% yield).

^{1}H NMR (400 MHz, $CDCl_3$) δ 7.75 – 7.70 (m, 3H, Het-*H*, 2 × Ar-*H*), 7.64 (dt, *J* = 7.0, 1.5 Hz, 1H, Het-*H*), 7.55 (dt, *J* = 7.5, 1.5 Hz, 1H, Het-*H*), 7.46 – 7.31 (m, 6H, 6 × Ar-*H*), 7.25 – 7.21 (m, 1H, Ar-*H*), 6.79 (td, *J* = 7.0, 1.5 Hz, 1H, Het-*H*), 4.54 (s, 2H, Ar-C*H$_2$*-Het); **^{13}C NMR** (101 MHz, $CDCl_3$) δ 145.2, 144.7, 138.7, 134.1, 132.2, 131.4, 131.0, 130.1, 128.9, 128.3, 128.2, 124.8, 123.0, 118.6, 118.0, 116.16, 113.4, 112.9, 29.6; **IR** (neat)/v_{max} 2927, 2230, 1735, 1503, 1483, 1446, 1391, 1359; **HRMS (ESI)** calculated for $C_{21}H_{15}N_3$, $[M+H]^+$ is 310.1339 (100%), 311.1372 (25%), found 310.1337 (100%), 311.1370 (25%).

5-(3,5-Difluorobenzyl)-1-methyl-1H-indazole (5ap)

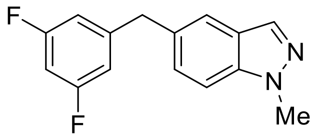

General procedure F was followed, using 1-(bromomethyl)-3,5-difluorobenzene (52 µL, 0.40 mmol, 2.0 equiv.), 5-bromo-1-methyl-1H-indazole (42.2 mg, 0.20 mmol, 1.0 equiv.), β-nitrile sulfinate (62.1 mg, 0.44 mmol, 2.2 equiv.) and DMSO (1.0 mL, 0.2 M). The crude residue was purified by column chromatography (10 – 40% EtOAc in petrol) to give the product as an off-white solid (46.4 mg, 90% yield).

^{1}H NMR (400 MHz, $CDCl_3$) δ 7.93 (d, *J* = 1.0 Hz, 1H, Het-*H*), 7.51 (dq, *J* = 1.5, 1.0 Hz, 1H, Het-*H*), 7.34 (dt, *J* = 8.5, 1.0 Hz, 1H, Het-*H*), 7.19 (dd, *J* = 8.5, 1.5 Hz, 1H, Het-*H*), 6.73 – 6.67 (m, 2H, 2 × Ar-*H*), 6.64 (tt, *J* = 9.0, 2.5 Hz, 1H, Ar-*H*), 4.06 (s, 3H, -N-*CH$_3$*), 4.05 (s, 2H, Ar-*CH$_2$*-Het); **^{13}C NMR** (101 MHz, $CDCl_3$) δ 163.2 (dd, *J* = 248.0, 13.0 Hz), 145.7 (t, *J* = 9.0 Hz), 139.1, 132.6, 131.7, 127.9, 124.5, 120.8, 111.7 (dd, *J* = 18.0, 6.5 Hz), 109.4, 101.7 (t, *J* = 25.5 Hz), 41.5 (t, *J* = 2.0 Hz), 35.7; **^{19}F NMR** (377 MHz, $CDCl_3$) δ -110.31; **MP** (CH_2Cl_2) 74 °C; **IR** (neat)/v_{max} 2929, 2852, 1623, 1594, 1509, 1458, 1321, 1225, 1116; **HRMS (ESI)** calculated for $C_{15}H_{13}F_2N_2$, $[M+H]^+$ is 259.1041 (100%), 260.1075 (15%), found 259.1041 (100%), 260.1075 (15%).

5-(3,5-Difluorobenzyl)-1H-indazole (5aq)

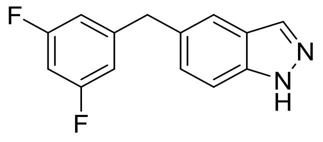

General procedure F was followed, using 1-(bromomethyl)-3,5-difluorobenzene (39 µL, 0.30 mmol, 1.5 equiv.), 5-bromo-1H-indazole (39.4 mg, 0.20 mmol, 1.0 equiv.) and SMOPS (52.2 mg, 0.30 mmol, 1.5 equiv.). The crude residue was first purified by column chromatography (10 – 50 % EtOAc in petrol) to give the product as an impure off-white solid. This resulting solid was further purified by preparative TLC (40% EtOAc in toluene) to give the product as a white solid (21.7 mg, 44% yield).

^{1}H NMR (400 MHz, $CDCl_3$) δ 9.59 (s, 1H, N*H*), 8.06 (s, 1H, Het-*H*), 7.56 (d, *J* = 1.0 Hz, 1H, Het-*H*), 7.46 (d, *J* = 9.0 Hz, 1H, Het-*H*), 7.21 (dd, *J* = 8.5, 1.5 Hz, 1H, Het-*H*), 6.71 (dt, *J* = 7.0, 2.0 Hz, 2H, 2 × Ar-*H*), 6.65 (tt, *J* = 9.0, 2.0 Hz, 1H, Ar-*H*), 4.06 (s, 2H); **^{13}C NMR** (101 MHz, $CDCl_3$) δ 163.2 (dd, *J* = 248.5, 13.0 Hz), 145.5 (t, *J* = 9.0 Hz), 139.3, 134.6, 132.5, 128.7, 123.7, 120.6, 111.8 (dd, *J* = 18.5, 6.5 Hz), 110.3, 101.8 (t, *J* = 25.5 Hz), 41.6 (t, *J* = 2.0 Hz); **^{19}F NMR** (377 MHz, $CDCl_3$) δ -110.25; **MP** (CH_2Cl_2) 45 – 47 °C; **IR** (neat)/v_{max} 3055, 2918, 2849, 1595, 1459, 1354, 1318, 1116; **HRMS (ESI)** calculated for $C_{14}H_{11}N_2F_2$, $[M+H]^+$ is 245.0885 (100%), 246.0918 (15%), found 245.0885 (100%), 246.0919 (15%).

4-(4-(Tert-butyl)benzyl)-1H-indazole (5ar)

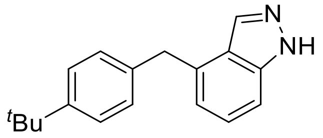

General procedure F was followed, using 4-*tert*-butylbenzyl bromide (55 µL, 0.30 mmol, 1.5 equiv.), 4-bromo-1H-indazole (39.4 mg, 0.20 mmol, 1.0 equiv.) and β-nitrile sulfinate (46.6 mg, 0.33 mmol, 1.65 equiv.). The crude residue was first purified by column chromatography (10 – 30% EtOAc in petrol) to give the product as an impure yellow oil (32.8 mg). This resulting solid was resubmitted to column chromatography (20 – 40% Et_2O in petrol) to give the product as a pale yellow solid (24.2 mg, 46% yield).

^{1}H NMR (400 MHz, $CDCl_3$) δ 9.49 (s, 1H, N*H*), 8.13 (s, 1H, Het-*H*), 8.05 (s, 1H, Het-*H*), 7.36 (d, *J* = 6.5 Hz, 1H, Het-*H*), 7.32 – 7.29 (m, 2H, 2 × Ar-*H*), 7.18 (d, *J* = 8.0 Hz, 2H, 2 × Ar-*H*), 6.98 (dd, *J* = 6.5, 1.0 Hz, 1H, Het-*H*), 4.28 (s, 2H, C*H*$_2$), 1.30 (s, 9H, 3 × C*H*$_3$); **^{13}C NMR (**101 MHz, $CDCl_3$) δ 149.3, 137.1, 134.9, 128.6, 127.9, 127.4, 125.6, 124.1, 121.3, 109.1, 108.0, 39.2, 34.5, 31.5; **MP** (CH_2Cl_2) 106 - 110 °C; **IR** (neat)/ν_{max} 2962, 2866, 1619, 1515, 1390, 1203; **HRMS (ESI)** calculated for $C_{18}H_{20}N_2$, $[M+H]^+$ is 265.1699, found 265.1700.

3-(4-Methylbenzyl)benzonitrile (5as)

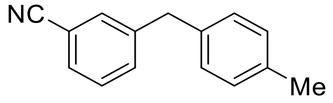

General procedure F was followed, using 3-(bromomethyl)benzonitrile (58.8 mg, 0.30 mmol, 1.5 equiv.), 4-bromotoluene (34.2 mg, 0.20 mmol, 1.0 equiv.) and β-nitrile sulfinate (46.6 mg, 0.33 mmol, 1.65 equiv.). The crude residue was purified by column chromatography (10 – 30% EtOAc in petrol) to give the product as a white crystalline solid (34.2 mg, 82% yield).

^{1}H NMR (400 MHz, $CDCl_3$) δ 7.53 – 7.34 (m, 4H, 4 × Ar-*H*), 7.14 (d, *J* = 8.0 Hz, 2H, 2 × Ar-*H*), 7.06 (d, *J* = 8.0 Hz, 2H, 2 × Ar-*H*), 3.97 (s, 2H, Ar-C*H*$_2$-Ar), 2.34 (s, 3H, Ar-C*H*$_3$); **^{13}C NMR** (101 MHz, $CDCl_3$) δ 143.1, 136.5, 136.4, 133.5, 132.4, 130.0, 129.6, 129.3, 128.9, 119.1, 112.6, 41.1, 21.1; **MP** (CH_2Cl_2) 49 – 50 °C; **IR** (neat)/ν_{max} 2922, 2229, 1600, 1583, 1483, 1436; **HRMS (ESI)** calculated for $C_{15}H_{14}N$, $[M+H]^+$ is 208.1121 (100%), found 208.1122 (100%).

3-((2,3-Dihydrobenzo[b][1,4]dioxin-6-yl)methyl)benzonitrile (5at)

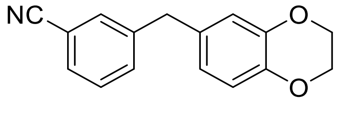

General procedure F was followed, using 3-(bromomethyl)benzonitrile (58.8 mg, 0.30 mmol, 1.5 equiv.), 6-Bromo-1,4-benzodioxane (27 µL, 0.20 mmol, 1.0 equiv.) and SMOPS (52.2 mg, 0.30 mmol, 1.5 equiv.). The crude residue was purified by column chromatography (5 – 30% EtOAc in petrol) to give the product as a clear oil (50.2 mg, 99% yield).

^{1}H NMR (400 MHz, $CDCl_3$) δ 7.48 (dt, *J* = 7.5, 1.5 Hz, 1H, Ar-*H*), 7.46 – 7.34 (m, 3H, 3 × Ar-*H*), 6.80 (d, *J* = 8.0 Hz, 1H, Ar-*H*), 6.66 – 6.59 (m, 2H, 2 × Ar-*H*), 4.24 (s, 4H, 2 × Ar-OC*H*$_2$), 3.89 (s, 2H, Ar-C*H*$_2$-Ar); **^{13}C NMR** (101 MHz, $CDCl_3$) δ 143.7, 142.9, 142.4, 133.4, 132.8, 132.4, 130.0, 129.3, 121.9, 119.1, 117.7, 117.6, 112.6, 64.5, 64.4, 40.8; **IR** (neat)/ν_{max} 2875, 2229, 1589, 1506, 1285, 1068; **HRMS (ESI)** calculated for $C_{16}H_{14}NO_2$, $[M+H]^+$ is 252.1019 (100%), 253.1053 (18%), found 252.1021 (95%), 253.1054 (15%).

3-(2-Phenylallyl)benzonitrile (5au)

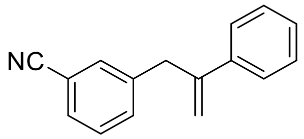

General procedure F was followed, using 3-(bromomethyl)benzonitrile (58.8 mg, 0.30 mmol, 1.5 equiv.), α-bromostyrene (26 μL, 0.20 mmol, 1.0 equiv.) and SMOPS (52.2 mg, 0.30 mmol, 1.5 equiv.). The crude residue was purified by column chromatography (5 – 20% EtOAc in pentane) to give the title product as a yellow oil (14.7 mg, 34% yield).

^{1}H NMR (400 MHz, $CDCl_3$) δ 7.52 (d, *J* = 1.5 Hz, 1H, Ar-*H*), 7.49 – 7.44 (m, 2H, 2 × Ar-*H*), 7.41 – 7.27 (m, 6H, 6 × Ar-*H*), 5.54 (d, *J* = 1.5 Hz, 1H, C*H*H'), 5.06 (q, *J* = 1.5 Hz, 1H, CH*H'*), 3.87 (s, 2H, Ar-CH_2-R); **^{13}C NMR** (101 MHz, $CDCl_3$) δ 145.9, 141.2, 140.0, 133.5, 132.5, 130.2, 129.3, 128.6, 128.0, 126.2, 119.1, 115.6, 112.6, 41.3; **IR** (neat)/ν_{max} 3062, 2917, 2849, 2230, 1600, 1484, 800.

The data is consistent with the literature.[18]

3-(((8R,9S,13S,14S)-13-Methyl-17-oxo-7,8,9,11,12,13,14,15,16,17-decahydro-6H-cyclopenta[a]phenanthren-3-yl)methyl)benzonitrile (5av)

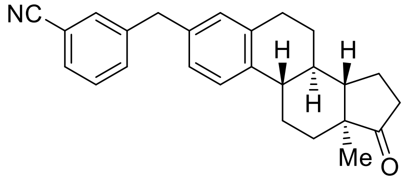

General procedure F was followed, using 3-(bromomethyl)benzonitrile (58.8 mg, 0.30 mmol, 1.5 equiv.) and estrone triflate (3-trifluoromethylsulfonate-estra-1,3,5(10)-triene-17-one) (80.5 mg, 0.20 mmol, 1.0 equiv.) and β-nitrile sulfinate (42.3 mg, 0.30 mmol, 1.5 equiv.). The crude residue was purified by column chromatography (10 – 40% Et_2O in petrol) to give the title product as a colourless oil (72.2 mg, 98% yield).

^{1}H NMR (400 MHz, $CDCl_3$) δ 7.49 (dt, *J* = 7.5, 1.5 Hz, 1H, Ar-*H*), 7.45 (dd, *J* = 6.0, 1.5 Hz, 2H, 2 × Ar-*H*), 7.38 (dd, *J* = 8.5, 7.5 Hz, 1H, Ar-*H*), 7.24 (d, *J* = 8.0 Hz, 1H, Ar-*H*), 6.95 (dd, *J* = 8.0, 2.0 Hz, 1H, Ar-*H*), 6.89 (d, *J* = 2.0 Hz, 1H, Ar-*H*), 3.94 (s, 2H, Ar-*CH_2*-Ar), 2.89 (dd, *J* = 8.5, 4.0 Hz, 2H, Ar-*CH_2*-CH_2-), 2.56 – 2.45 (m, 1H, -CO-*C(H')*H-), 2.46 – 2.37 (m, 1H, Ar-*CH*(CHR_2)CH_2-), 2.29 (dq, *J* = 10.5, 4.5 Hz, 1H, -CO-CH'*(H")*-), 2.21 – 1.89 (m, 4H, -CO-CH'(H")-*CH'(H")*, Ar-CH(CHR_2)CH-CH'*(H")*-CH'*(H")*-), 1.71 – 1.37 (m, 6H, Ar-CH(CHR_2)CH-*CH'*(H")-*CH'*(H")-, Ar-CH$_2$-*CH_2*-, CO-CH'(H")-CH'(H")-*CH*R-*CH*R-), 0.92 (s, 3H, Alk-*CH_3*); **^{13}C NMR** (101 MHz, $CDCl_3$) δ 220.9, 142.9, 138.3, 137.1, 137.0, 133.5, 132.5, 130.0, 129.6, 129.3, 126.4, 125.9, 119.1, 112.6, 50.6, 48.1, 44.4, 41.1, 38.2, 36.0, 31.7, 29.5, 26.6, 25.8, 21.7, 14.0; **IR** (neat)/ν_{max} 2928, 2228, 1736, 1482, 1454, 1084, 1053; **HRMS (ESI)** calculated for $C_{26}H_{28}ON$, $[M+H]^+$ is 370.2165, found 370.2166; **$[\alpha]_D^{25}$** = +110.7° (*c* = 1.0, $CHCl_3$).

3-(4-(5-(*p*-Tolyl)-3-(trifluoromethyl)-1H-pyrazol-1-yl)benzyl)benzonitrile (5aw)

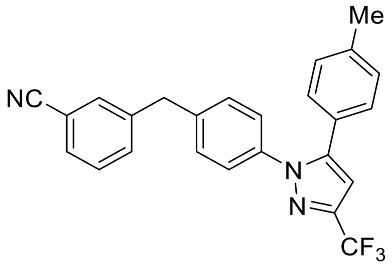

General procedure F was followed, using 3-(bromomethyl)benzonitrile (58.8 mg, 0.30 mmol) and 1-(4-bromophenyl)-4-(p-tolyl)-3-(trifluoromethyl)-1H-pyrazole (76.2 g, 0.20 mmol) and β-nitrile sulfinate (42.3 mg, 0.30 mmol, 1.5 equiv.). The crude reaction mixture was purified by column chromatography (10 – 50% Et_2O in petrol) to give the title product as a colourless oil (82.5 mg, 99% yield).

^{1}H NMR (400 MHz, $CDCl_3$) δ 7.51 (m, 1H, Ar-*H*), 7.42 (*app.* s, 1H, Ar-*H*), 7.41 – 7.37 (m, 2H, 2 × Ar-*H*), 7.27 (app. d, *J* = 8.5 Hz, 2H, 2 × Ar-*H*), 7.16 – 7.09 (m, 6H, 6 × Ar-*H*), 6.71 (s, 1H, Het-*H*), 4.02 (s, 2H, Ar-*CH_2*-Ar), 2.36 (s, 3H, Ar-*CH_3*); **^{13}C NMR** (101 MHz, $CDCl_3$) δ 144.9, 143.3 (q, *J* = 38.0 Hz), 141.9, 139.9, 139.3, 138.1, 133.5, 132.4, 130.3, 129.7, 129.53, 129.49, 128.7, 126.3, 125.9, 121.4 (q,

J = 268.5 Hz), 118.9, 112.8, 105.4 (q, *J* = 2.0 Hz), 40.9, 21.4; **^{19}F NMR** (377 MHz, $CDCl_3$) δ -62.16; **IR** (neat)/v_{max} 2910, 2230, 1473, 1235, 1160, 978; **HRMS (ESI)** calculated for $C_{25}H_{19}N_3F_3$, $[M+H]^+$ is 418.1526, found 418.1523.

8.0 ^{1}H, ^{13}C and ^{19}F NMR Spectra

8.1 Starting material spectra

Sodium ((3,4,5-trimethoxybenzoyl)oxy)methanesulfinate (Rongacyl) (S1)

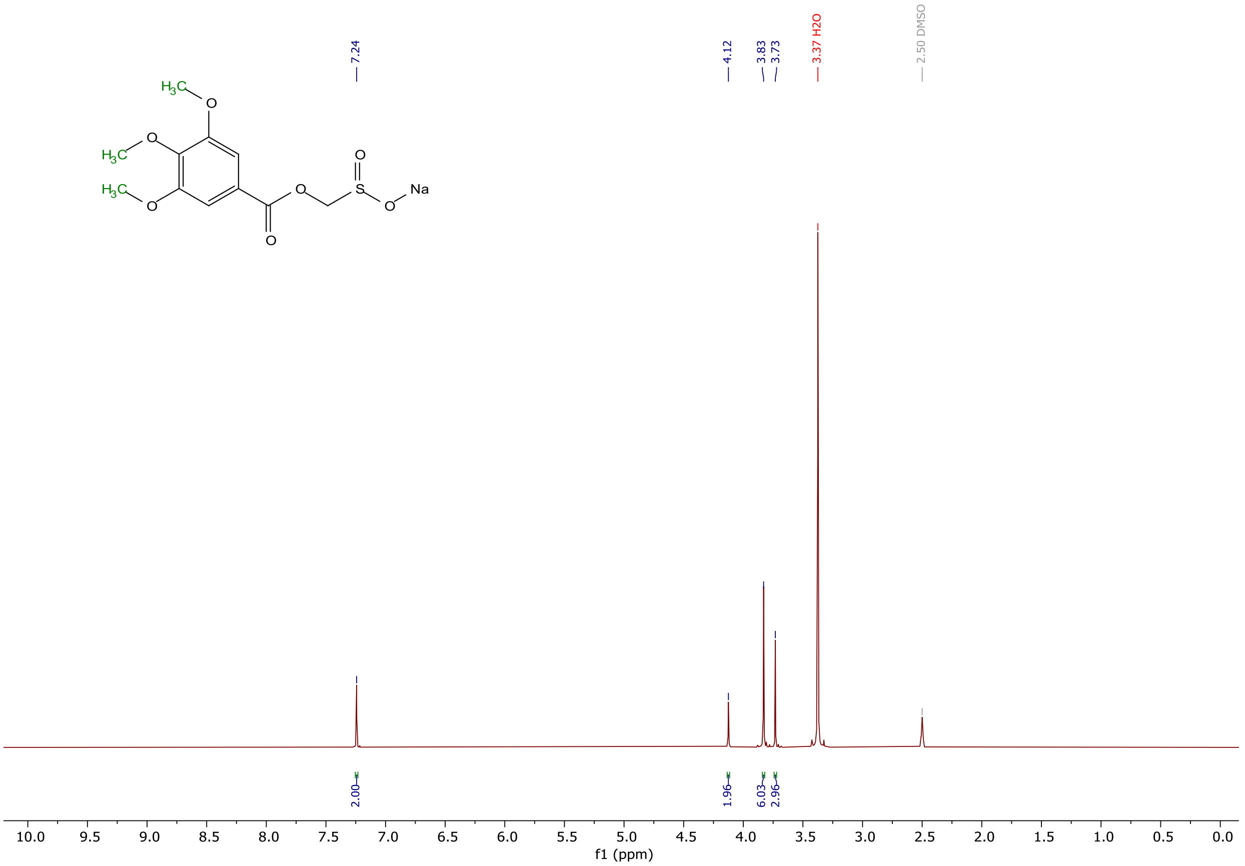

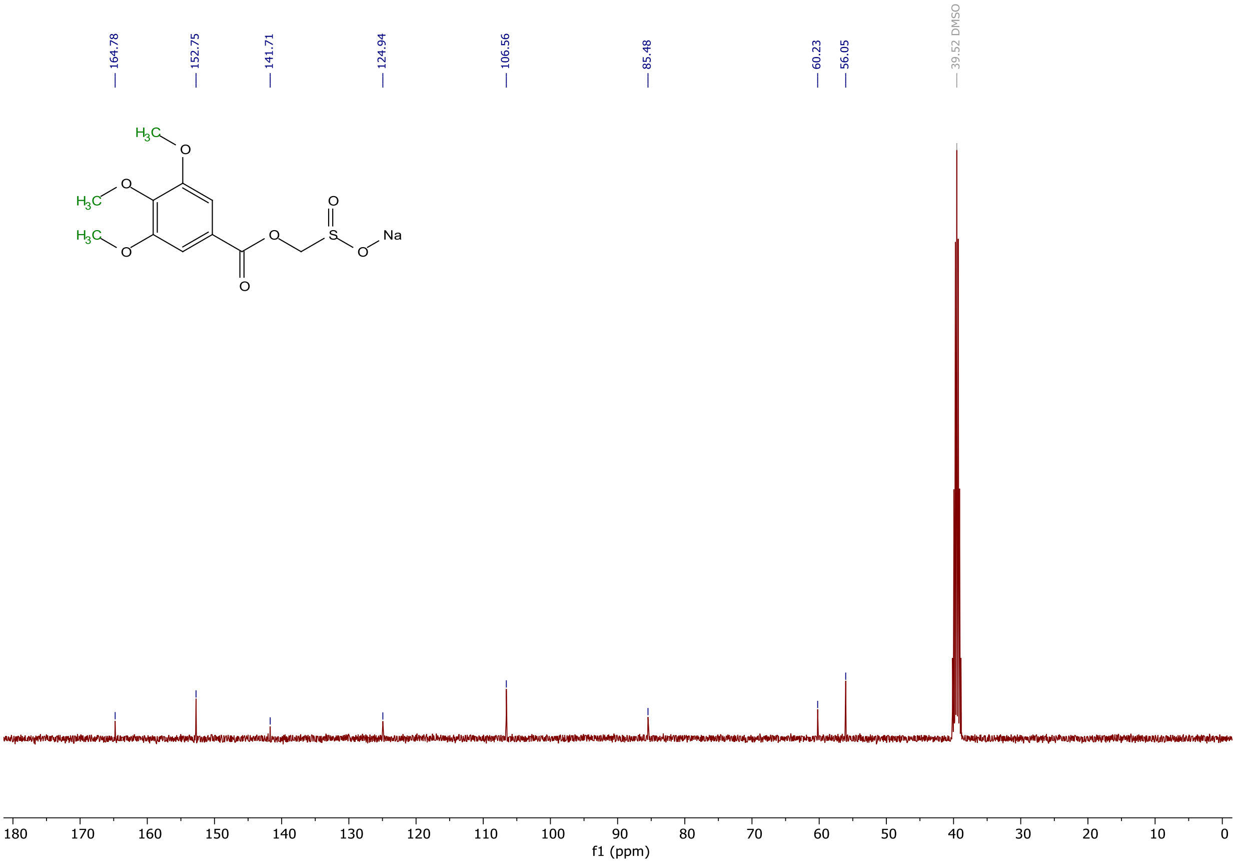

Sodium 2-cyanoethane-1-sulfinate (βECN sulfinate) (S2)

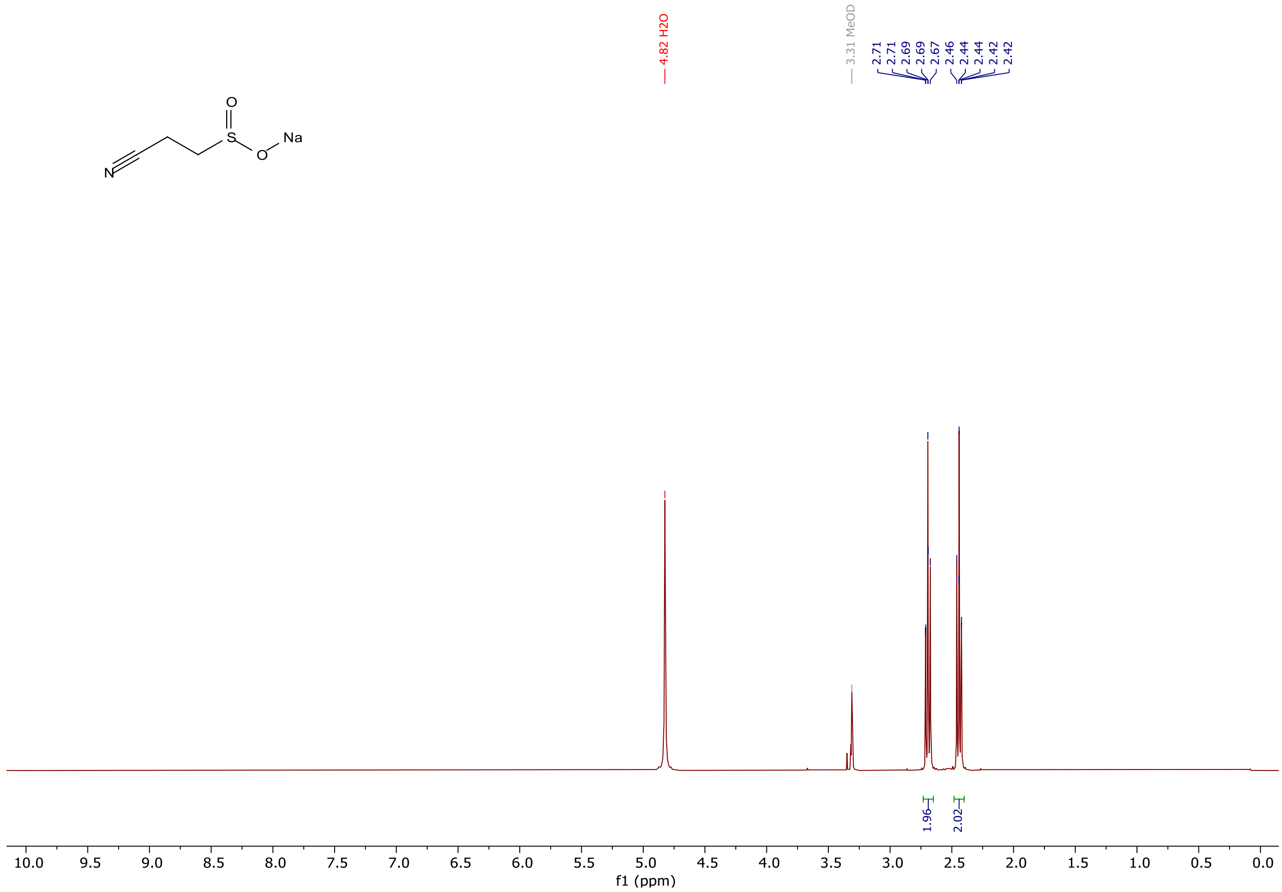

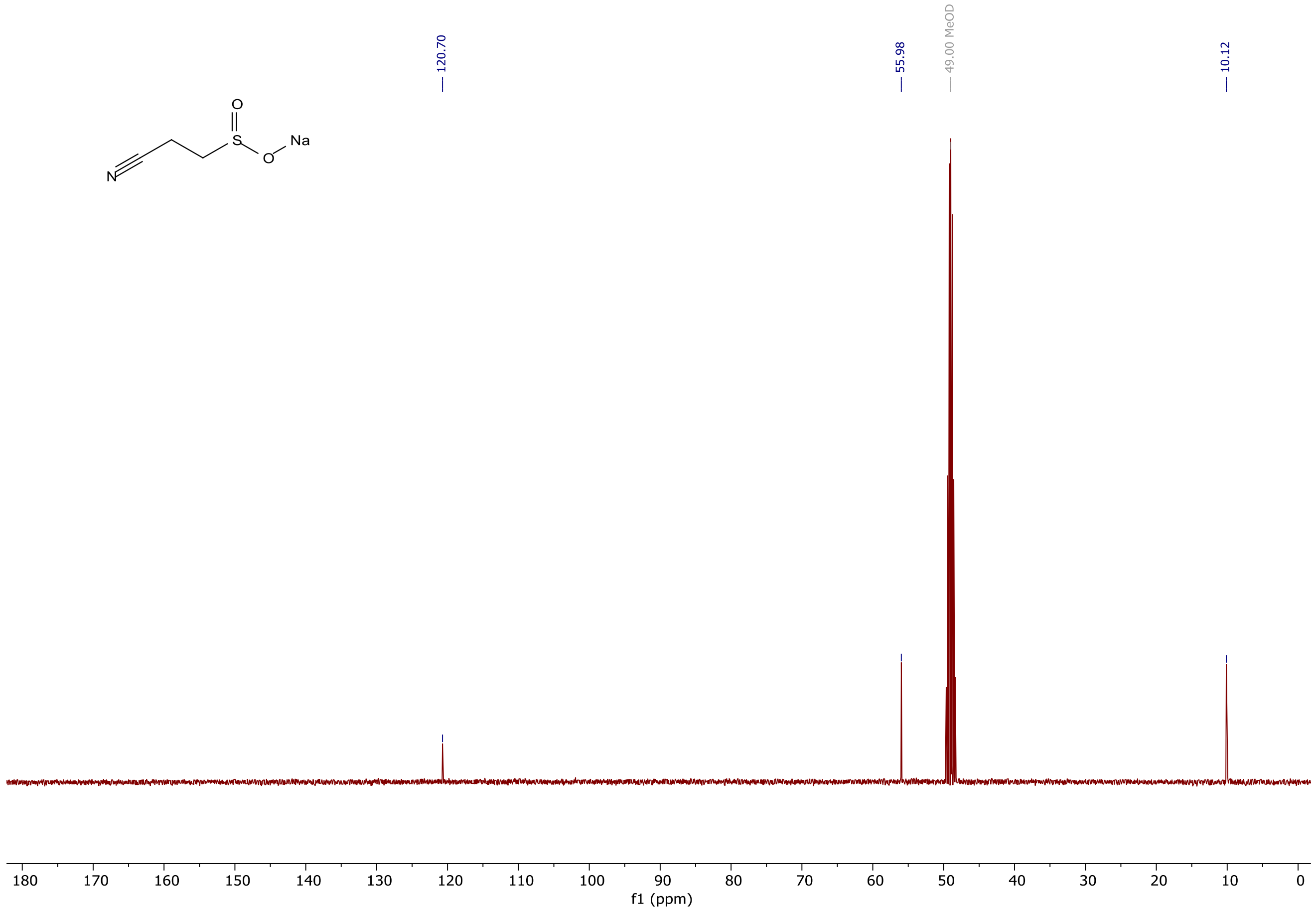

3-(Benzylsulfonyl)propanenitrile (3a)

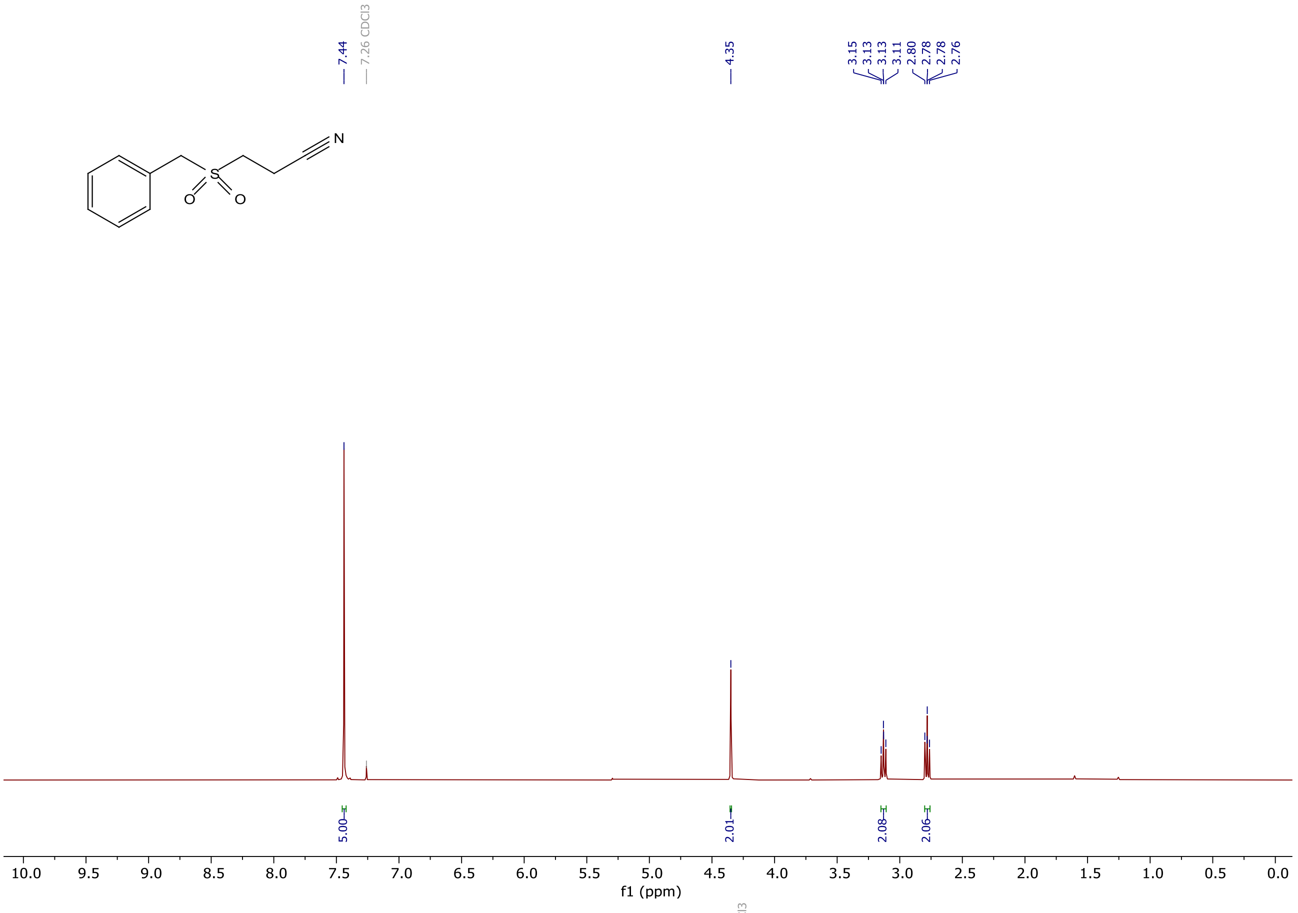

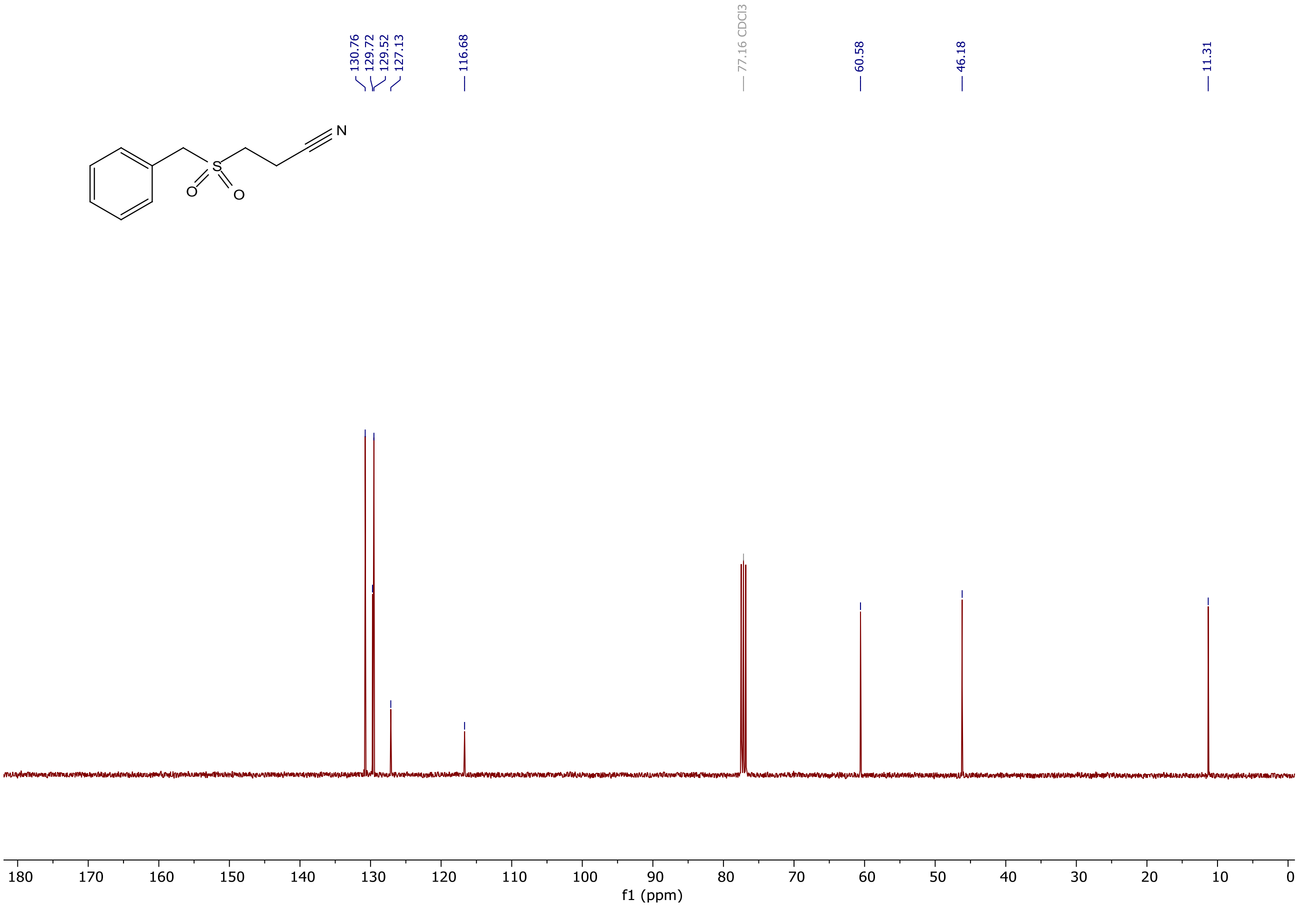

3-((4-(Trifluoromethyl)benzyl)sulfonyl)propanenitrile (3b)

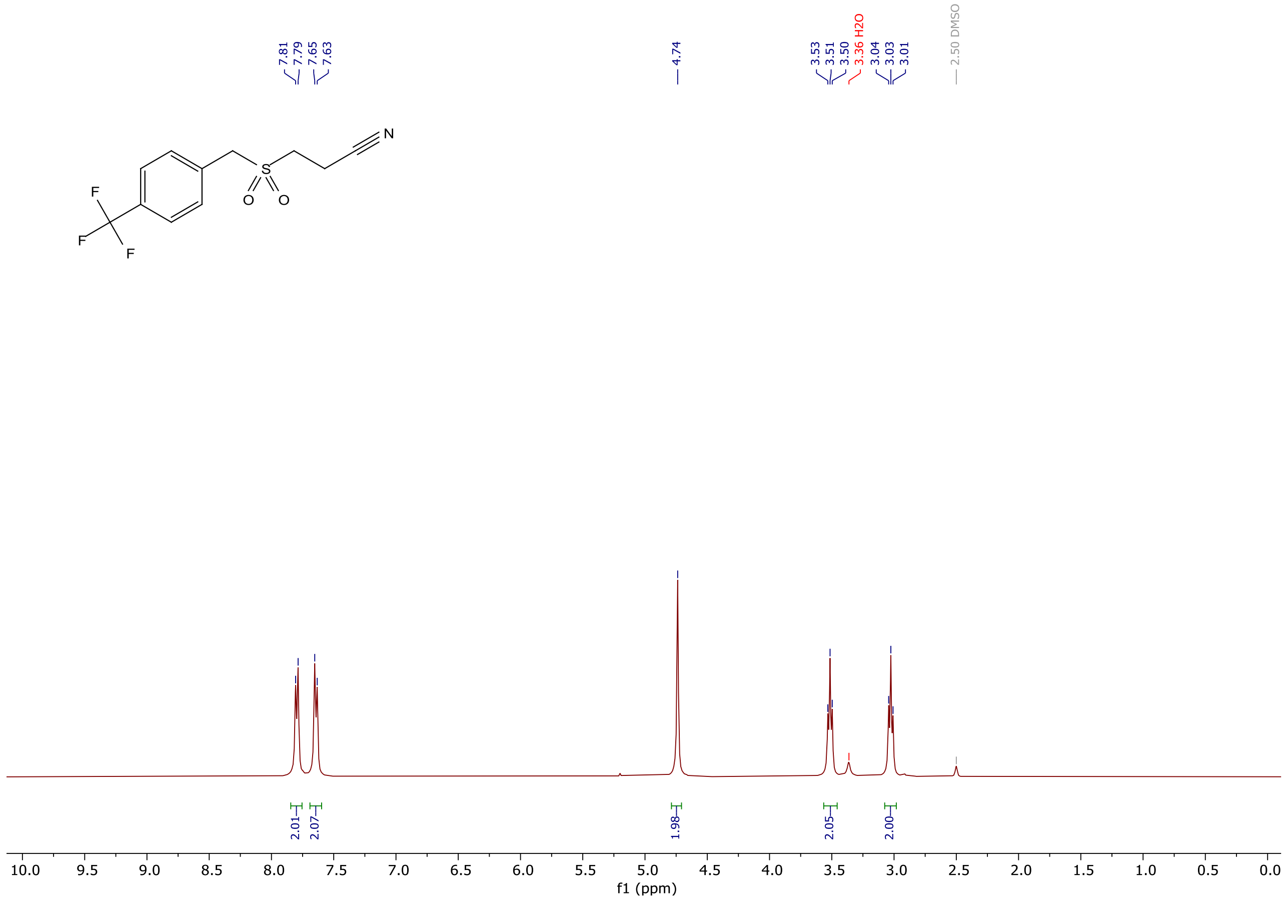
7.81
7.79
7.65
7.63
4.74
3.53
3.51
3.50
3.36 H2O
3.04
3.03
3.01
2.50 DMSO
N
S
O
O
F
F
F
2.01
2.07
1.98
2.05
2.00
10.0
9.5
9.0
8.5
8.0
7.5
7.0
6.5
6.0
5.5
5.0
4.5
4.0
3.5
3.0
2.5
2.0
1.5
1.0
0.5
0.0
f1 (ppm)

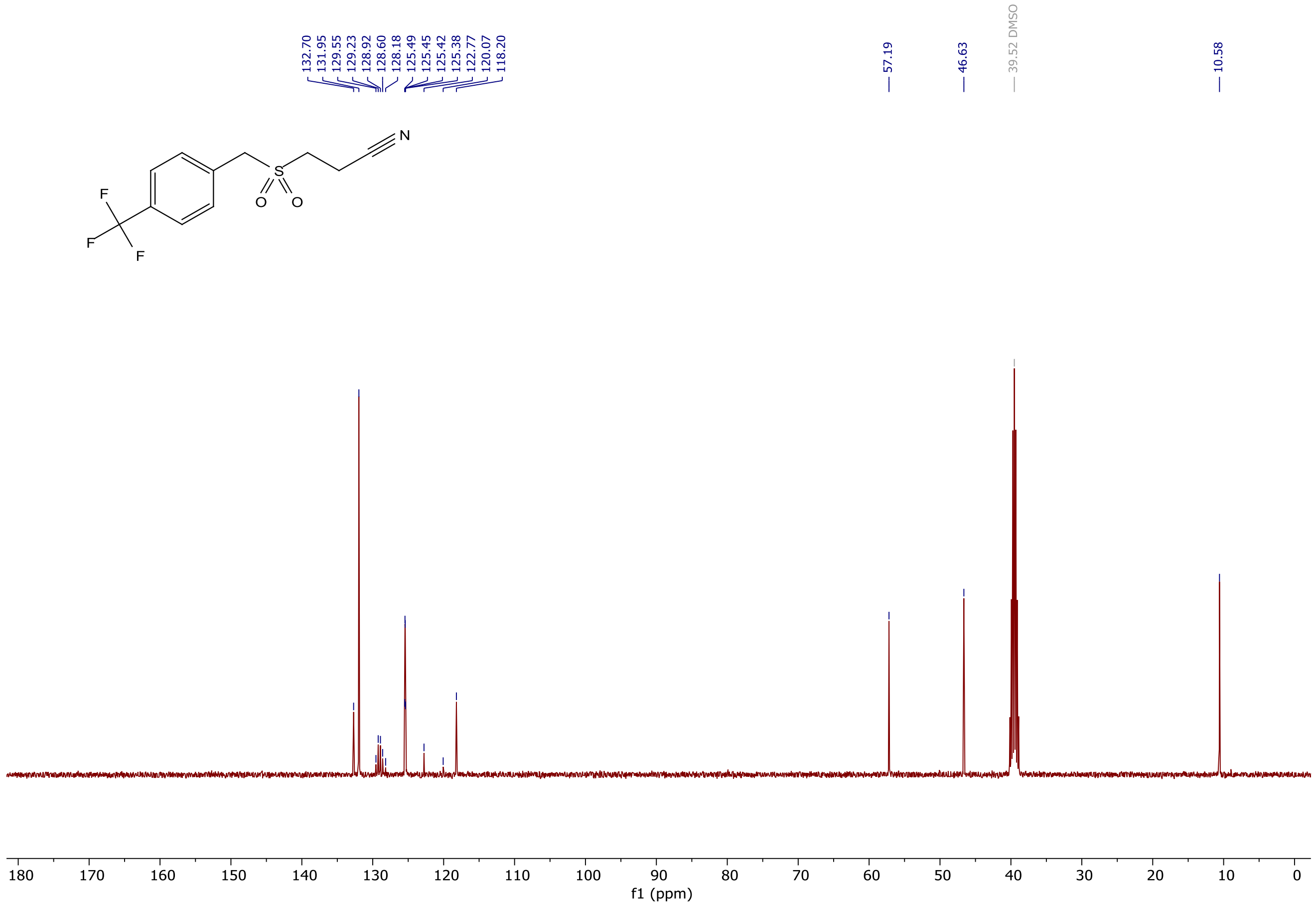
132.70
131.95
129.55
129.23
128.92
128.60
128.18
125.49
125.45
125.42
125.38
122.77
120.07
118.20
57.19
46.63
39.52 DMSO
10.58
N
S
O
O
F
F
F
180
170
160
150
140
130
120
110
100
90
80
70
60
50
40
30
20
10
0
f1 (ppm)

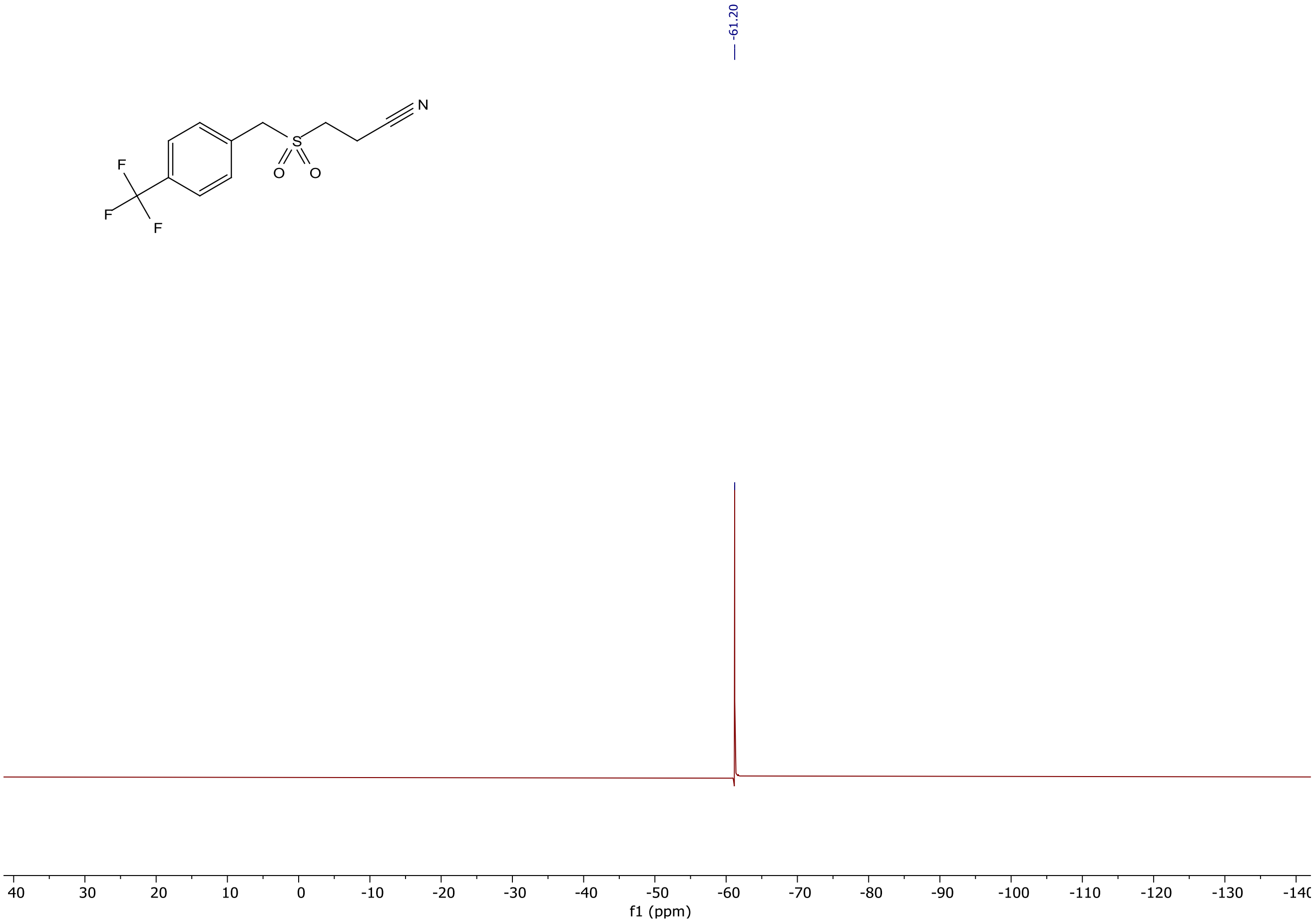
-61.20
F
F
F
S
O
O
N
40
30
20
10
0
-10
-20
-30
-40
-50
-60
-70
-80
-90
-100
-110
-120
-130
-140
f1 (ppm)

((4-Methoxybenzyl)sulfonyl)methyl 3,4,5-trimethoxybenzoate (3c)

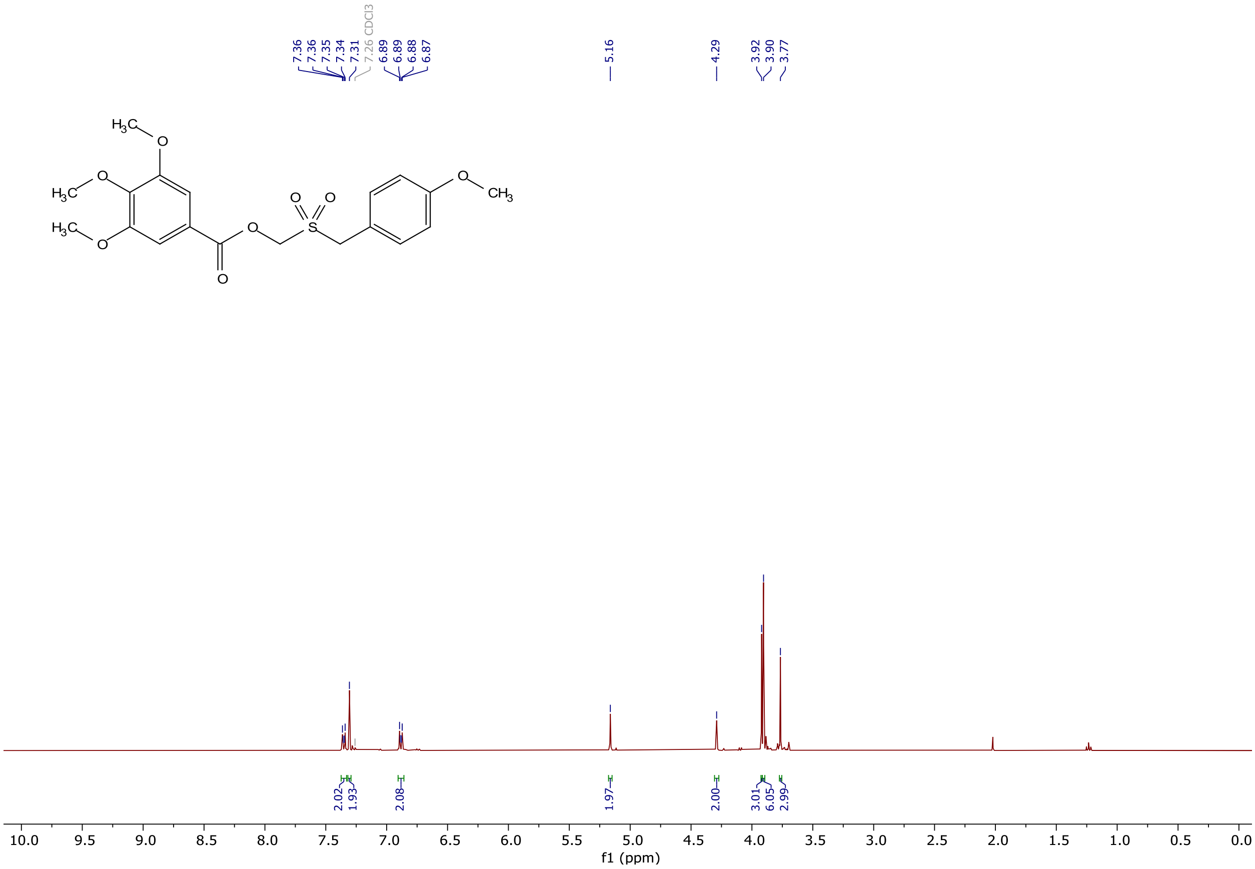

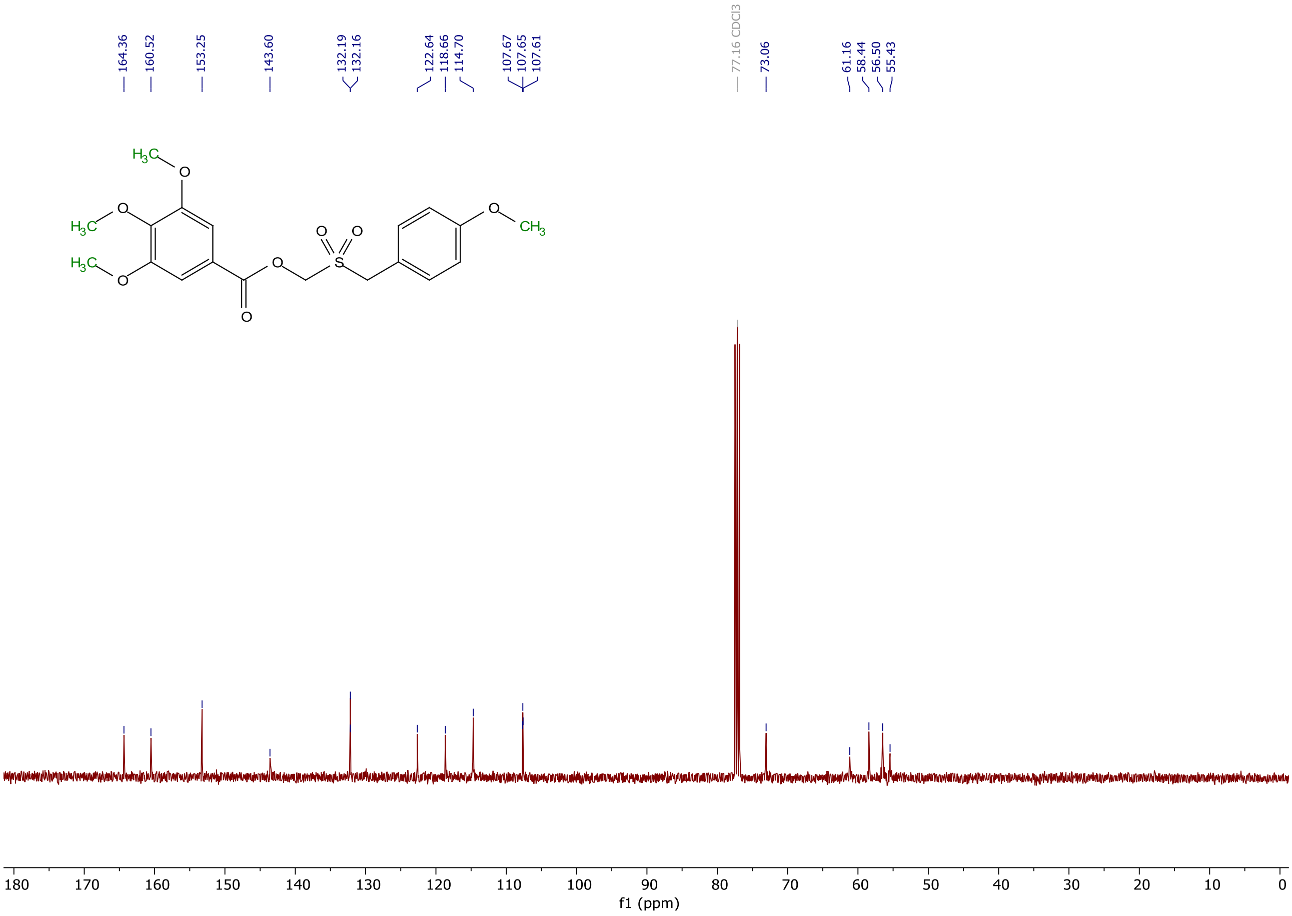

3-(((2-Cyanoethyl)sulfonyl)methyl)benzonitrile (3d)

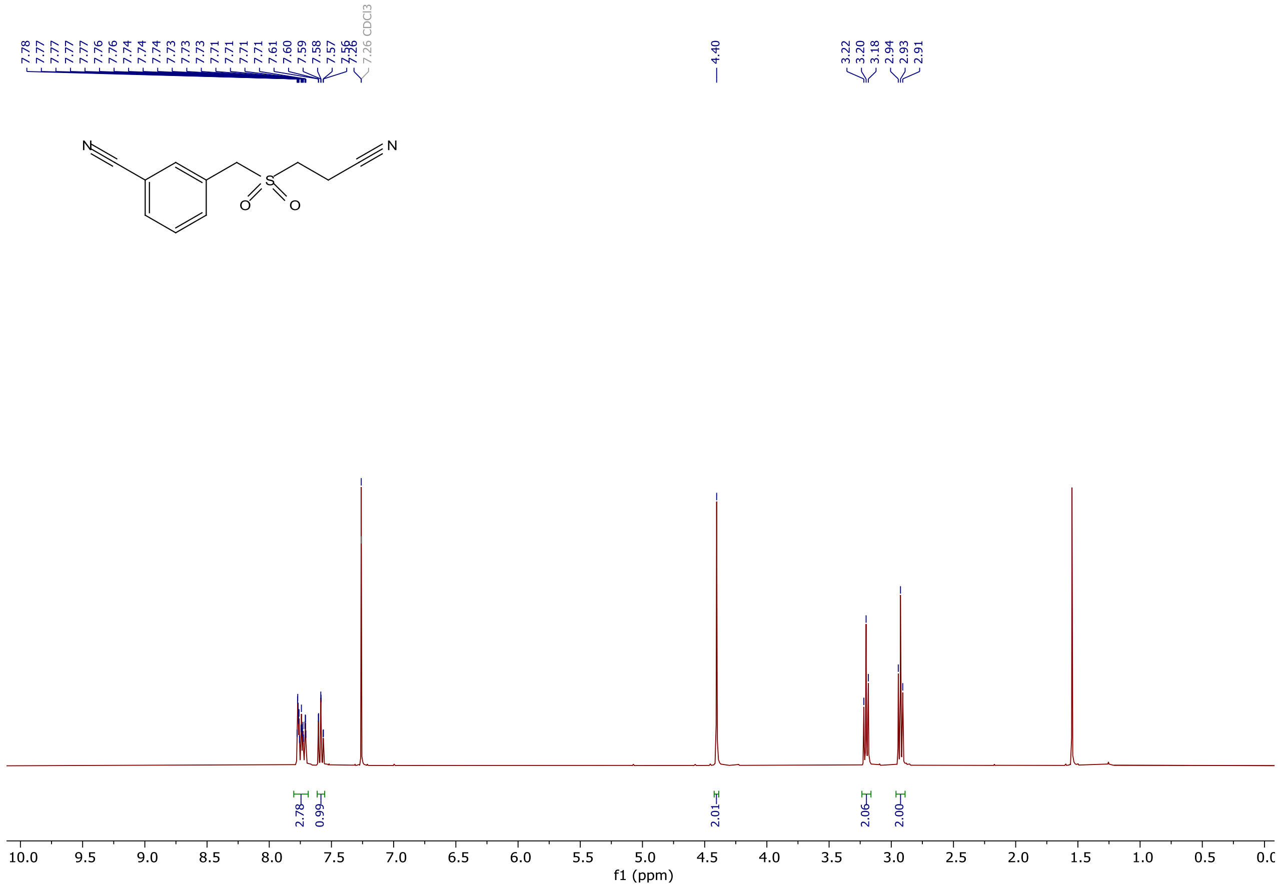

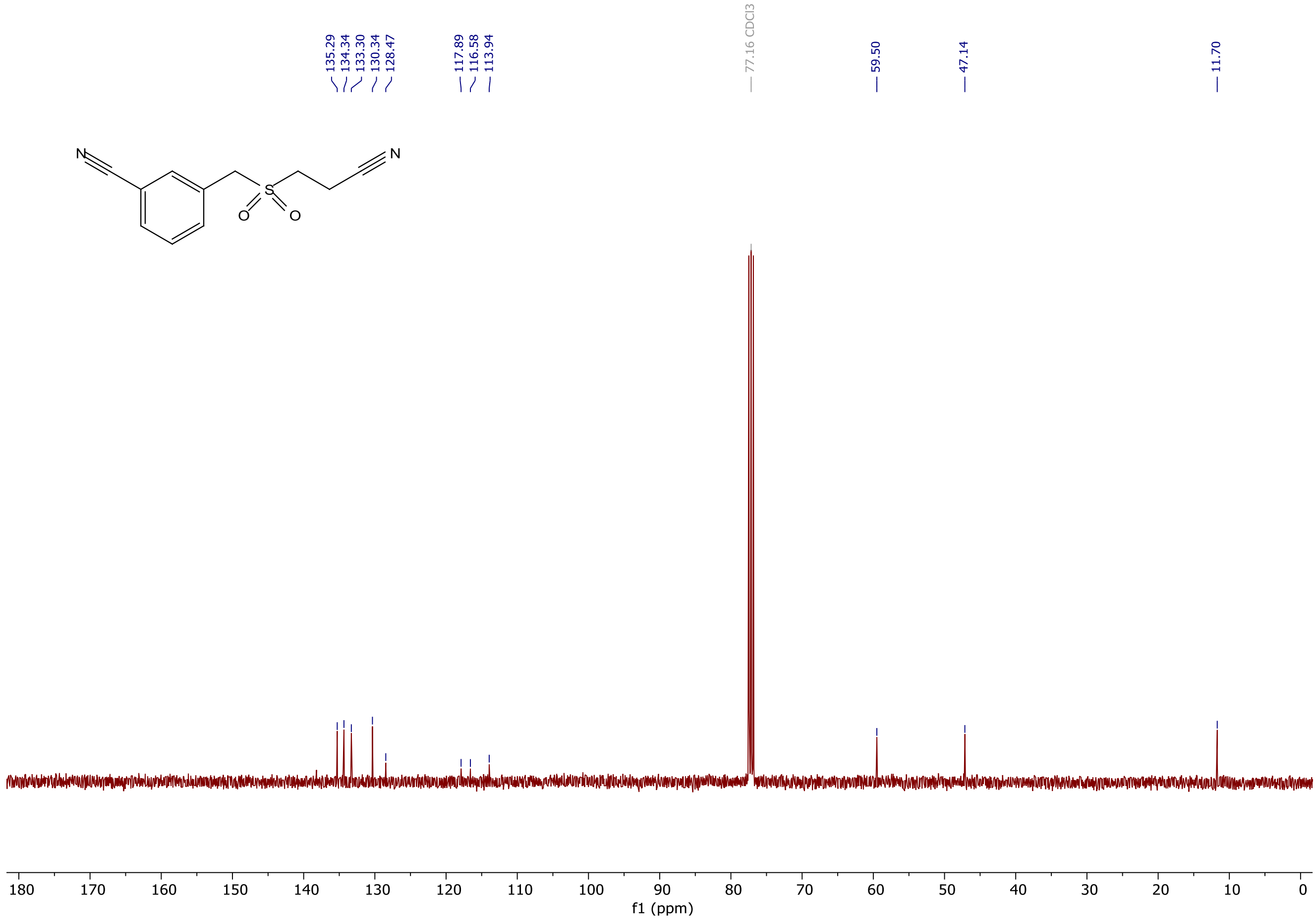

Sodium benzylsulfinate (4a)

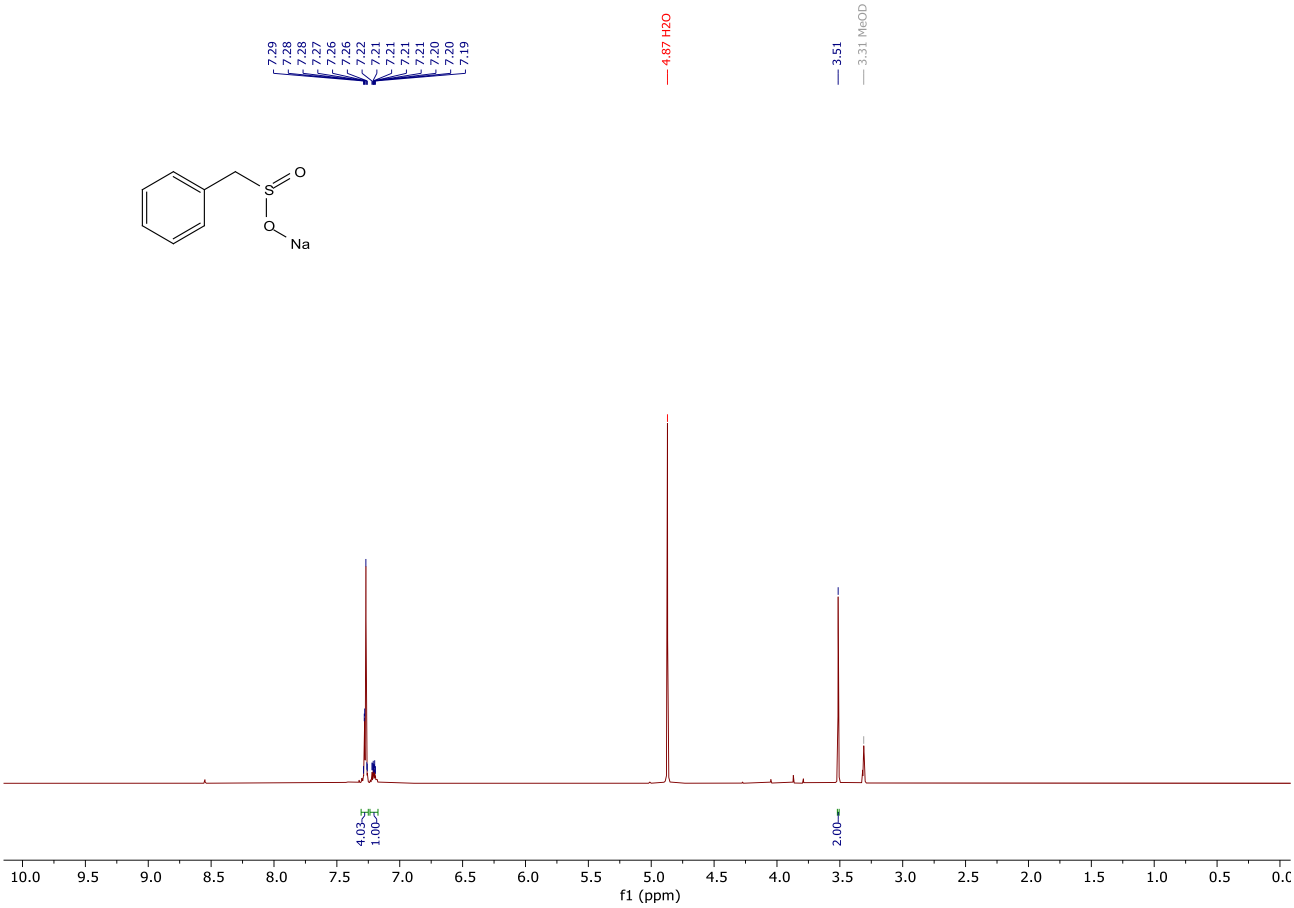

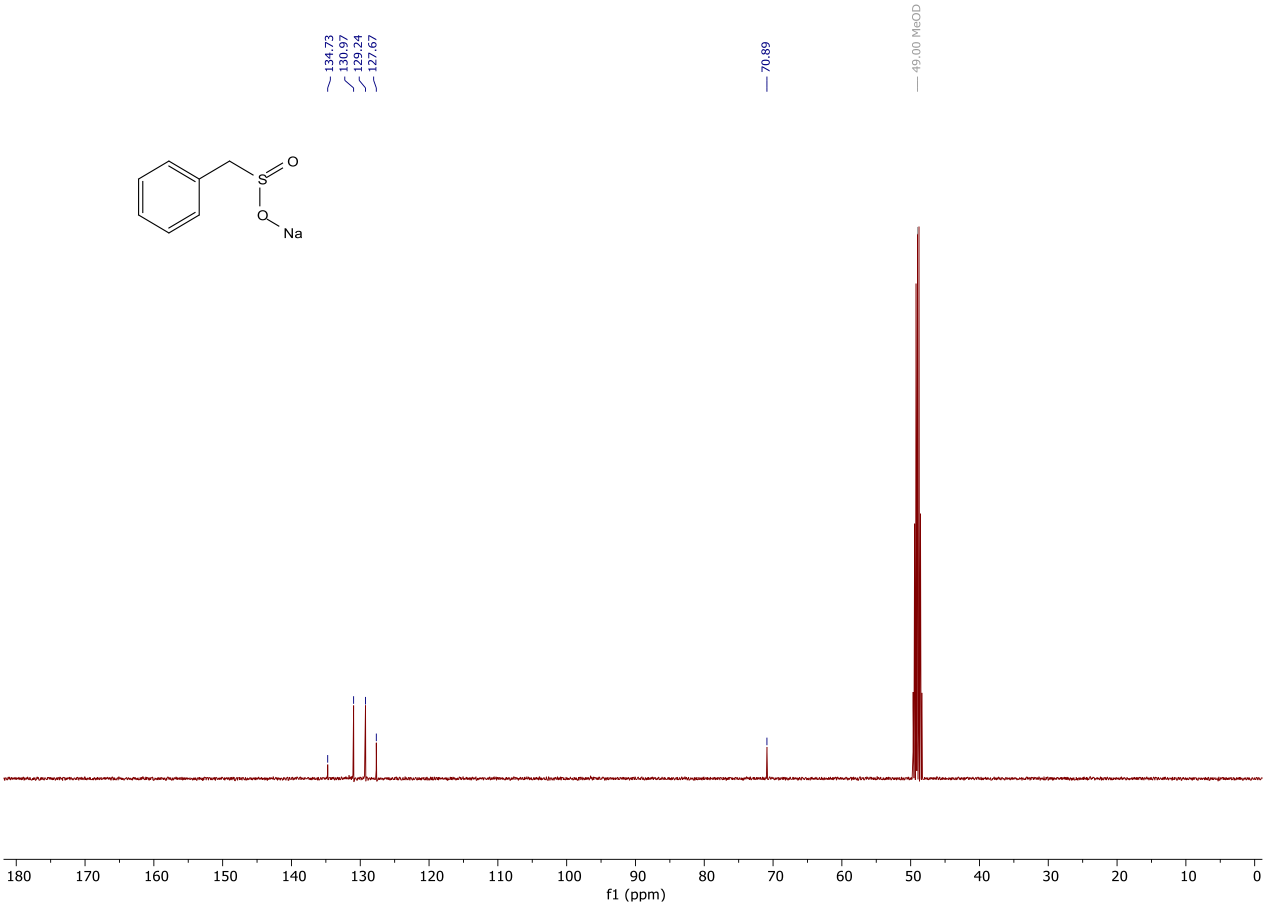

Sodium 4-(trifluoromethyl)benzylsulfinate (4b)

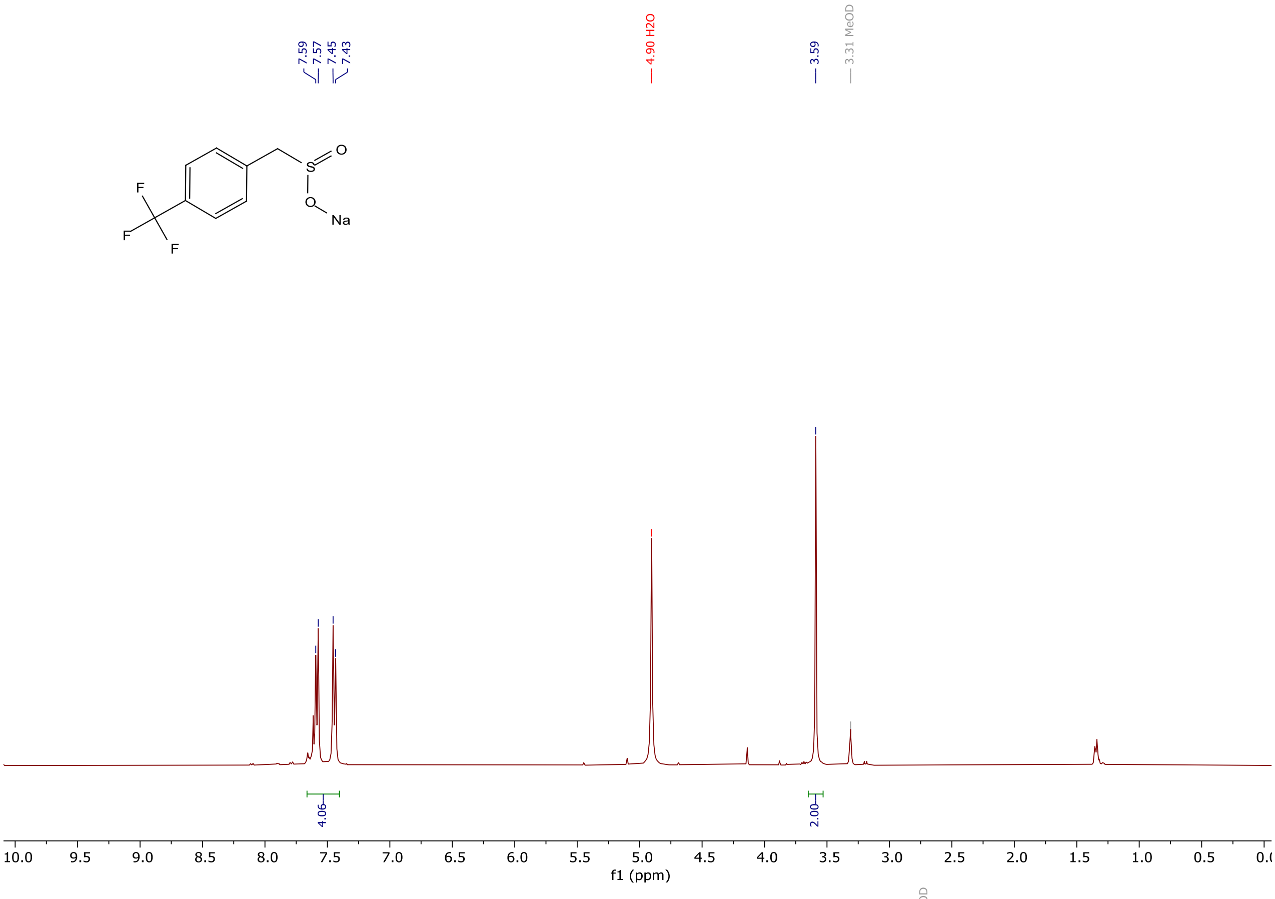

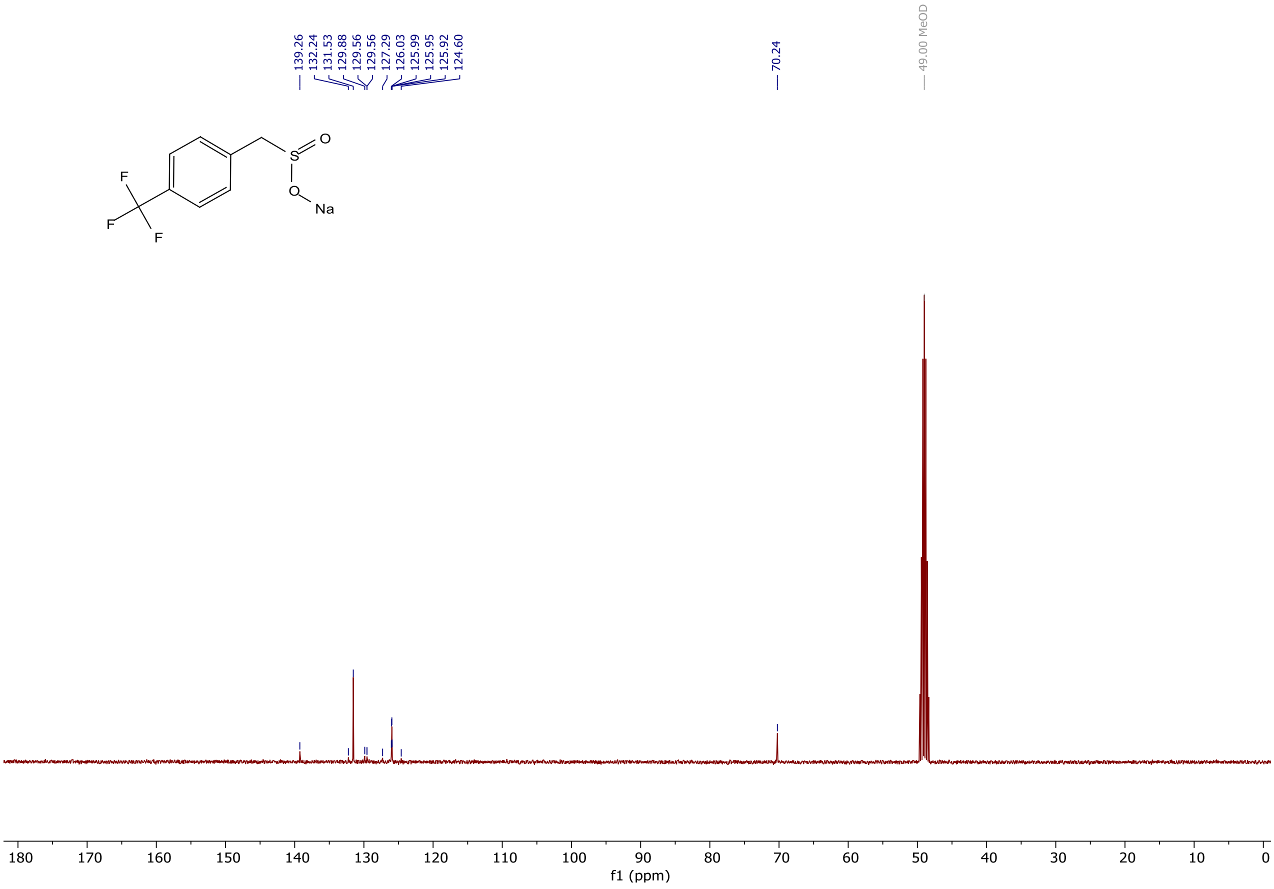

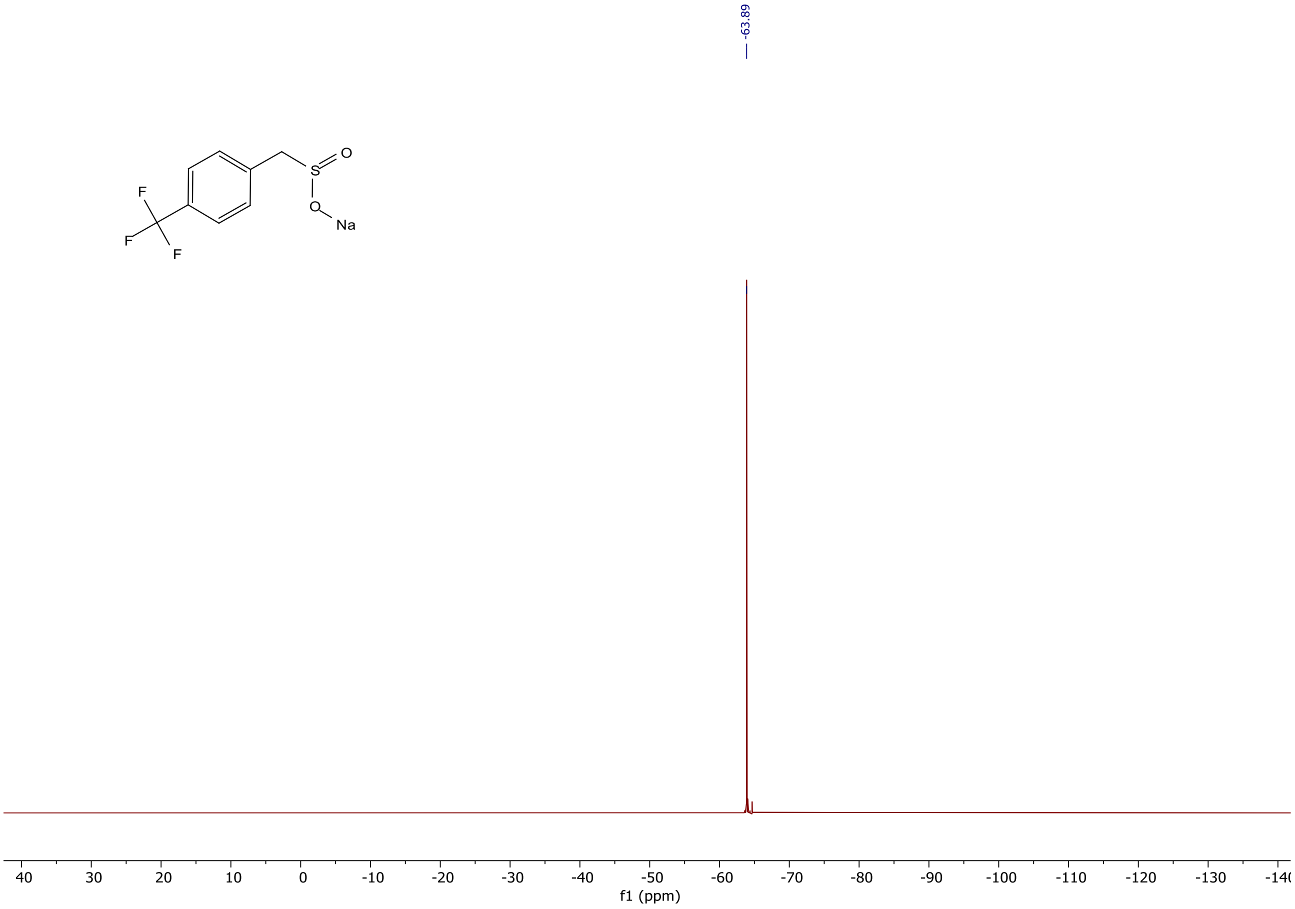
-63.89
O
S
O
Na
F
F
F
40 30 20 10 0 -10 -20 -30 -40 -50 -60 -70 -80 -90 -100 -110 -120 -130 -140
f1 (ppm)

Sodium (4-methoxy)benzylsulfinate (4c)

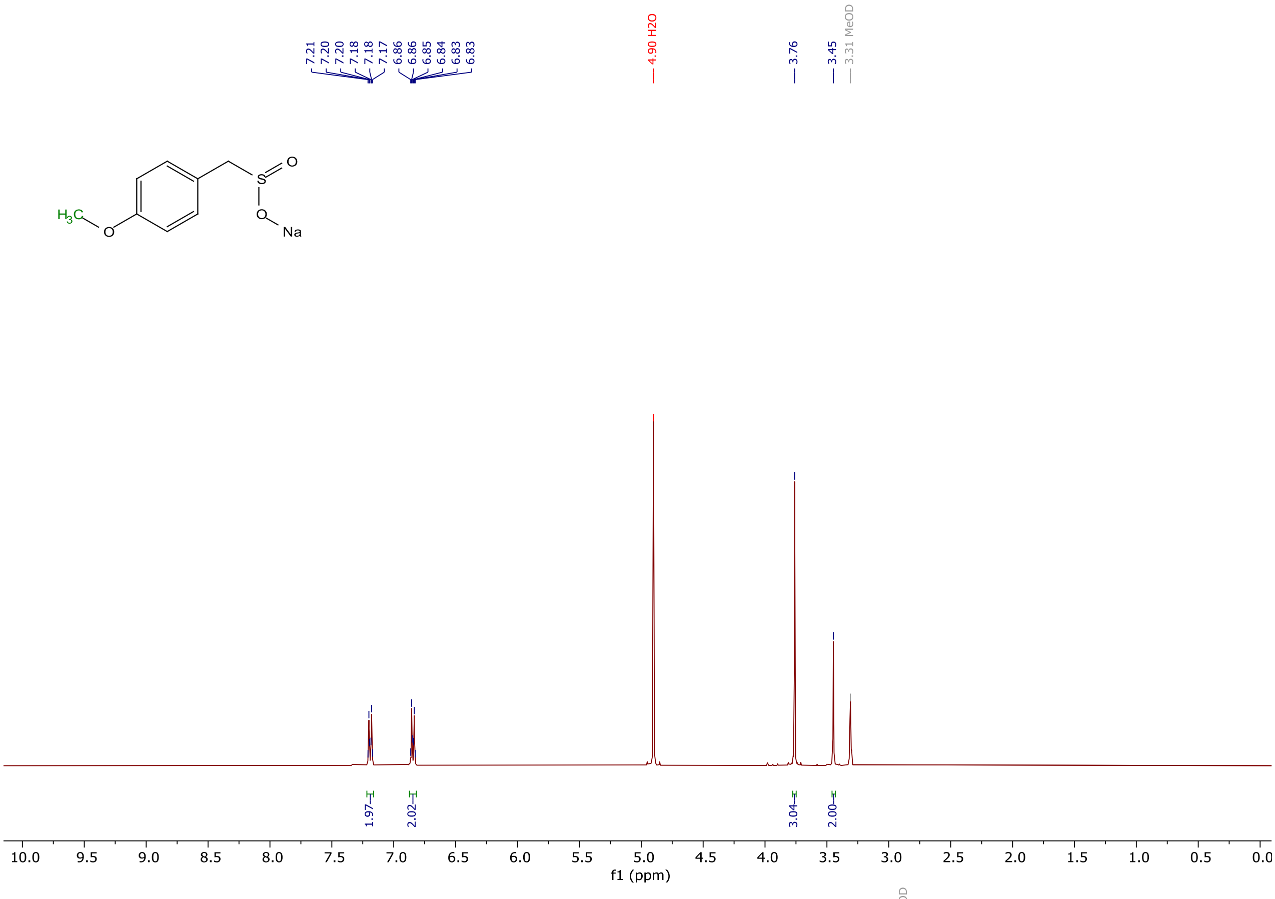

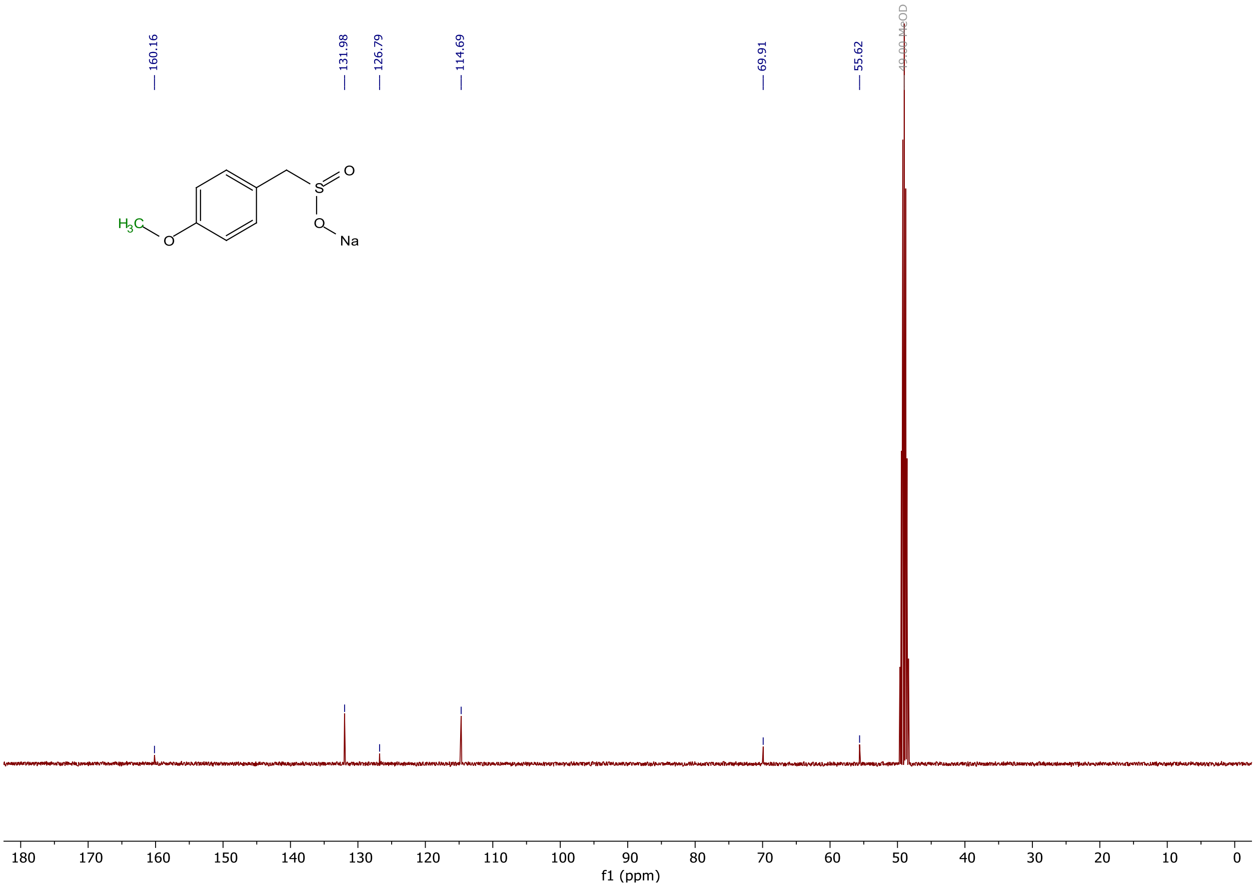

Sodium (3-cyanophenyl)methanesulfinate (4d)

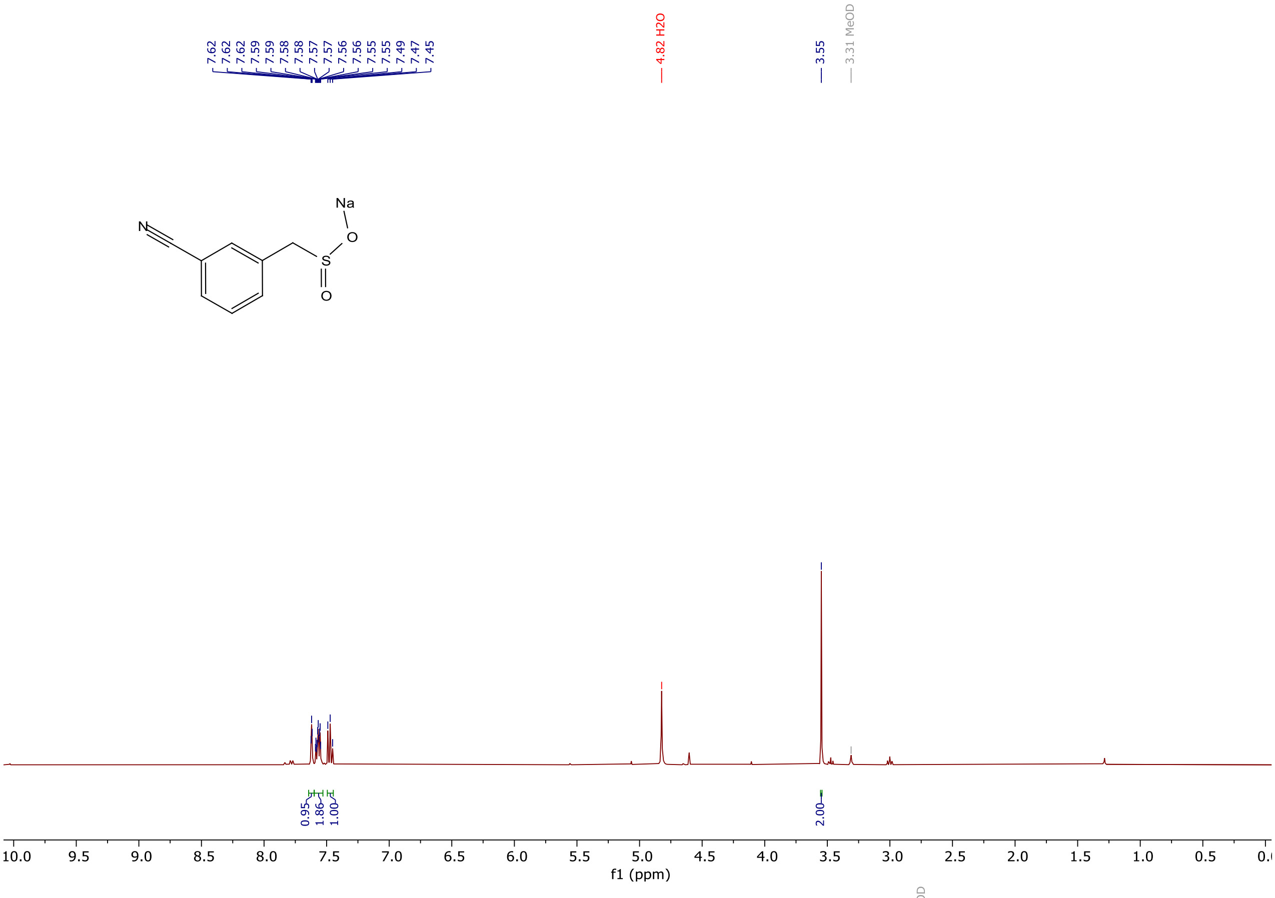

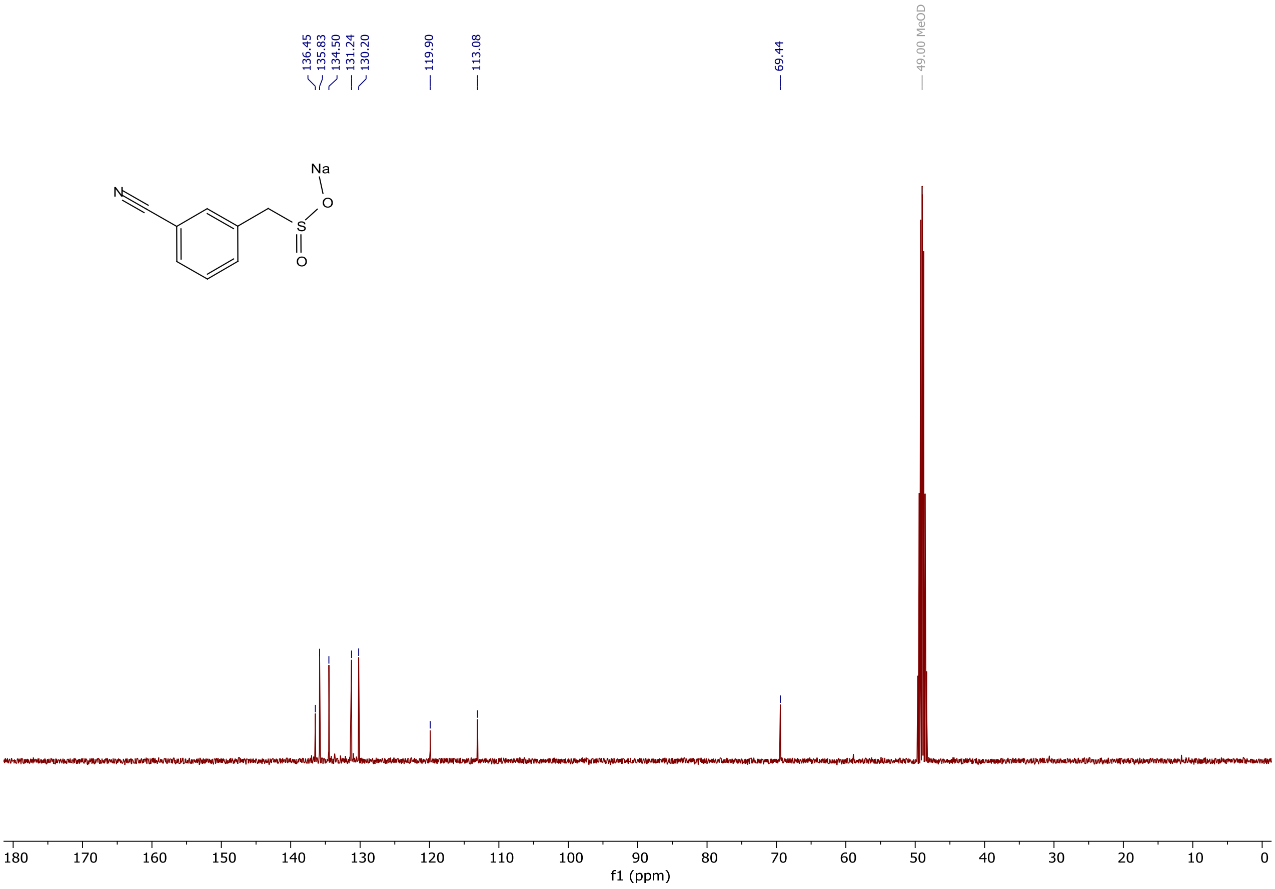

Benzyl 4-methylbenzenesulfonate (S3)

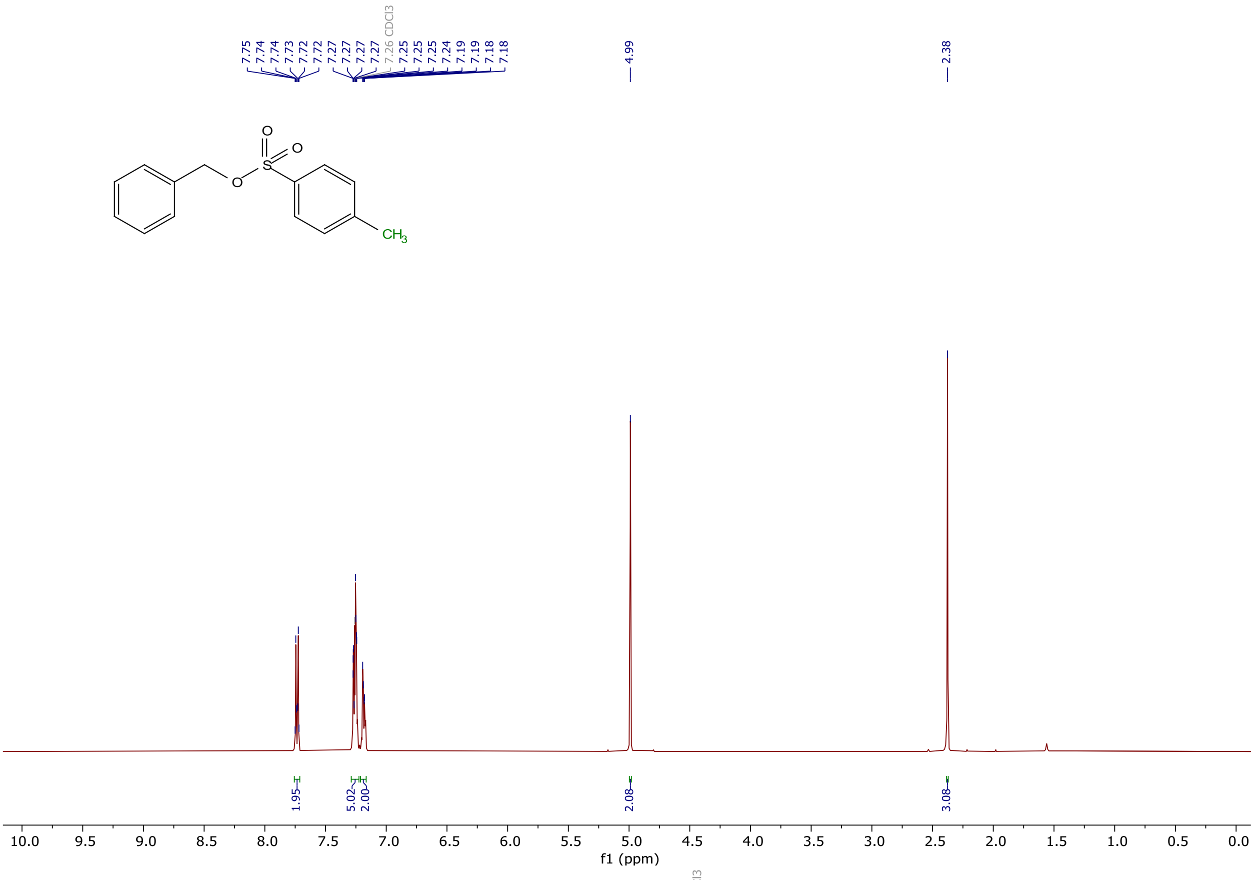

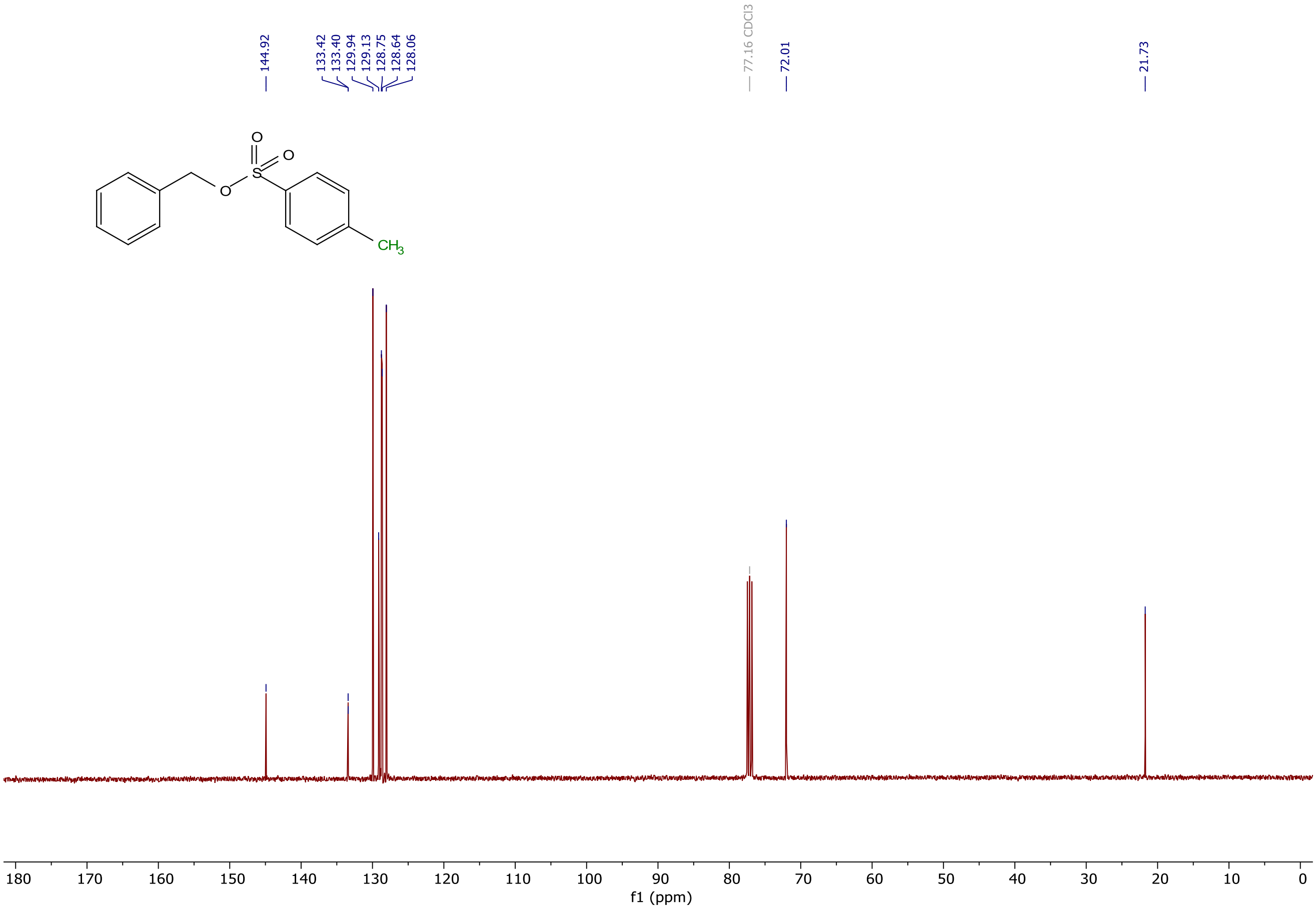

3-Methoxybenzyl 4-methylbenzenesulfonate (S4)

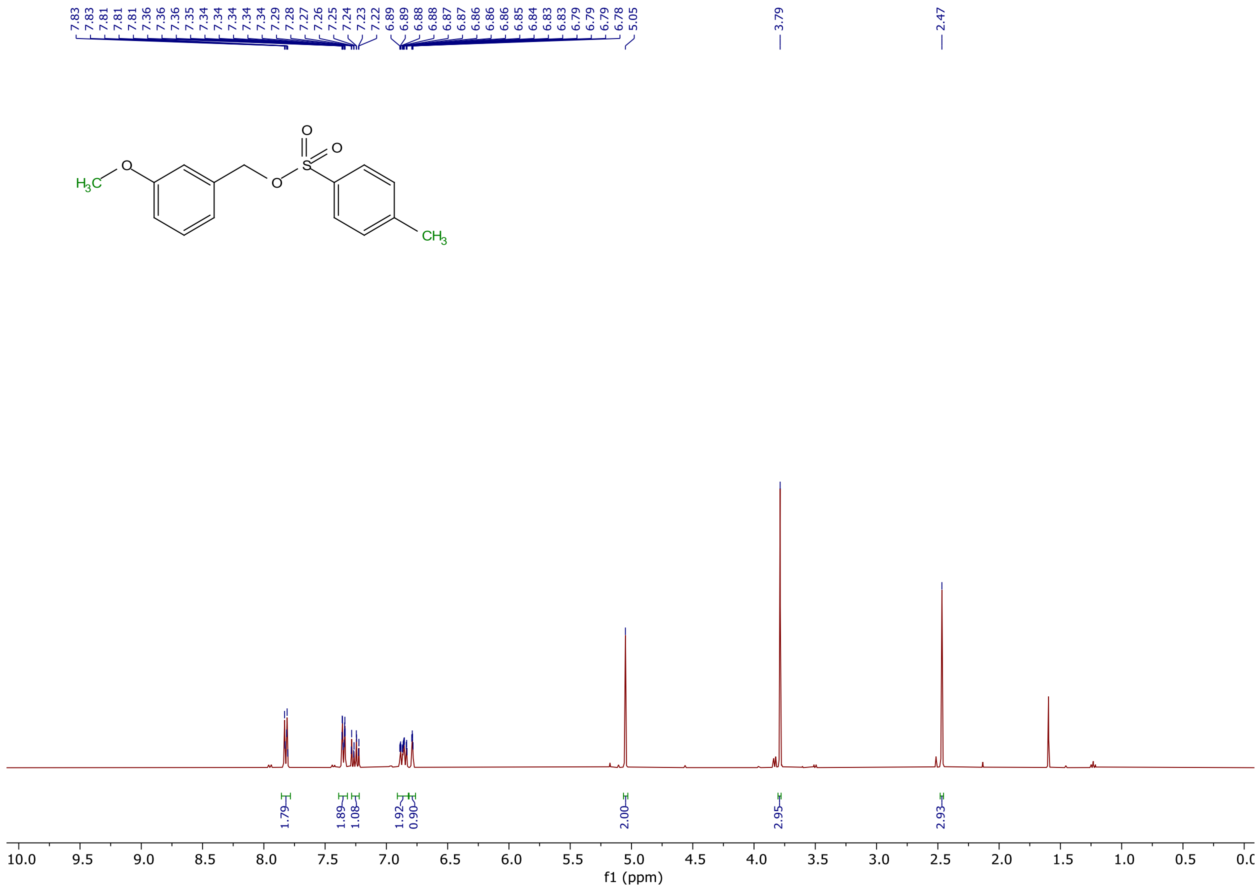

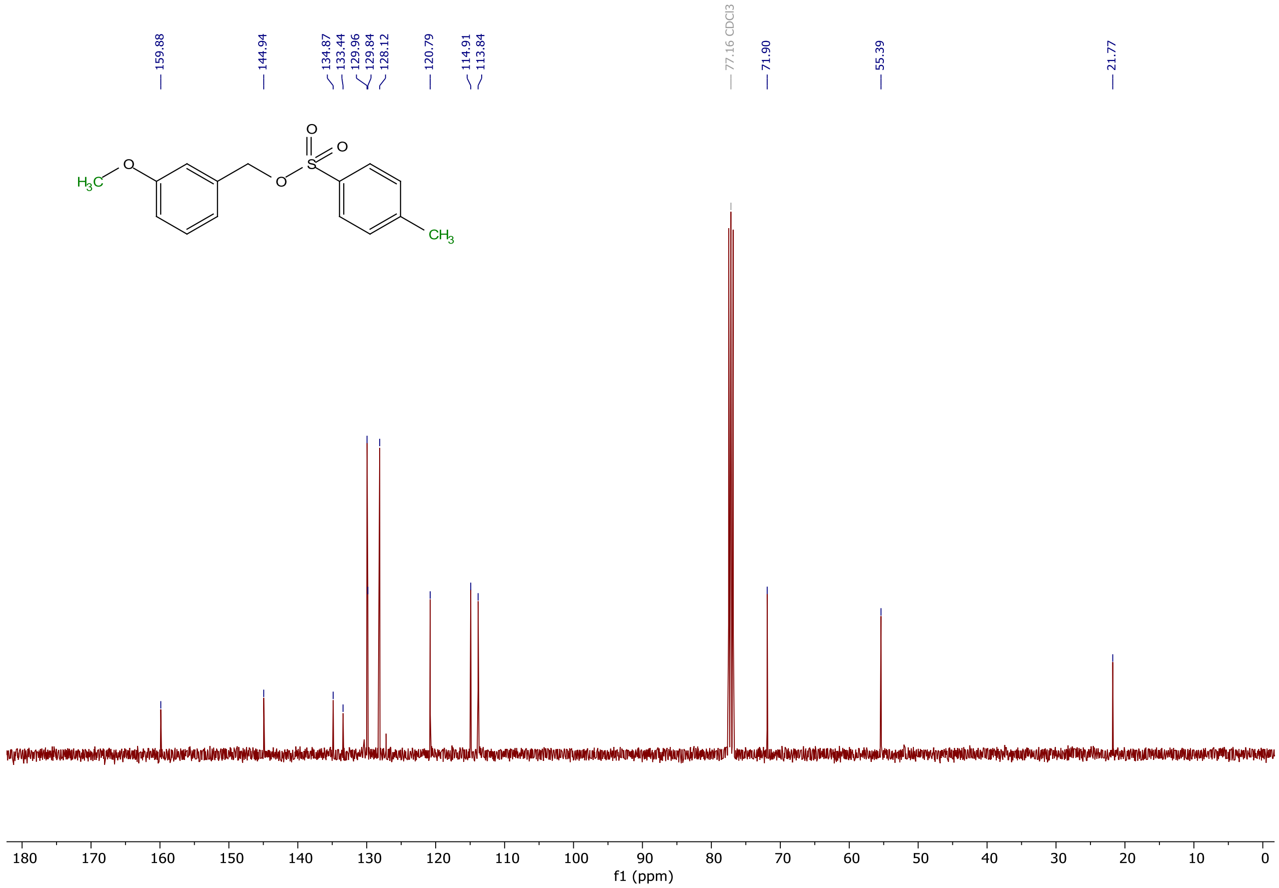

8.2 Di(hetero)arylmethane spectra

3-Benzylquinoline (5a)

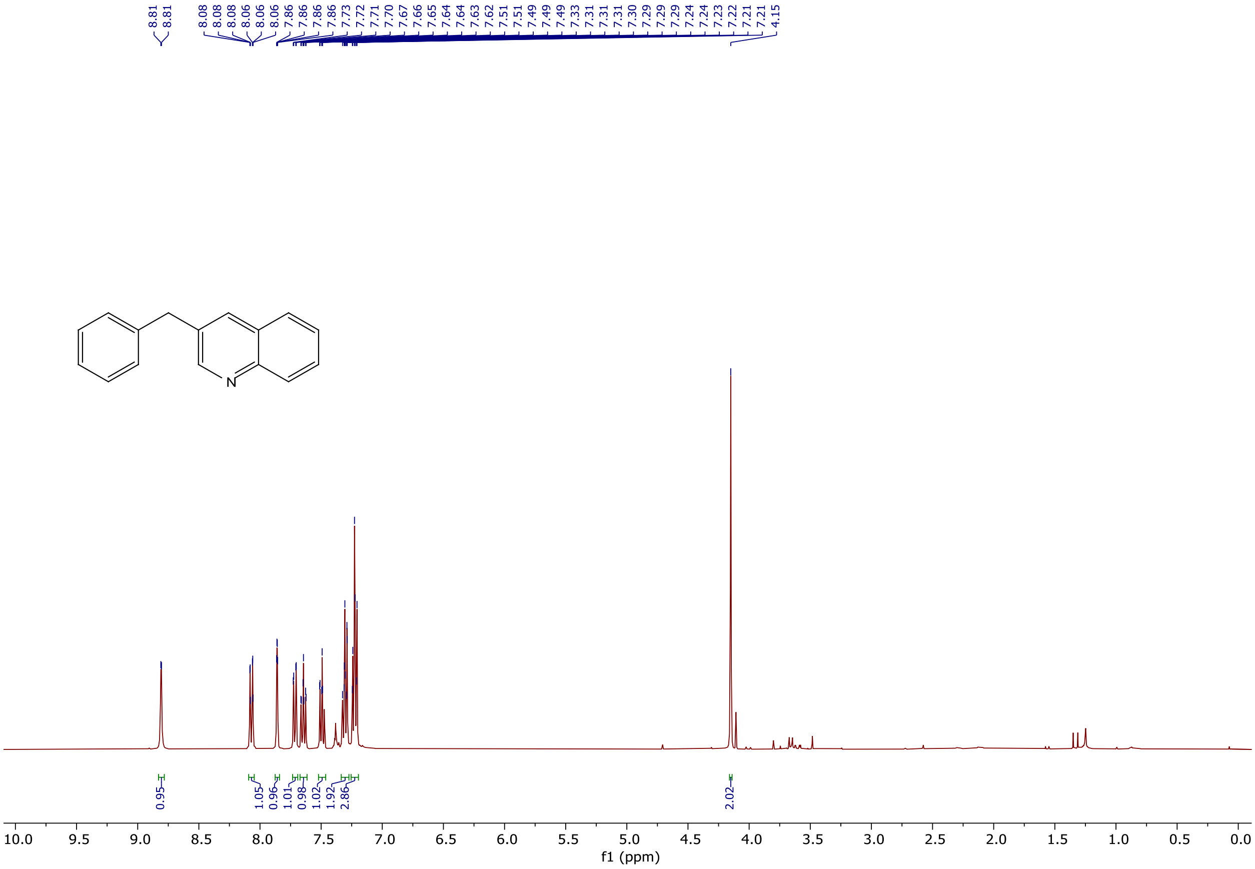

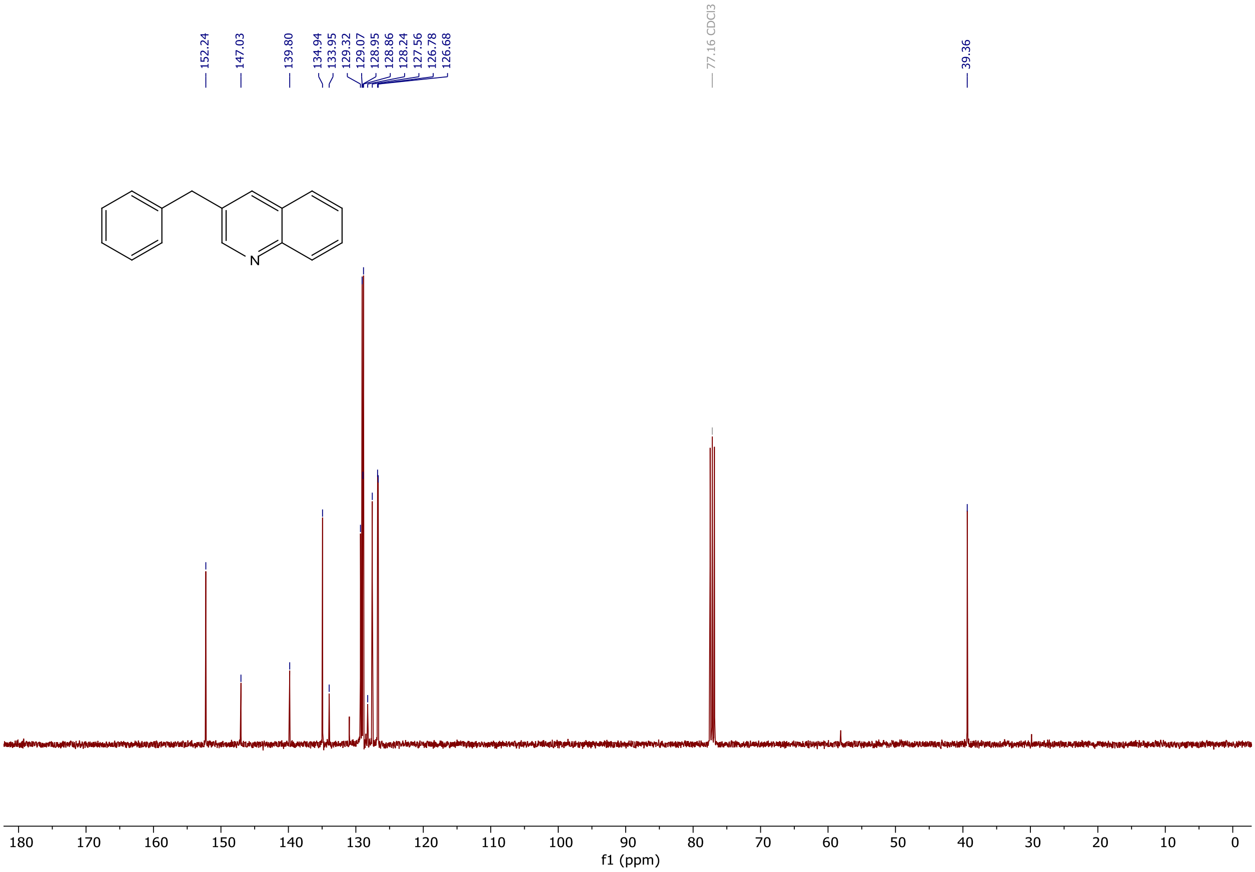

3-(2-Methylbenzyl)quinoline (5b)

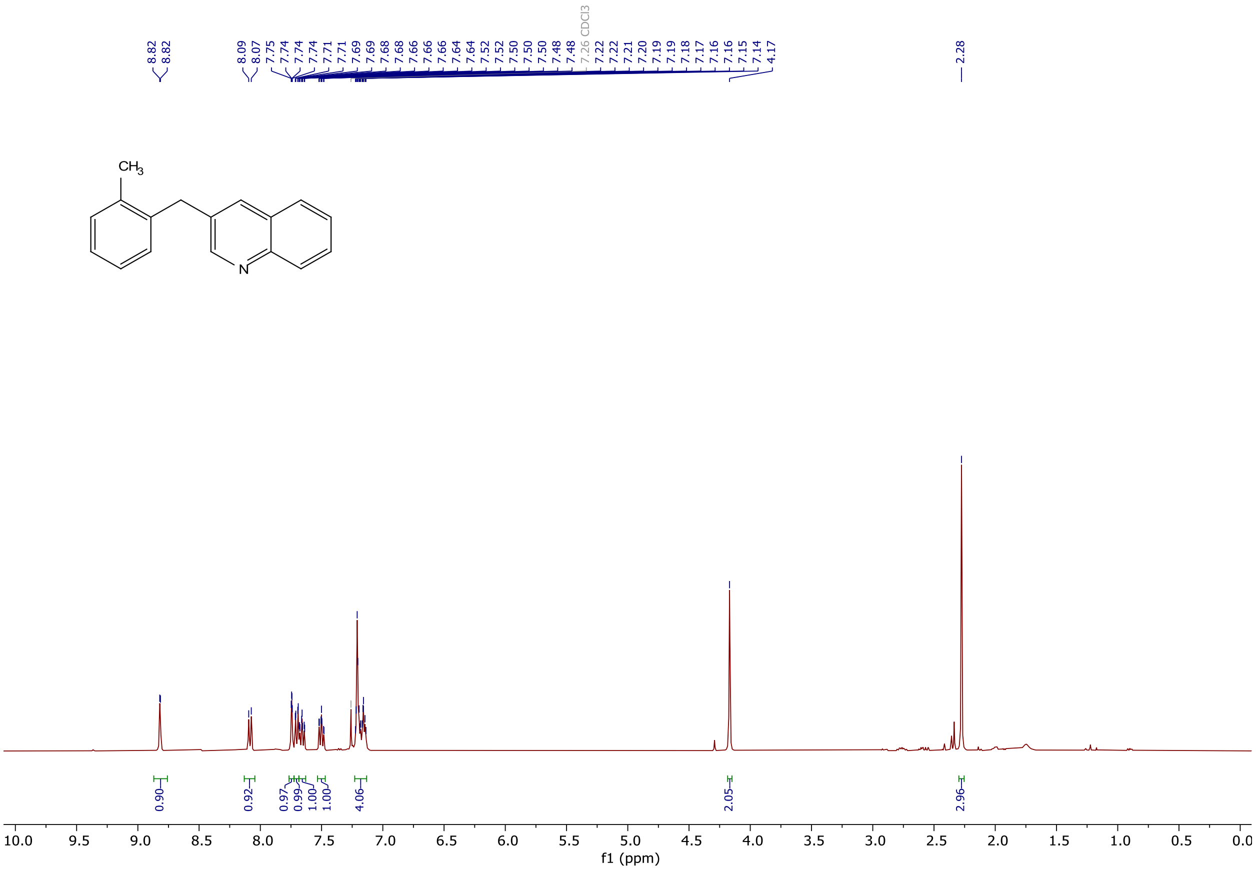

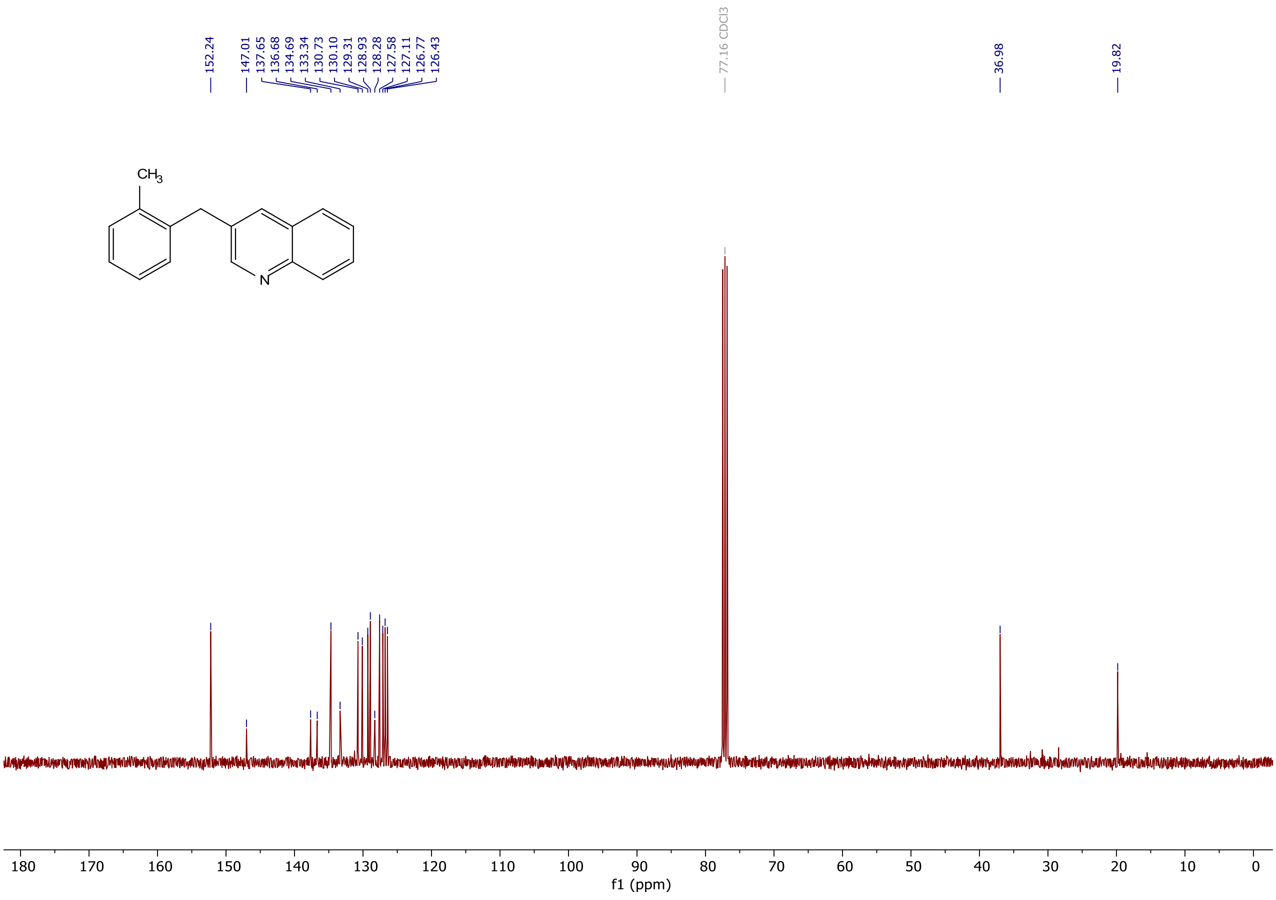

3-(3-Methoxybenzyl)quinoline (5c)

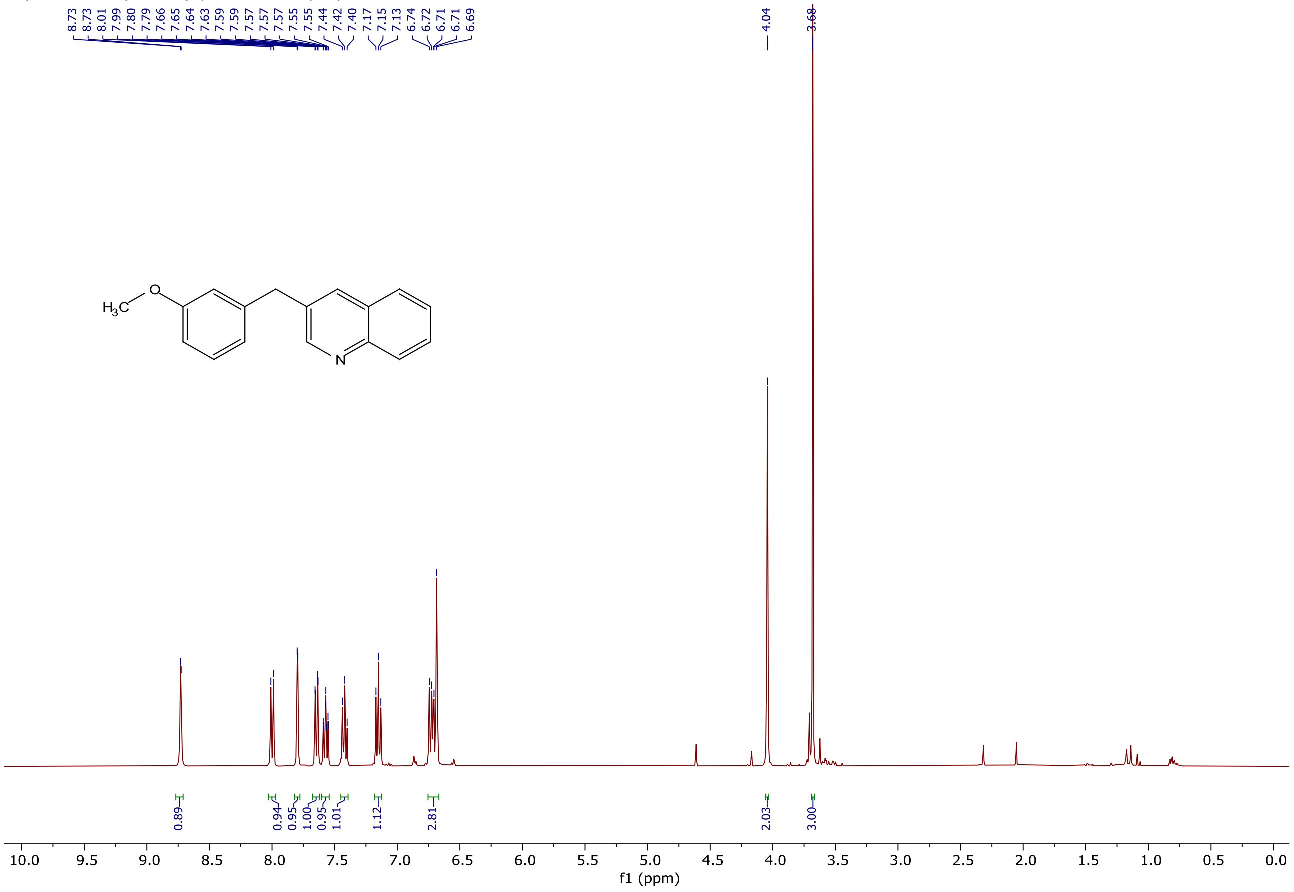

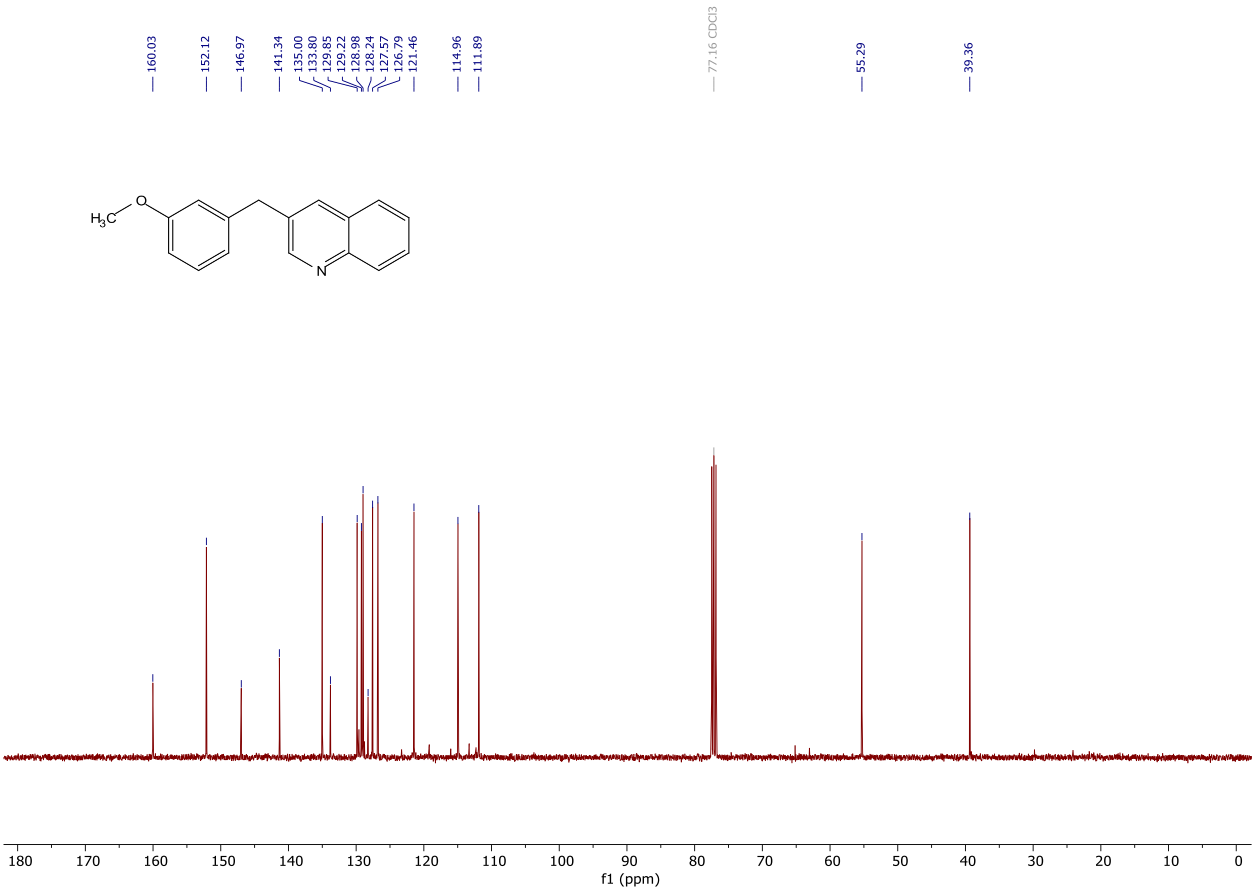

3-(4-Phenoxybenzyl)quinoline (5d)

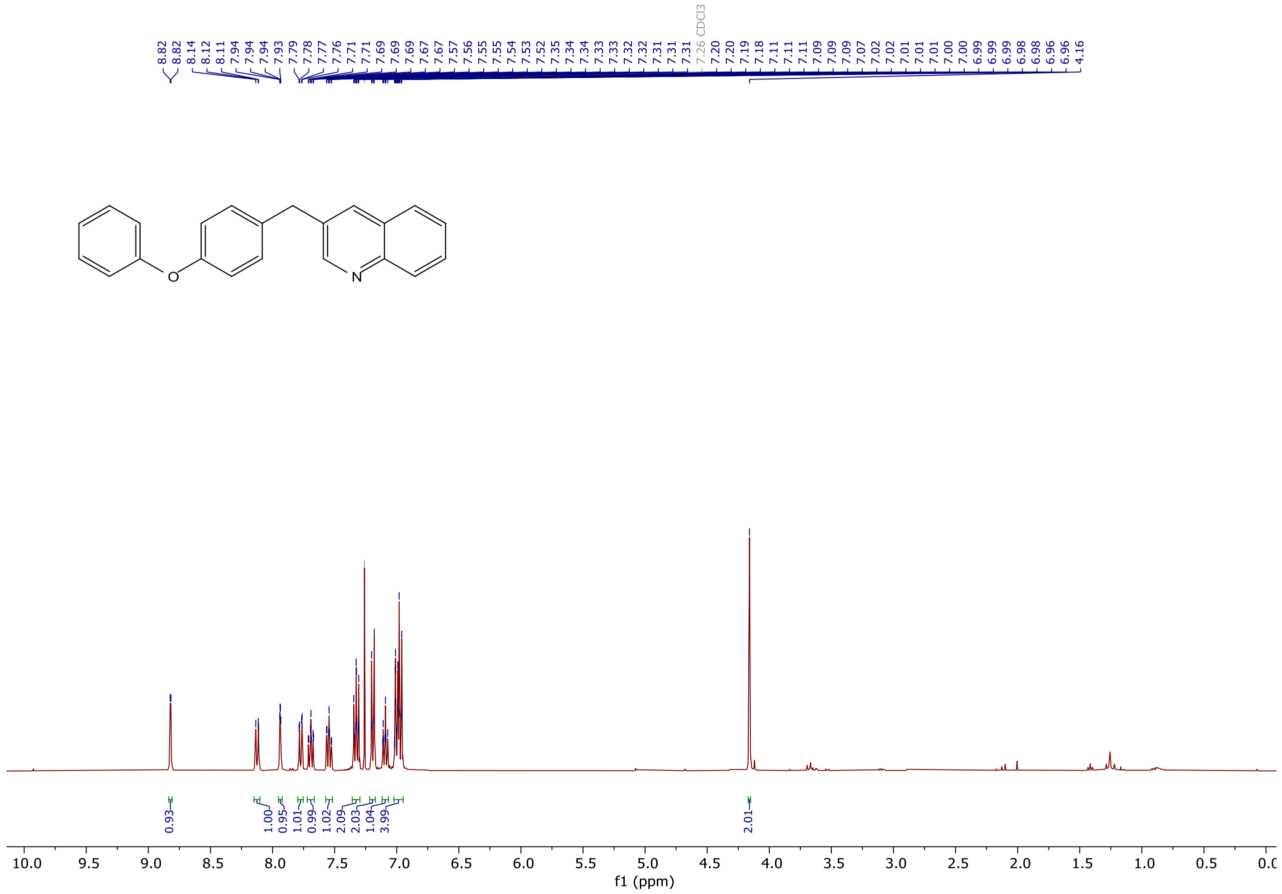

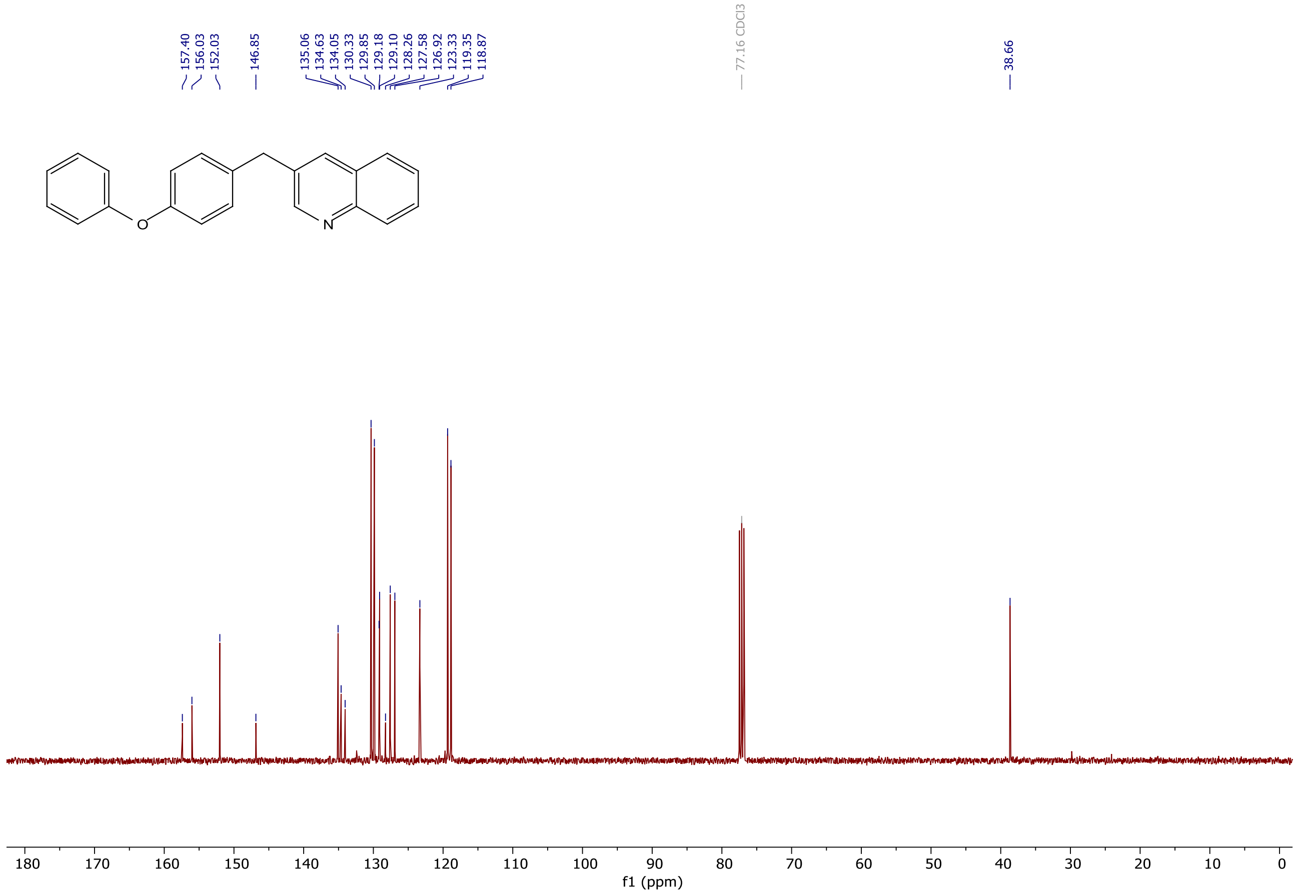

Methyl 4-(quinolin-3-ylmethyl)benzoate (5e)

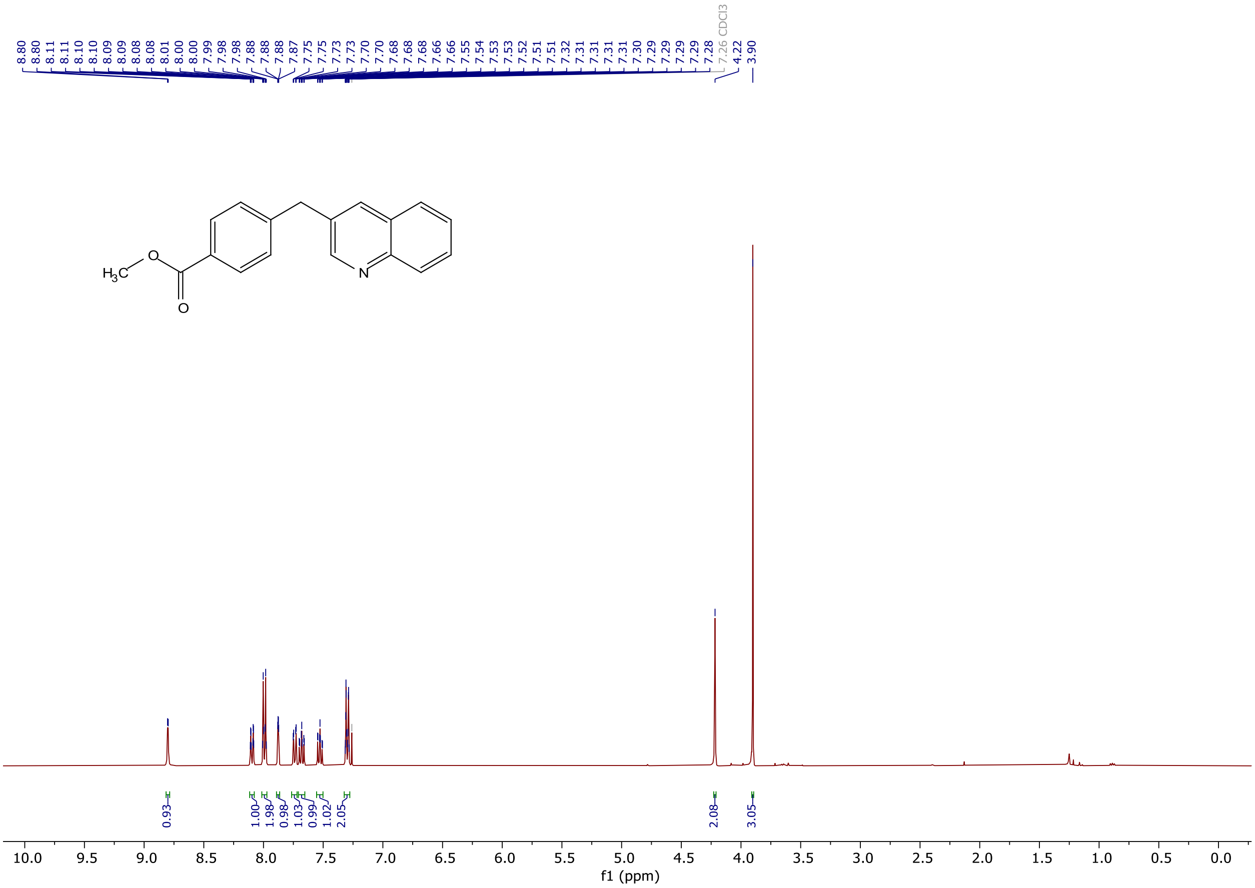

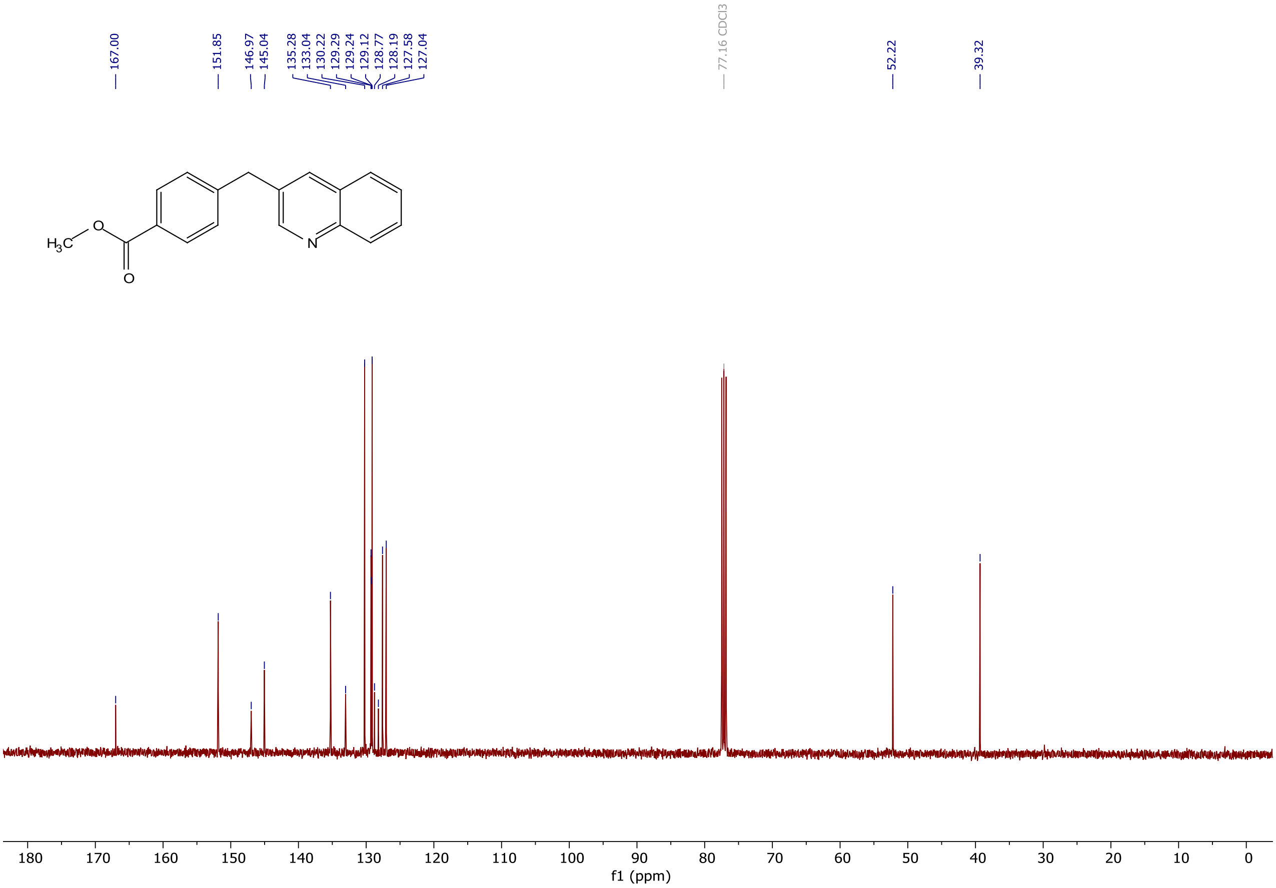

Ethyl 4-(quinolin-3-ylmethyl)benzoate (5f)

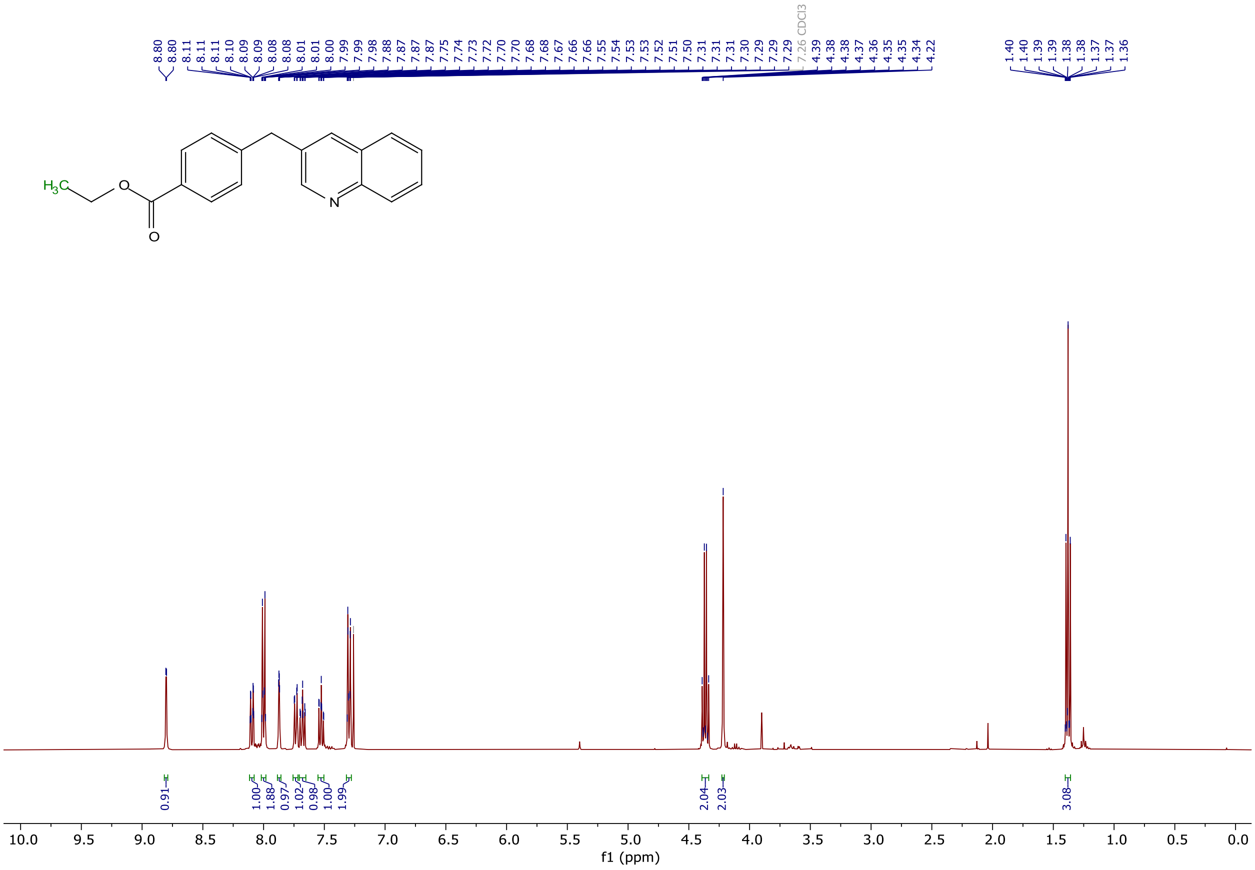

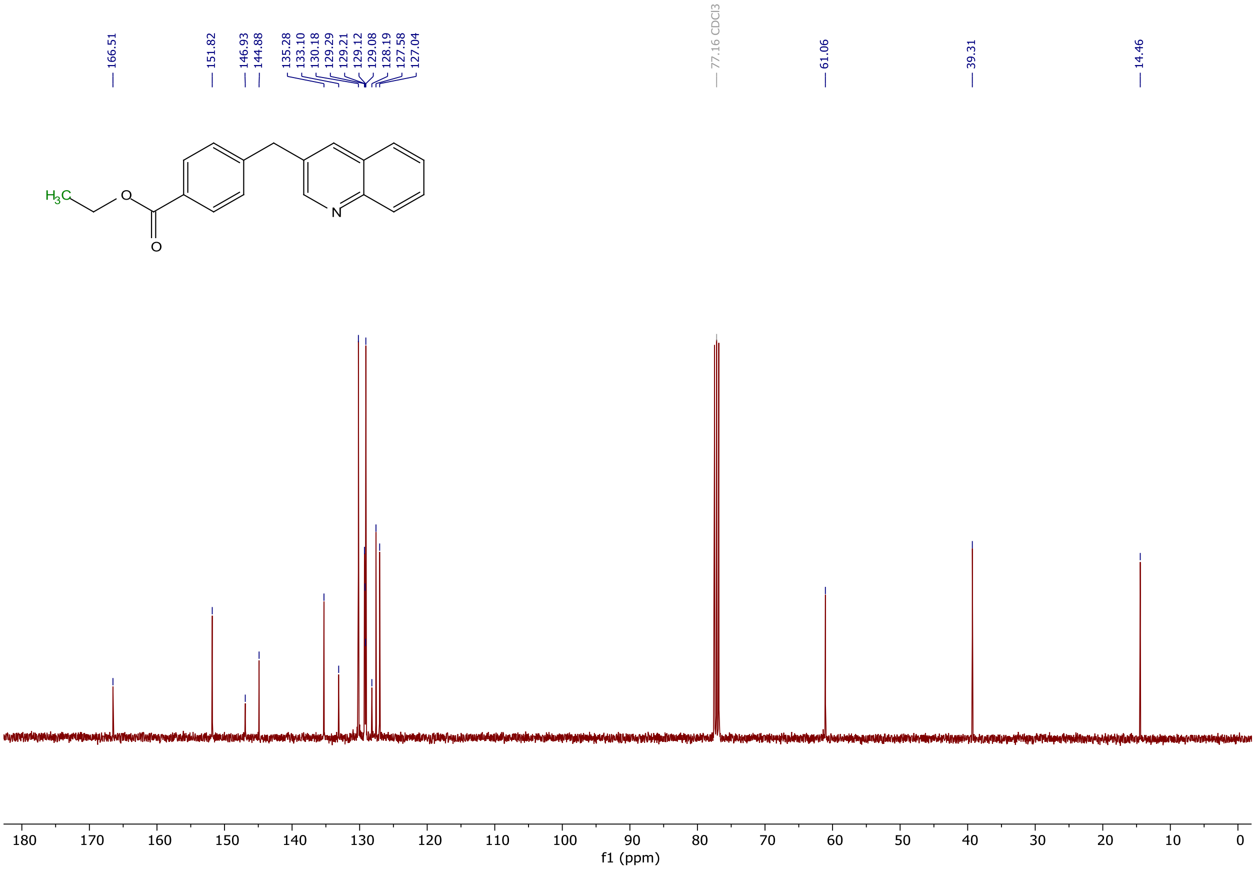

3-(3-Bromobenzyl)quinoline (5g)

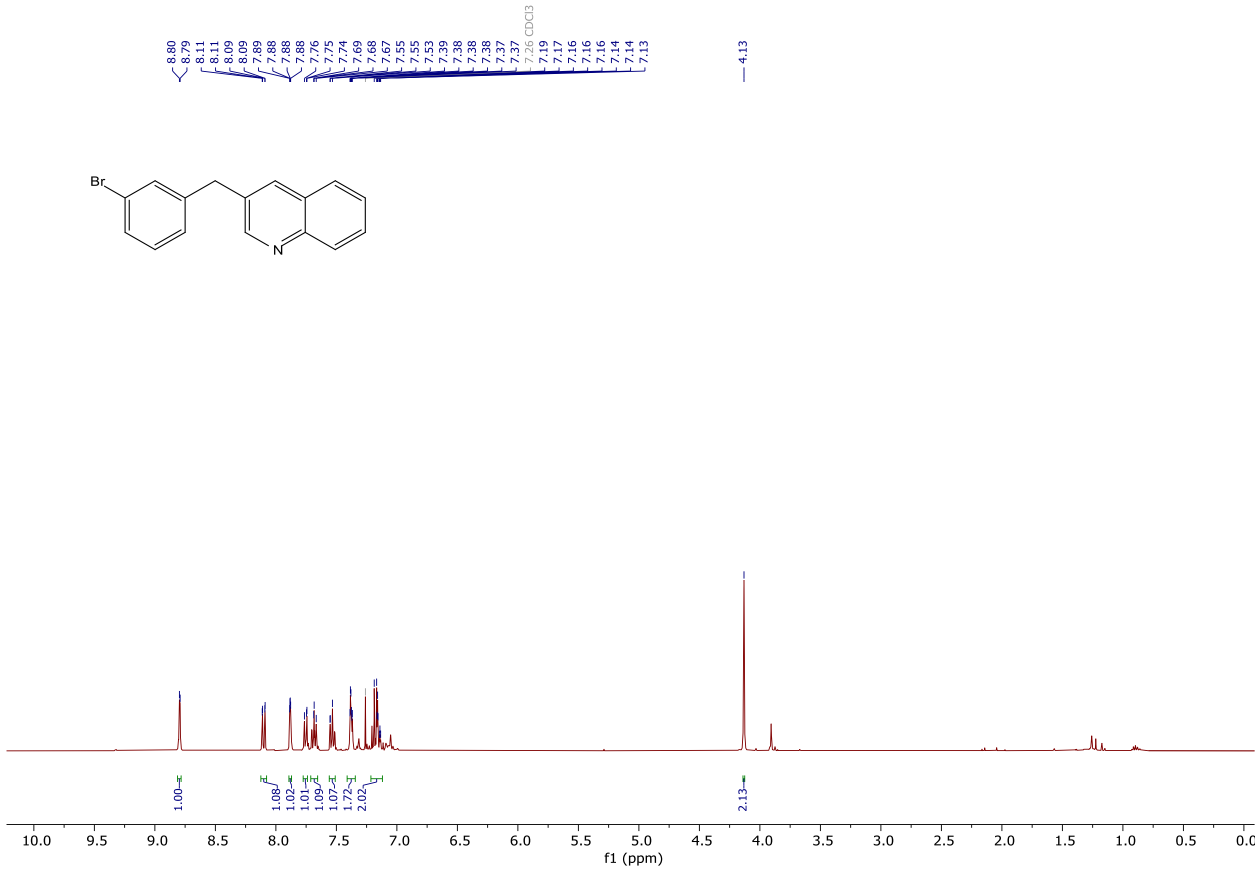

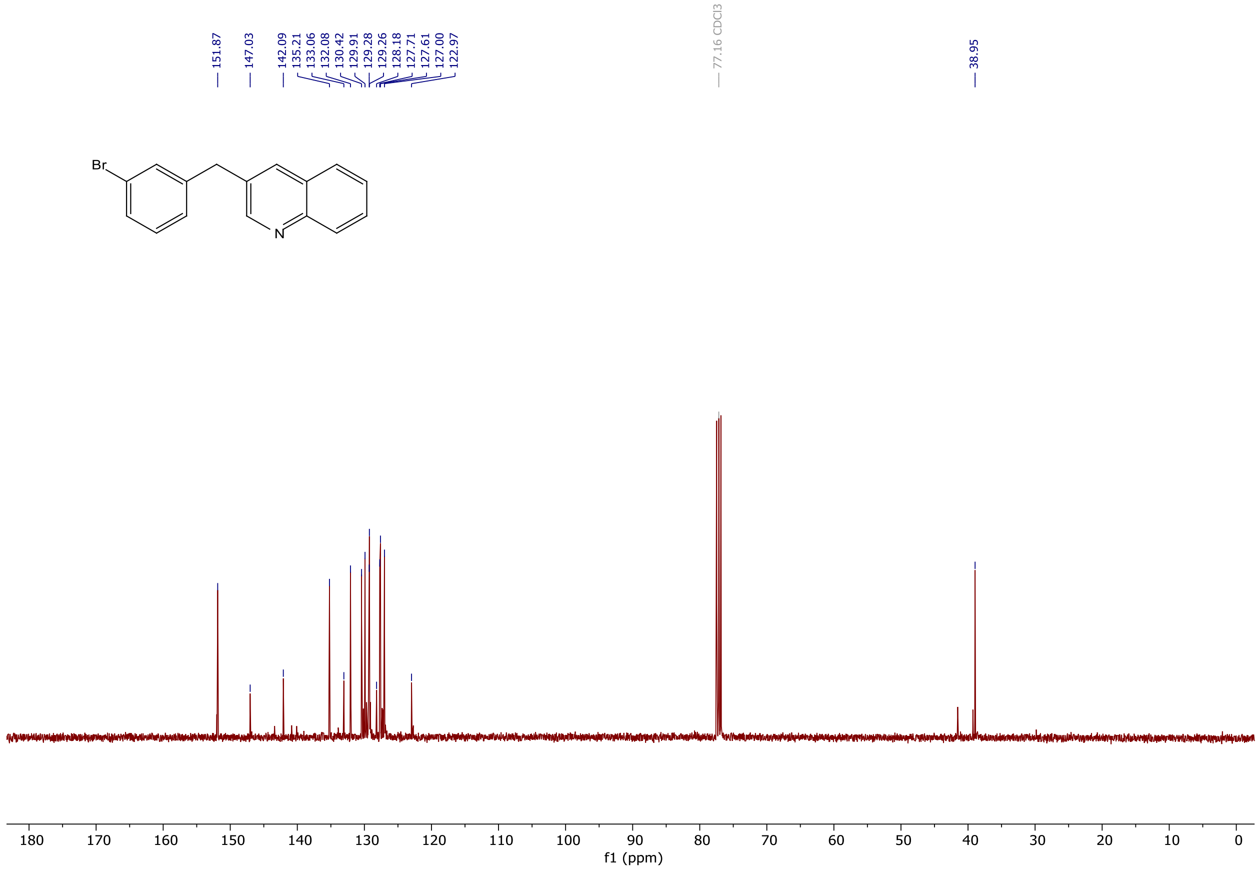

3-(2-Bromobenzyl)quinoline (5h)

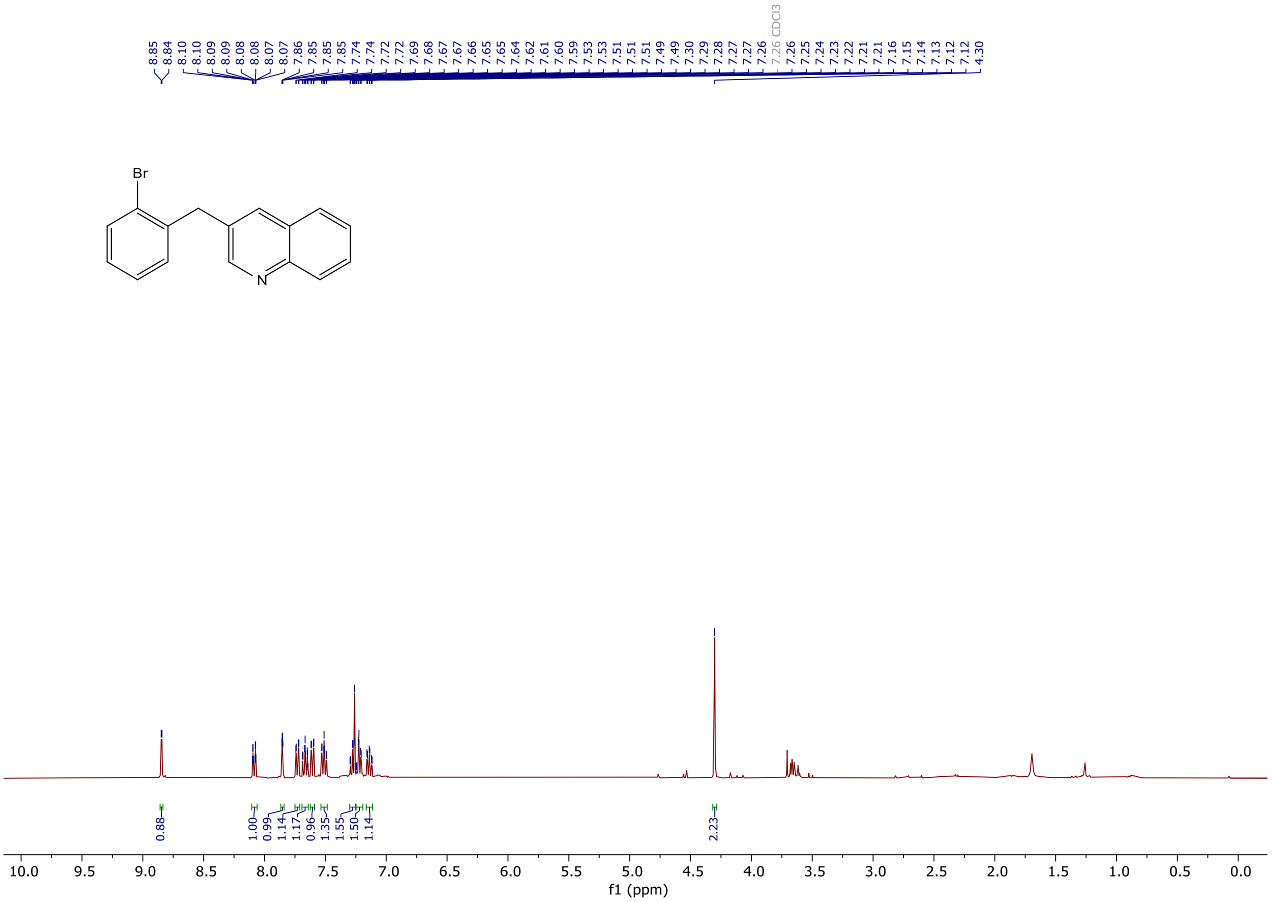

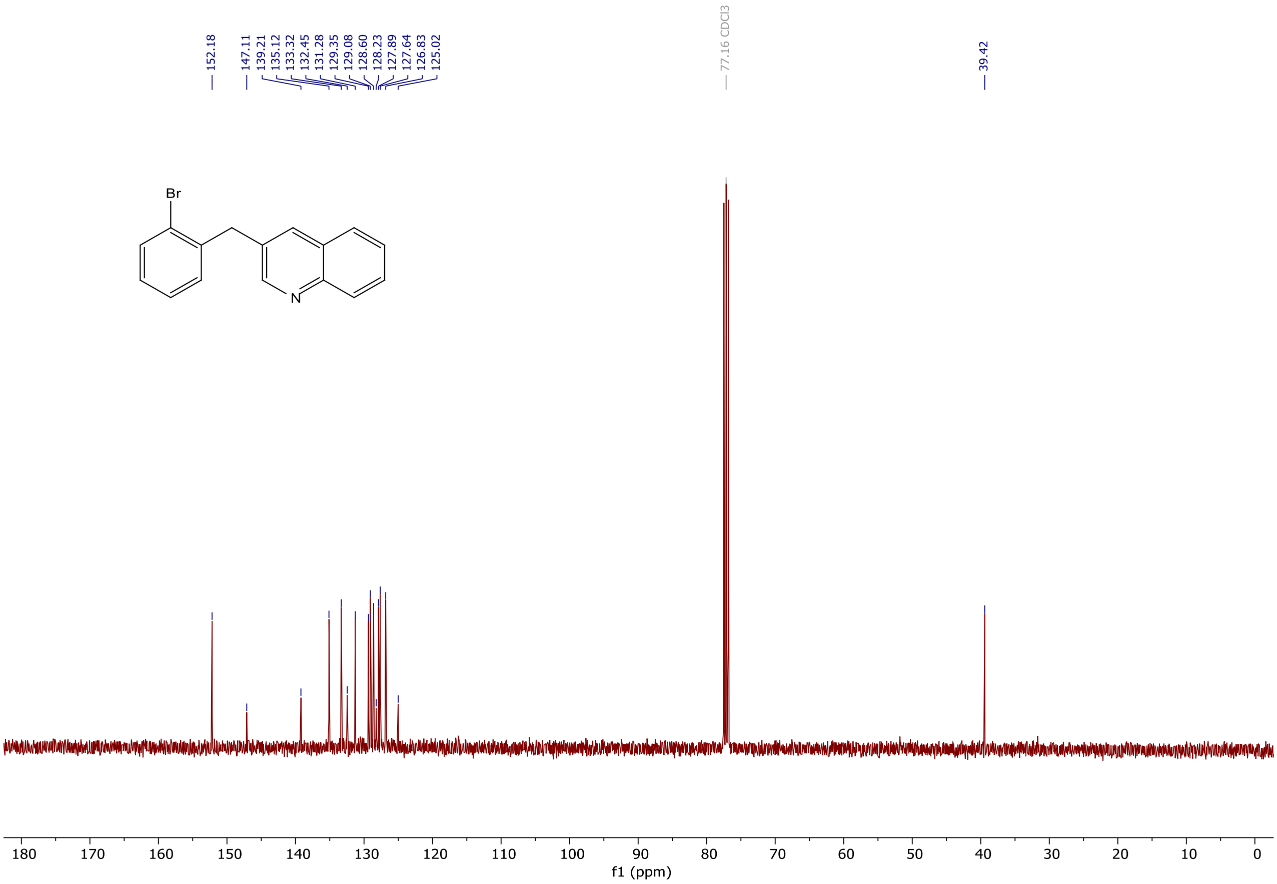

3-(3-Chlorobenzyl)quinoline (5i)

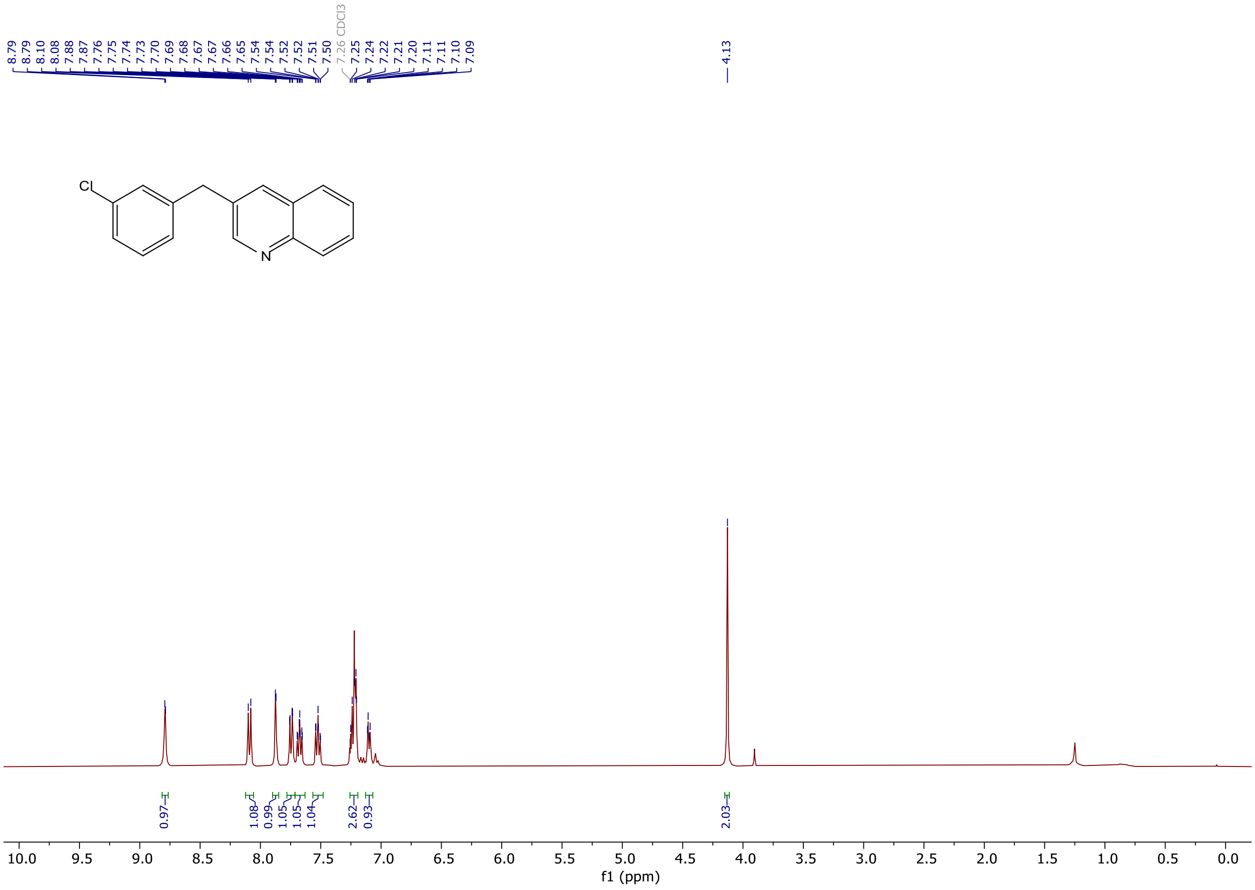

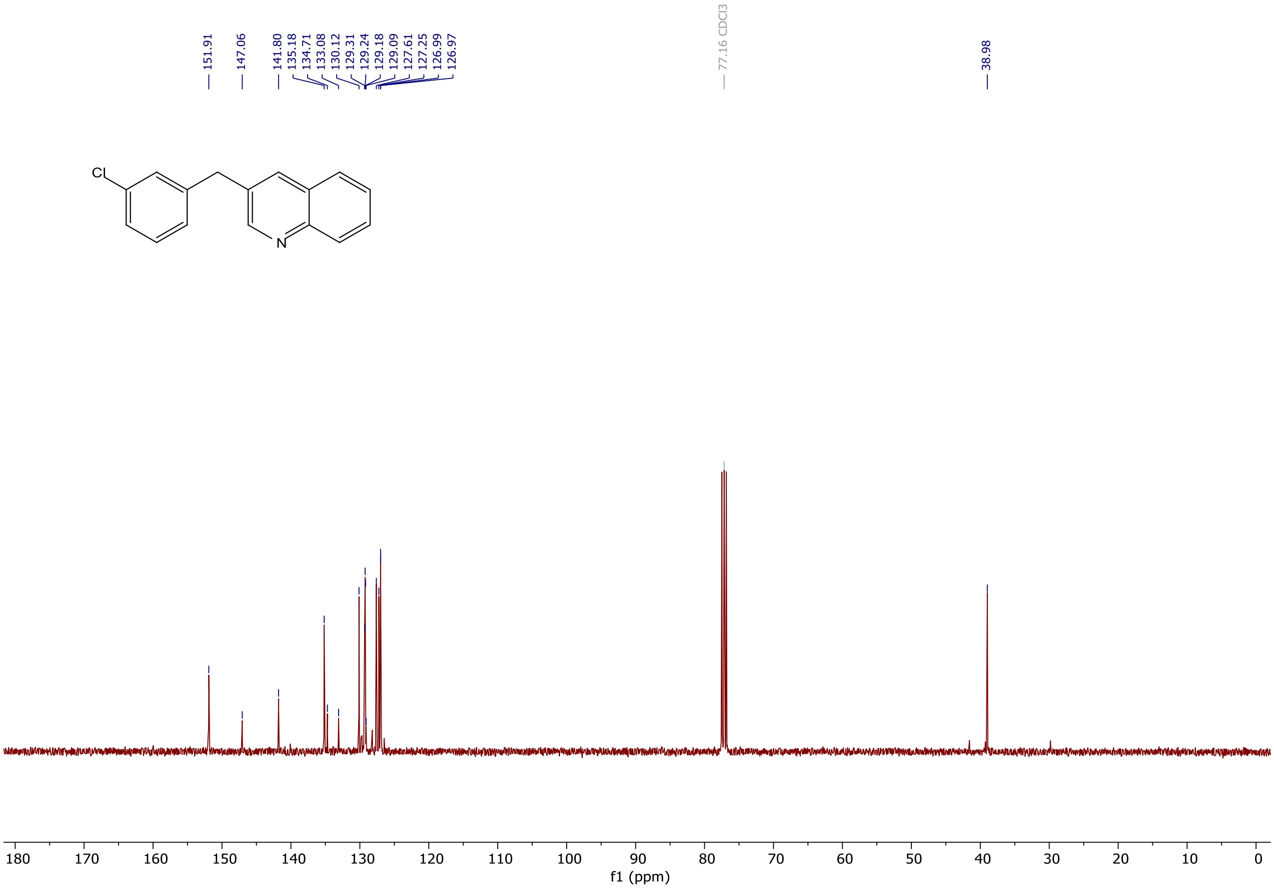

3-(3,5-Difluorobenzyl)quinoline (5j)

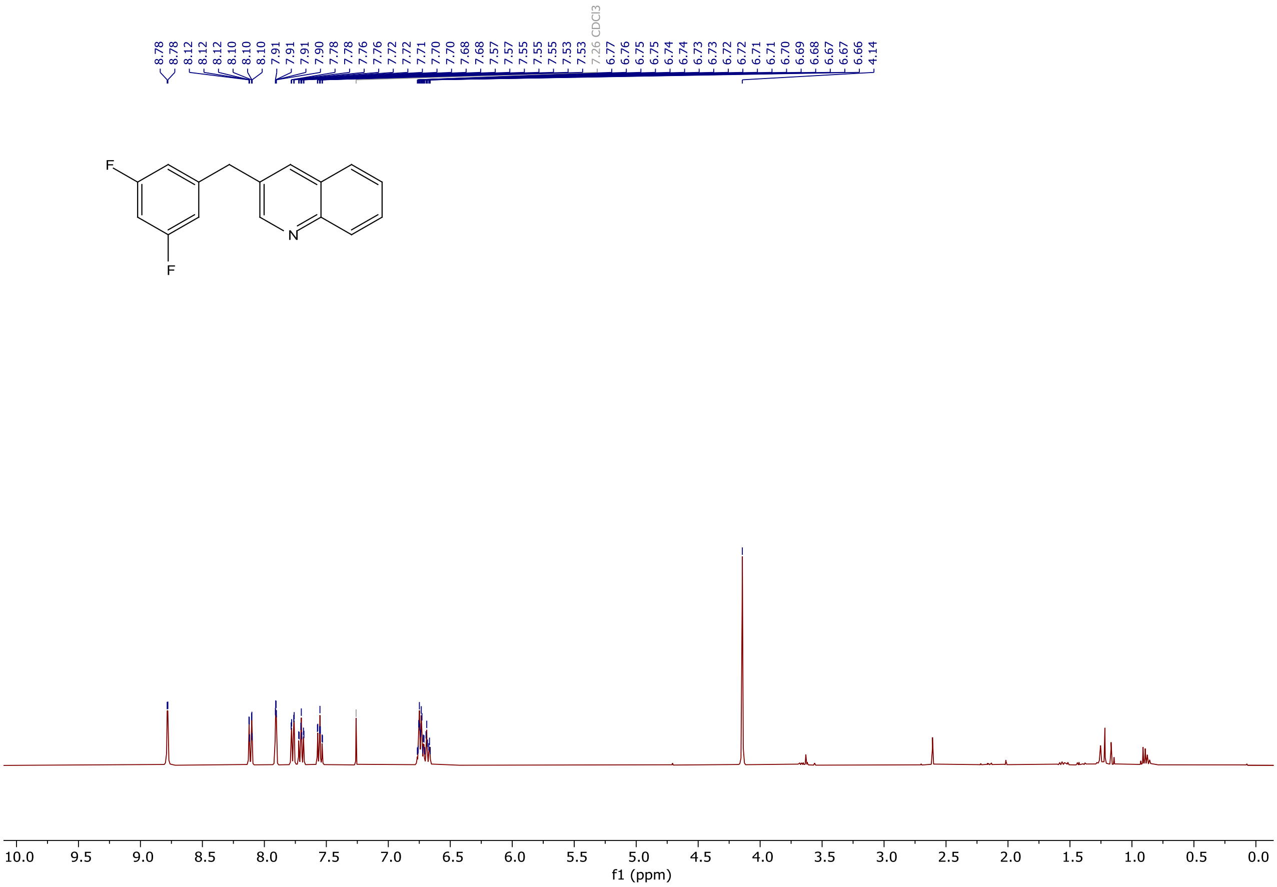

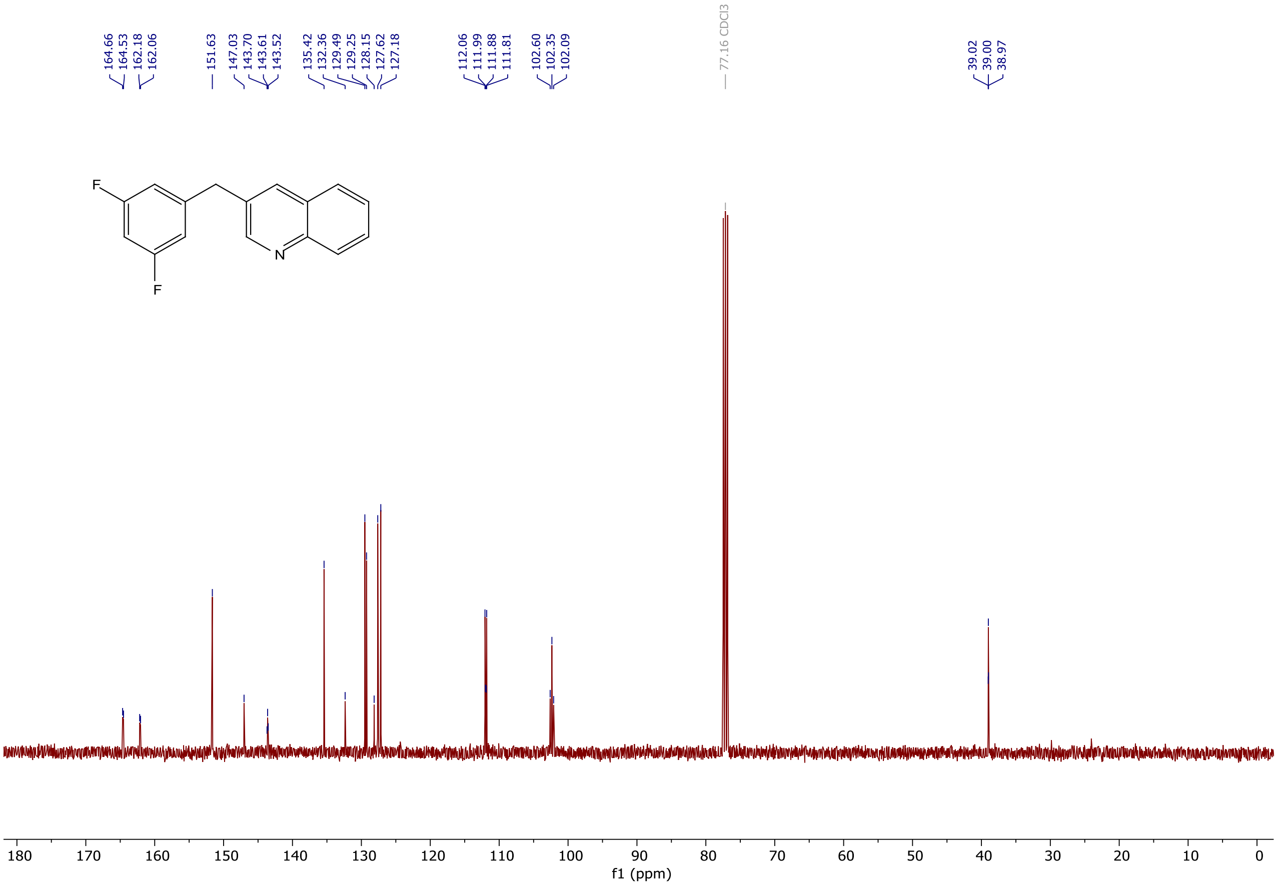

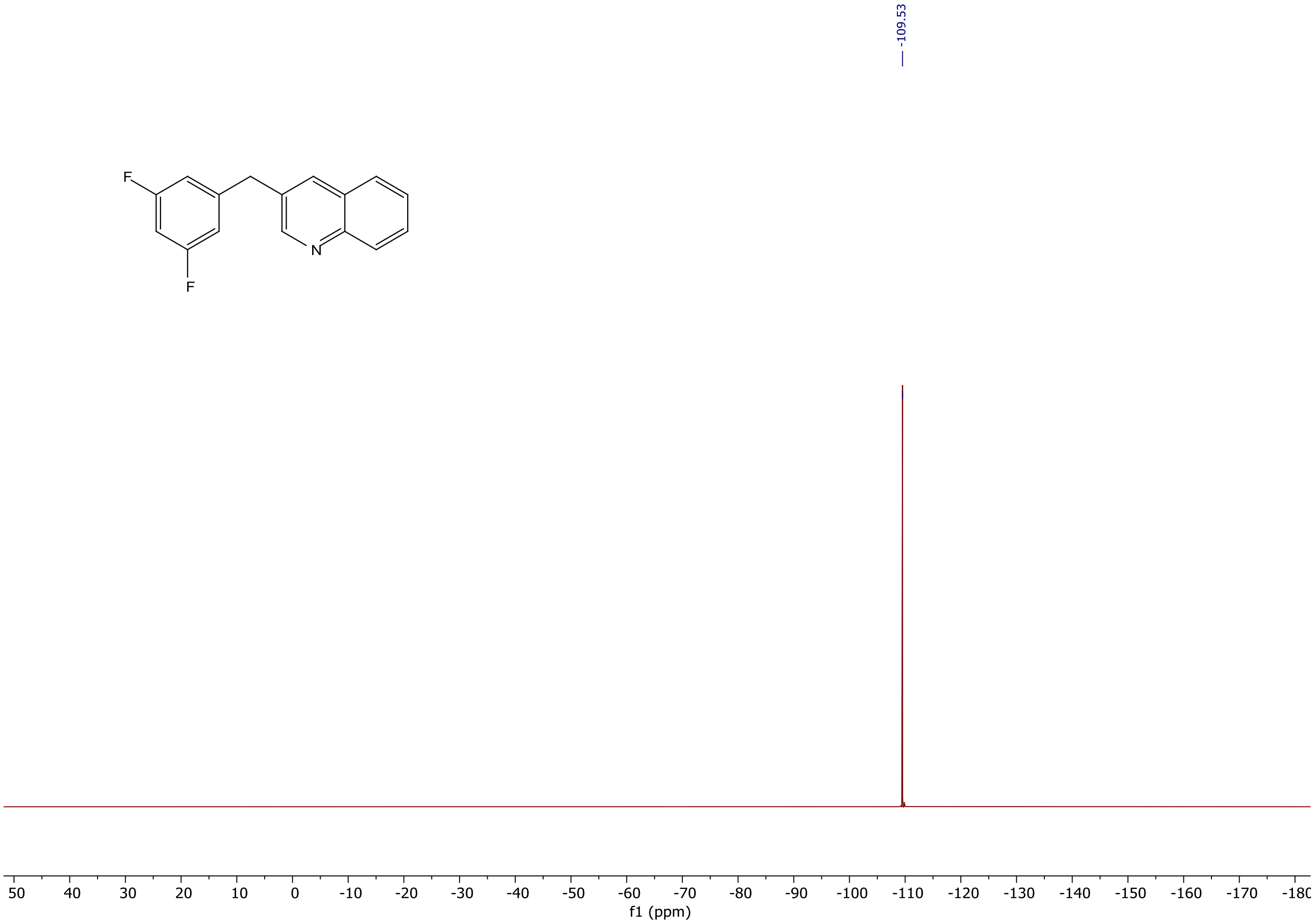
-109.53
F
F
N
50 40 30 20 10 0 -10 -20 -30 -40 -50 -60 -70 -80 -90 -100 -110 -120 -130 -140 -150 -160 -170 -180
f1 (ppm)

3-(2,6-Difluorobenzyl)quinoline (5k)

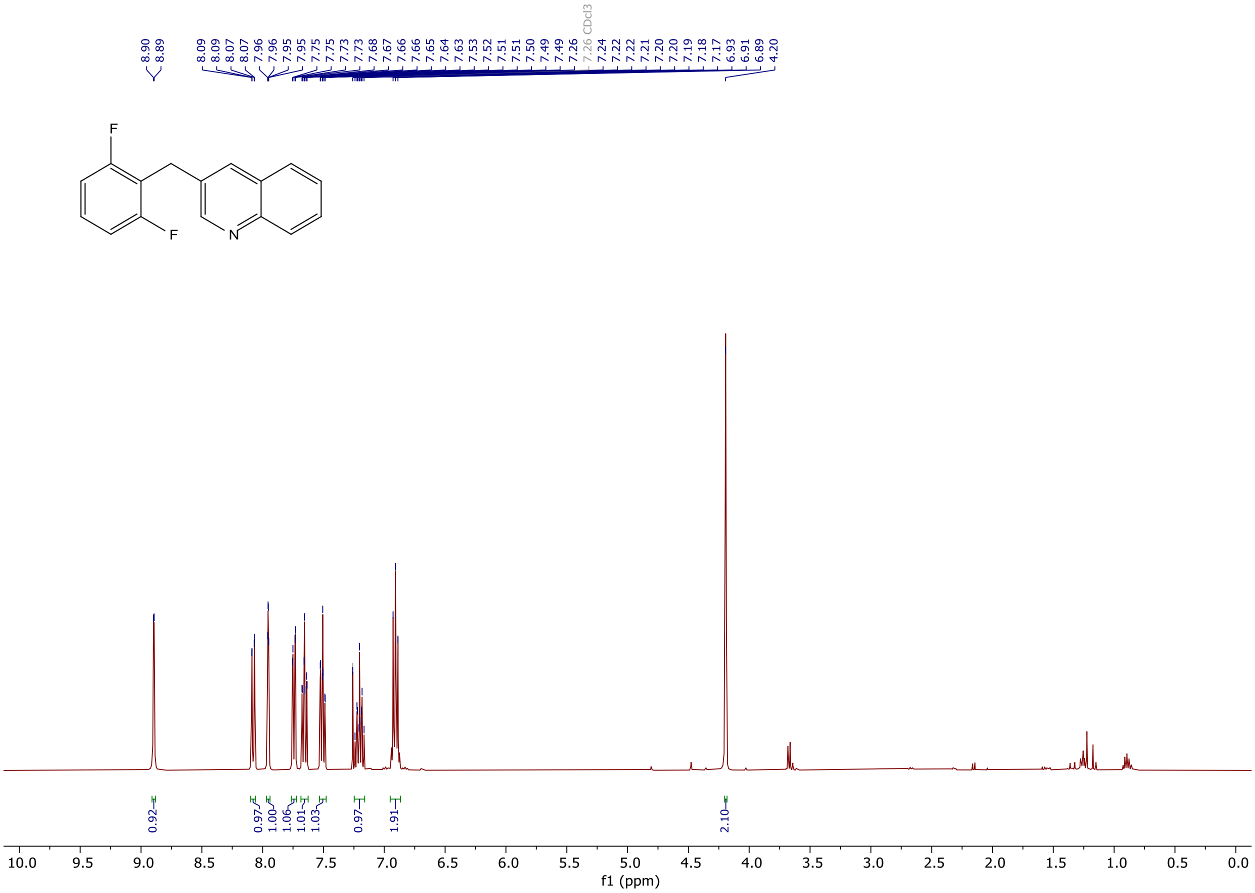

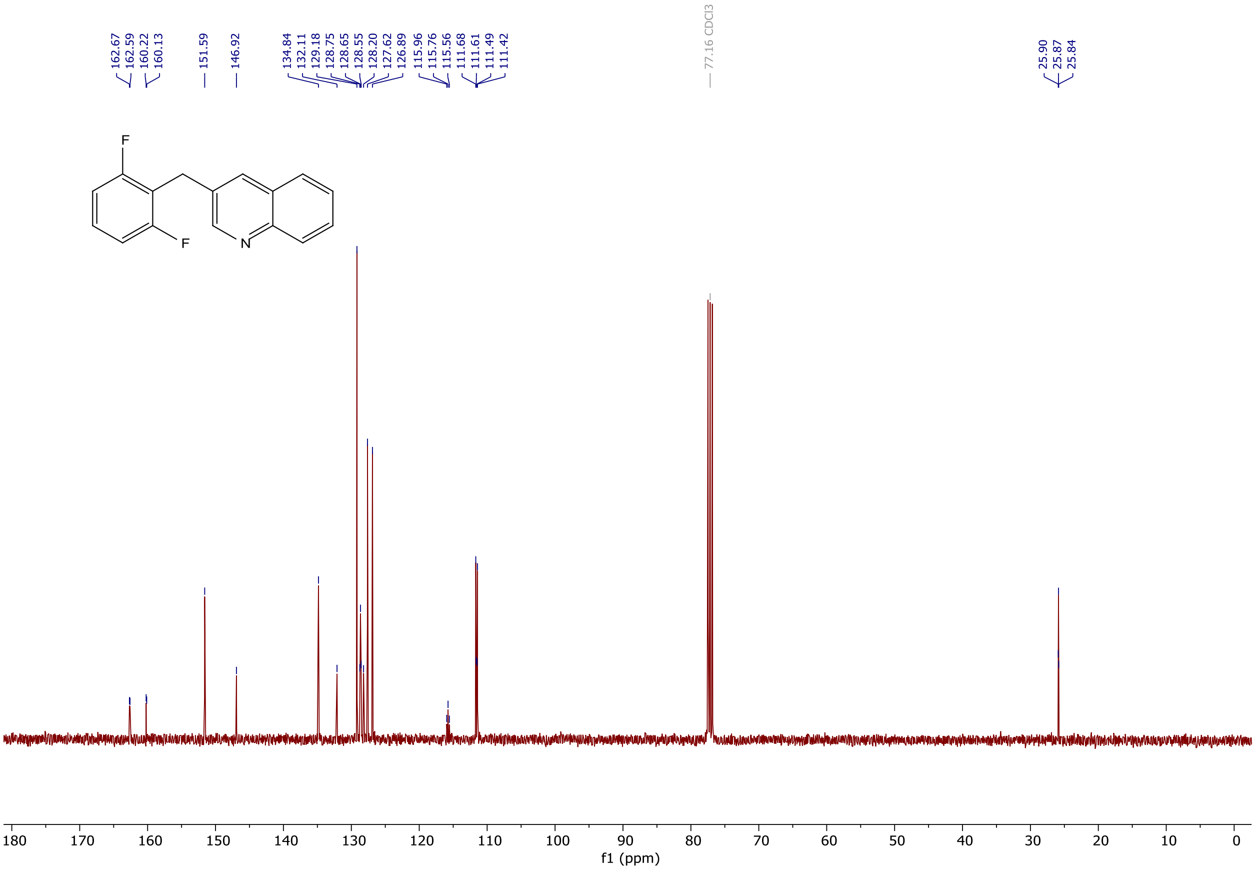

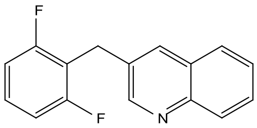
F
F
N

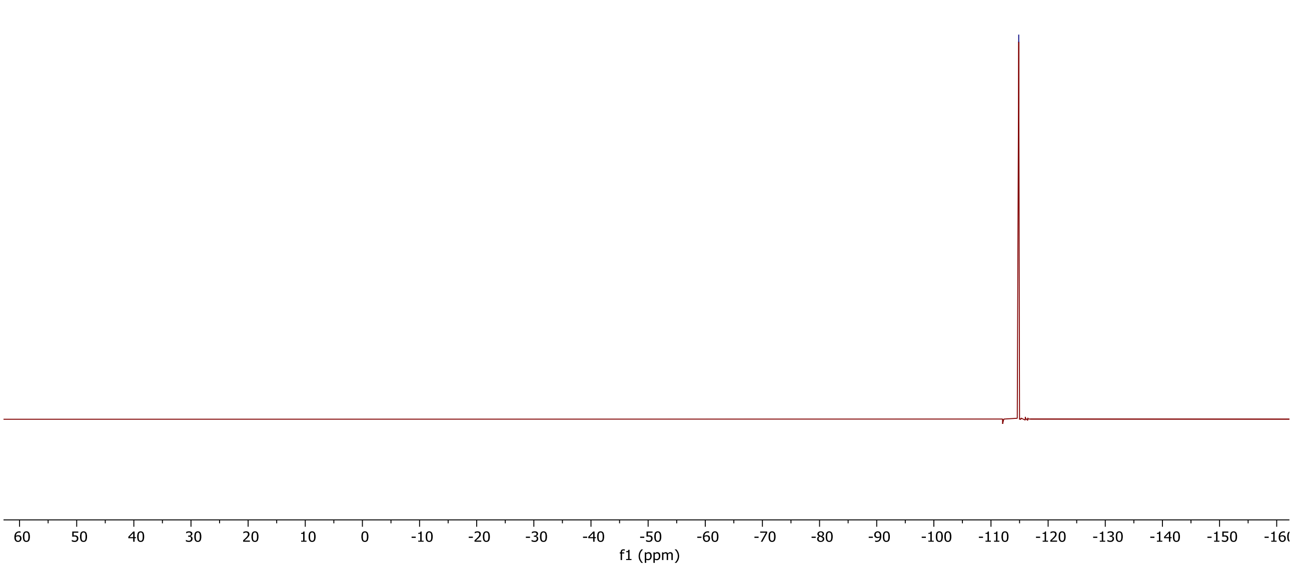
-114.85
60 50 40 30 20 10 0 -10 -20 -30 -40 -50 -60 -70 -80 -90 -100 -110 -120 -130 -140 -150
f1 (ppm)

3-(Thiophen-3-ylmethyl)quinoline (5l)

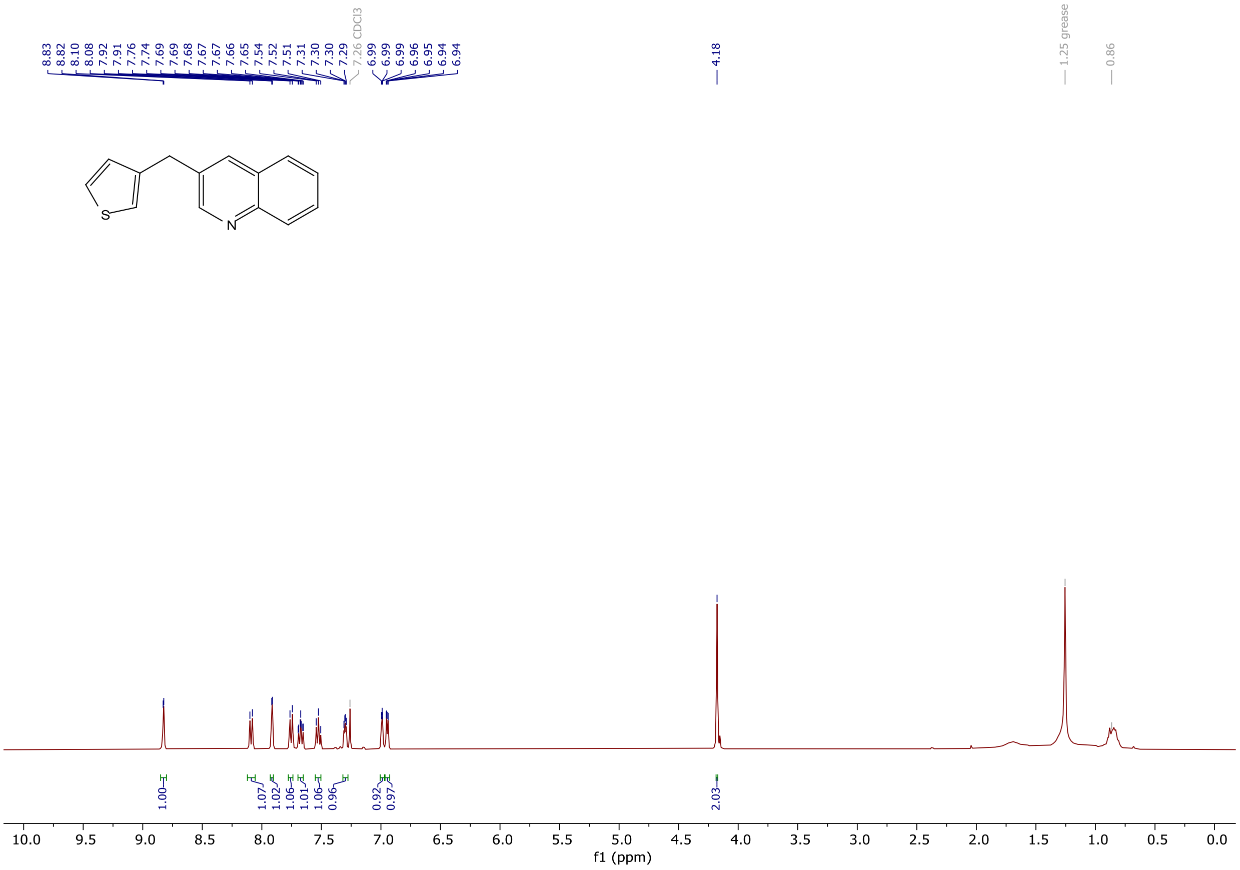

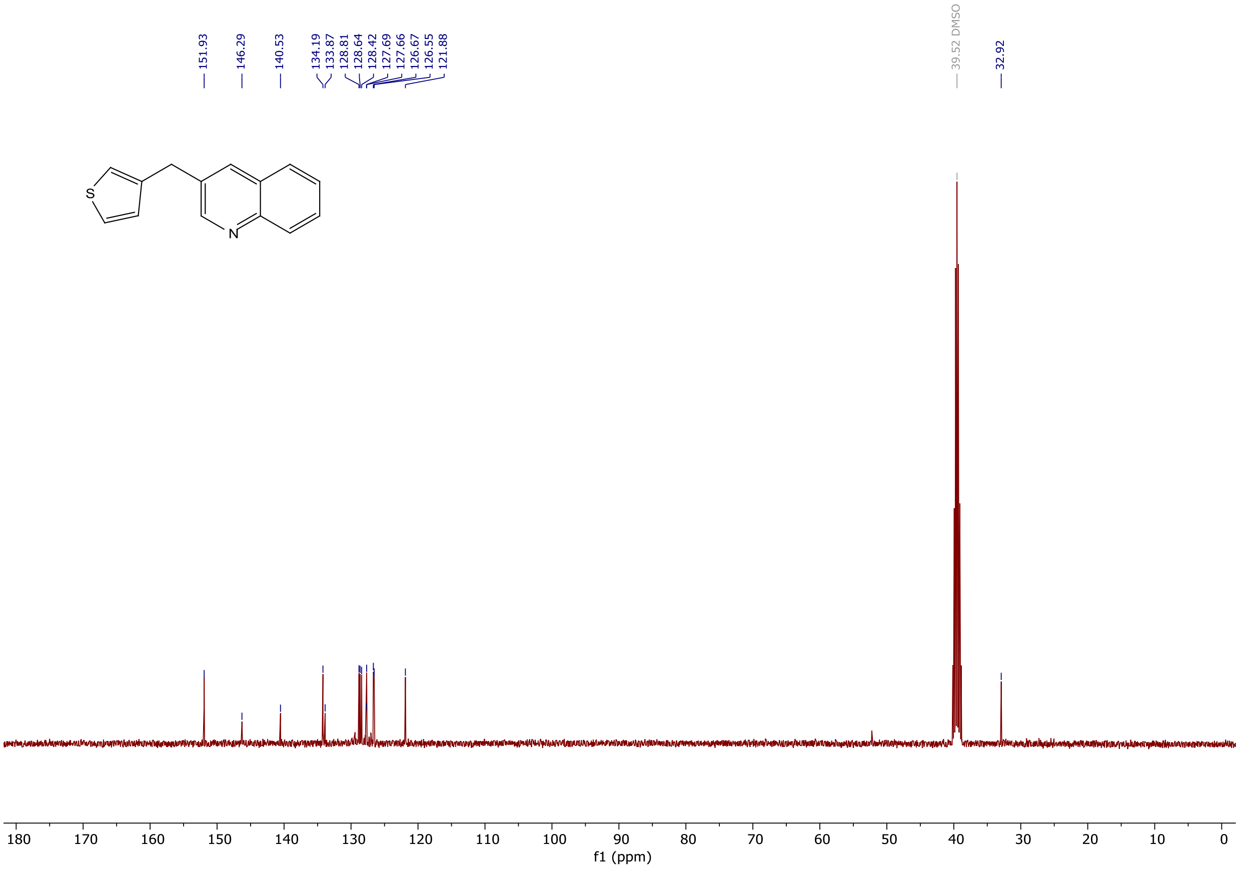

3,5-Dimethyl-4-(quinolin-3-ylmethyl)isoxazole (5m)

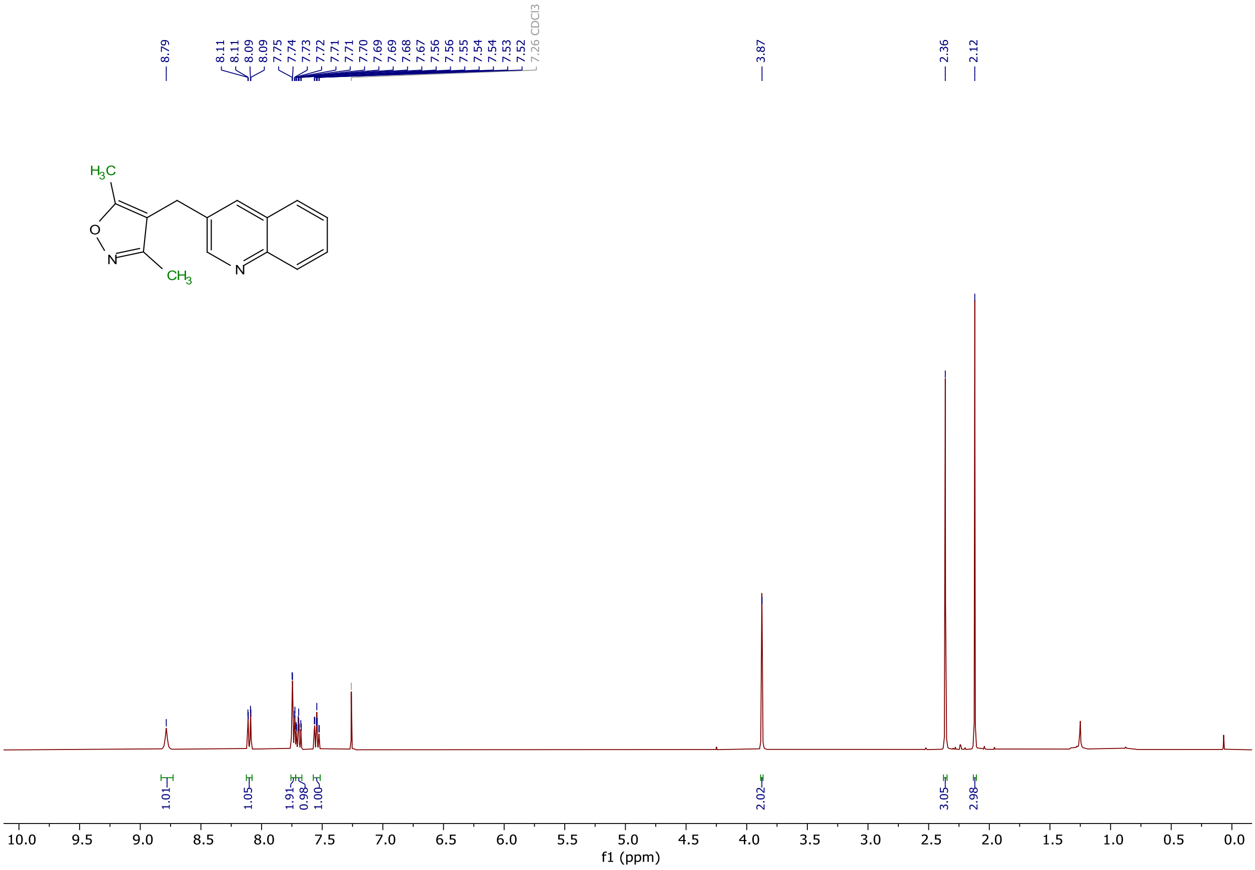

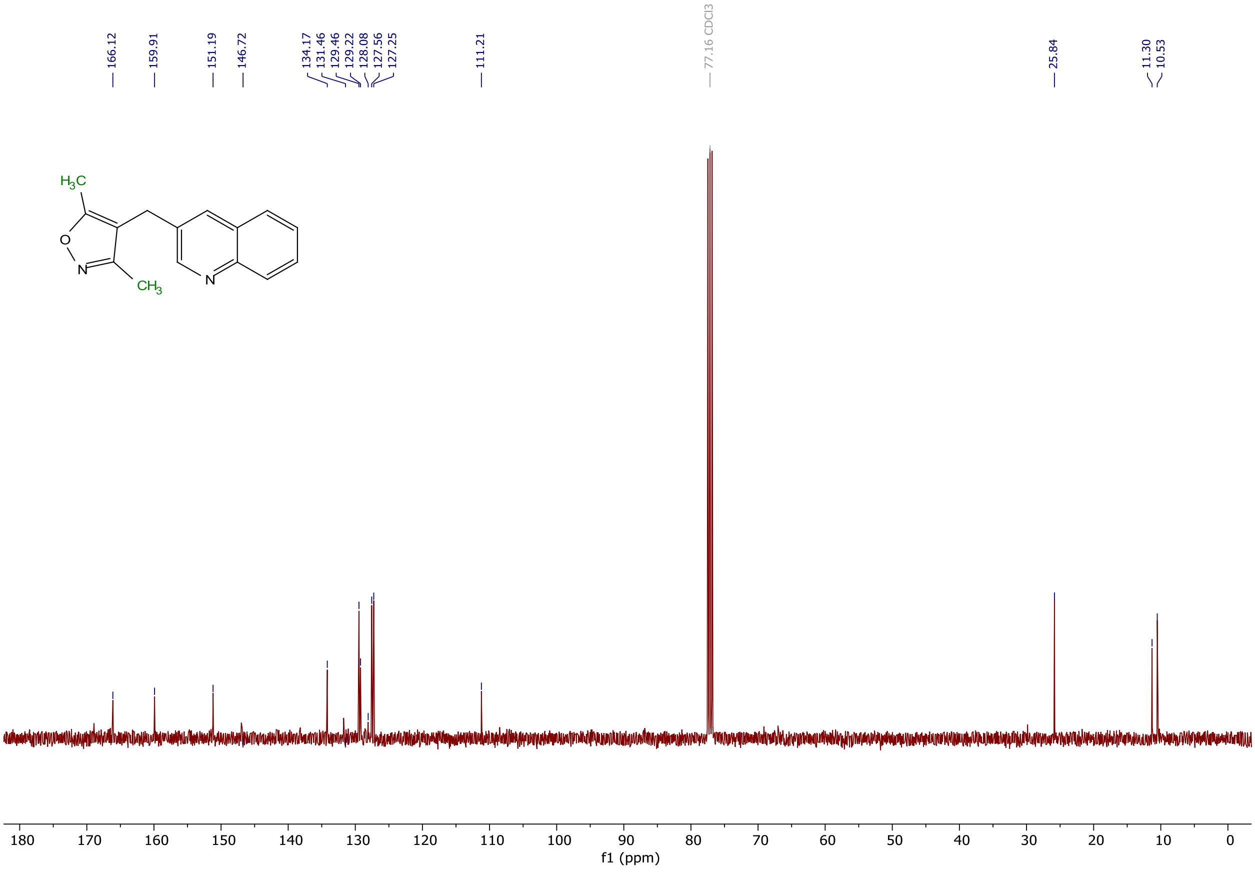

4-Butoxy-6-(quinolin-3-ylmethyl)-2-(trifluoromethyl)quinoline (5n)

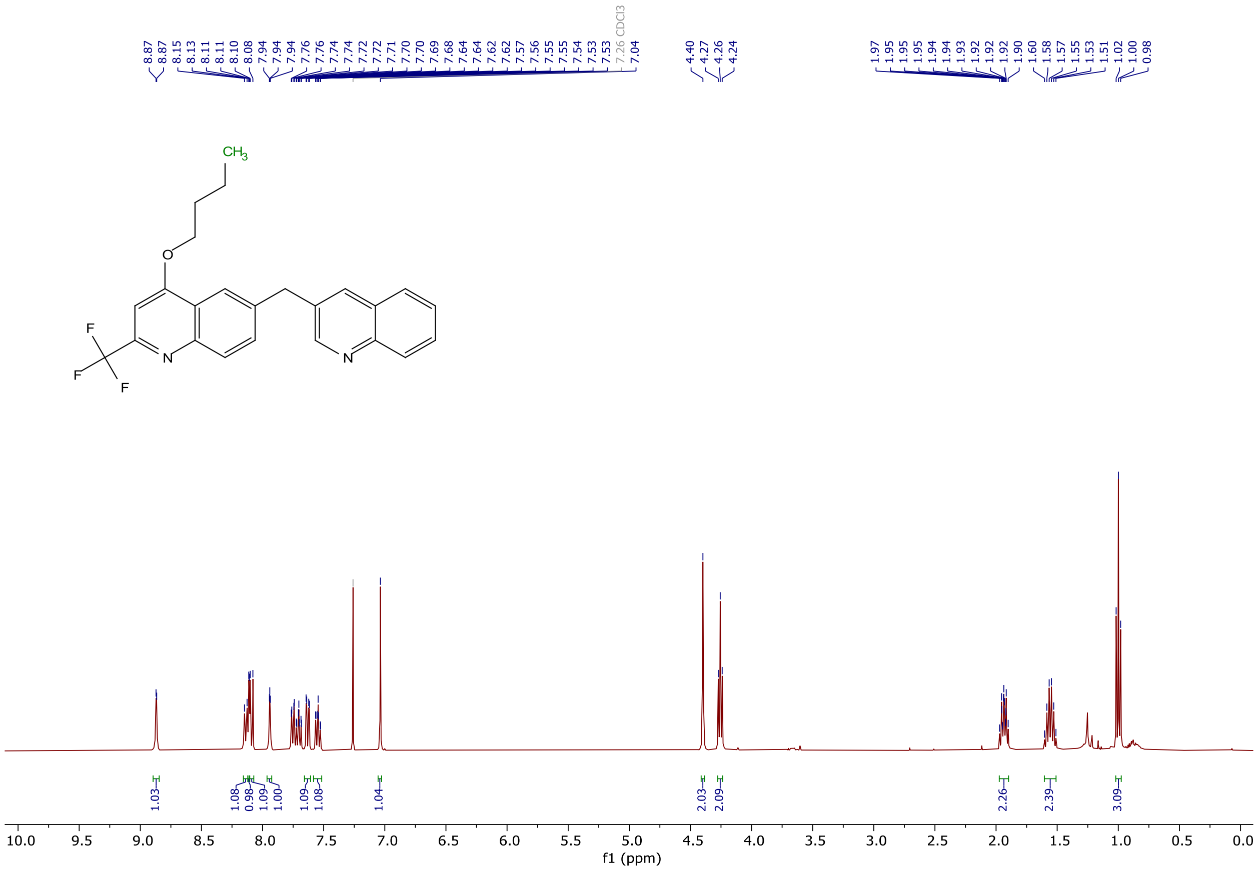

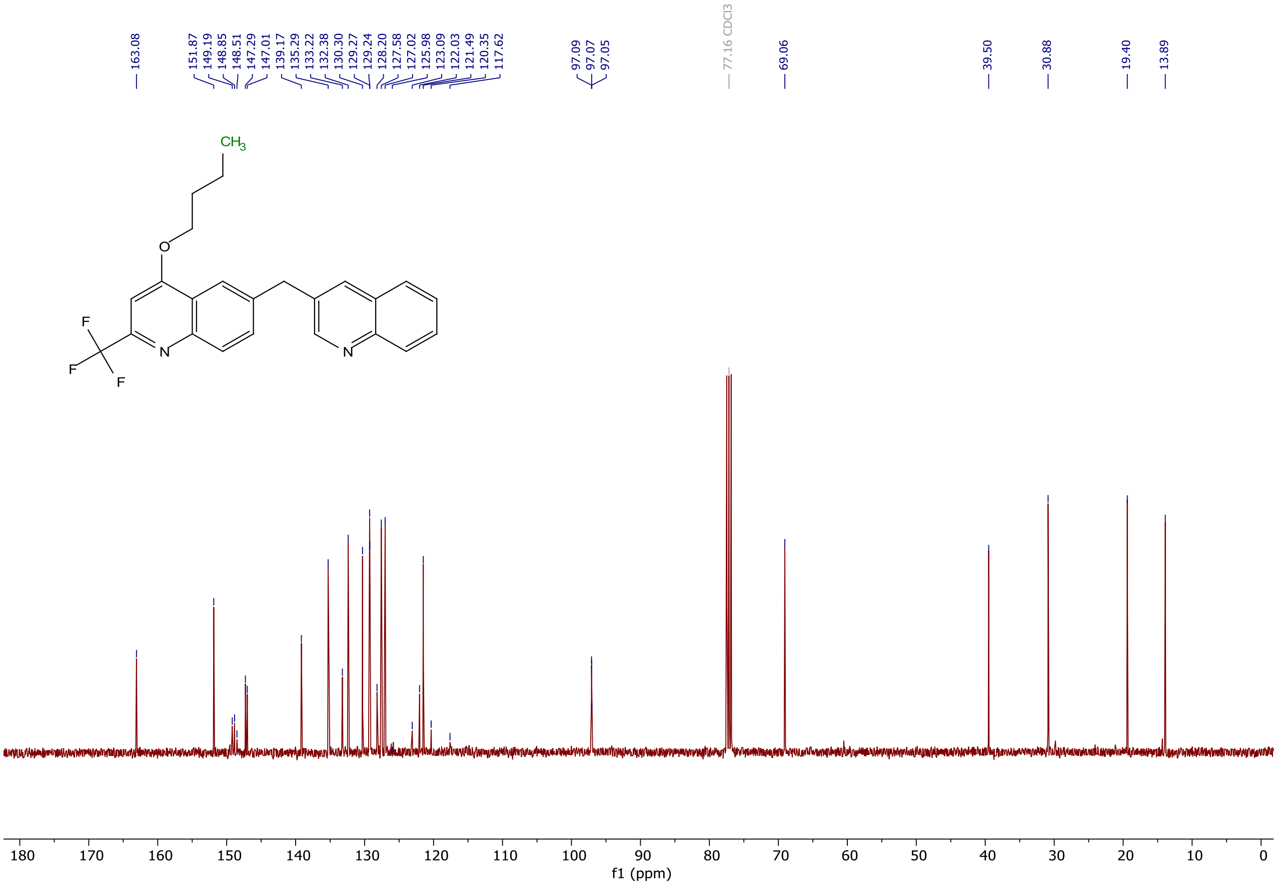

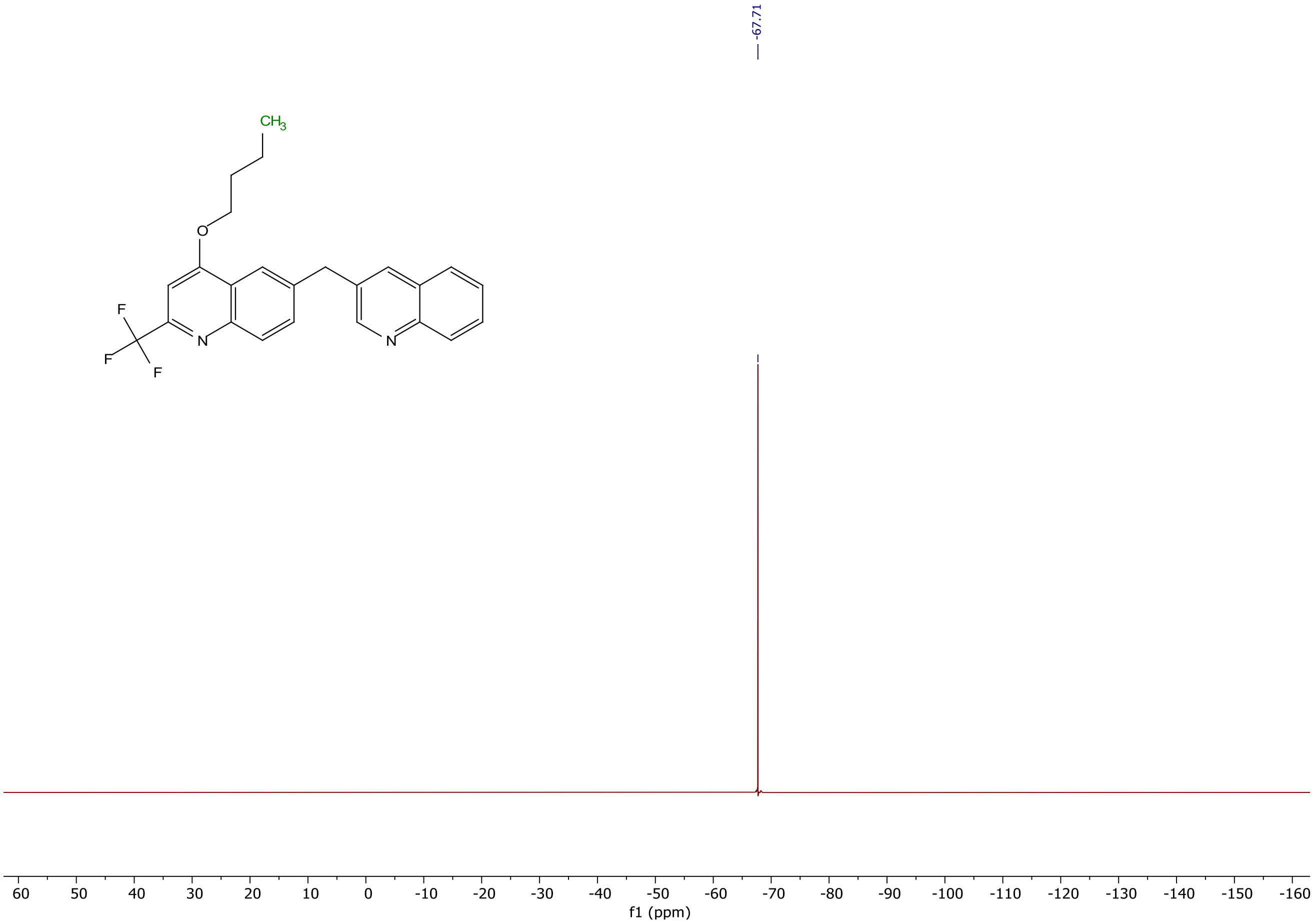
-67.71
CH3
O
F
F
F
N
N
60
50
40
30
20
10
0
-10
-20
-30
-40
-50
-60
-70
-80
-90
-100
-110
-120
-130
-140
-150
-160
f1 (ppm)

6-(Quinolin-3-ylmethyl)benzo[d]thiazole (5o)

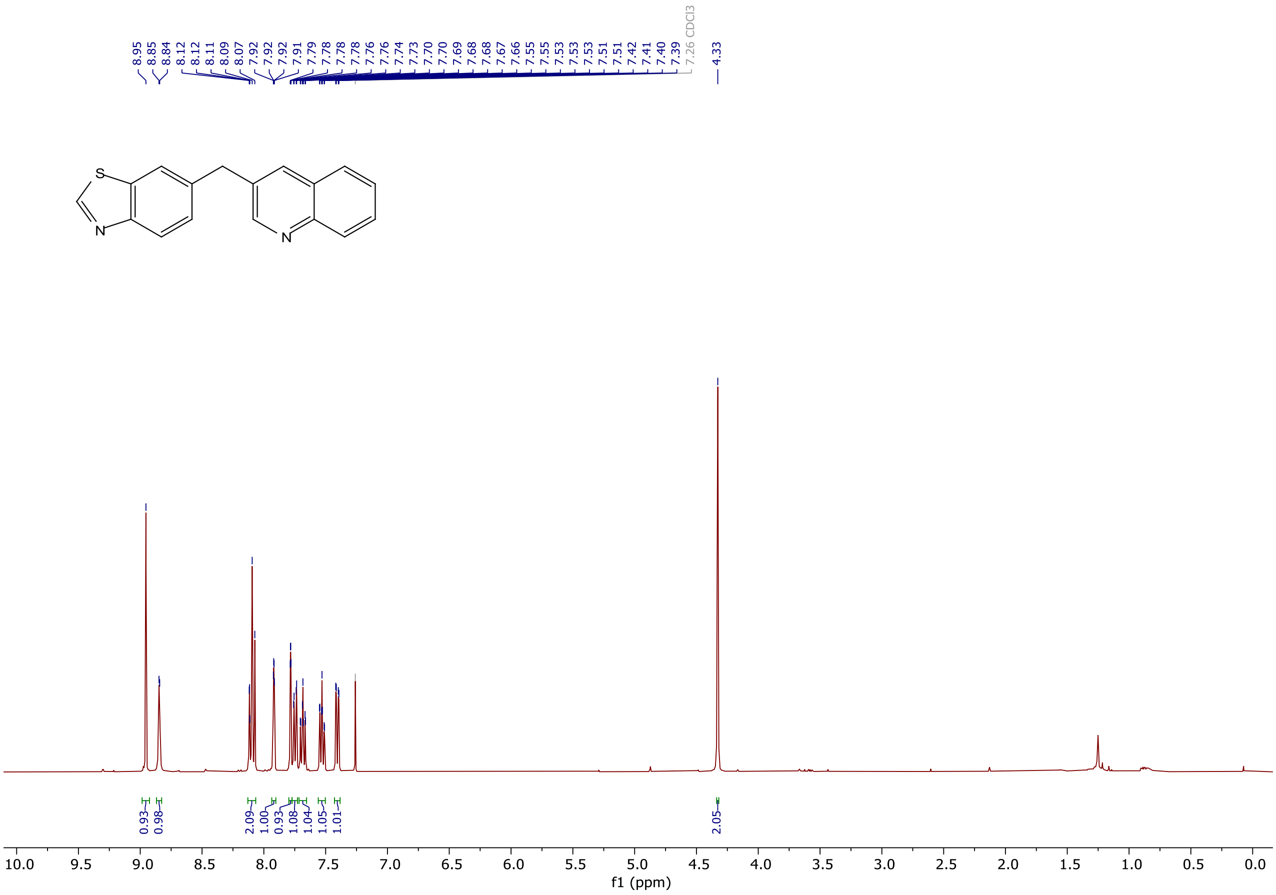

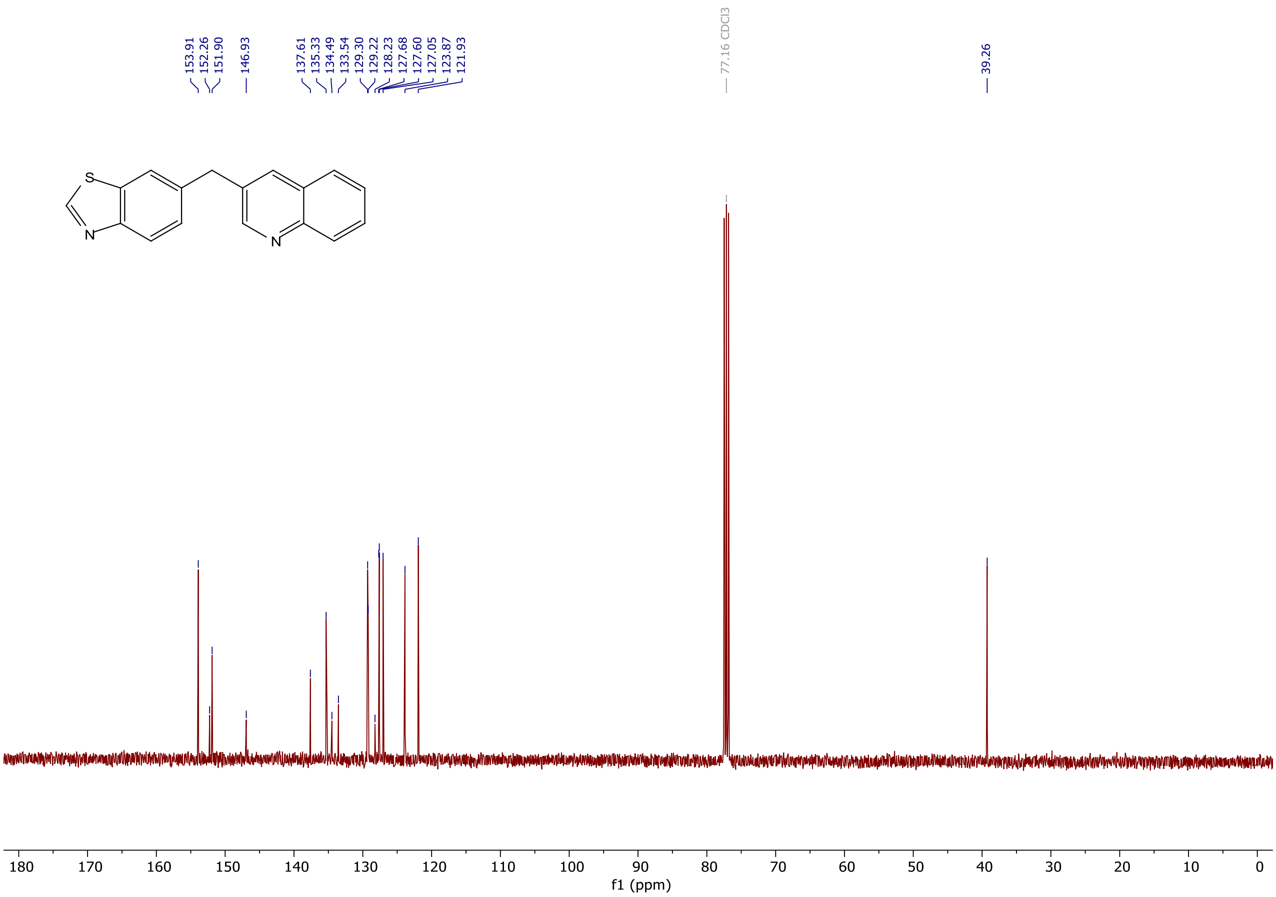

(E)-3-(3-phenylprop-1-en-1-yl)quinoline (5q')

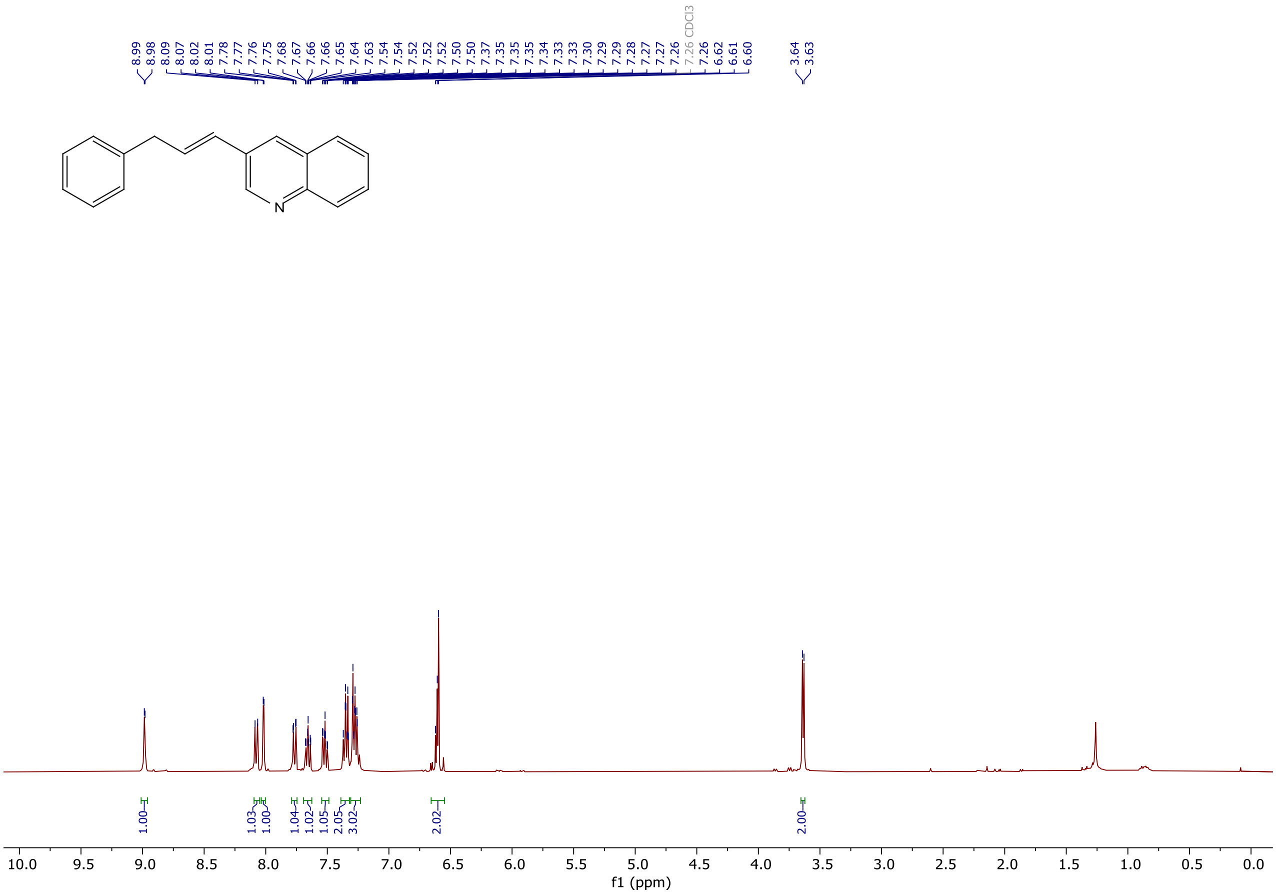

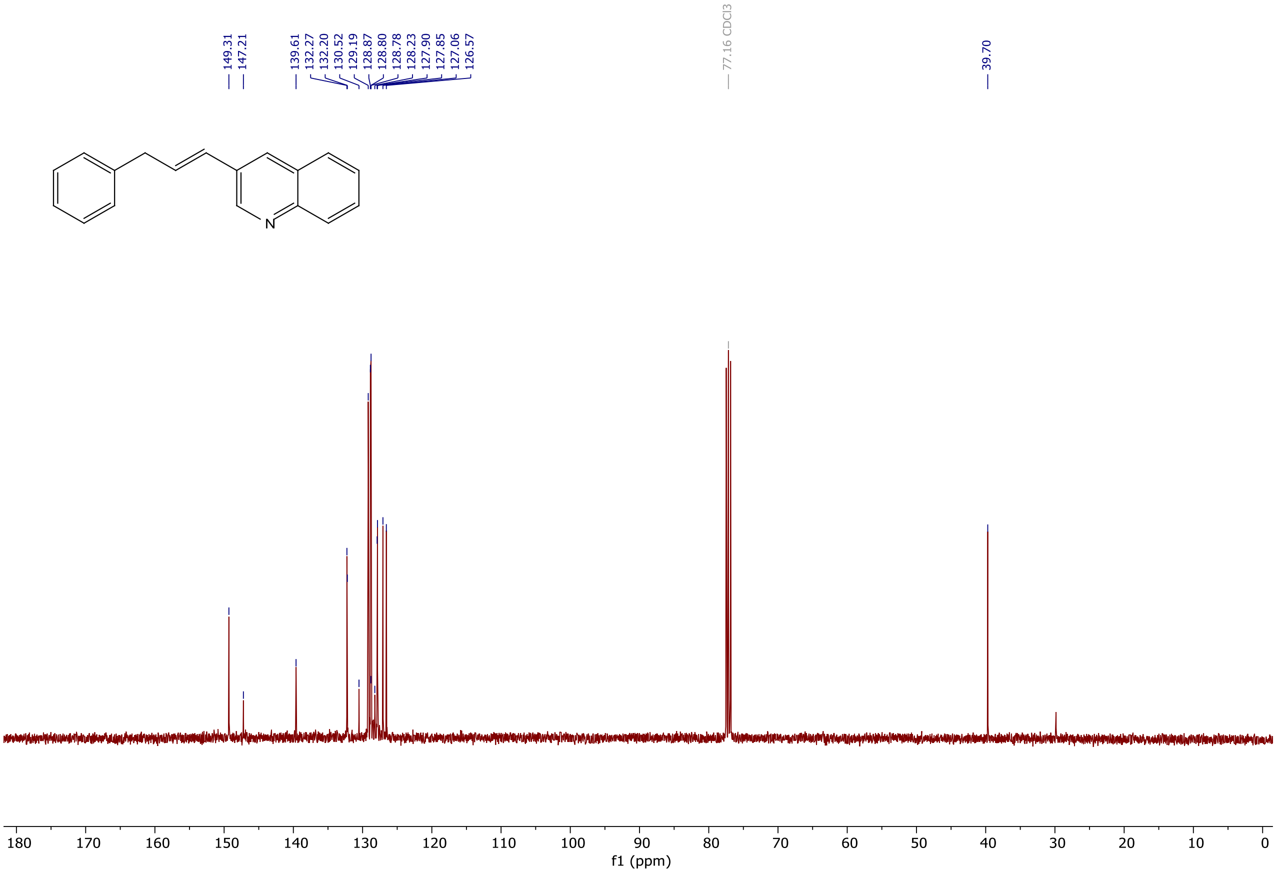

3-Cinnamylquinoline (5q'')

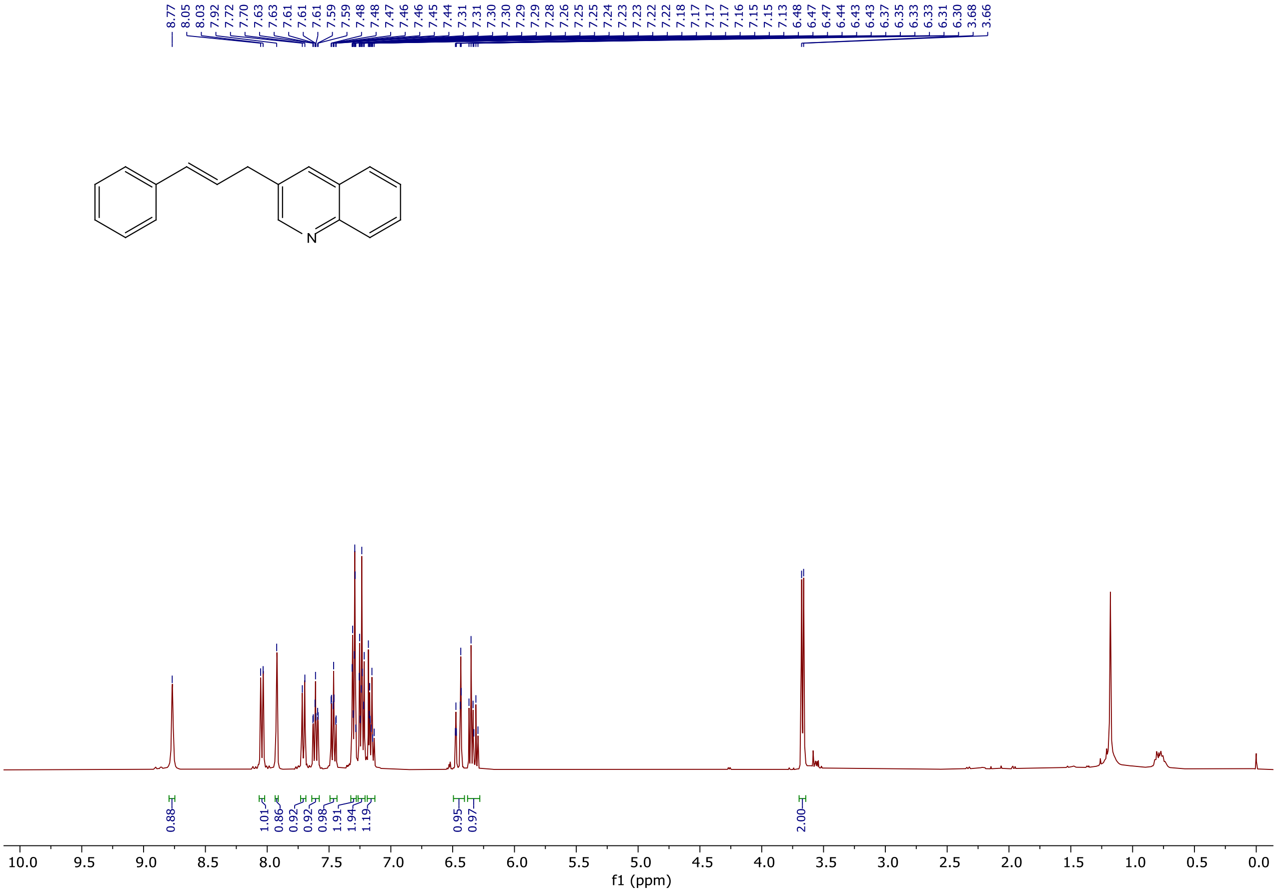

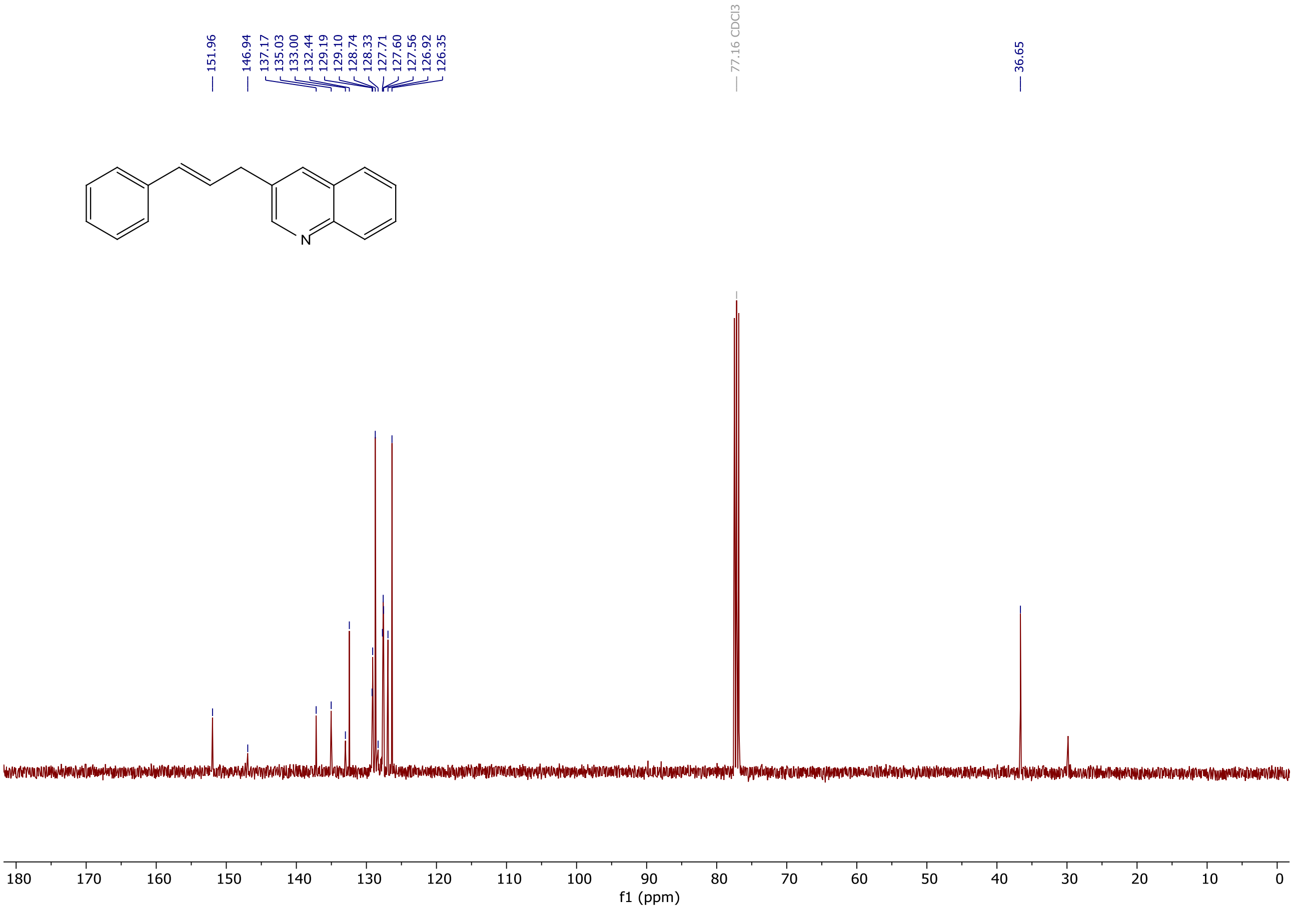

3-(4-(Methylsulfonyl)benzyl)quinoline (5r)

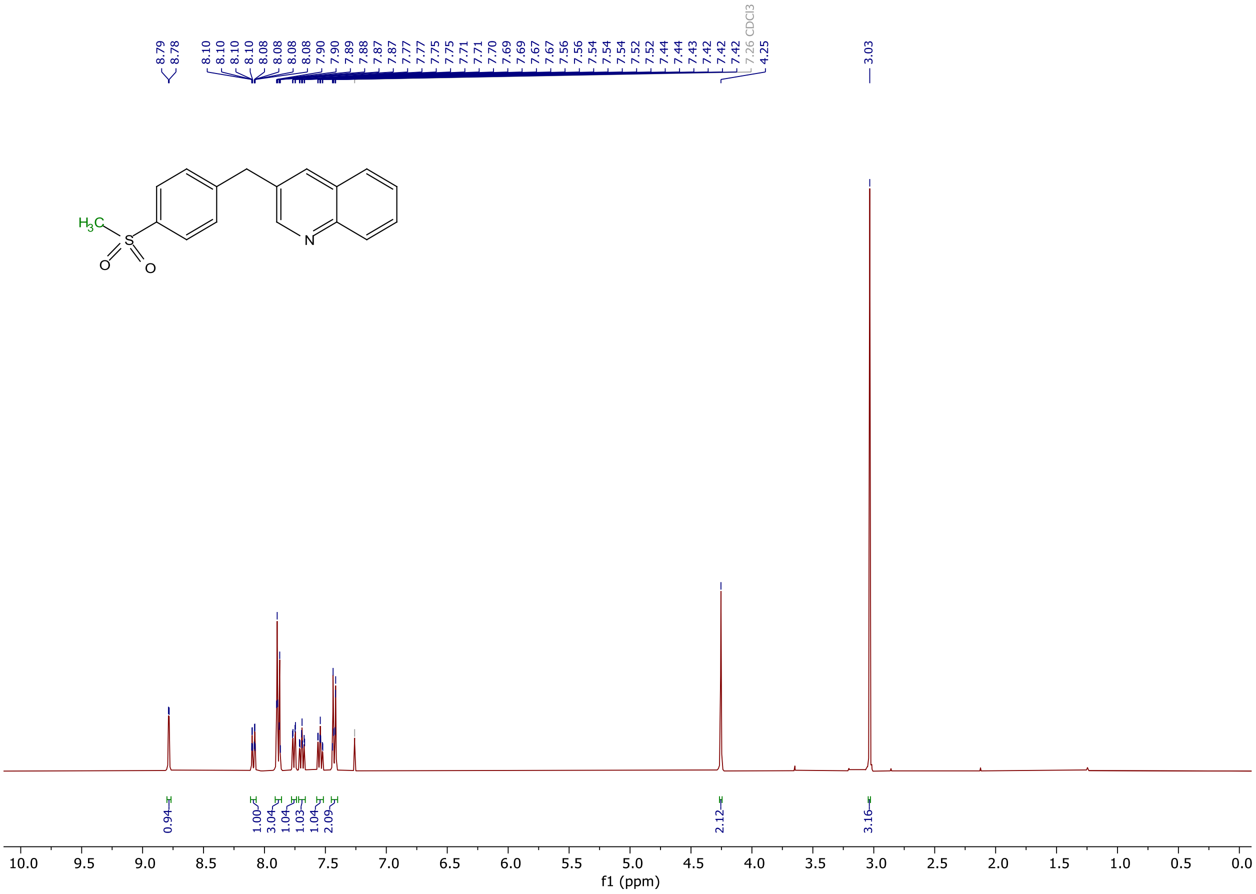

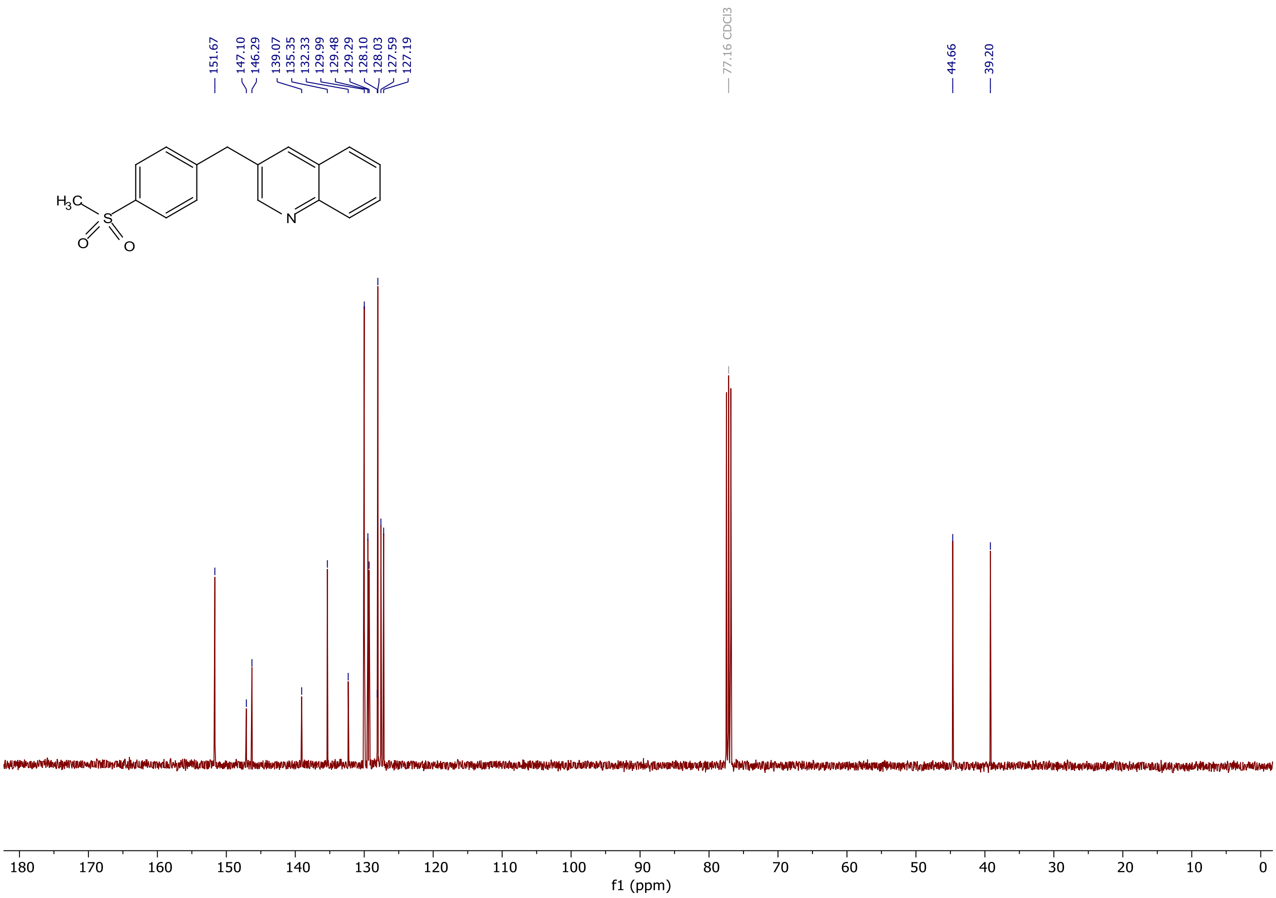

3-(3-(Pentafluorothio)benzyl)quinoline (5s)

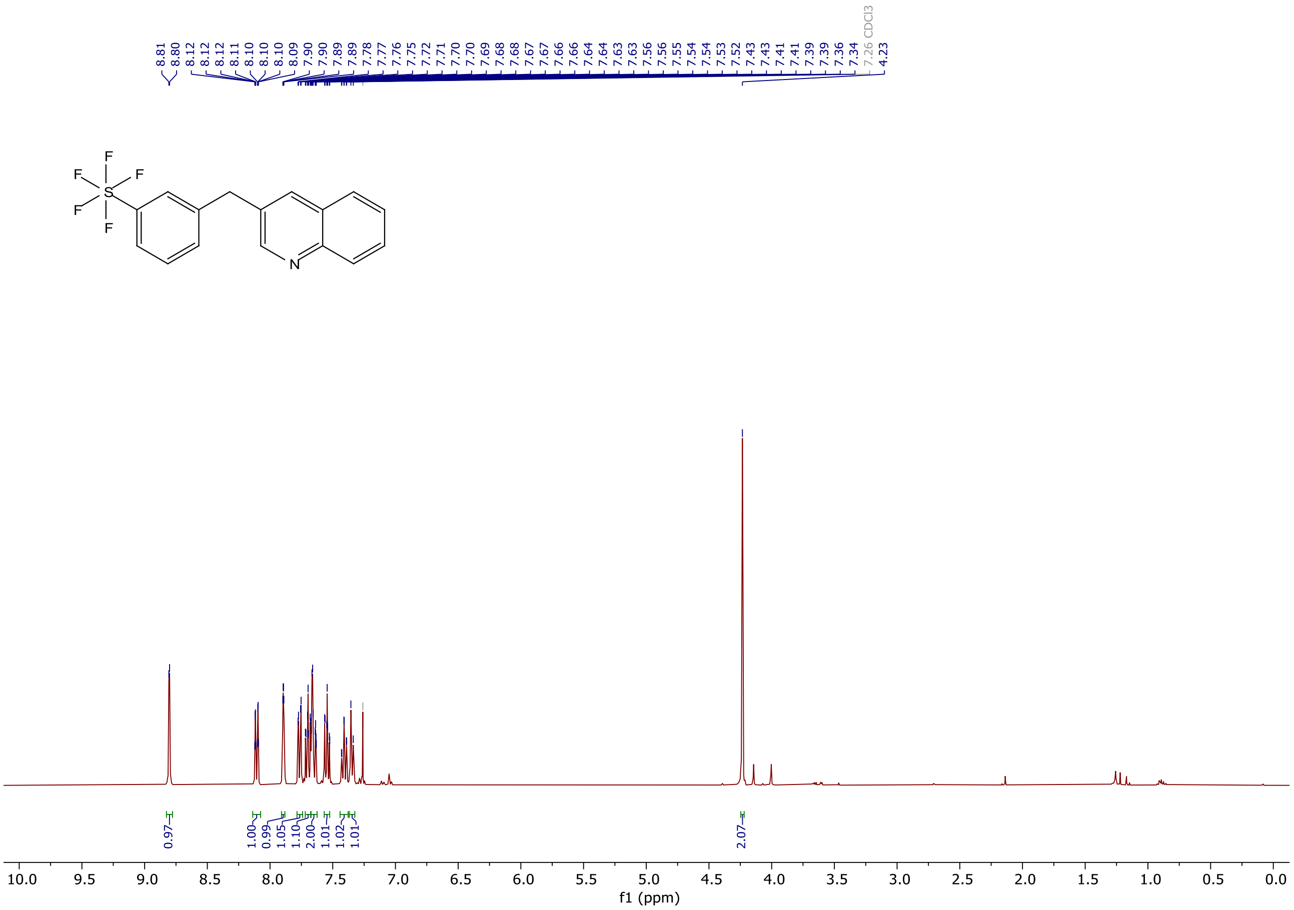

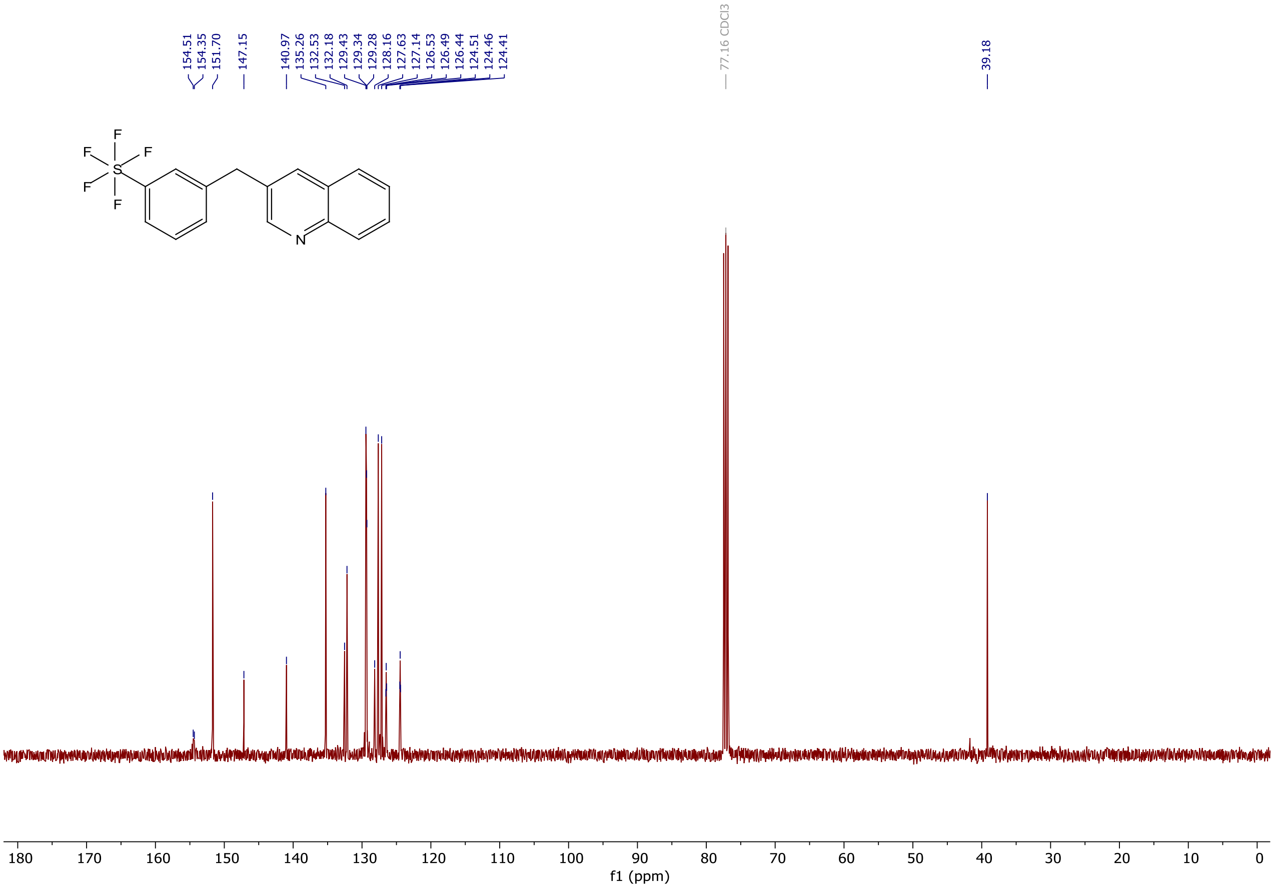

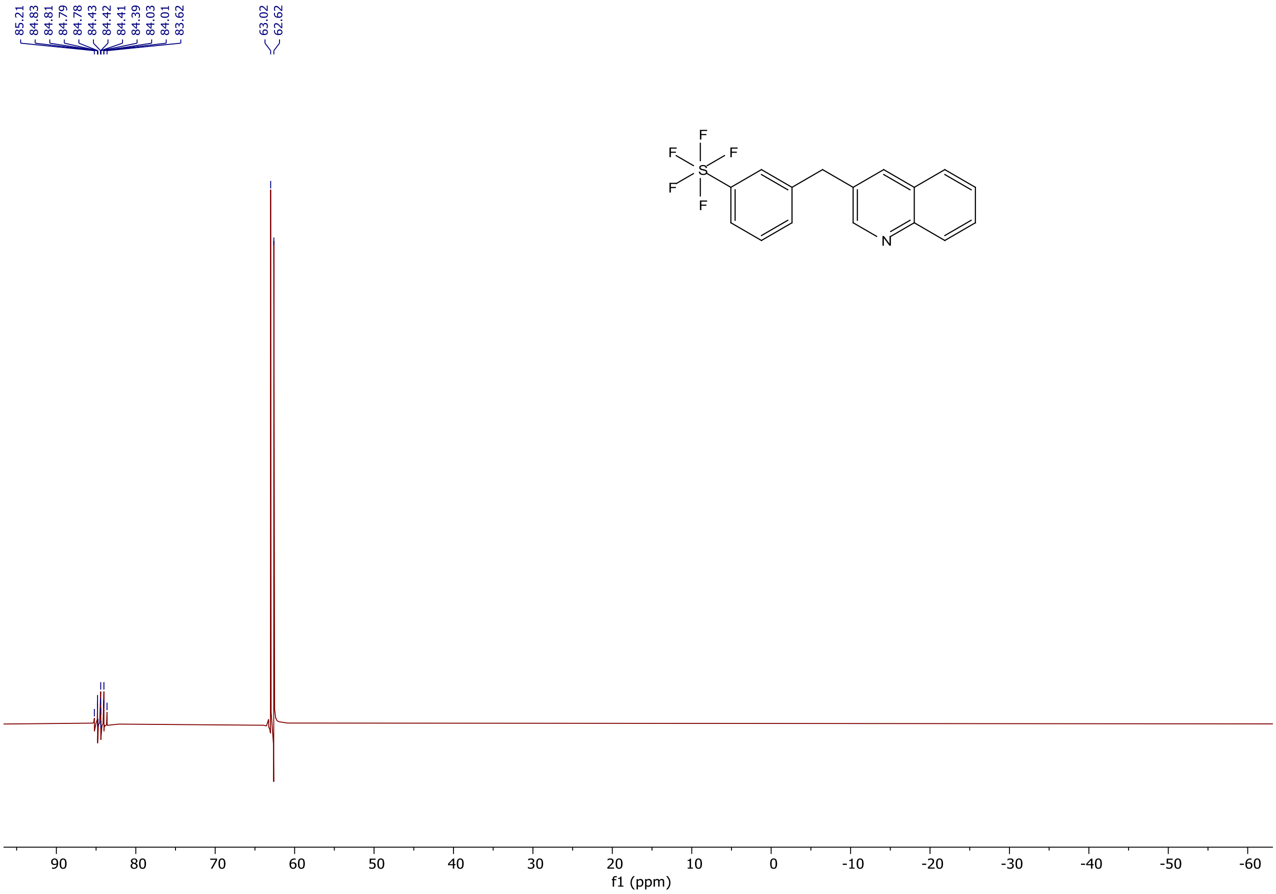
85.21
84.83
84.81
84.79
84.78
84.43
84.42
84.41
84.39
84.03
84.01
83.62
63.02
62.62
F
F
F
F
F
S
N
90
80
70
60
50
40
30
20
10
0
-10
-20
-30
-40
-50
-60
f1 (ppm)

3-(3-Methoxy-2-(trifluoromethyl)benzyl)quinoline (5t)

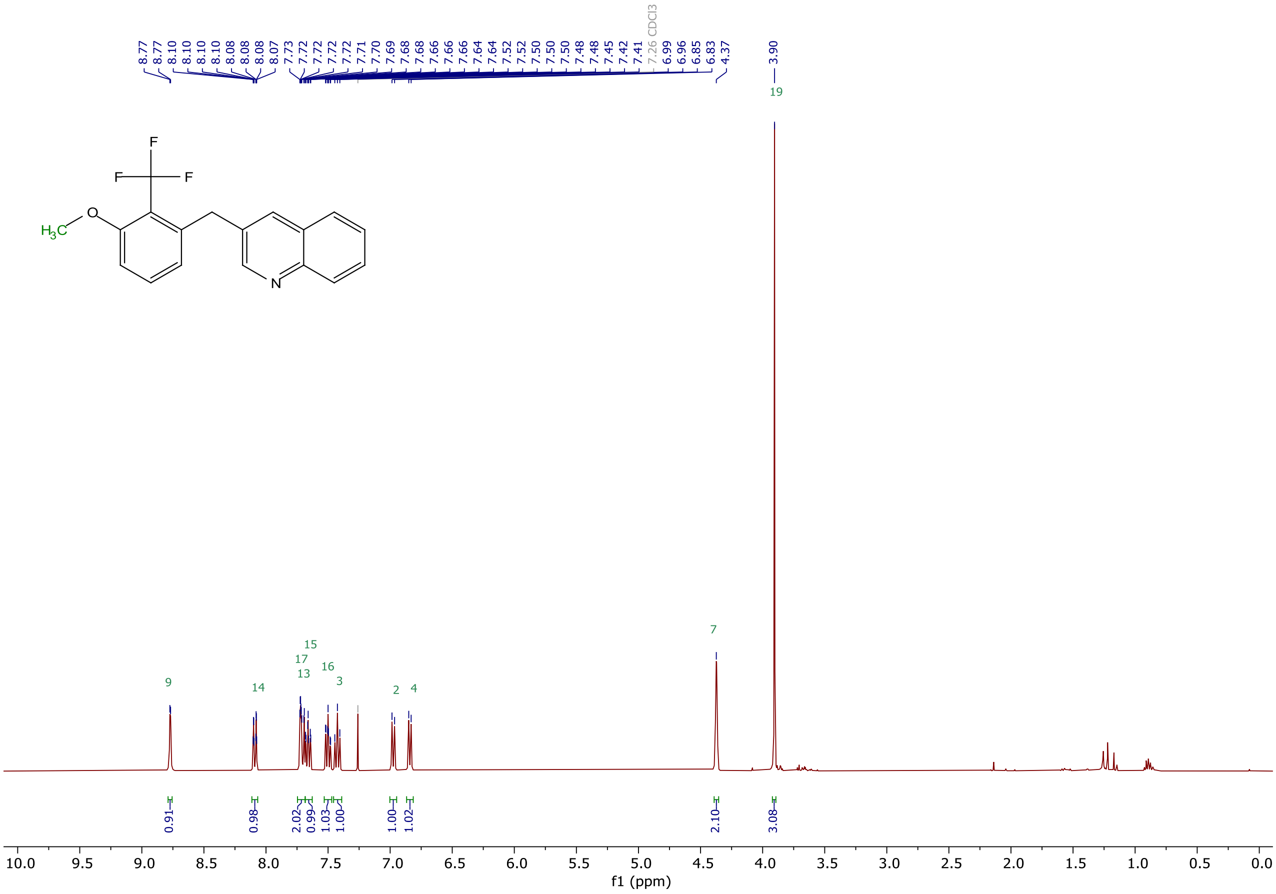

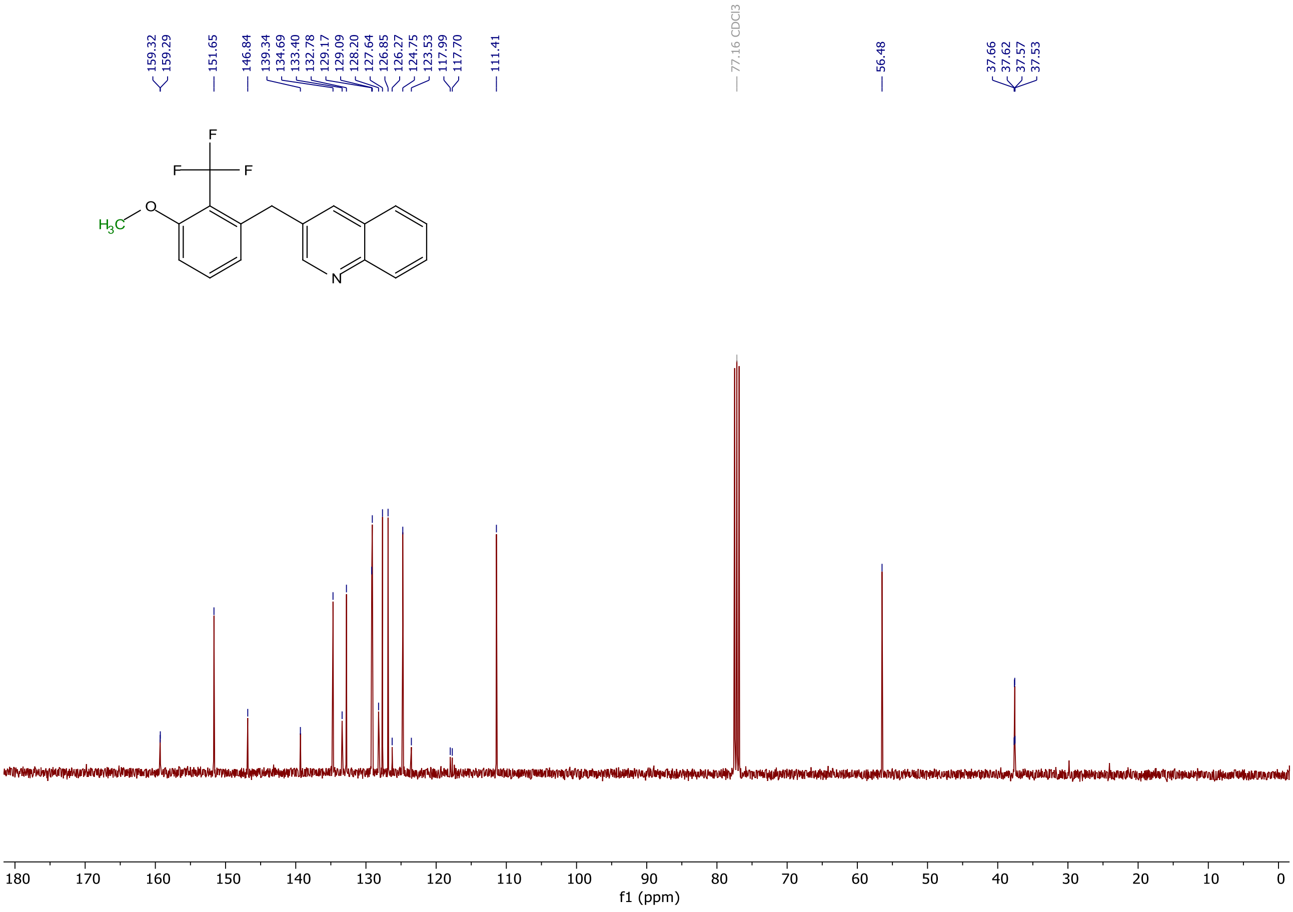

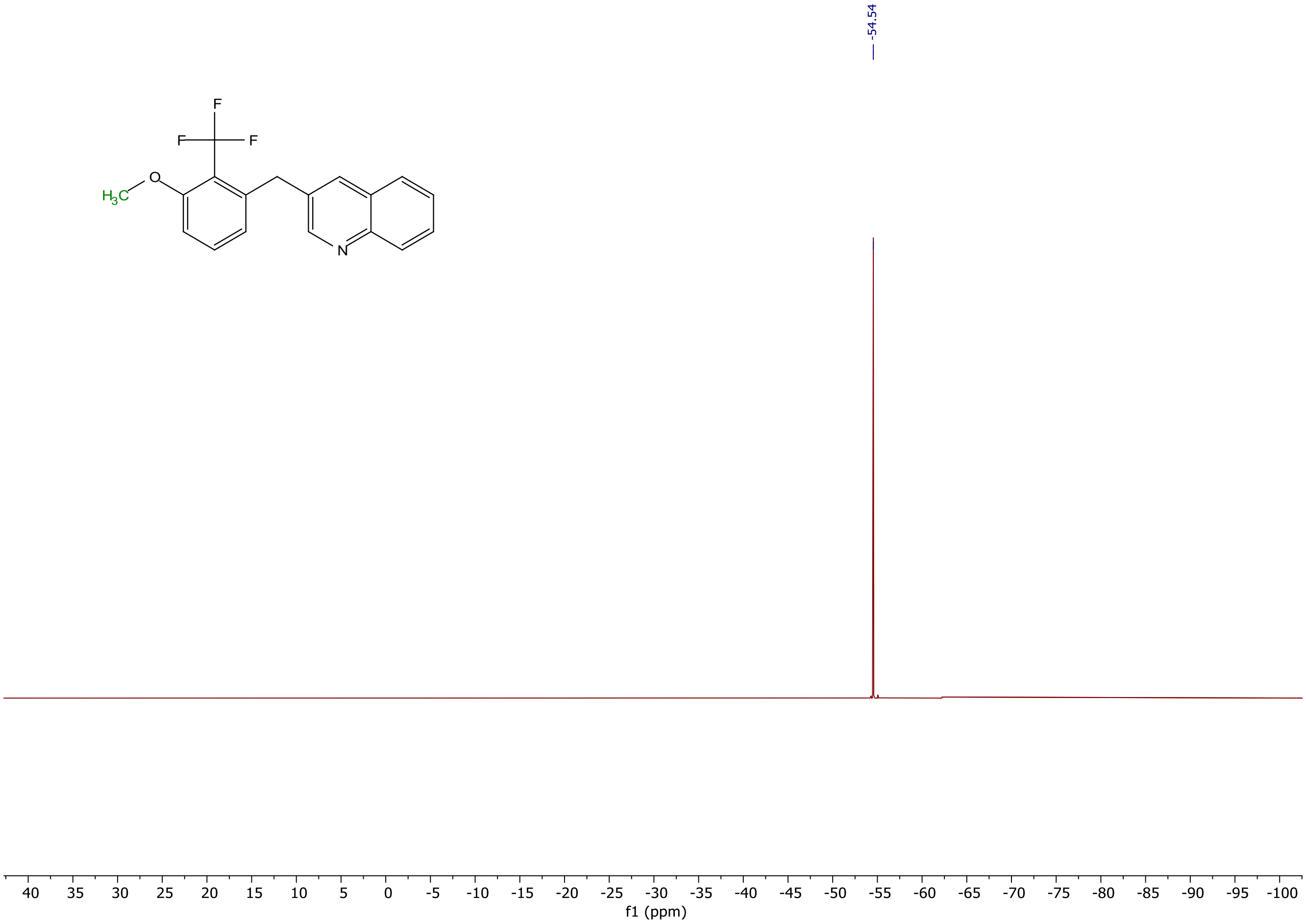
F
F
F
O
H3C
N
-54.54
40
35
30
25
20
15
10
5
0
-5
-10
-15
-20
-25
-30
-35
-40
-45
-50
-55
-60
-65
-70
-75
-80
-85
-90
-95
-100
f1 (ppm)

3-(4-Methoxybenzyl)pyridine (5u)

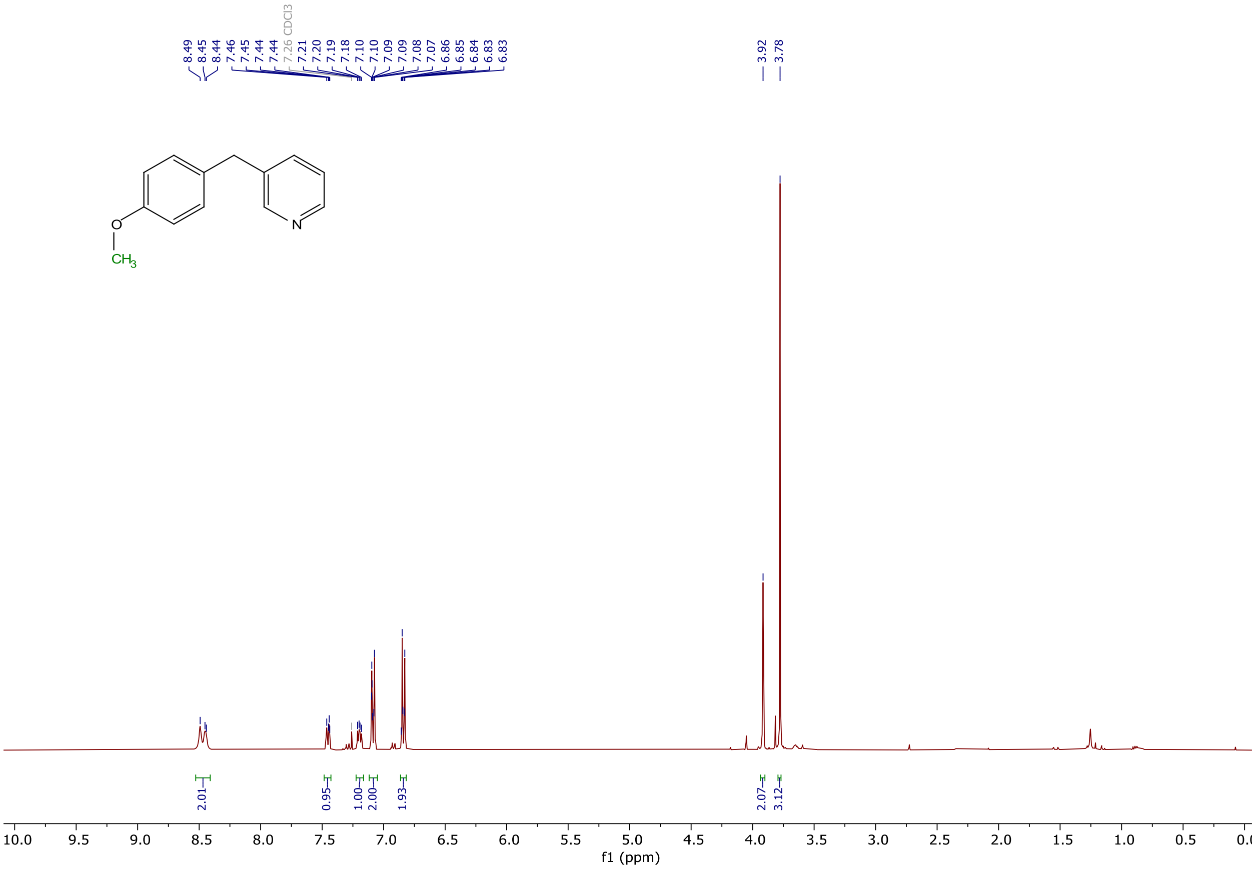

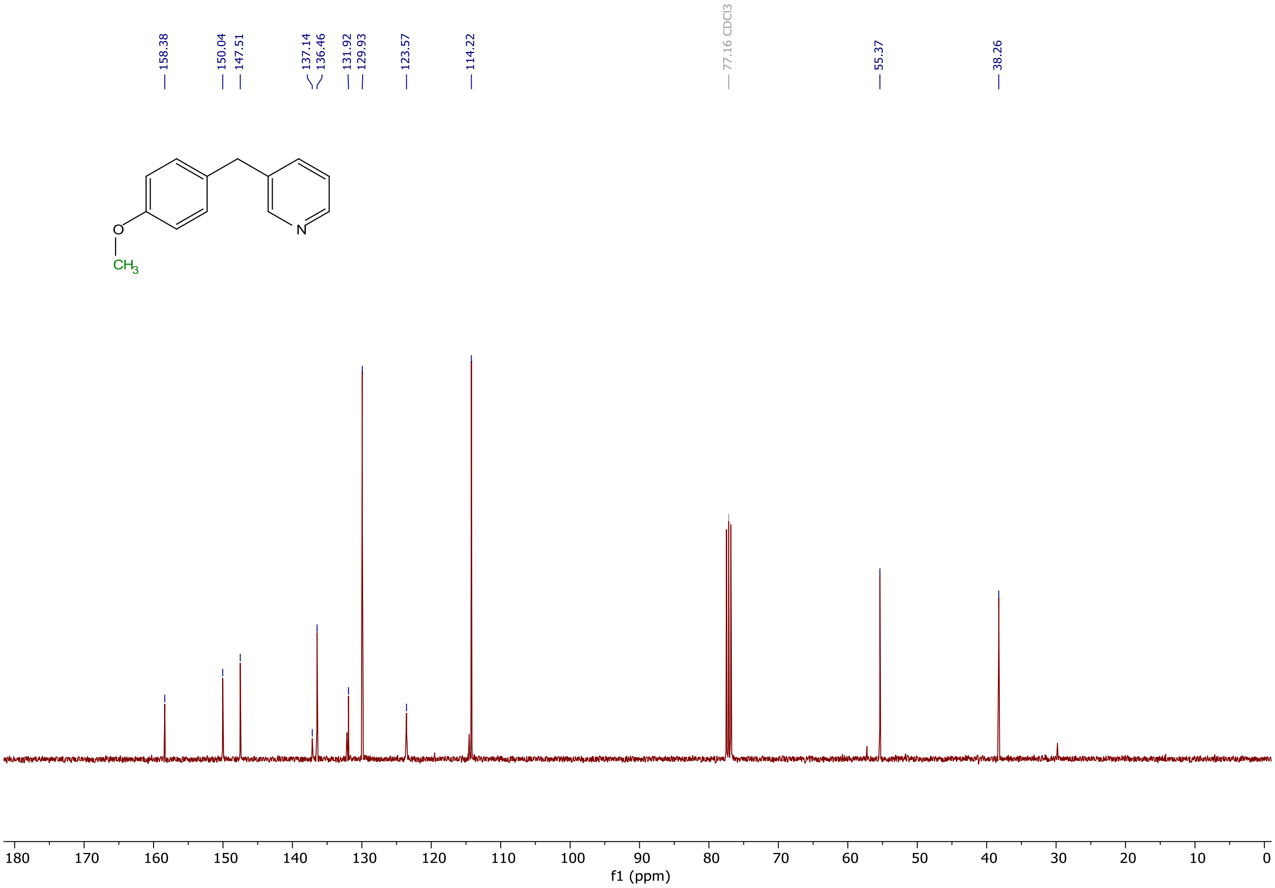

3-(4-(Trifluoromethyl)benzyl)pyridine (5v)

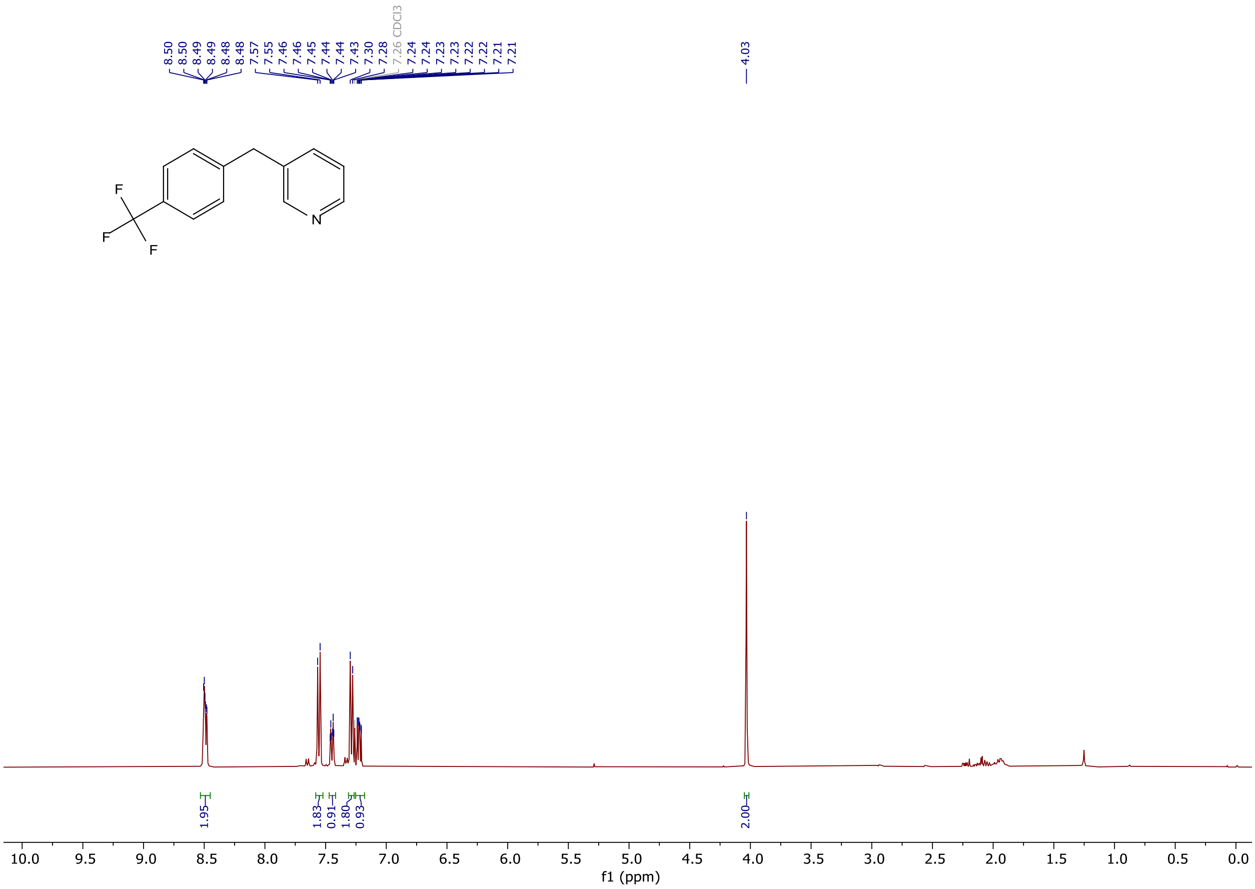

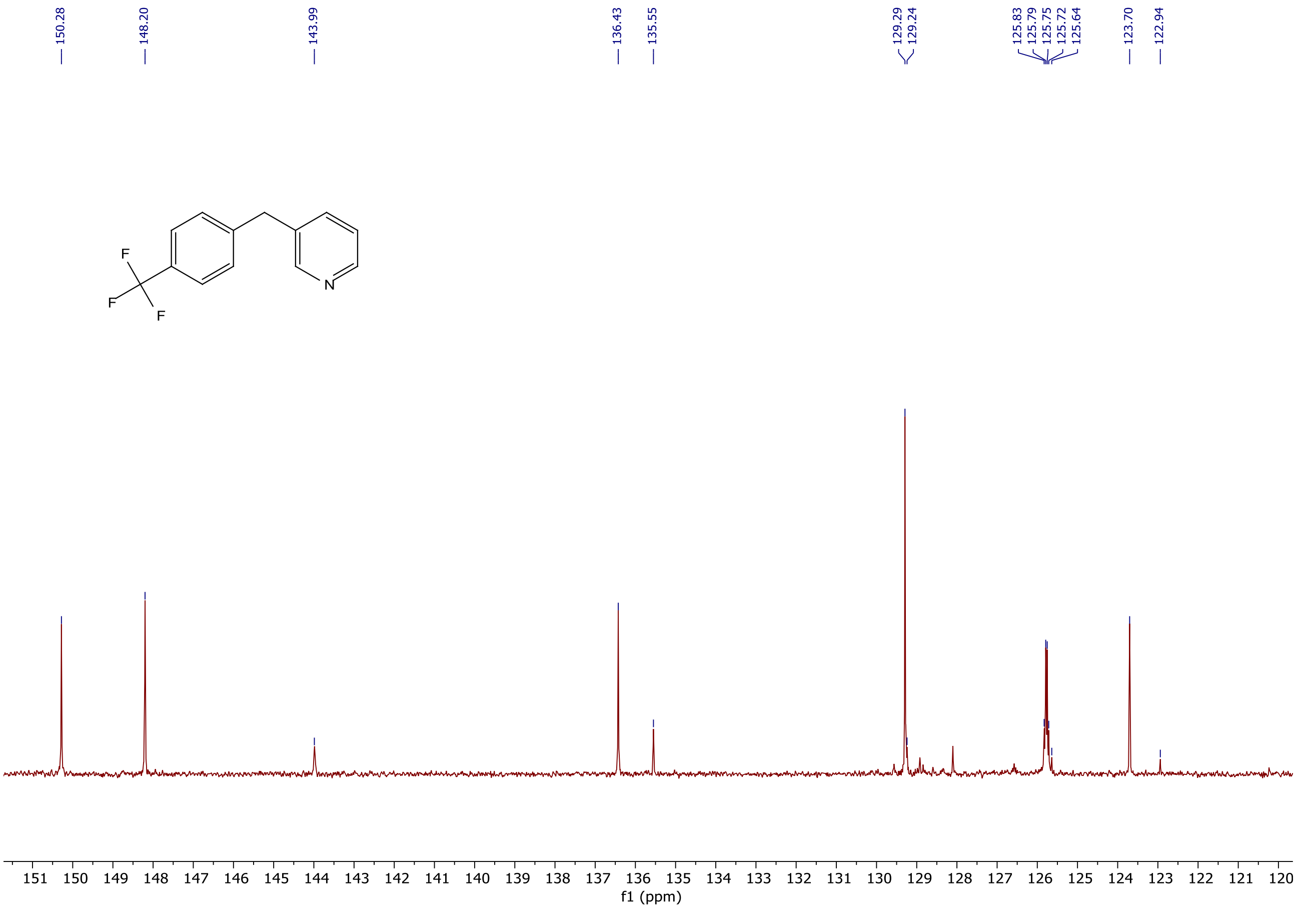

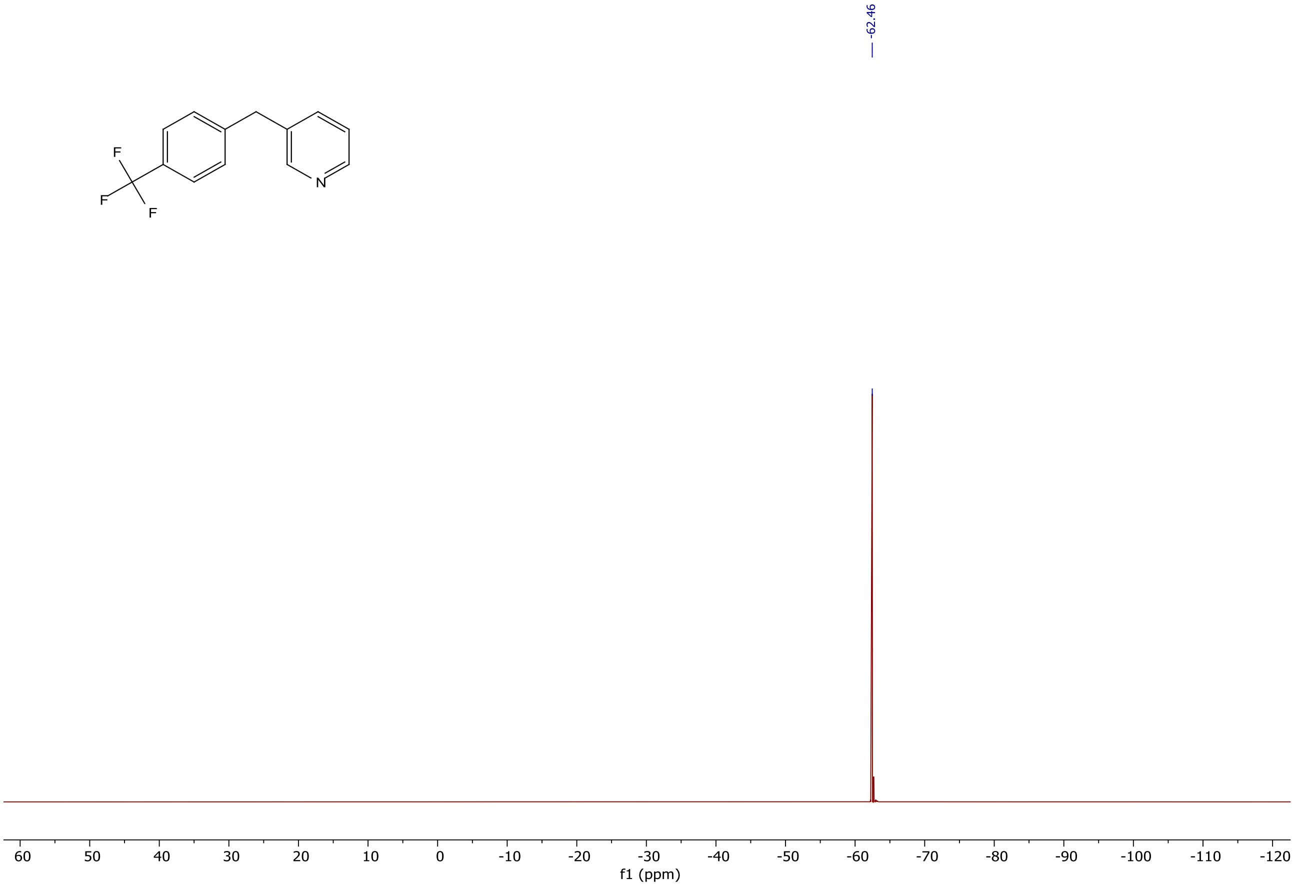
-62.46
F
F
F
N
60
50
40
30
20
10
0
-10
-20
-30
-40
-50
-60
-70
-80
-90
-100
-110
-120
f1 (ppm)

5-Benzyl-2-methoxypyridine (5w)

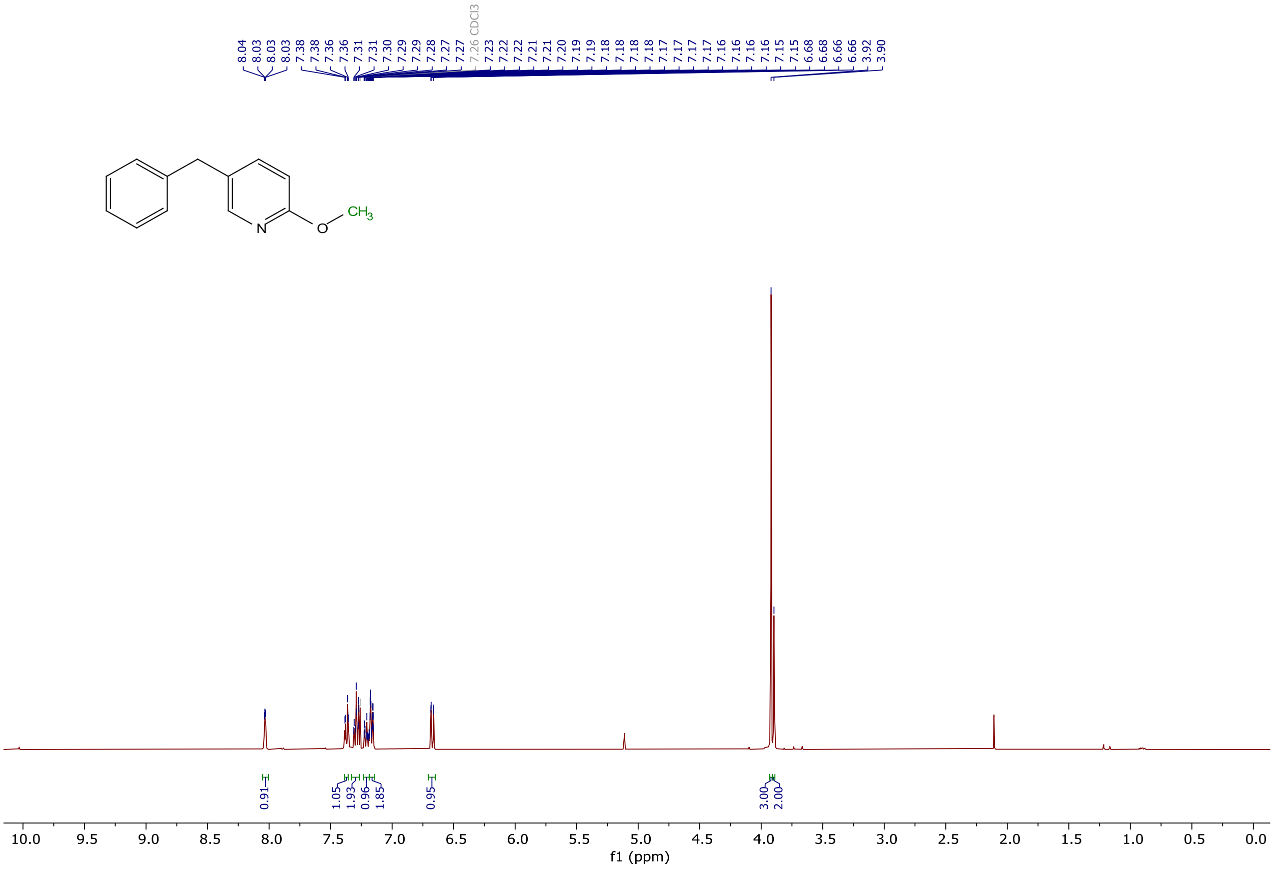

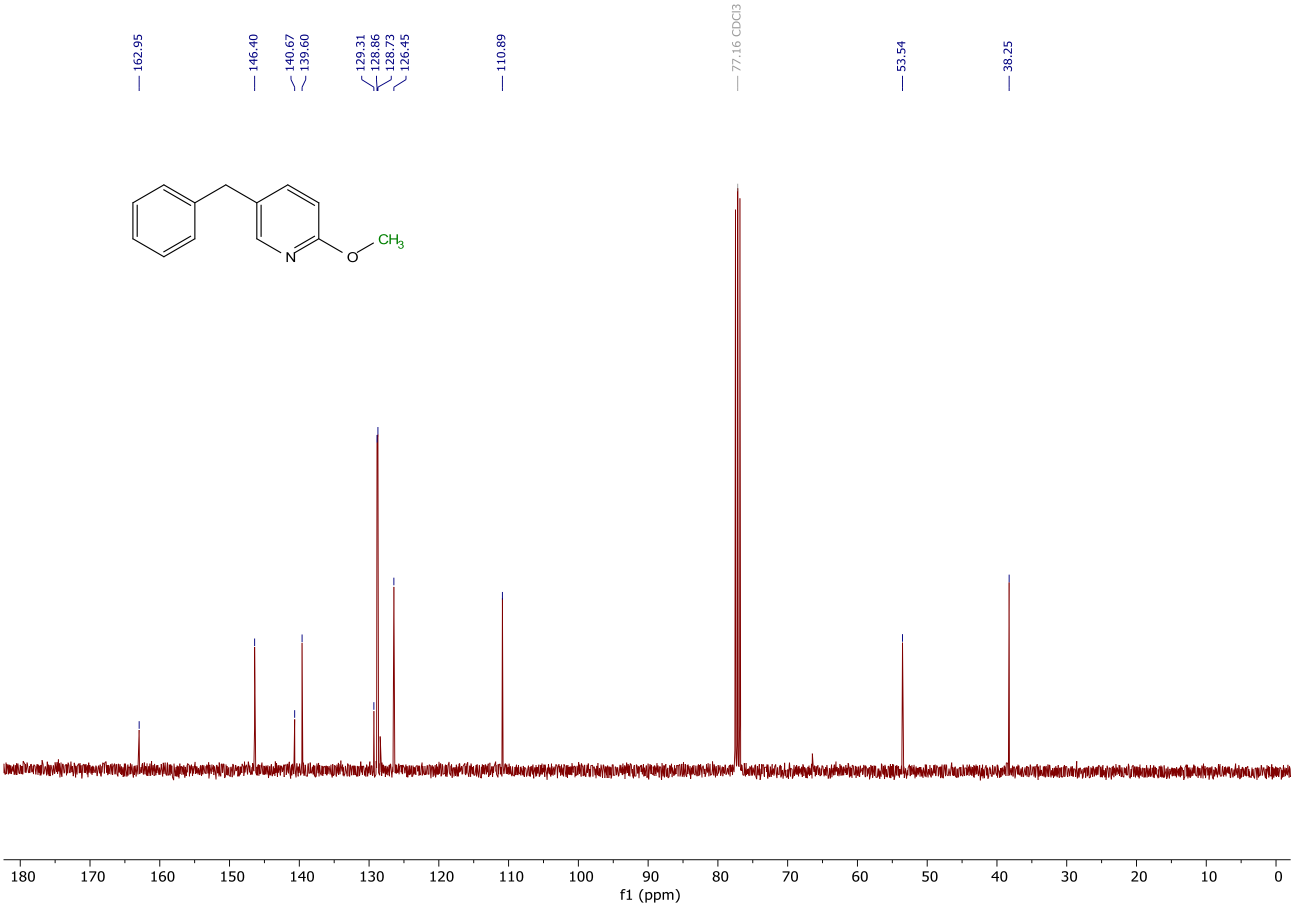

4-(2,6-Difluorobenzyl)-2-methoxypyridine (5x)

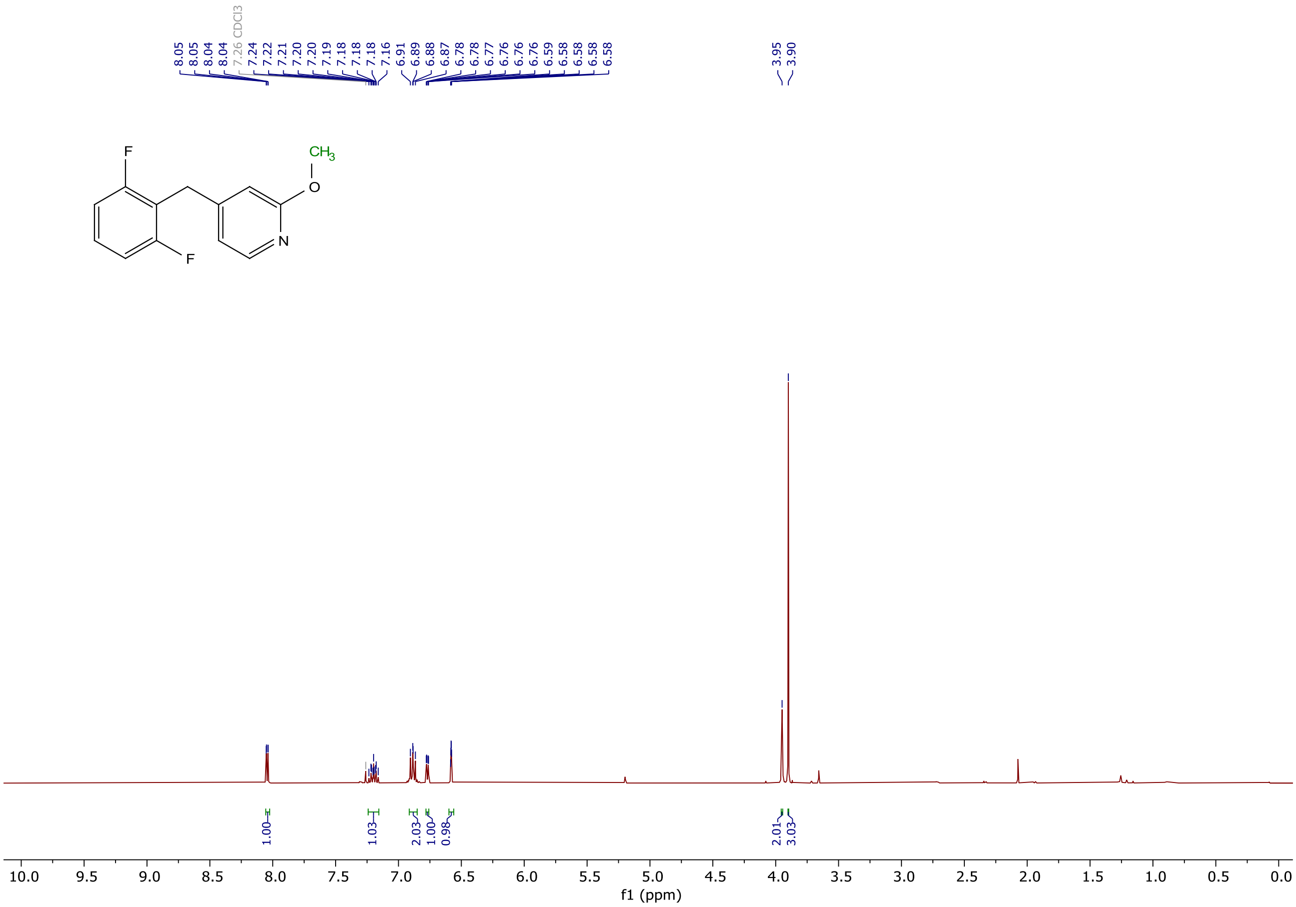

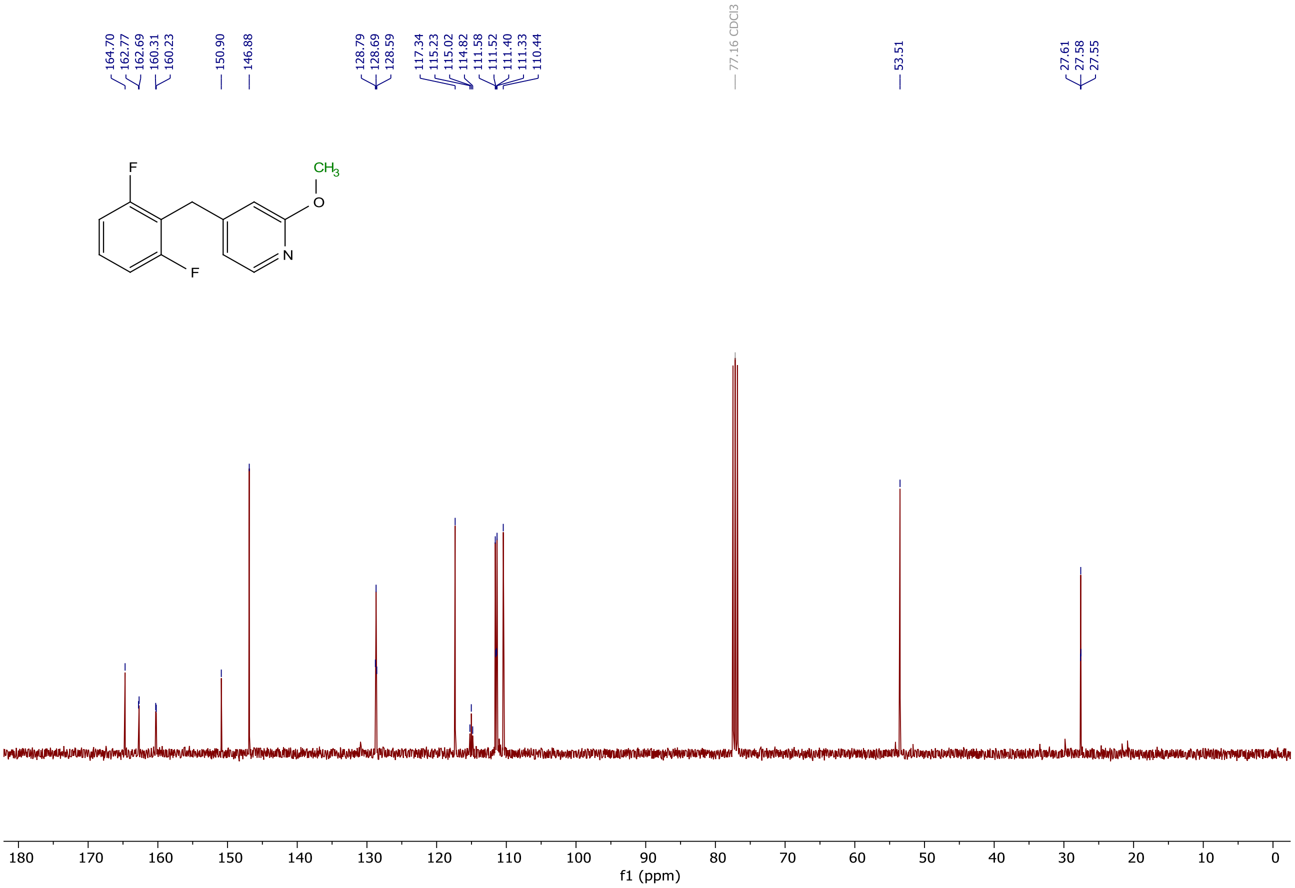

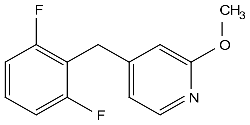
F
CH3
O
N
F

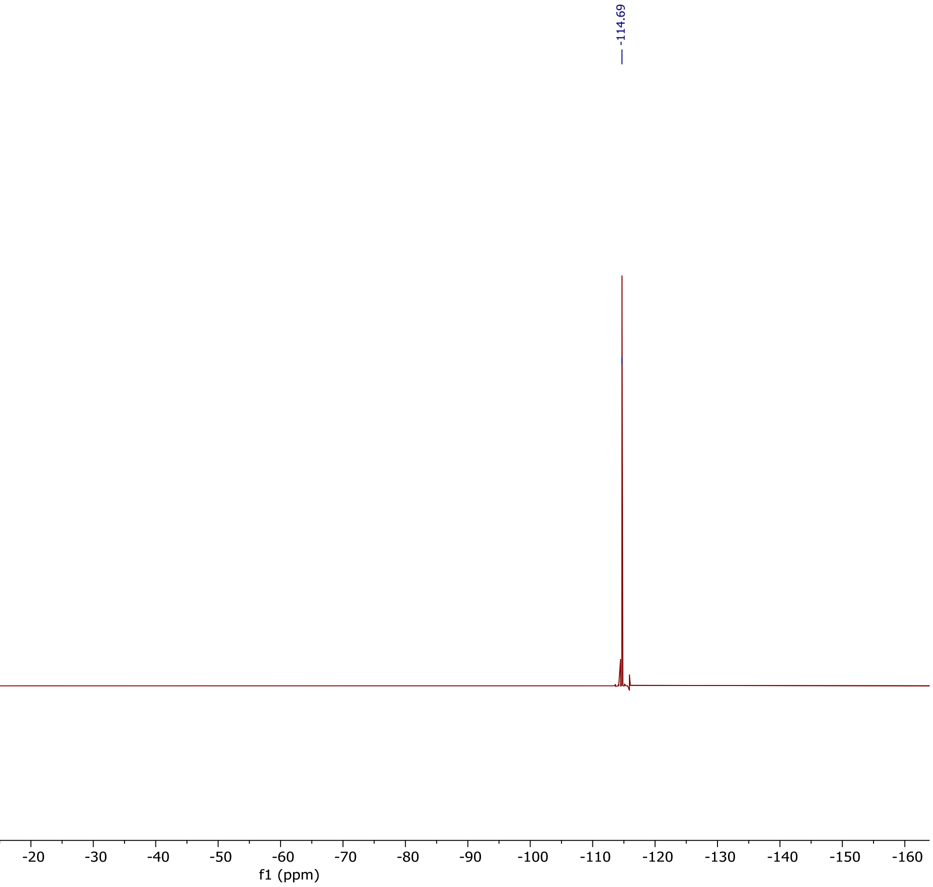
-114.69
40 30 20 10 0 -10 -20 -30 -40 -50 -60 -70 -80 -90 -100 -110 -120 -130 -140 -150 -160
f1 (ppm)

5-Benzylpyridin-3-ol (5y)

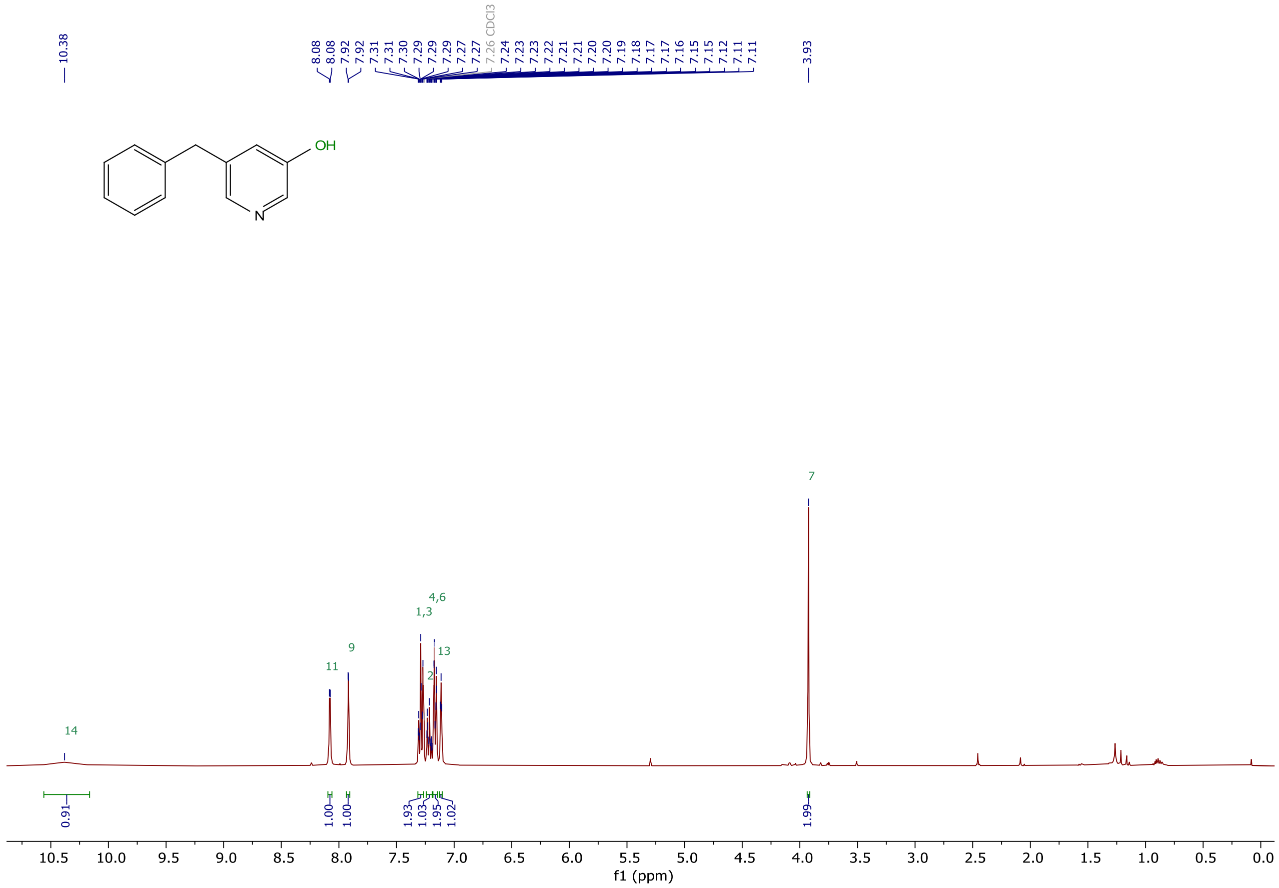

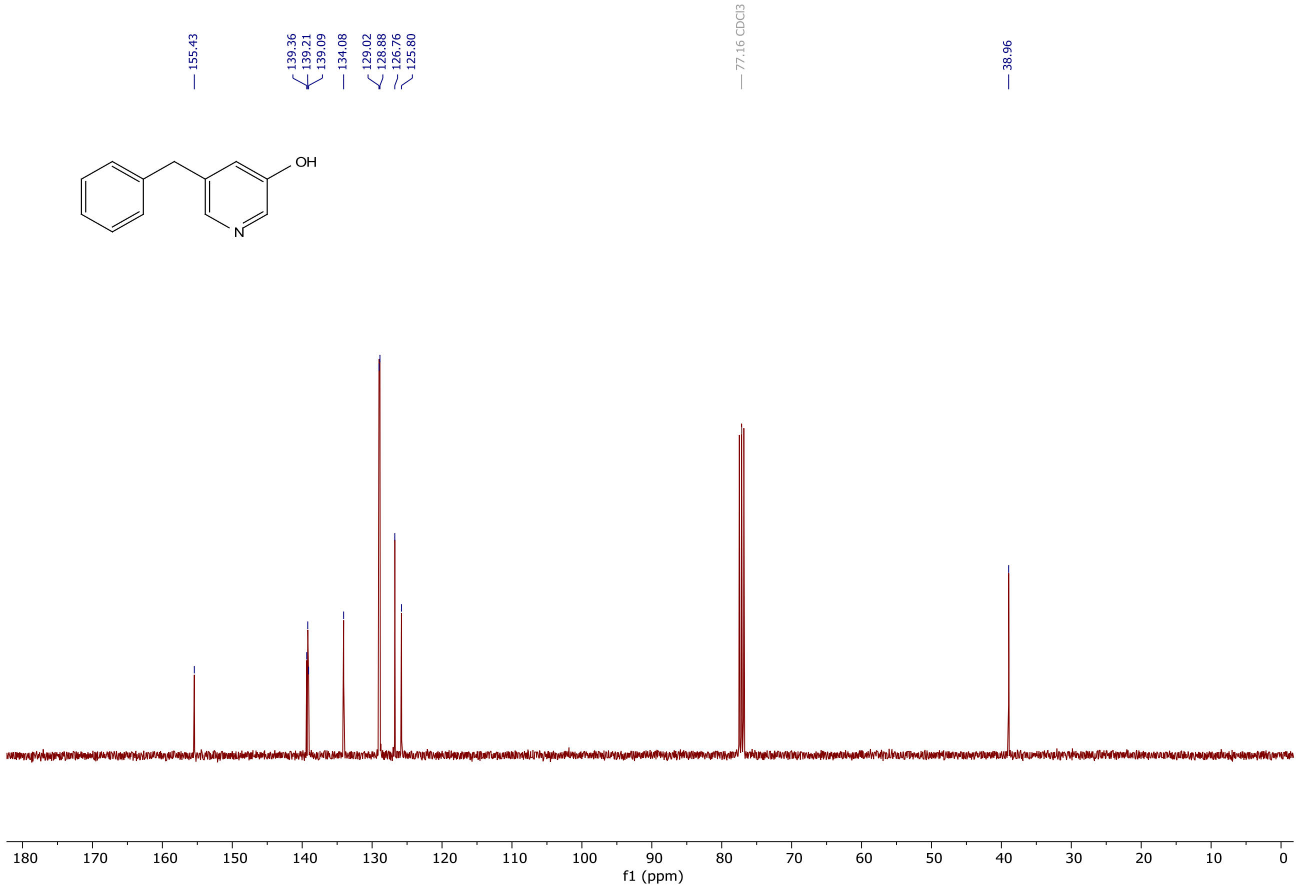

Tert-butyl 4-(5-(3-cyanobenzyl)pyridin-2-yl)piperazine-1-carboxylate (5z)

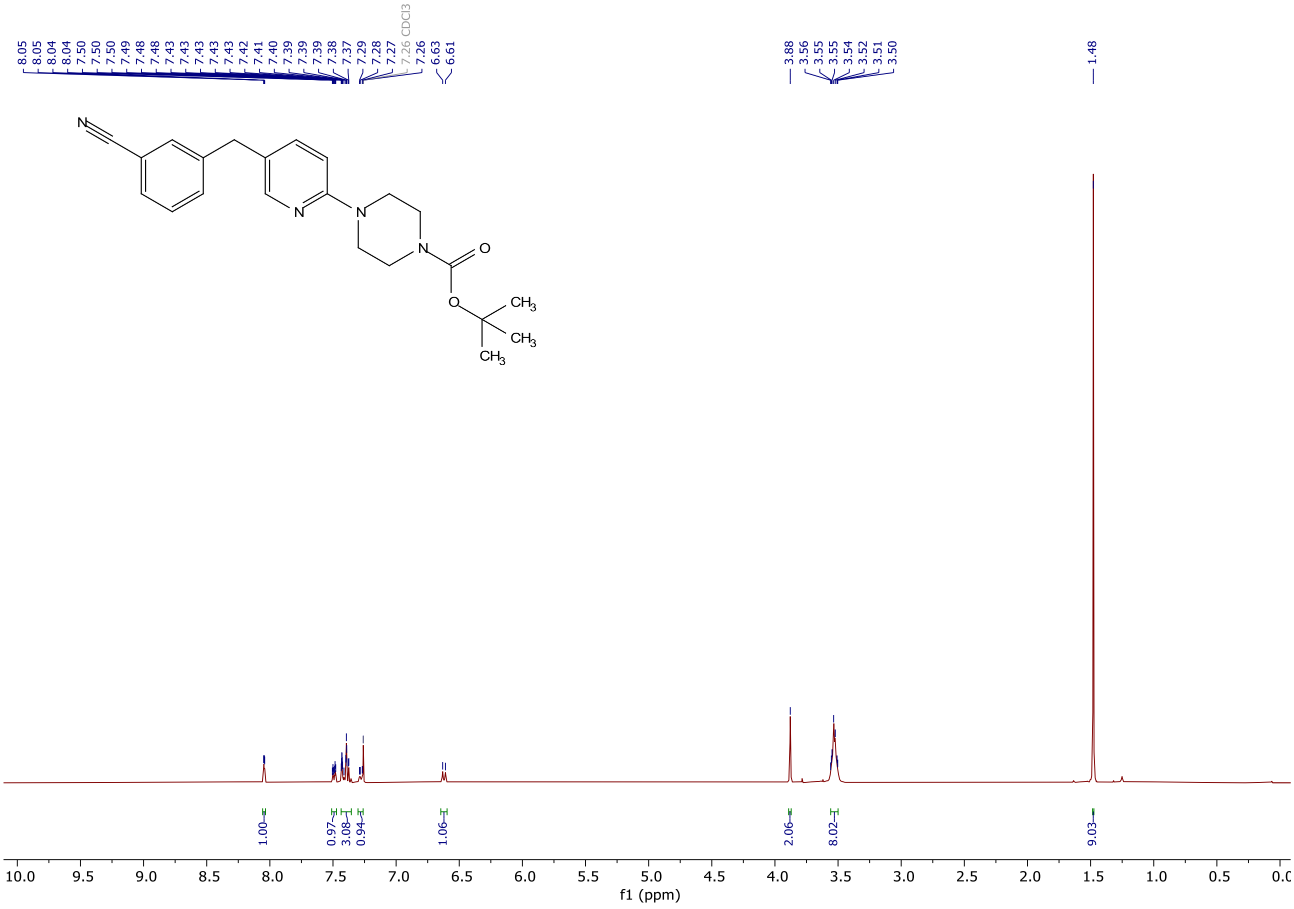

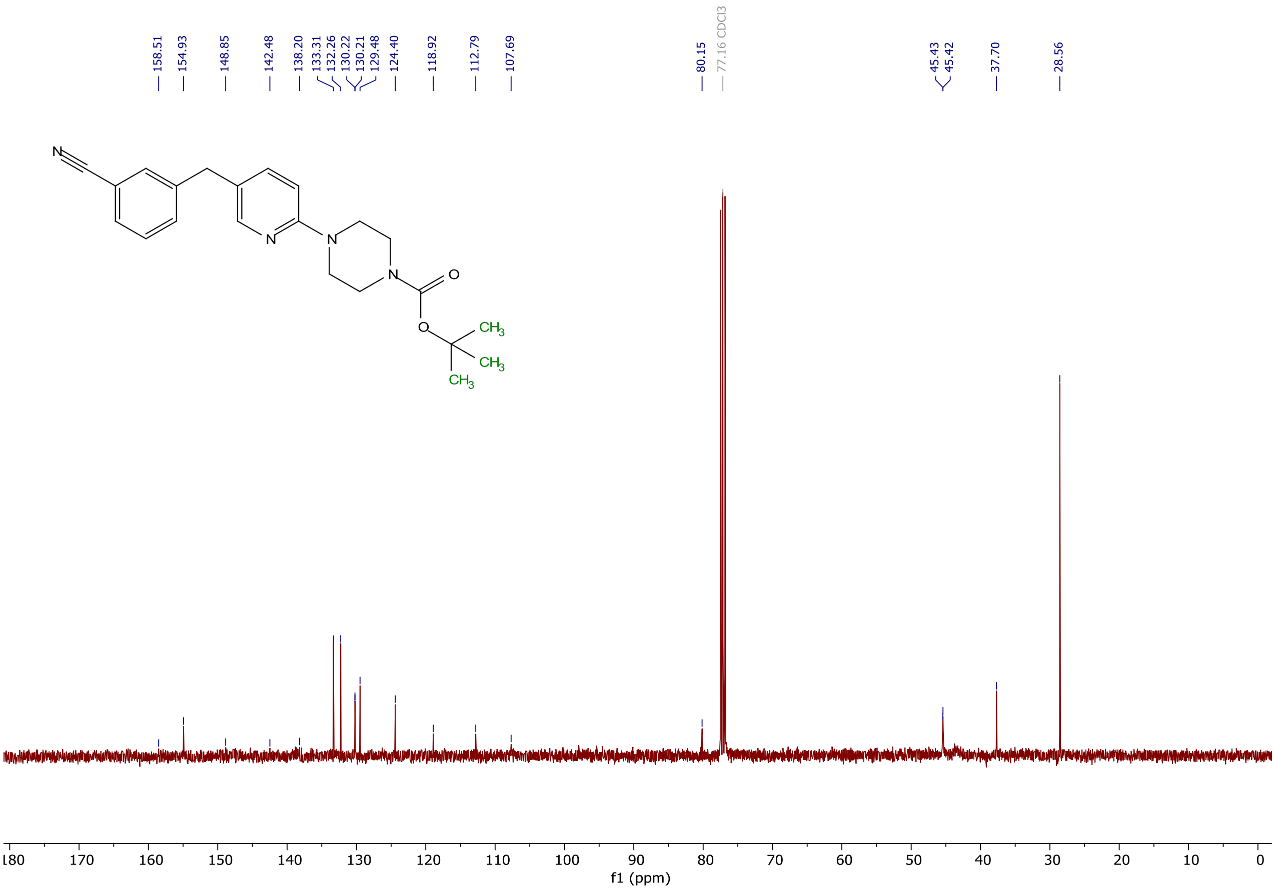

3-((2-Methylpyridin-3-yl)methyl)benzonitrile (5aa)

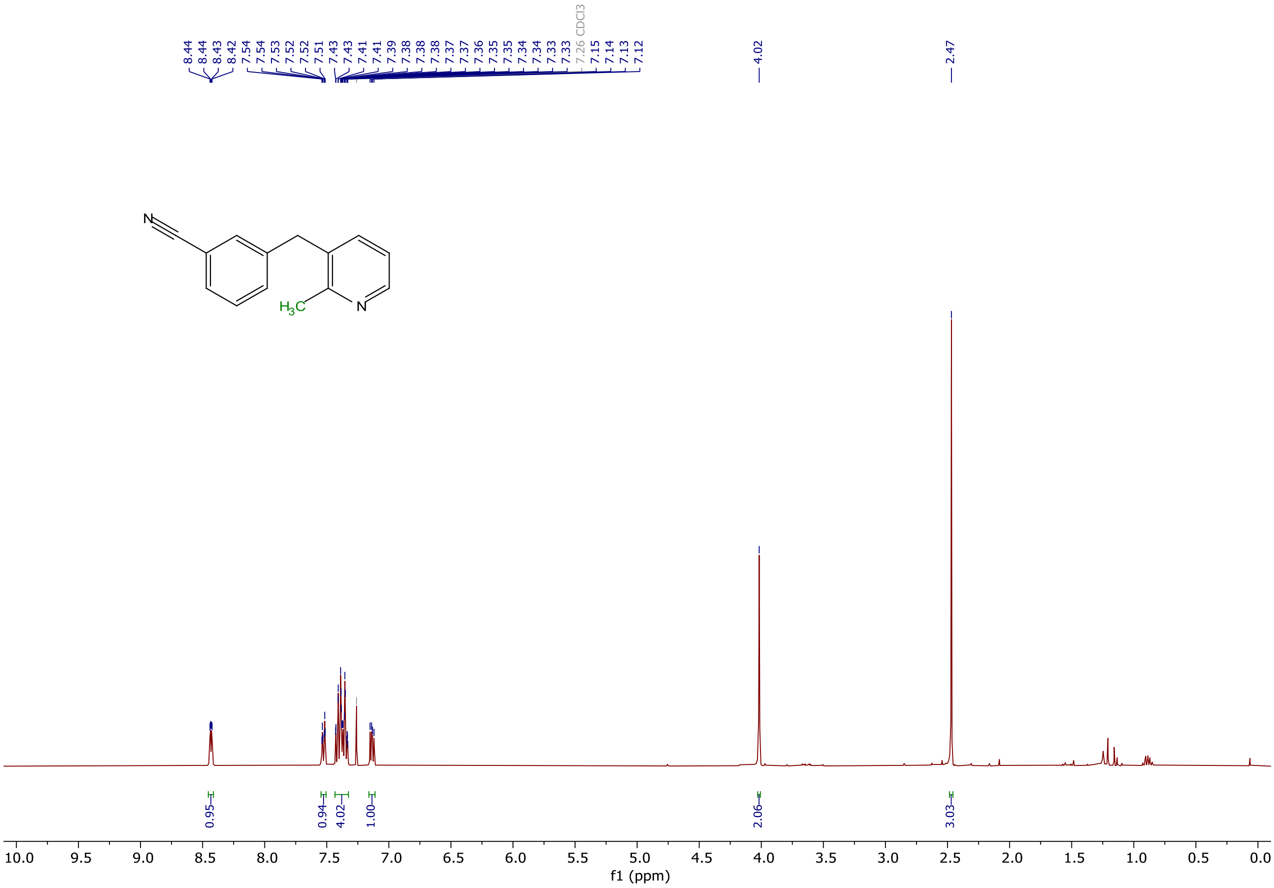

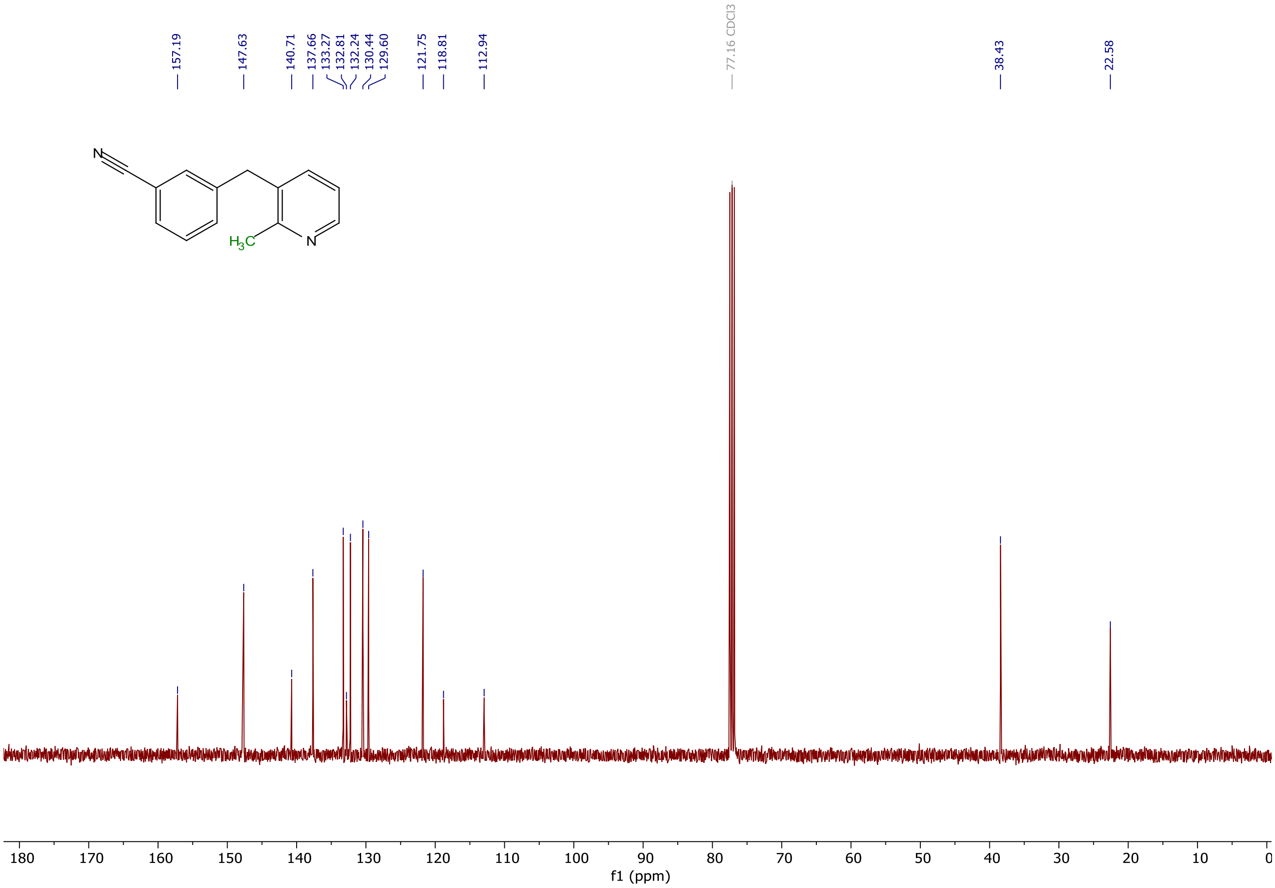

3-((3-Methylpyridin-2-yl)methyl)benzonitrile (5ab)

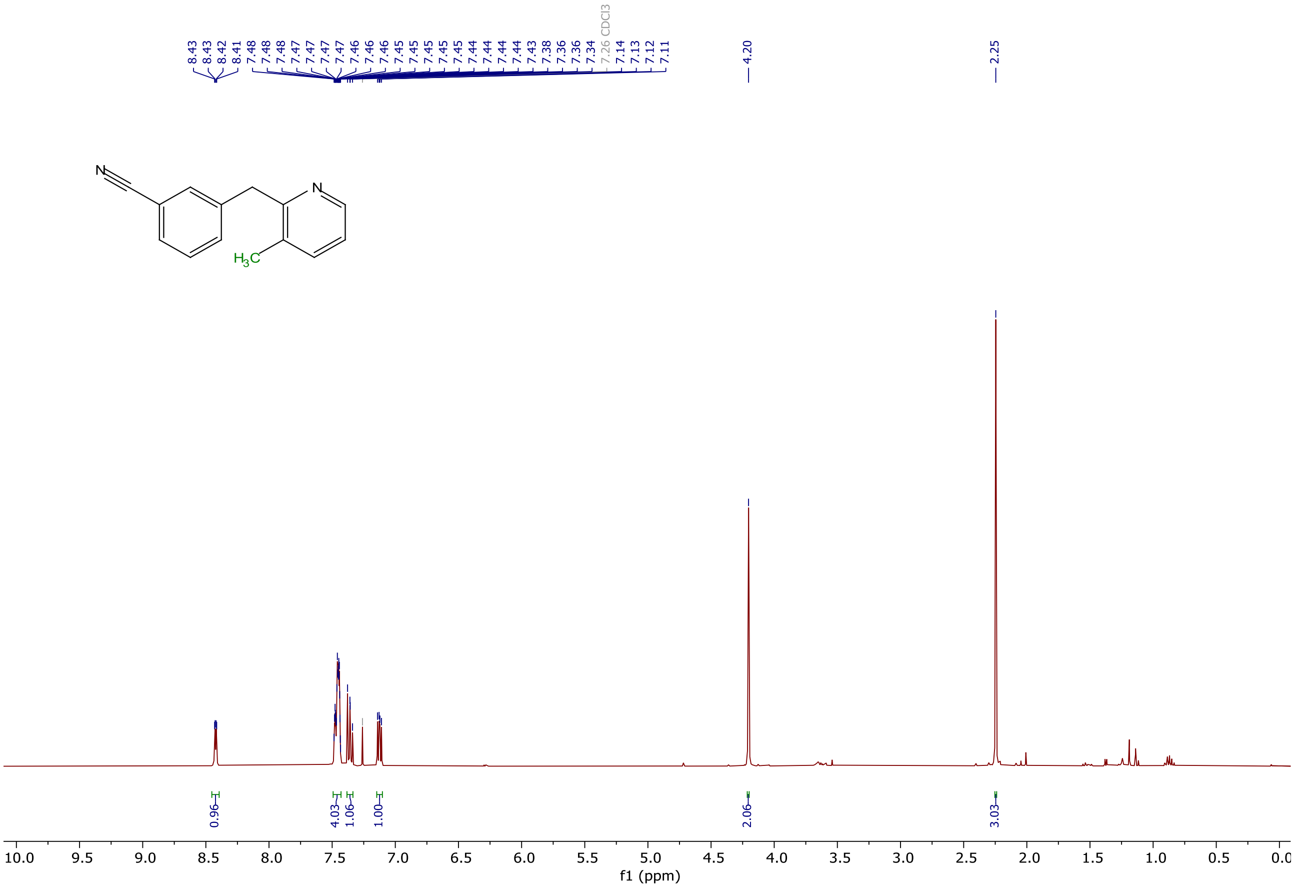

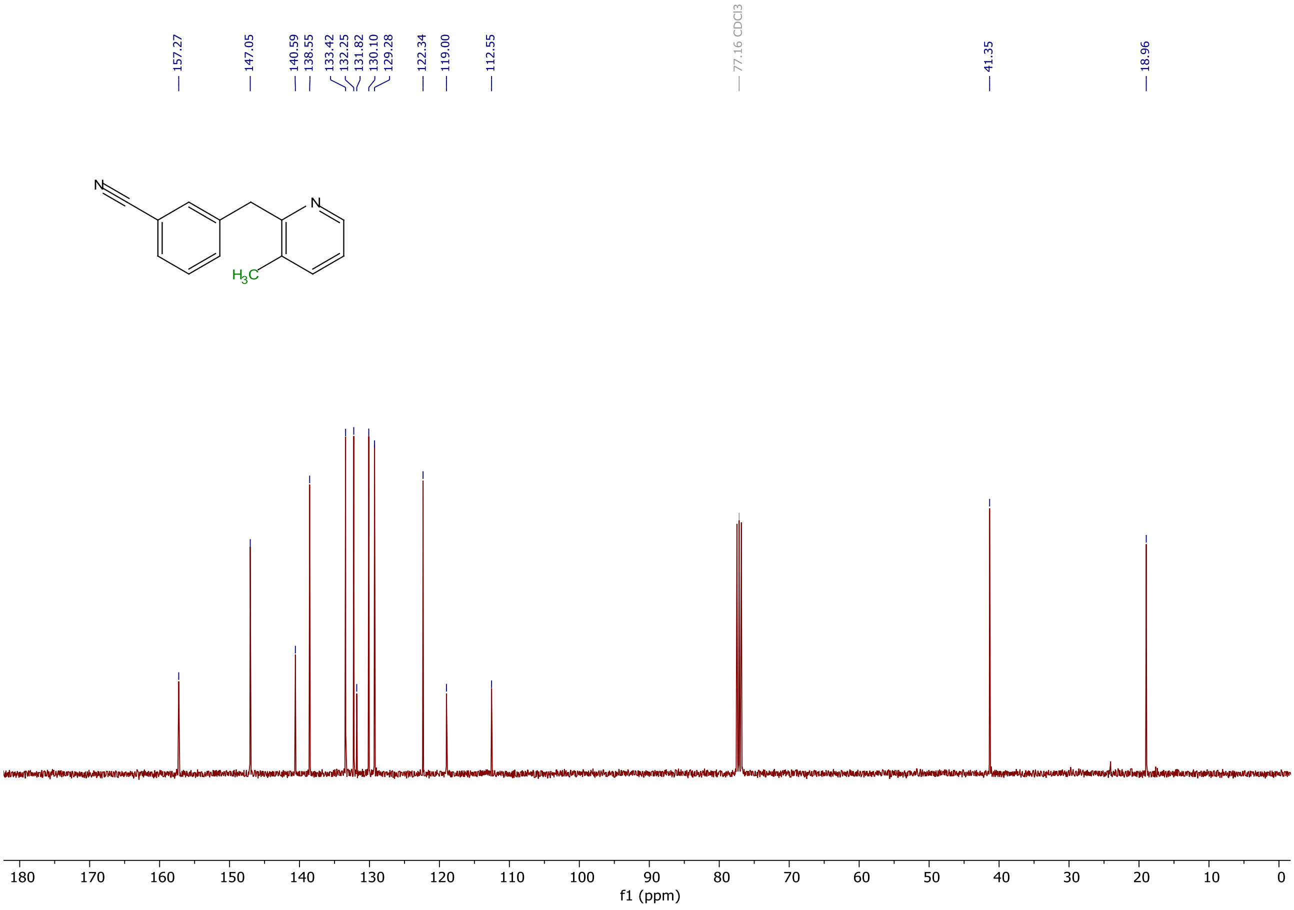

3-((6-Methylpyridin-2-yl)methyl)benzonitrile (5ac)

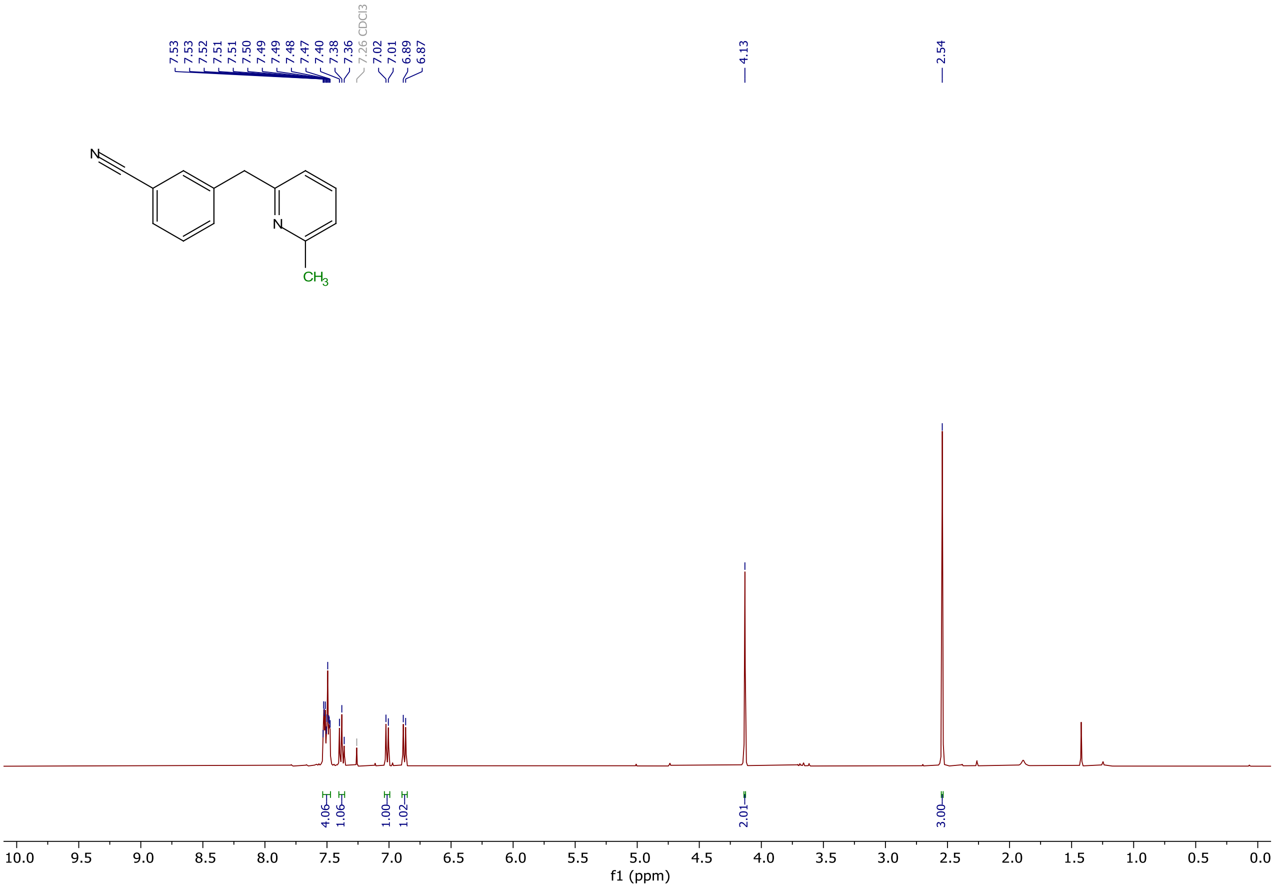

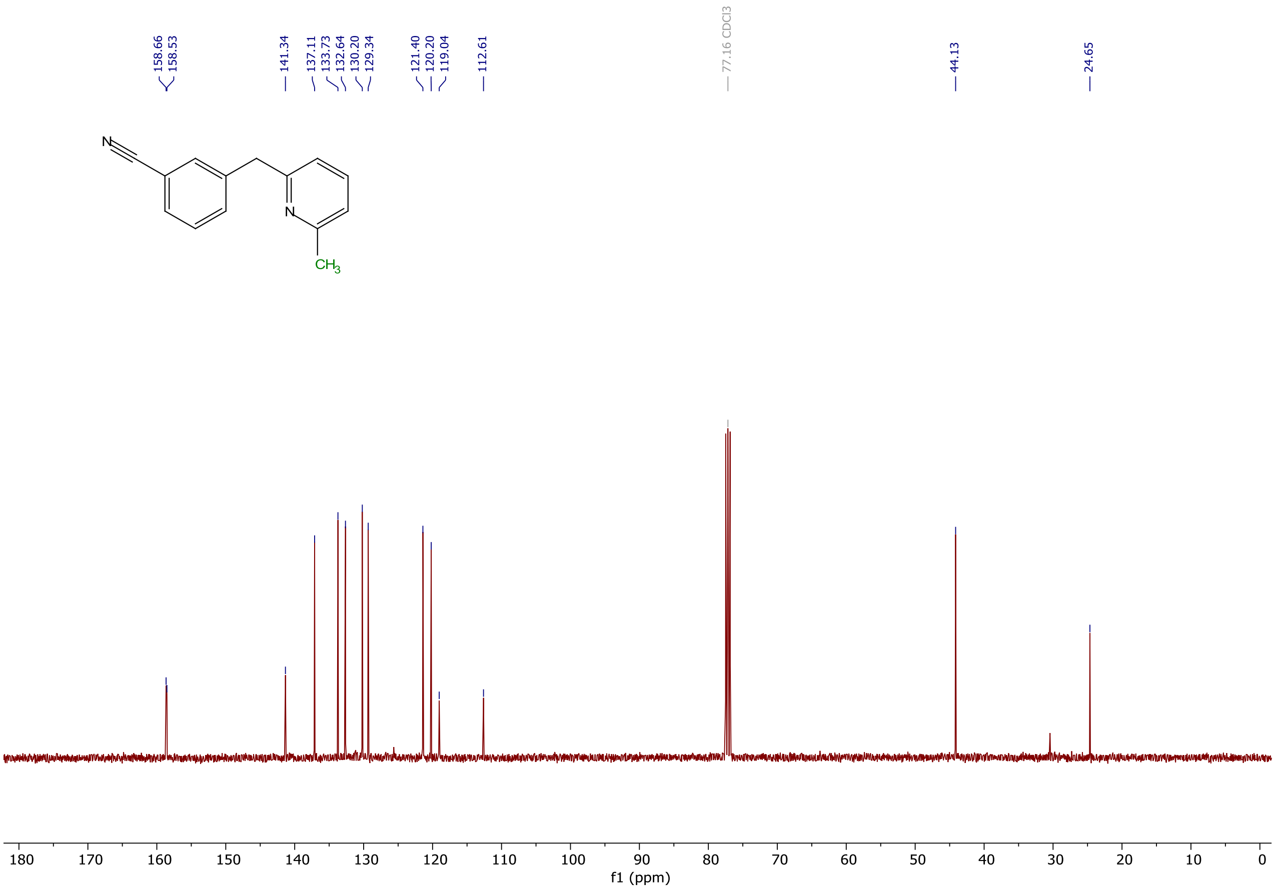

5-Benzyl-2-(trifluoromethyl)pyridine (5ad)

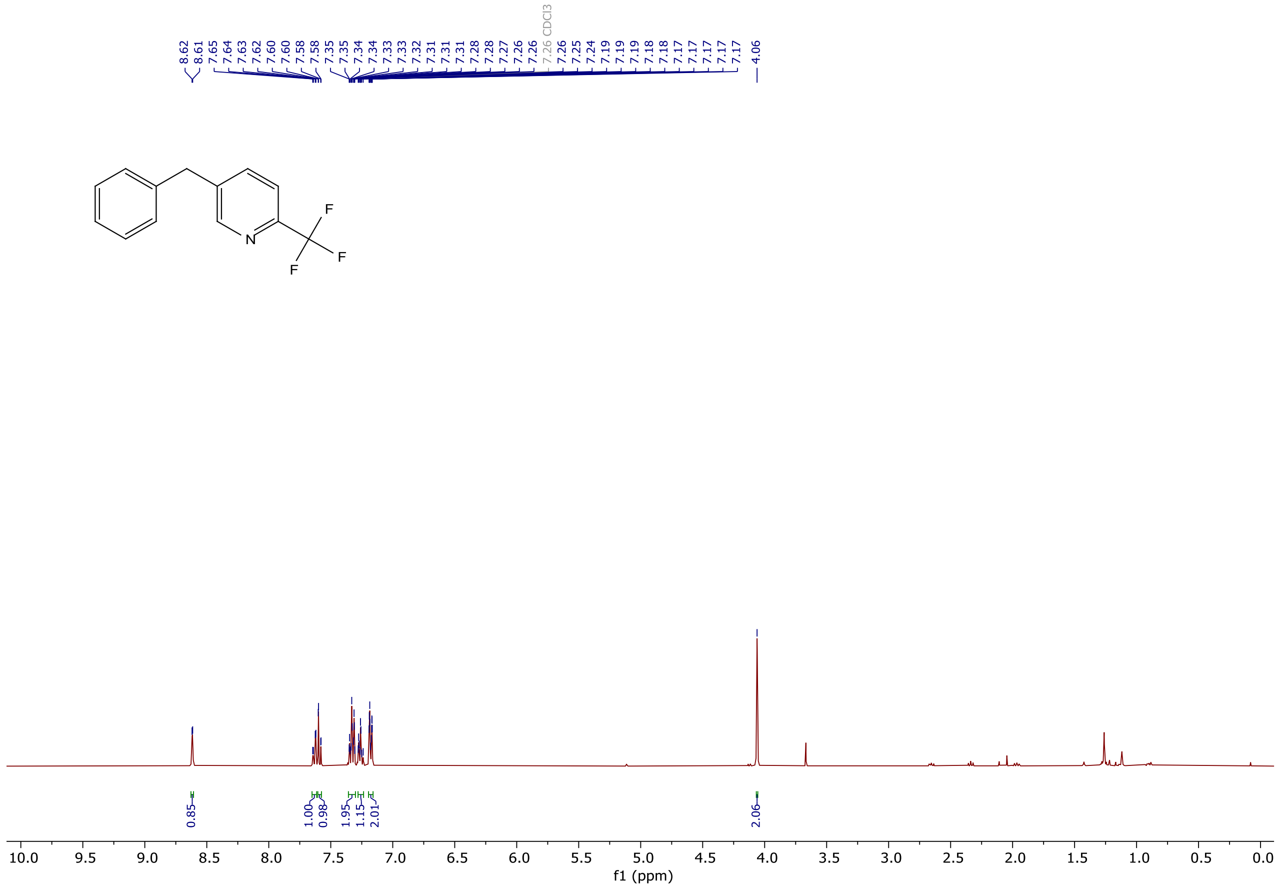

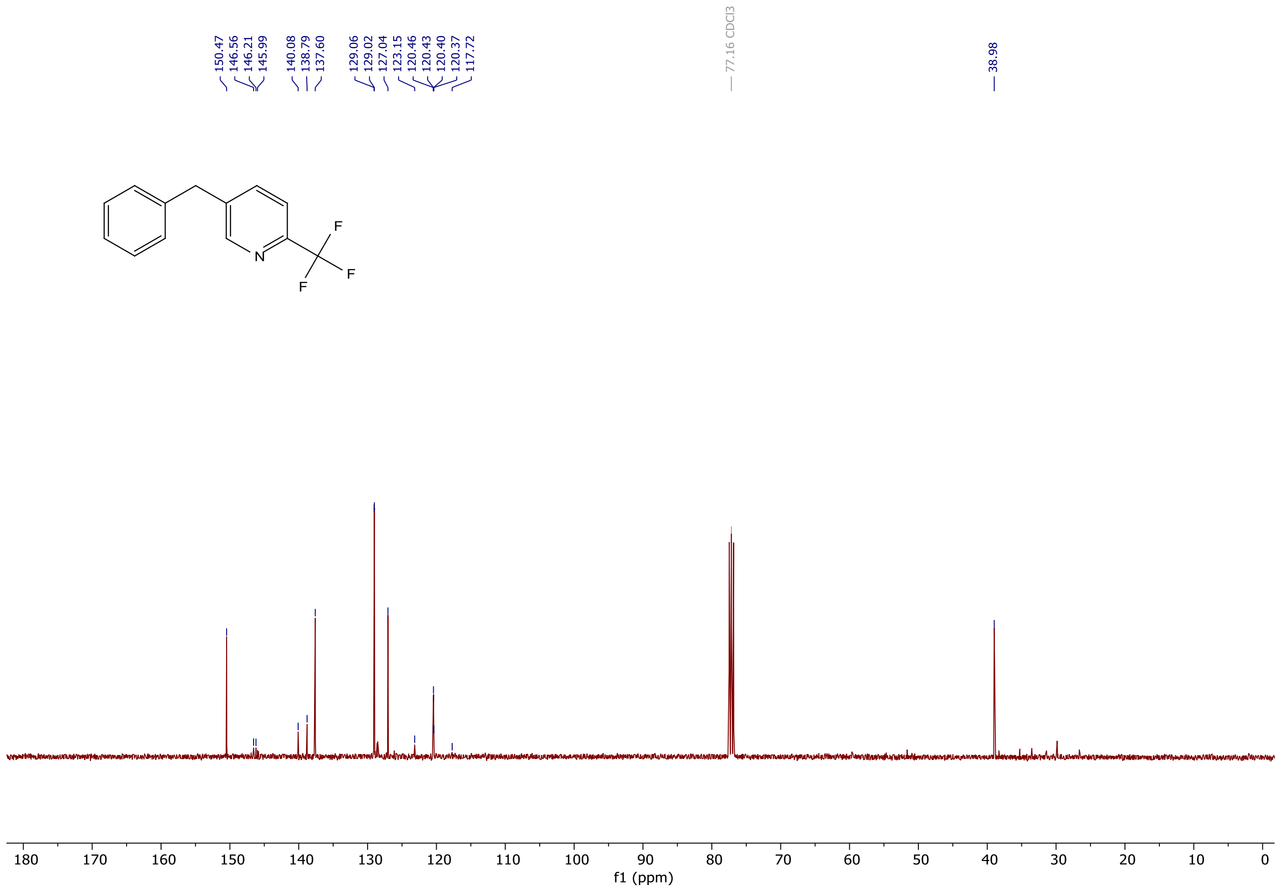

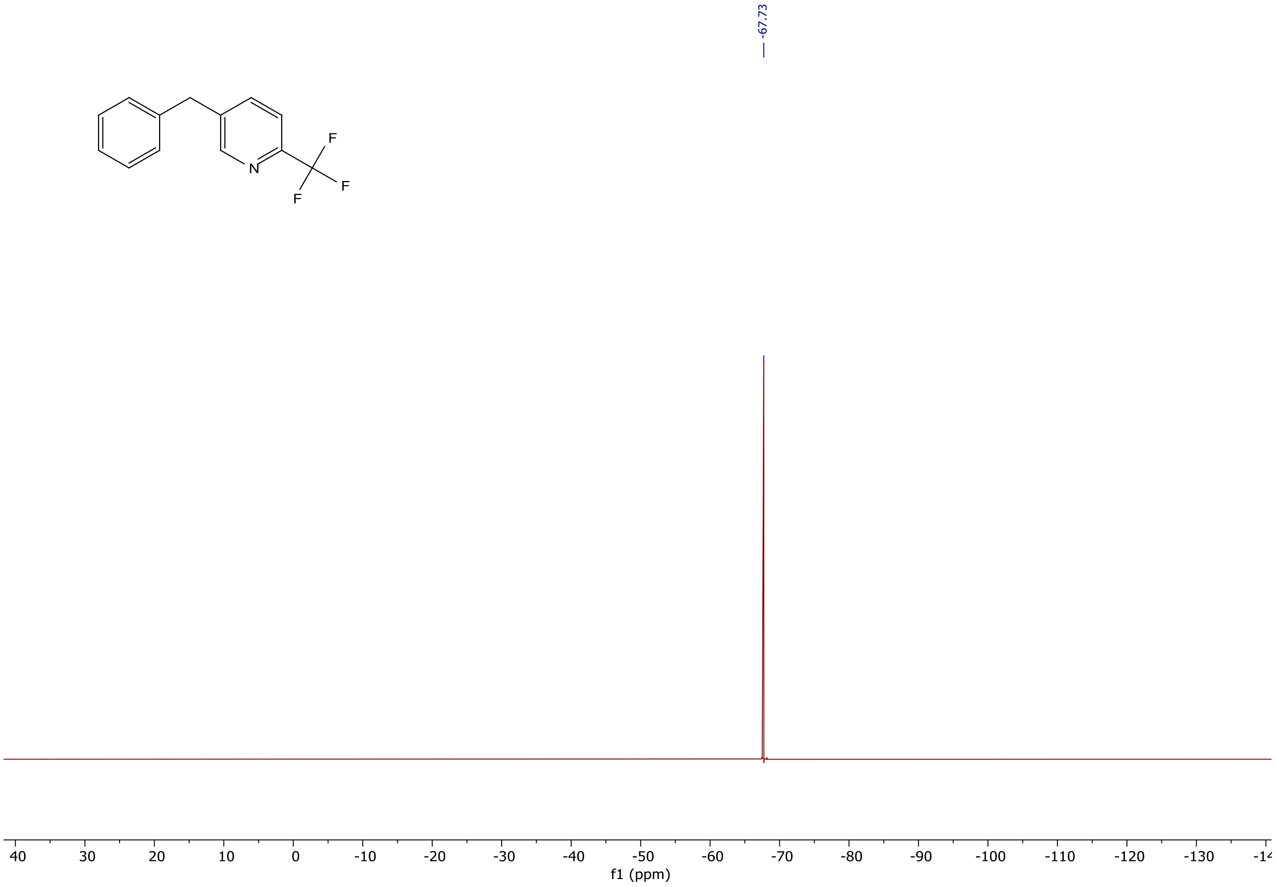
-67.73
N
F
F
F
40 30 20 10 0 -10 -20 -30 -40 -50 -60 -70 -80 -90 -100 -110 -120 -130
f1 (ppm)

Methyl 6-(3-cyanobenzyl)nicotinate (5ae)

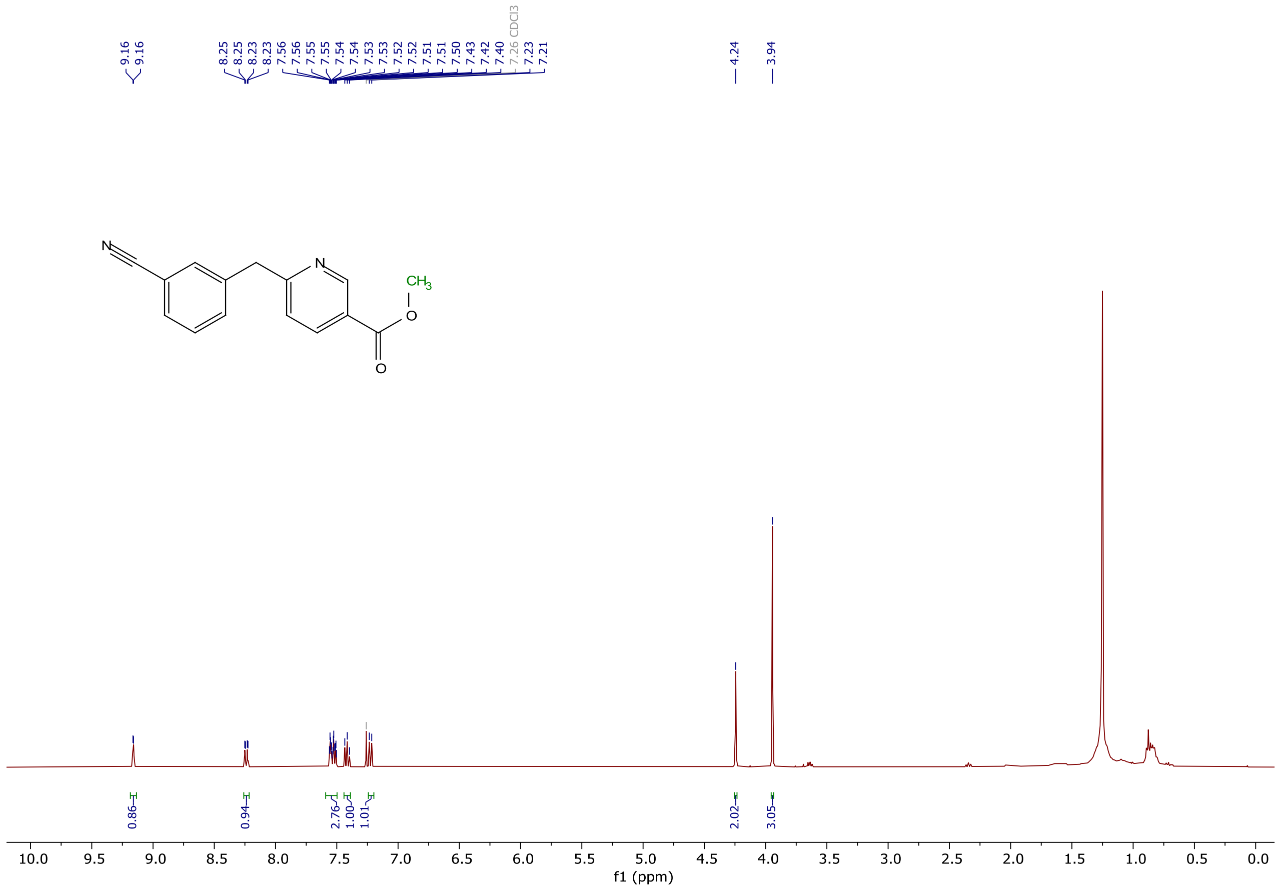

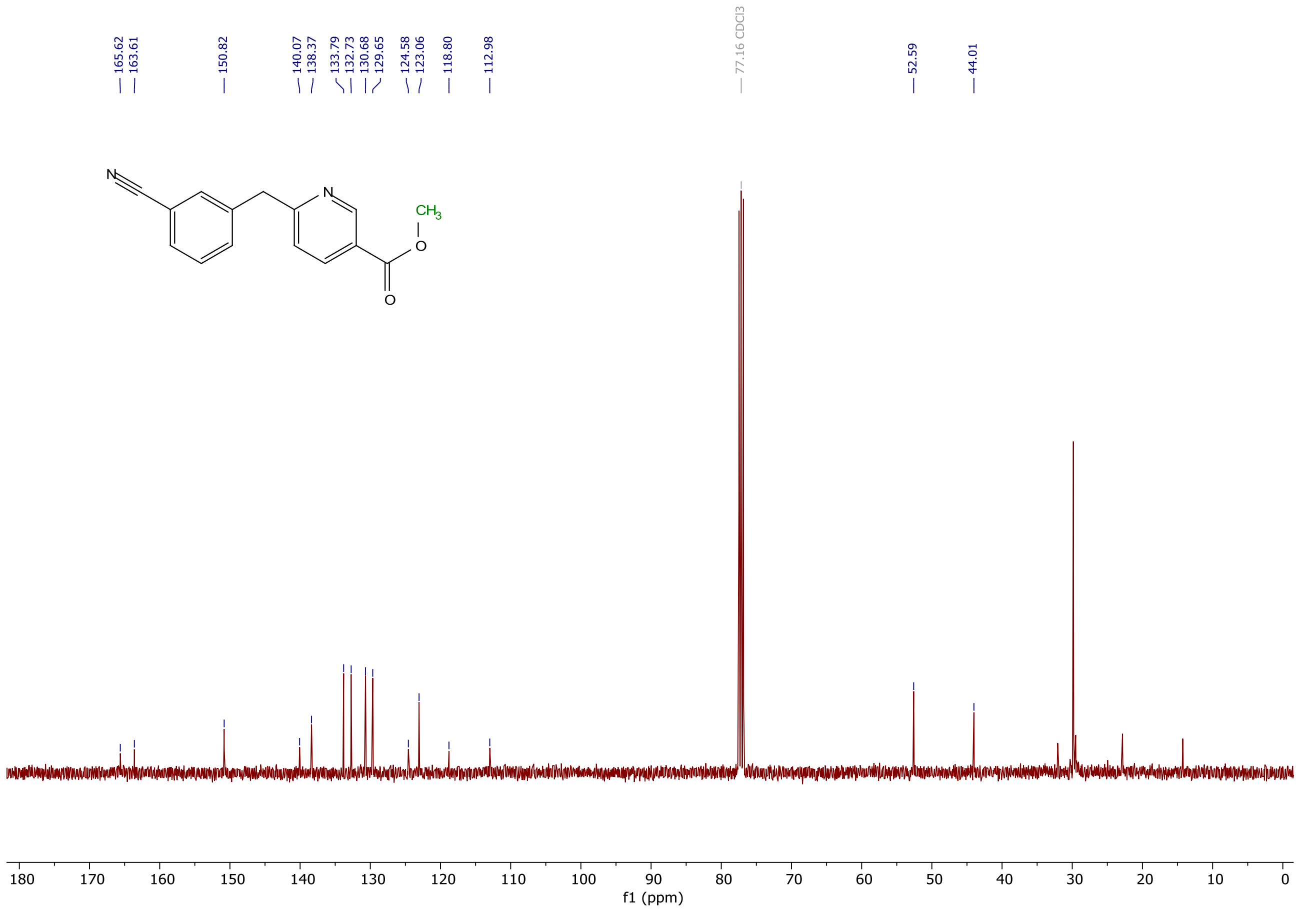

3-(Pyrazin-2-ylmethyl)benzonitrile (5af)

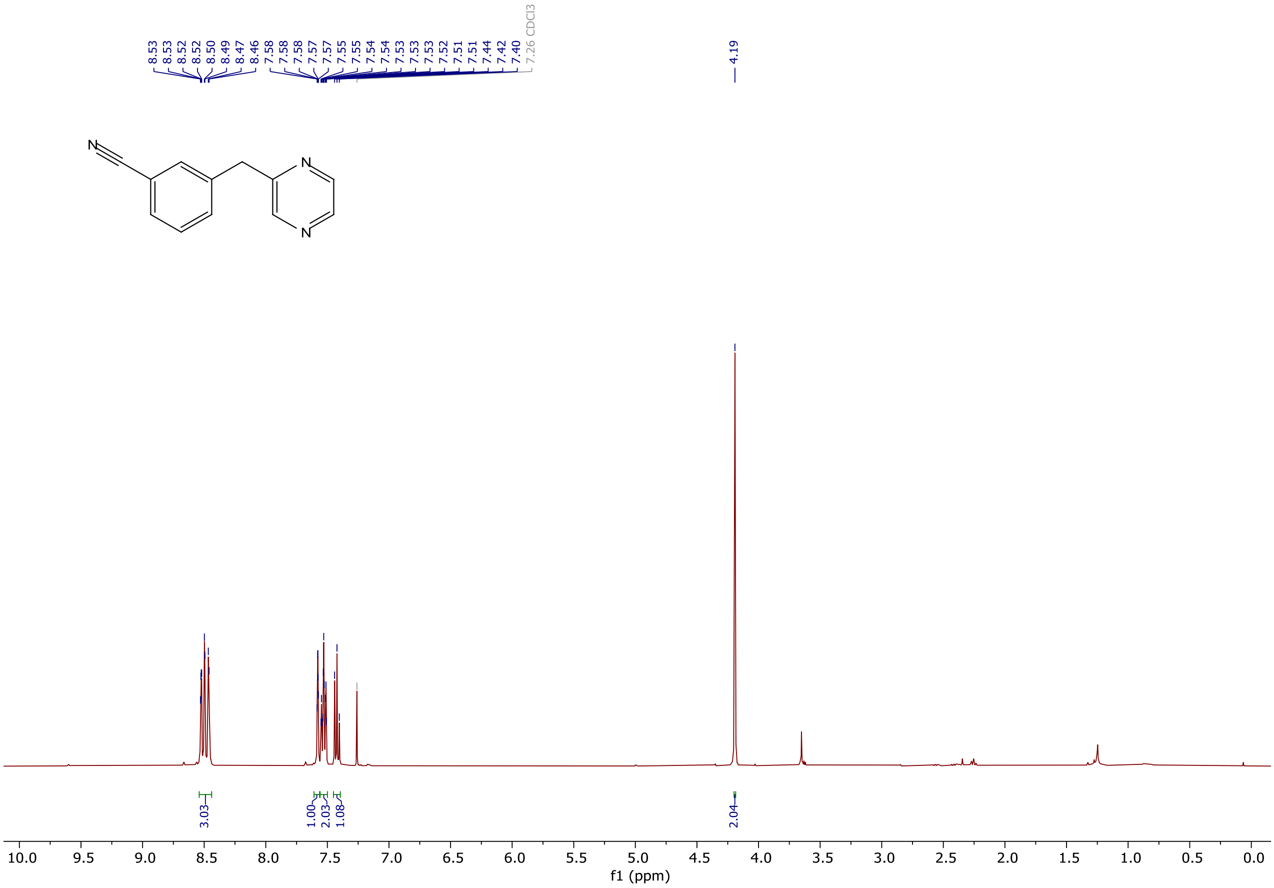

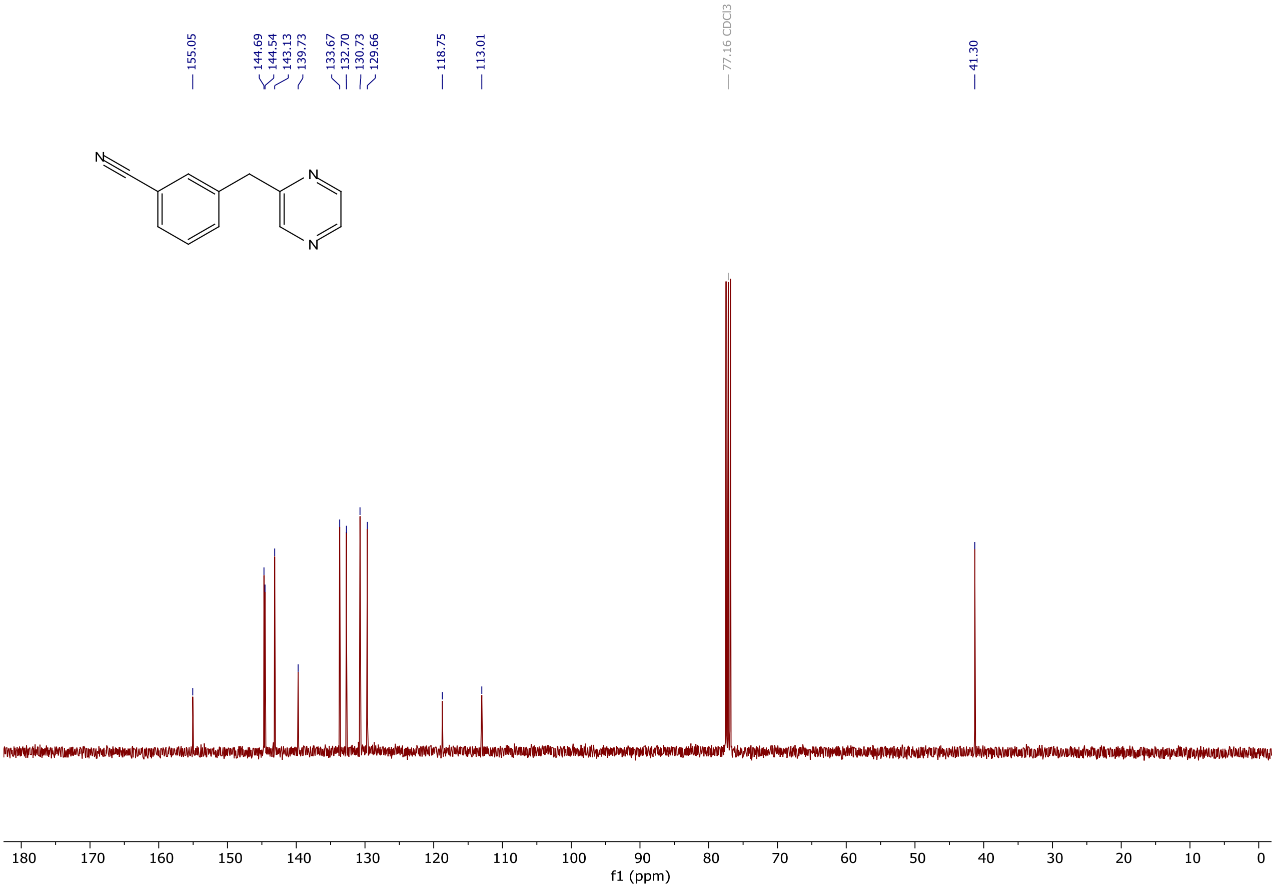

3-((2-Methoxypyrimidin-5-yl)methyl)benzonitrile (5ag)

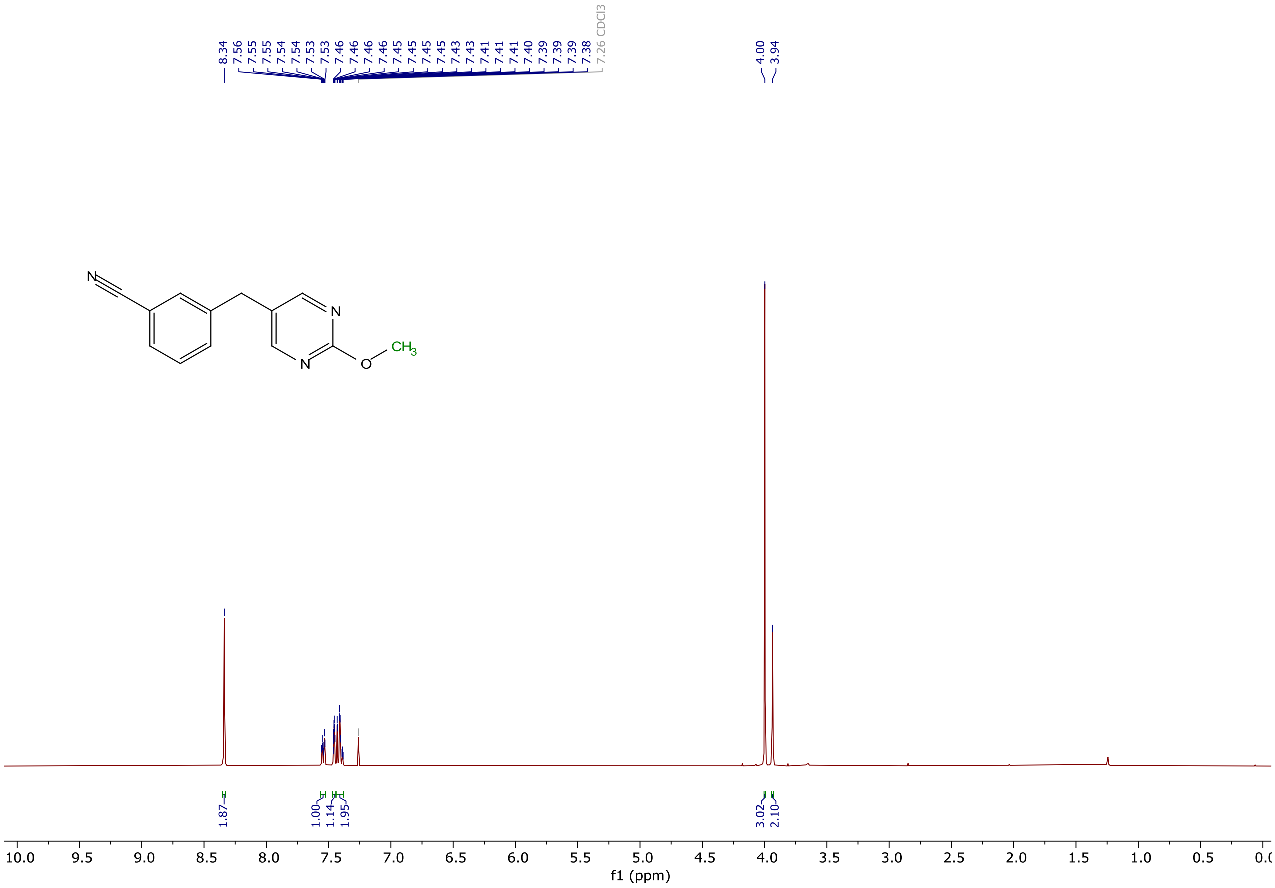

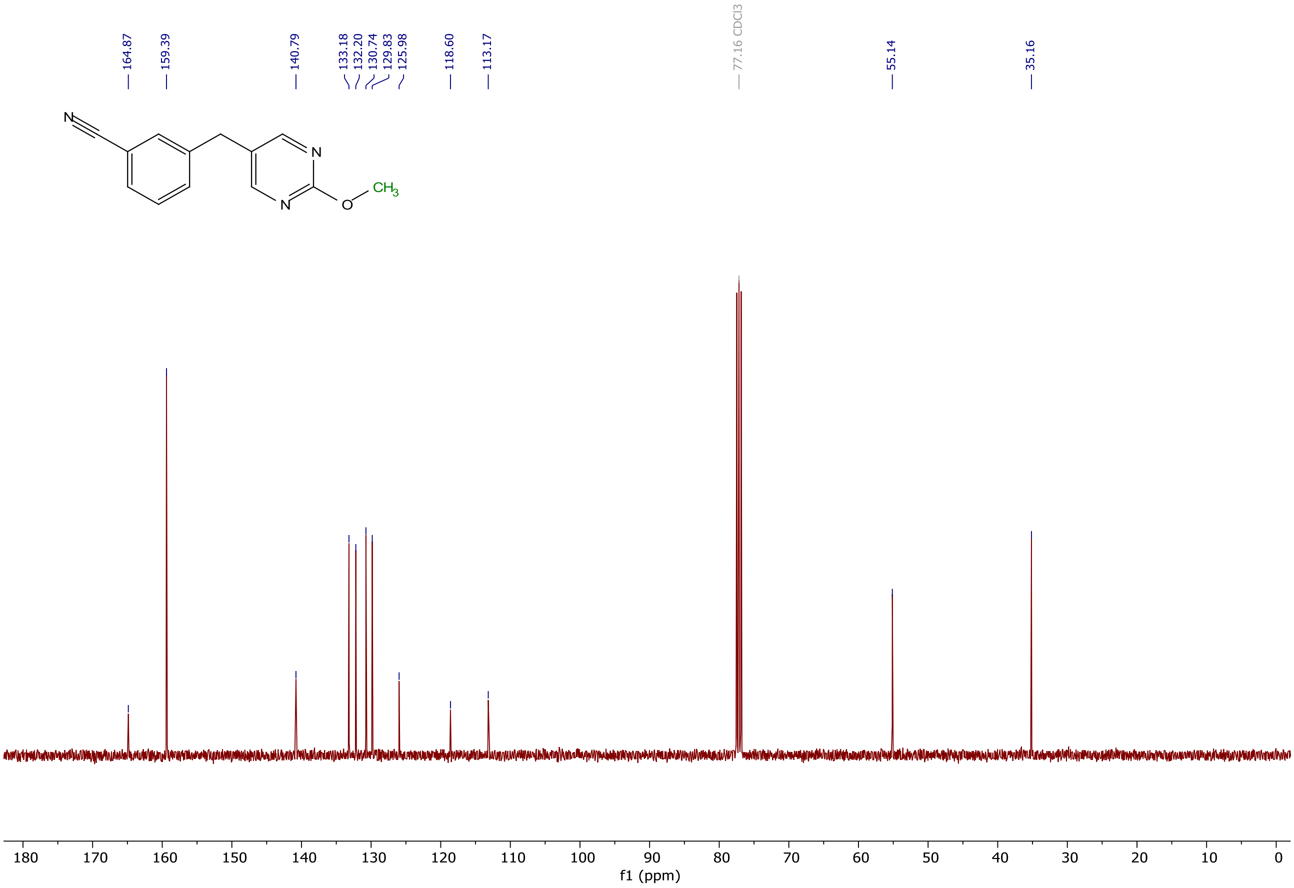

4-(3,5-Difluorobenzyl)-6-methyl-2-phenylpyrimidine (5ah)

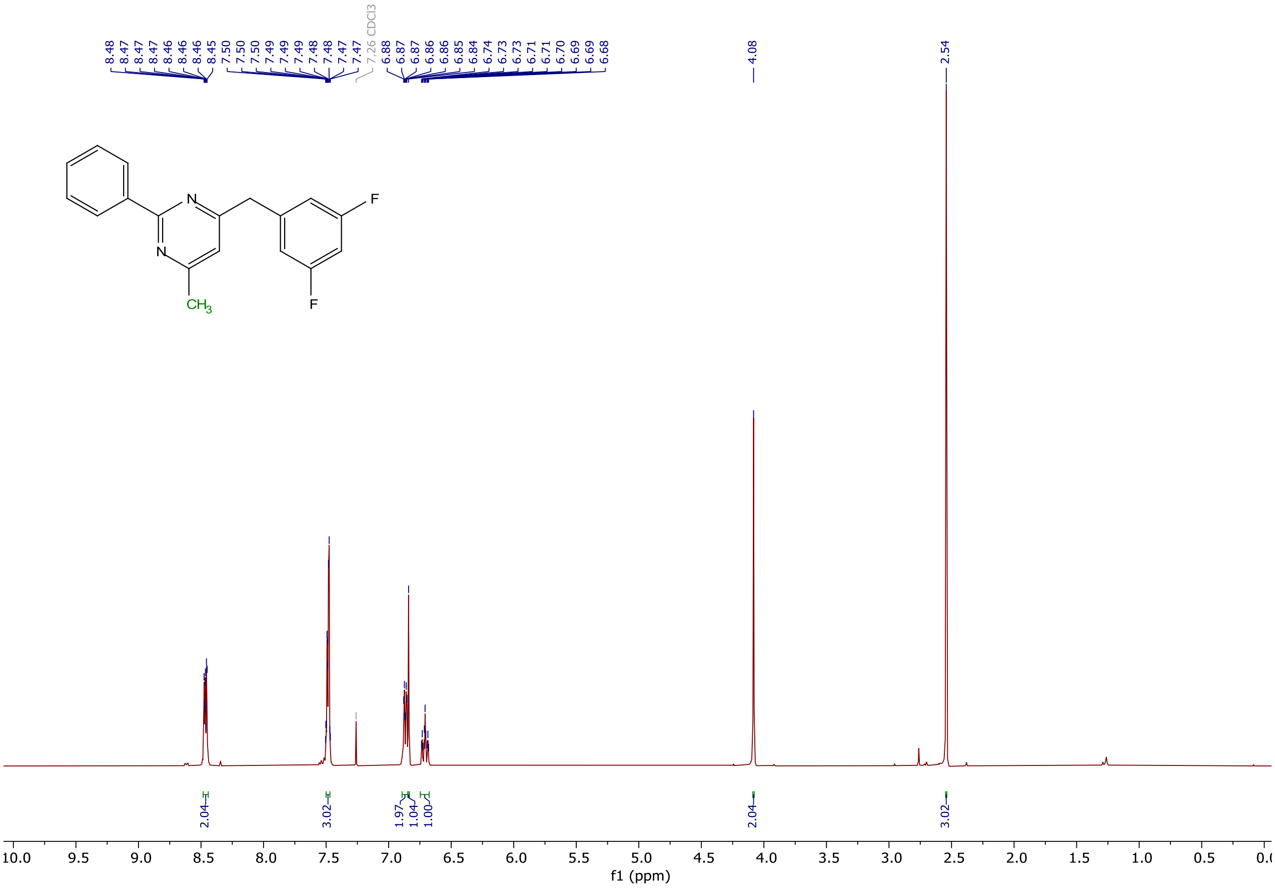

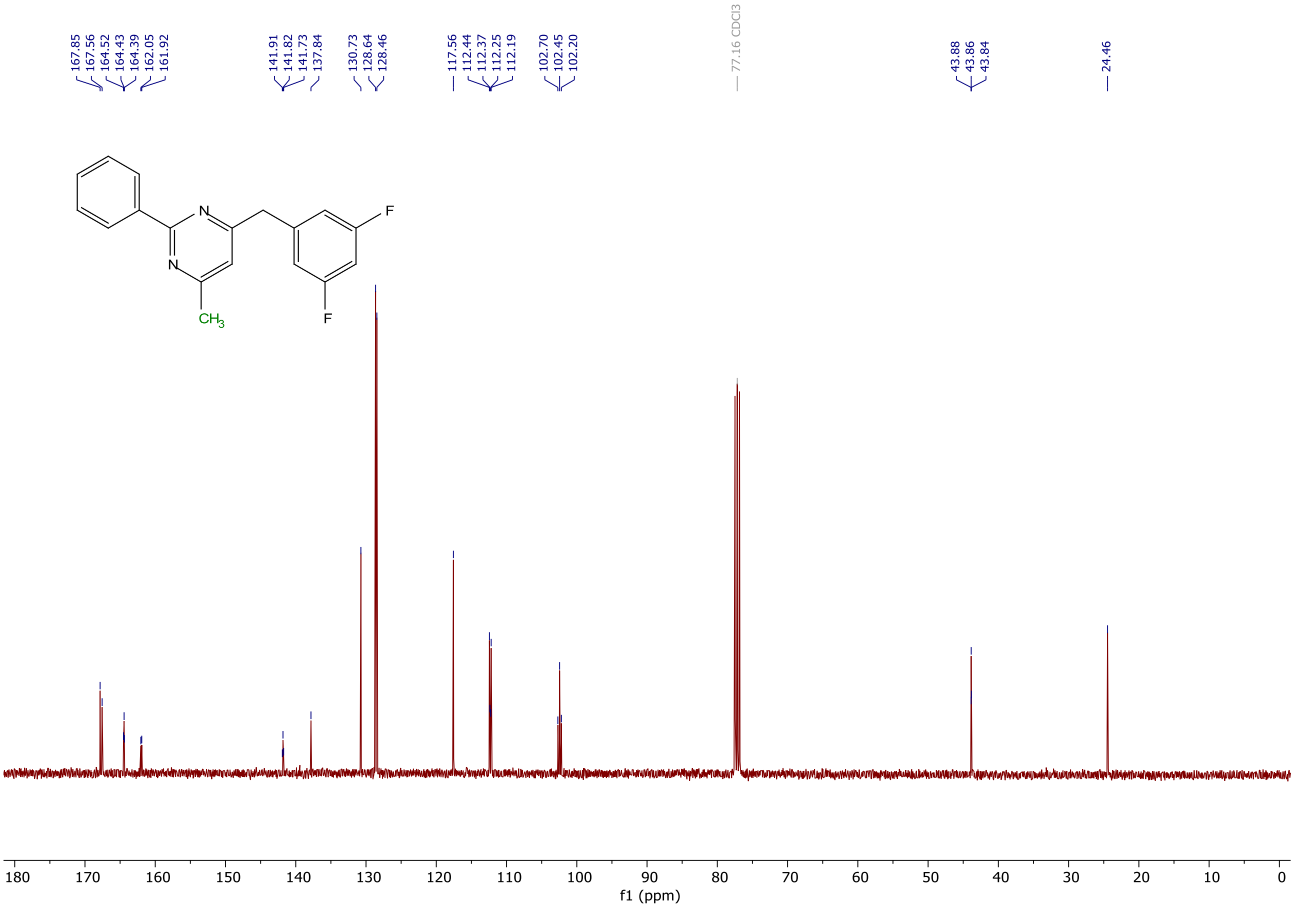

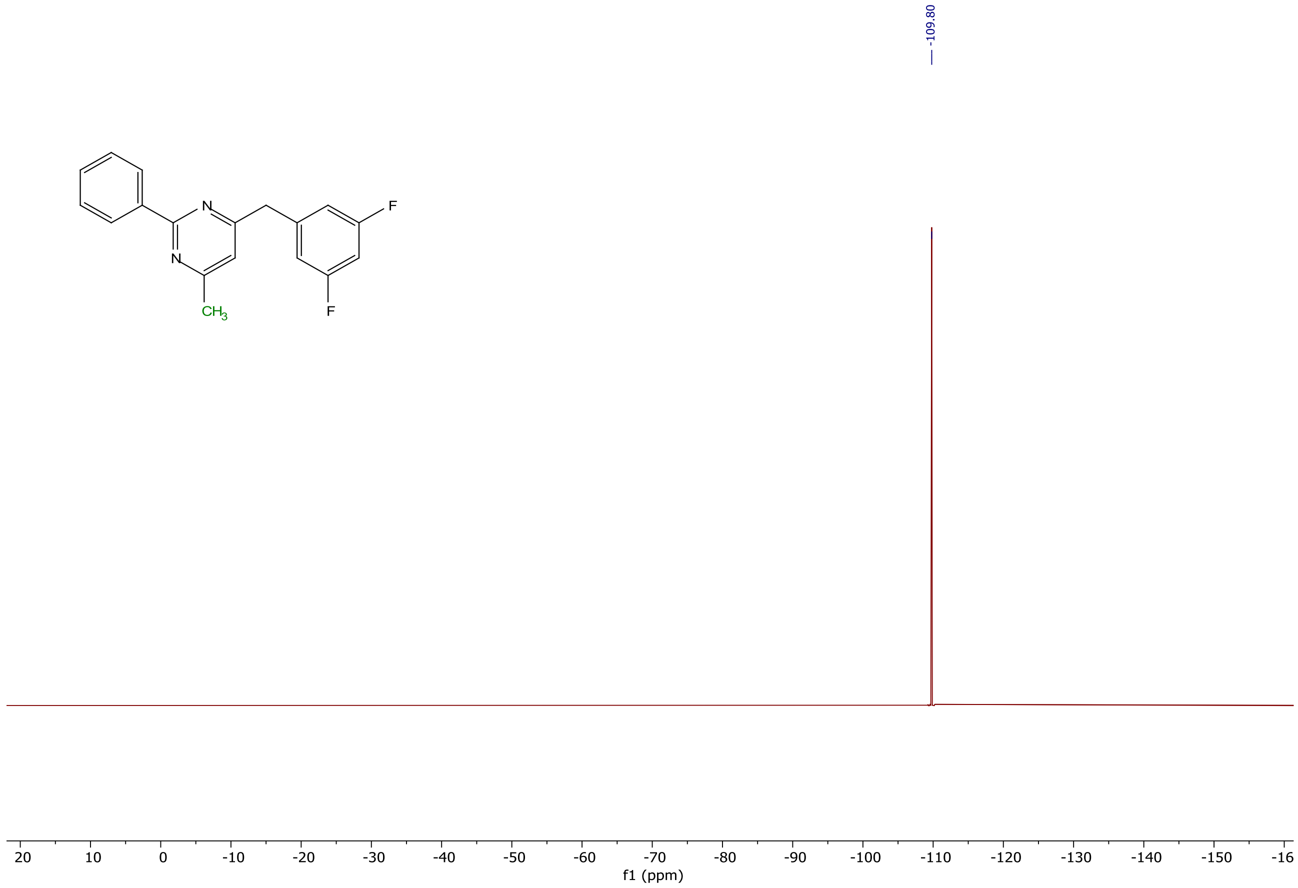
-109.80
N
N
F
F
CH3
20
10
0
-10
-20
-30
-40
-50
-60
-70
-80
-90
-100
-110
-120
-130
-140
-150
-16
f1 (ppm)

3-((2,6-Dimorpholinopyrimidin-4-yl)methyl)benzonitrile (5ai)

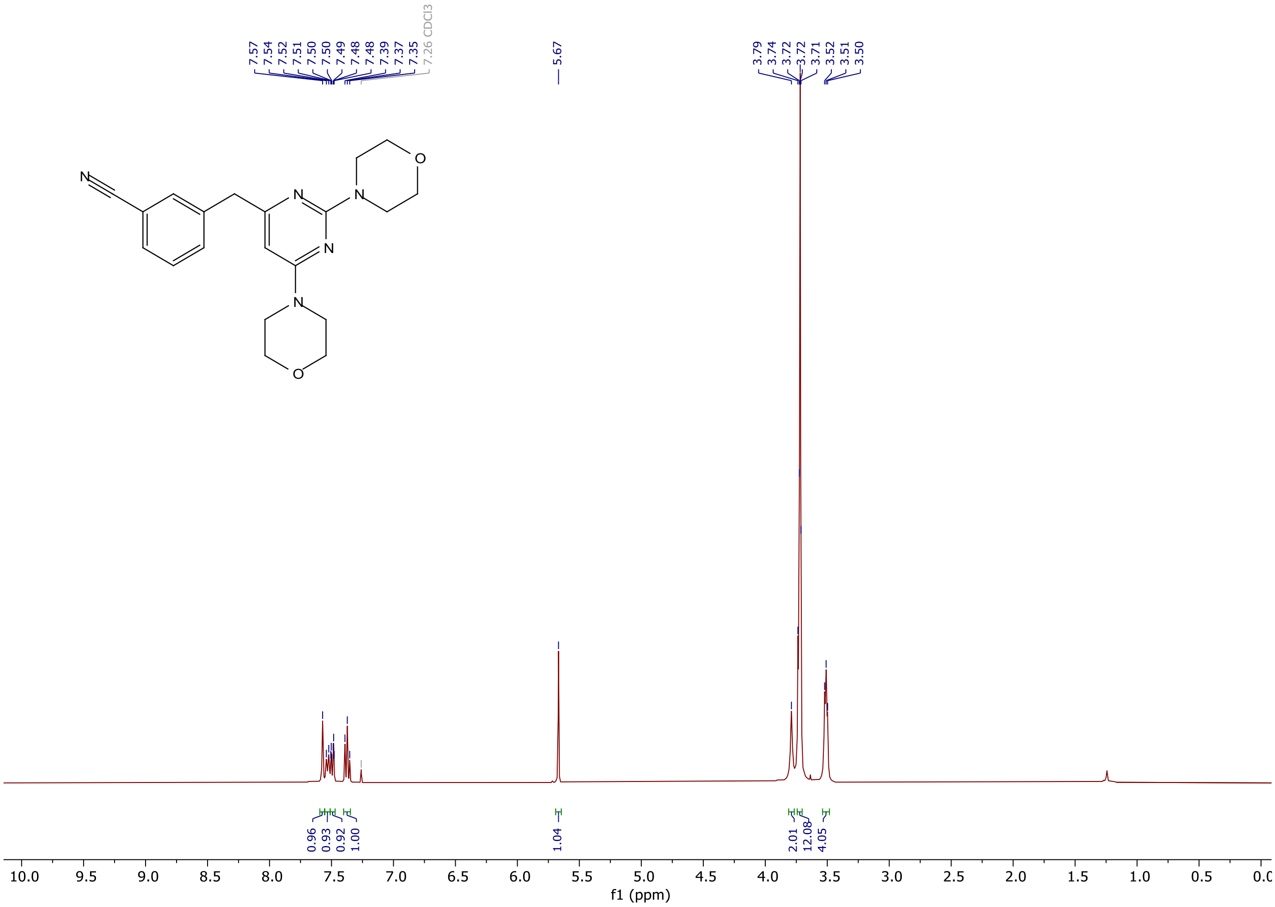

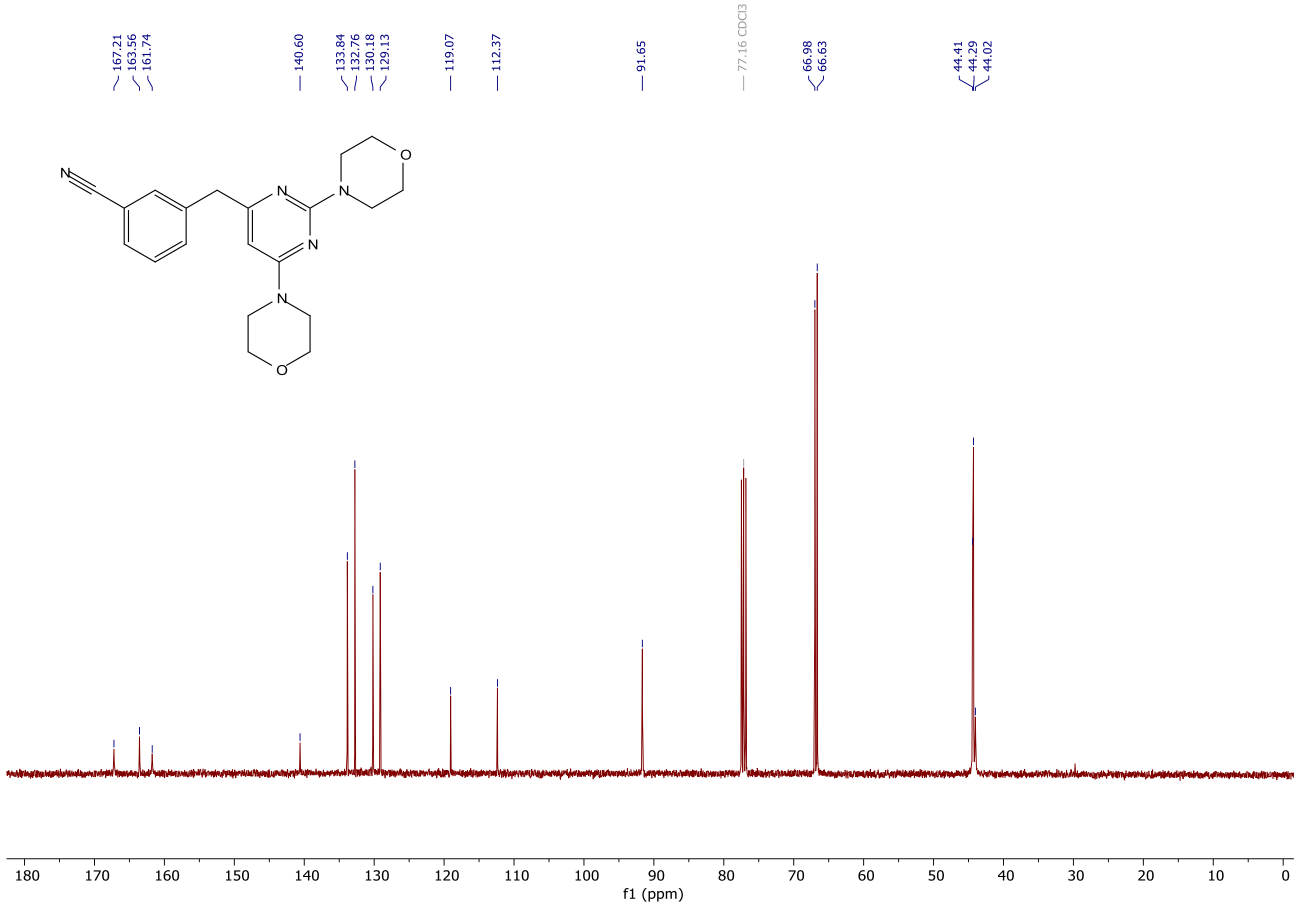

3-((4-Methyl-3-yl)methyl)benzonitrile (5aj)

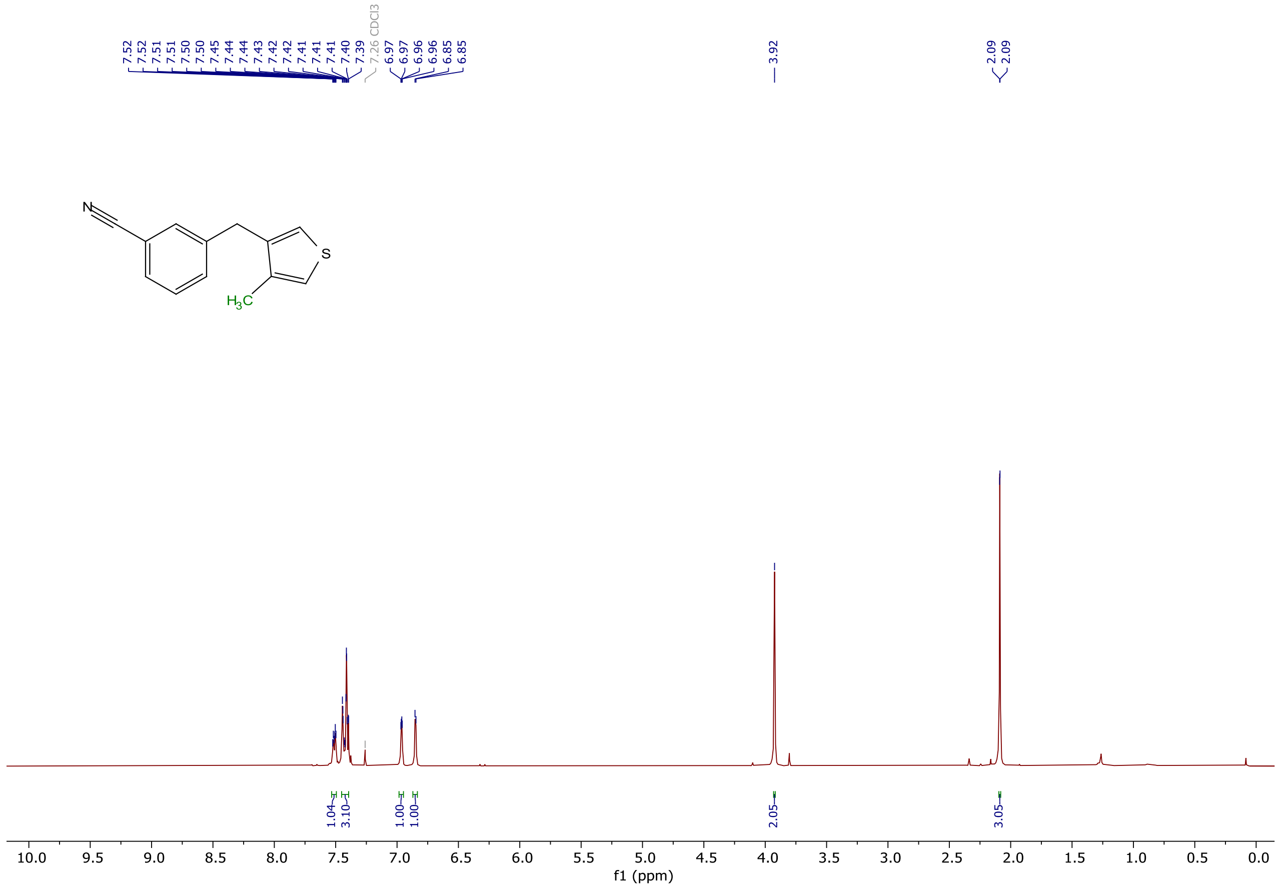

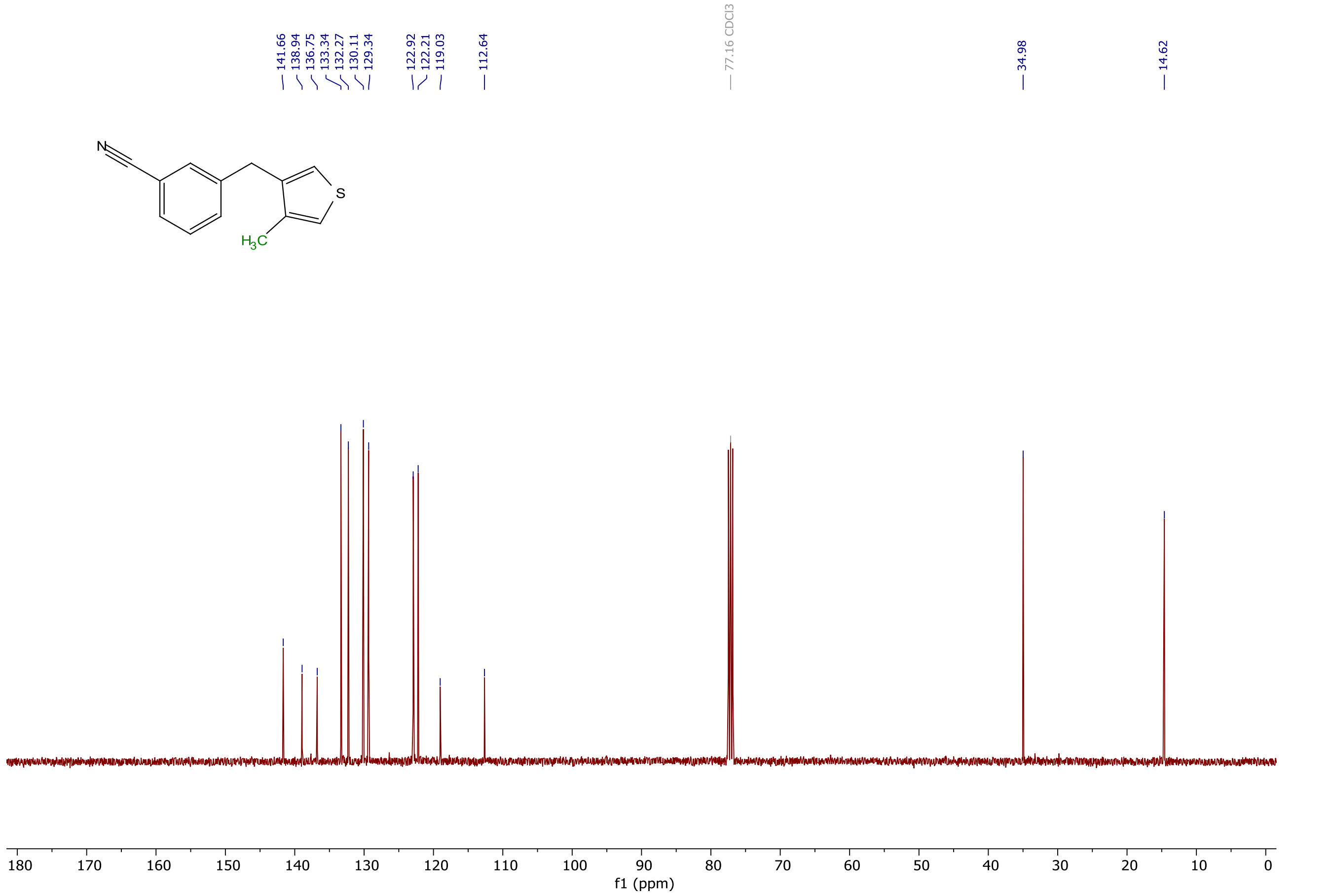

3-((3,5-Dimethylisoxazol-4-yl)methyl)benzonitrile (5ak)

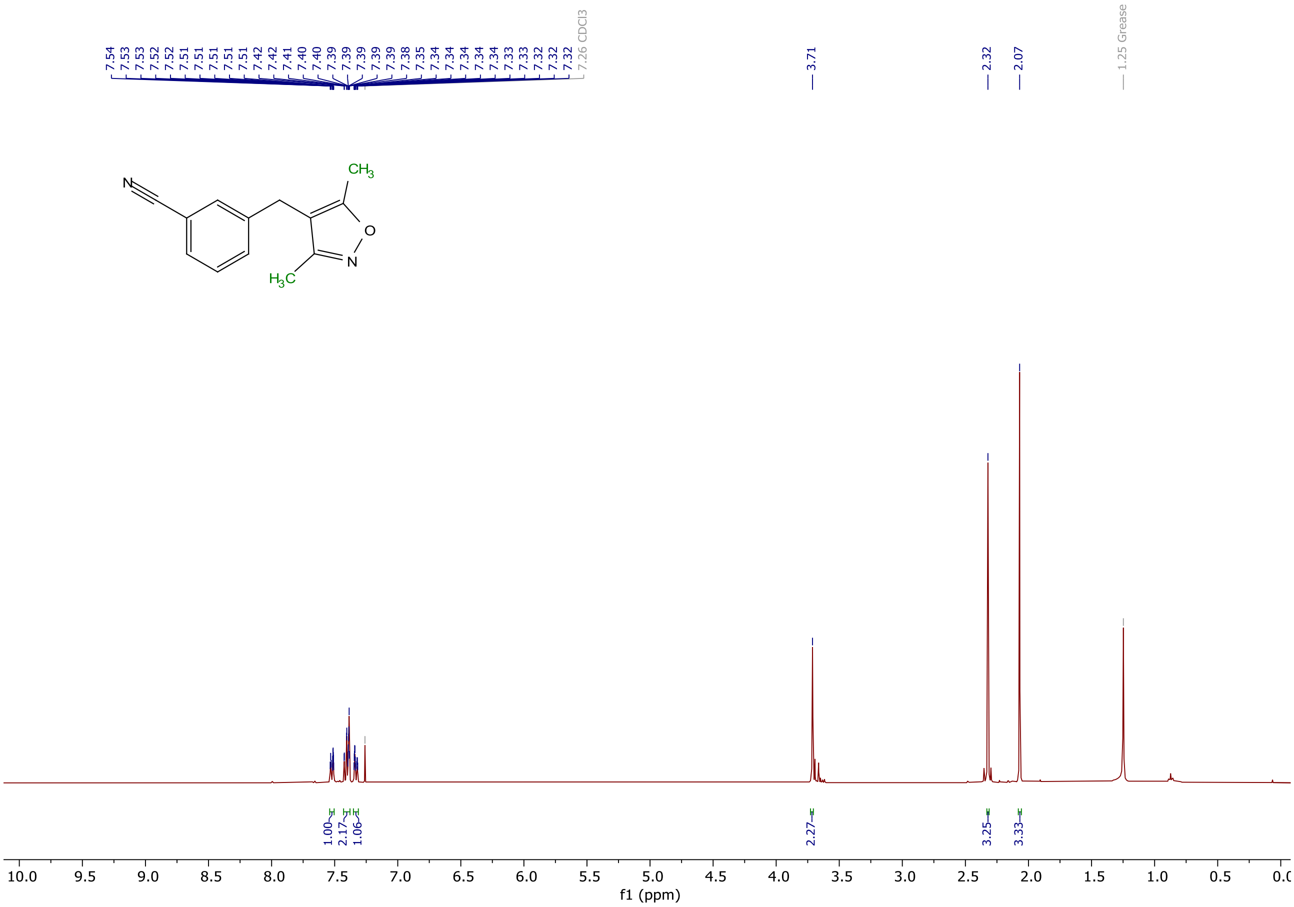
7.54
7.53
7.53
7.52
7.52
7.51
7.51
7.51
7.51
7.51
7.42
7.42
7.41
7.40
7.40
7.39
7.39
7.39
7.39
7.39
7.39
7.38
7.35
7.34
7.34
7.34
7.34
7.34
7.33
7.33
7.32
7.32
7.32
7.26 CDCl3
3.71
2.32
2.07
1.25 Grease
CH3
N
O
N
H3C
1.00
2.17
1.06
2.27
3.25
3.33
f1 (ppm)

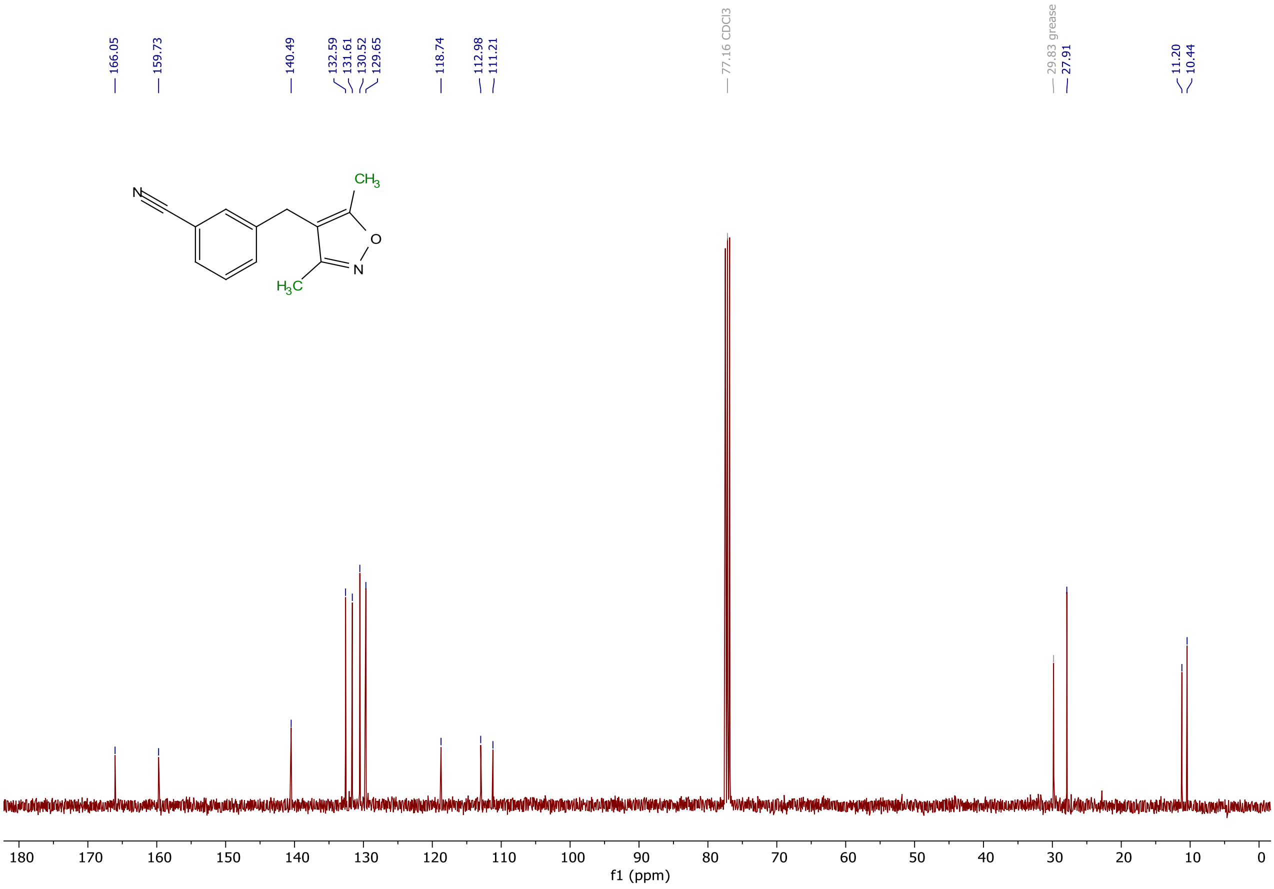
166.05
159.73
140.49
132.59
131.61
130.52
129.65
118.74
112.98
111.21
77.16 CDCl3
29.83 grease
27.91
11.20
10.44
CH3
N
O
N
H3C
f1 (ppm)

3-((1-Phenyl-1H-pyrazol-3-yl)methyl)benzonitrile (5al)

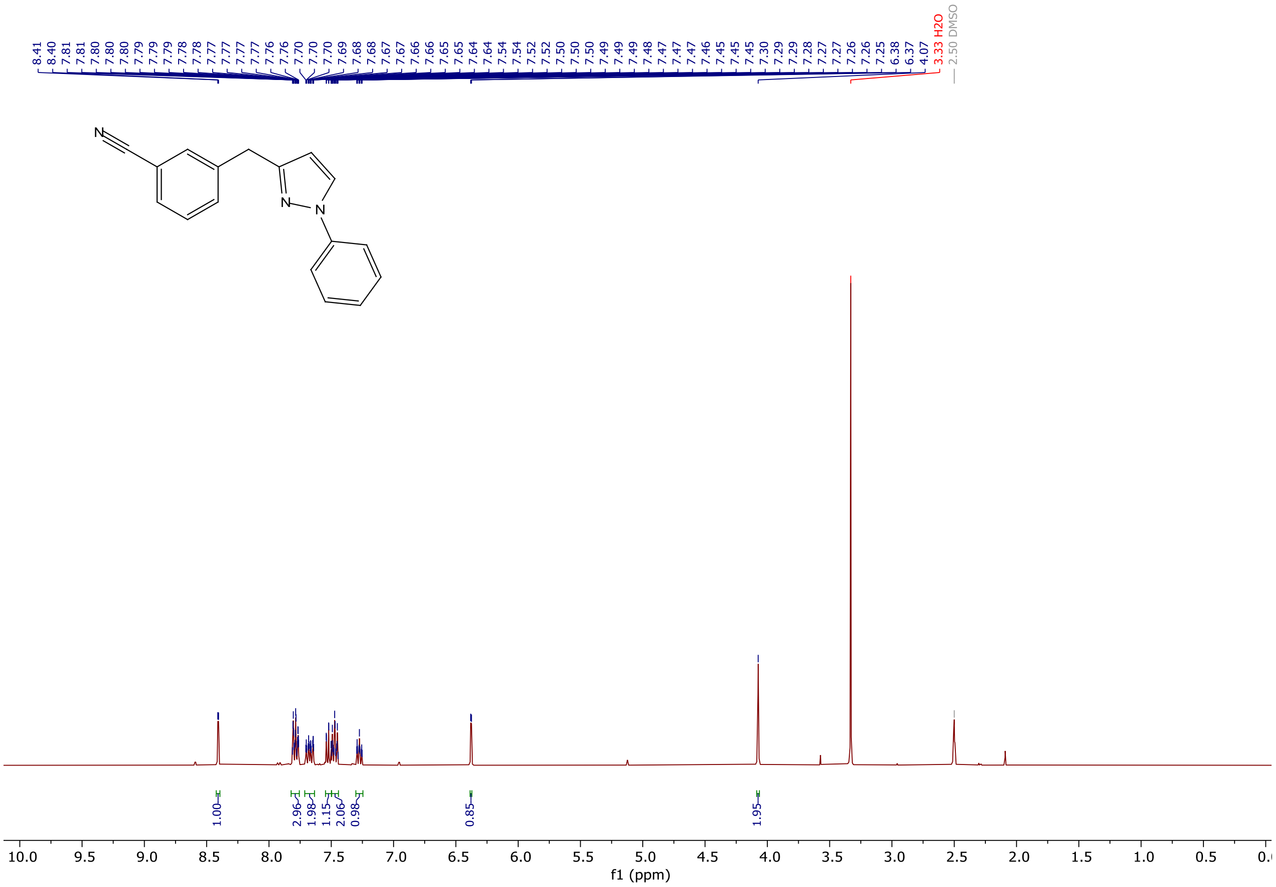

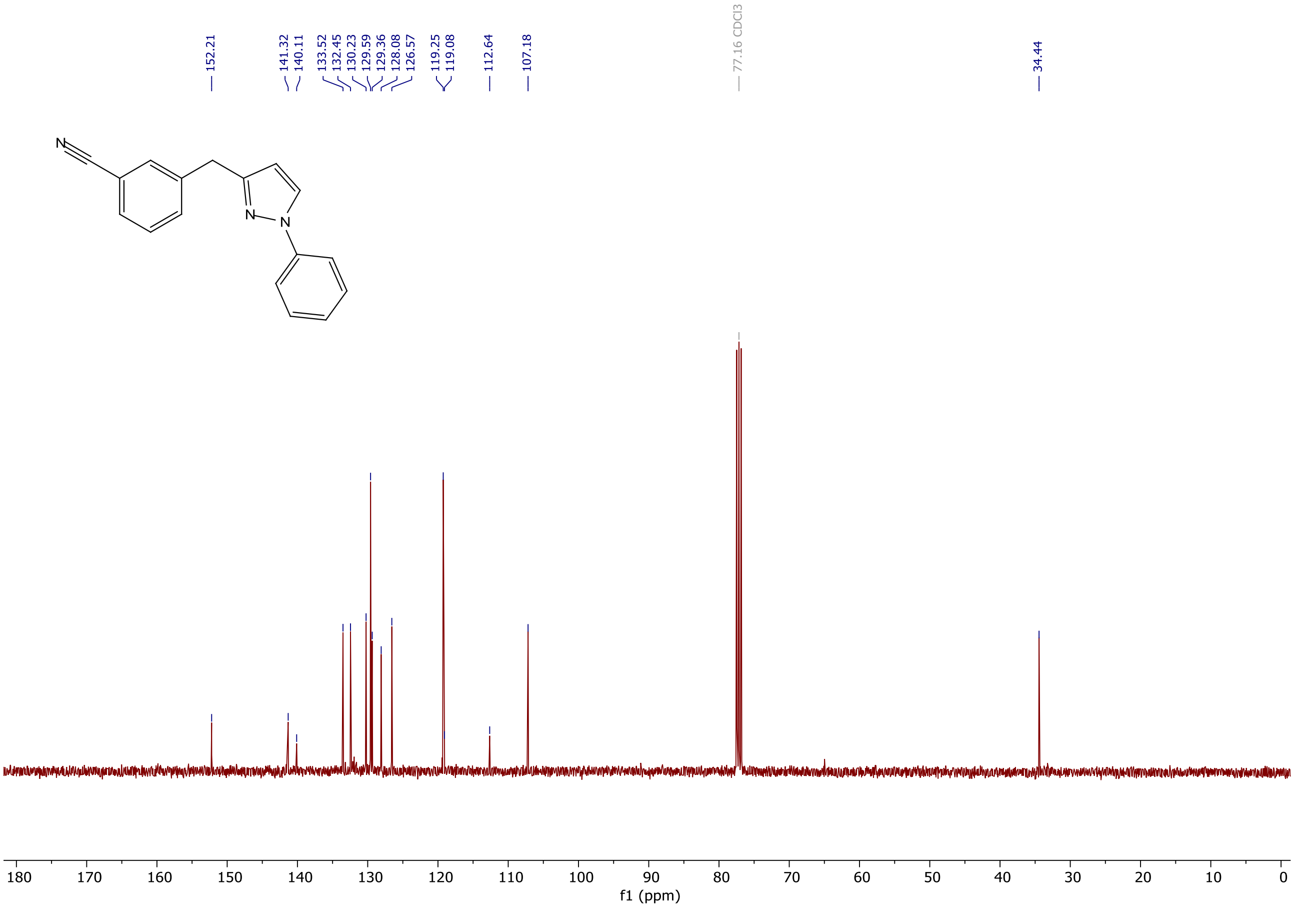

3-(Imidazo[1,2-a]pyrazin-3-ylmethyl)benzonitrile (5am)

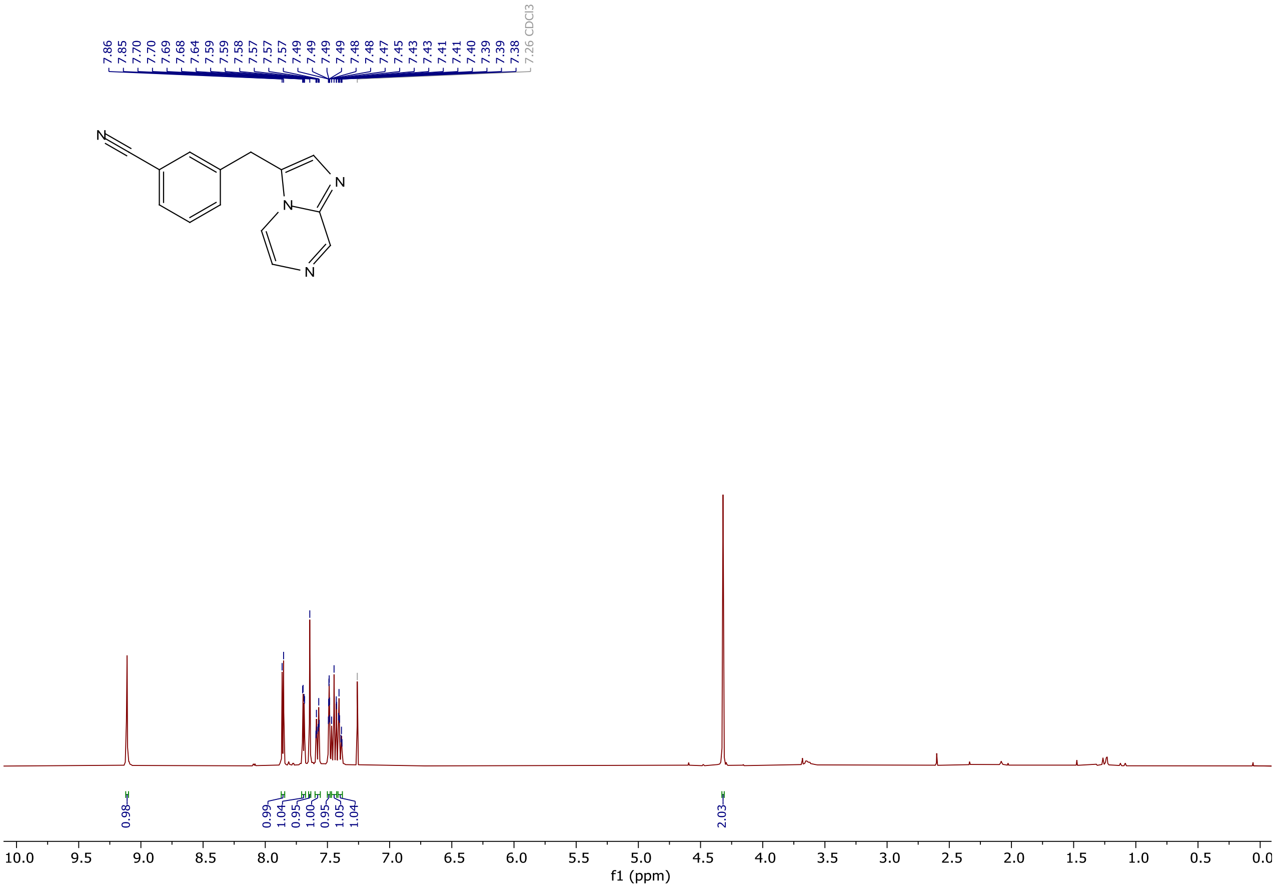

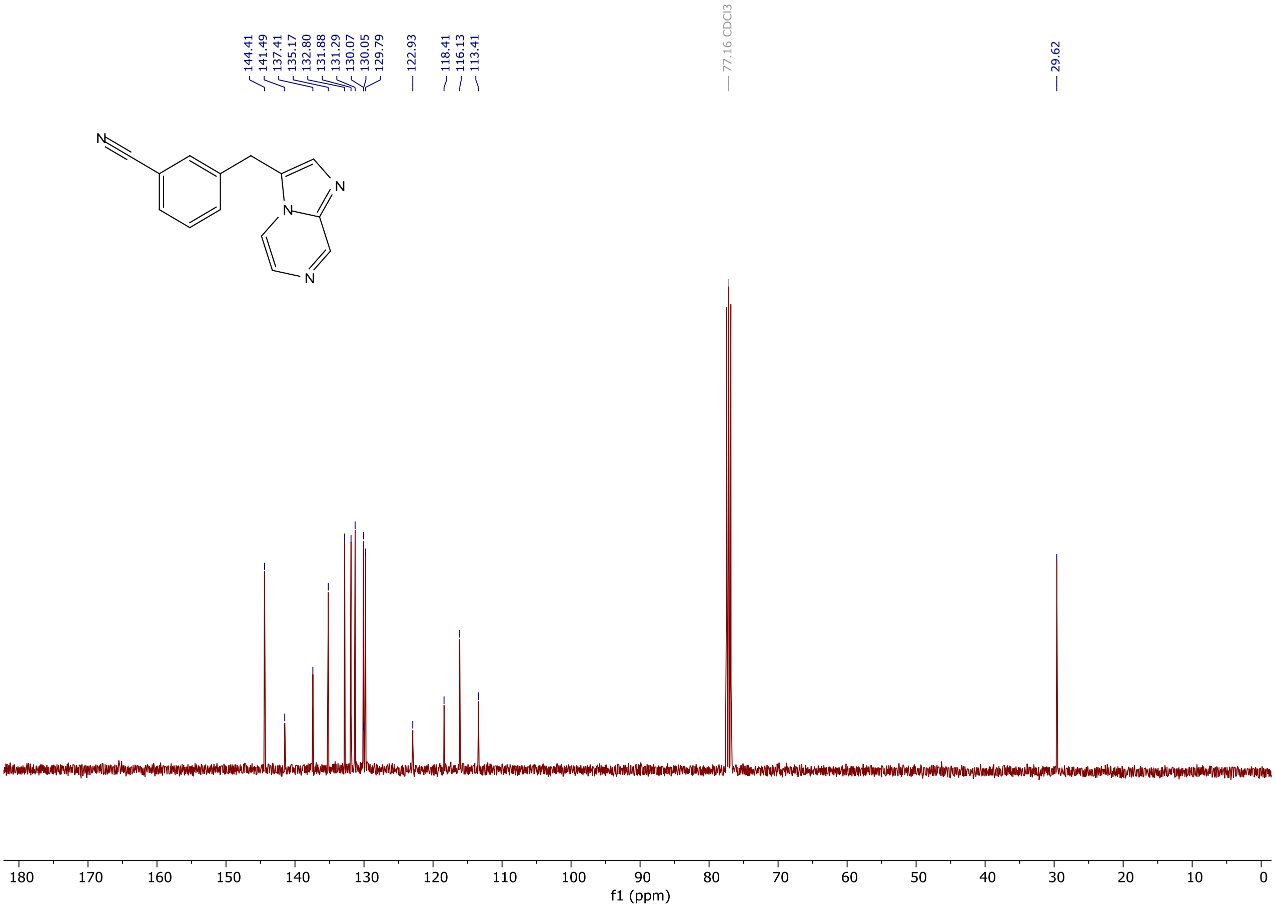

6-Benzylimidazo[1,2-a]pyridine (5an)

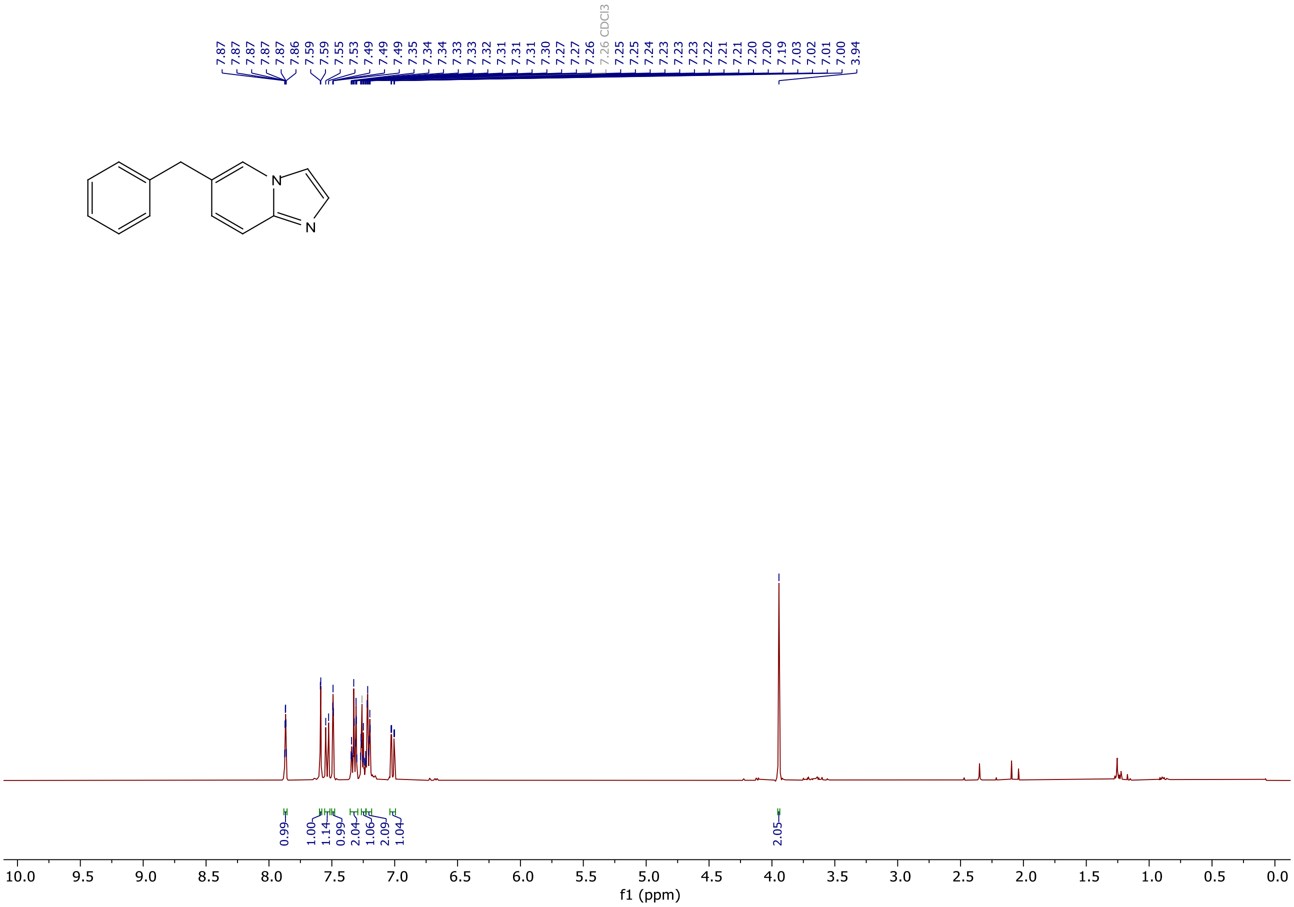

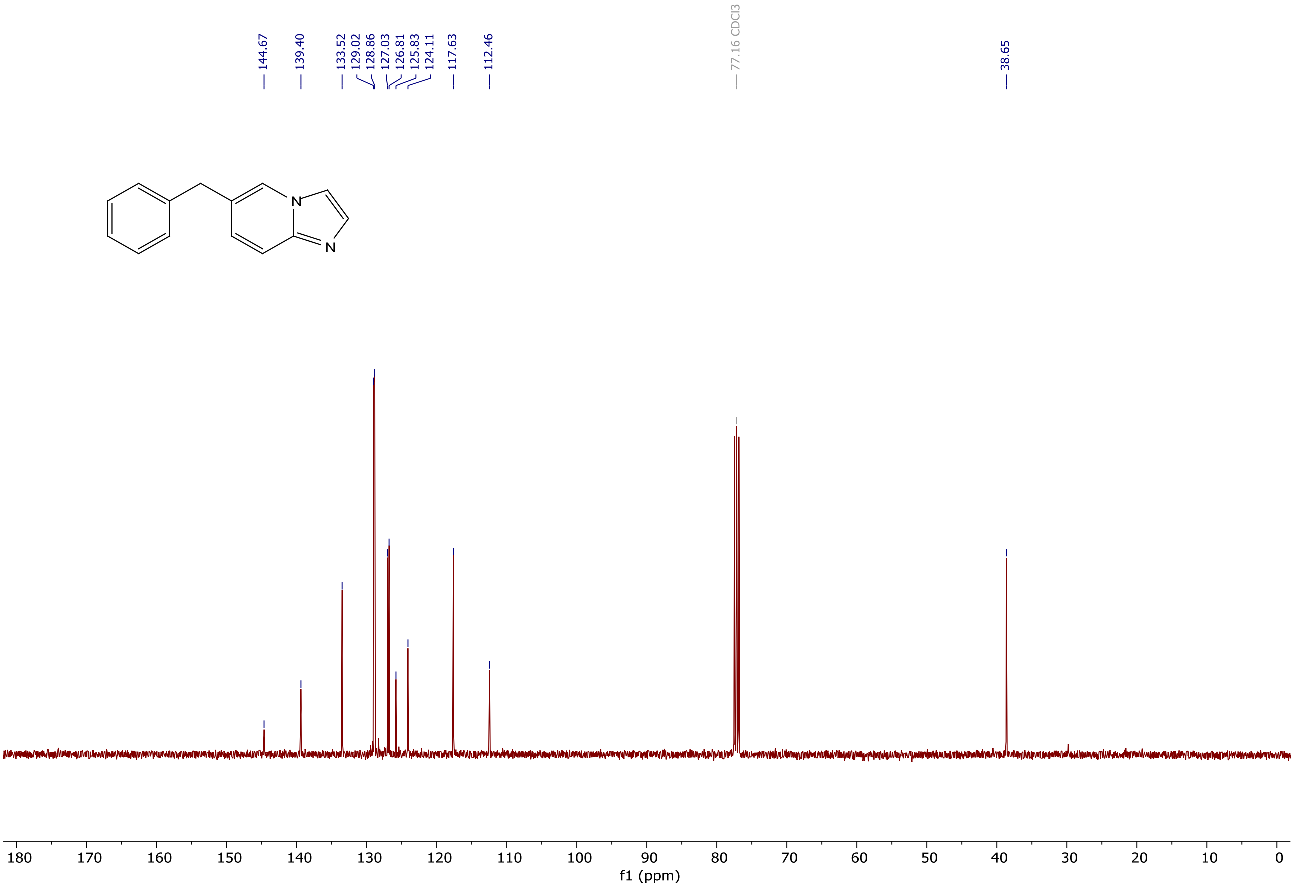

3-((2-Phenylimidazo[1,2-a]pyridin-3-yl)methyl)benzonitrile (5ao)

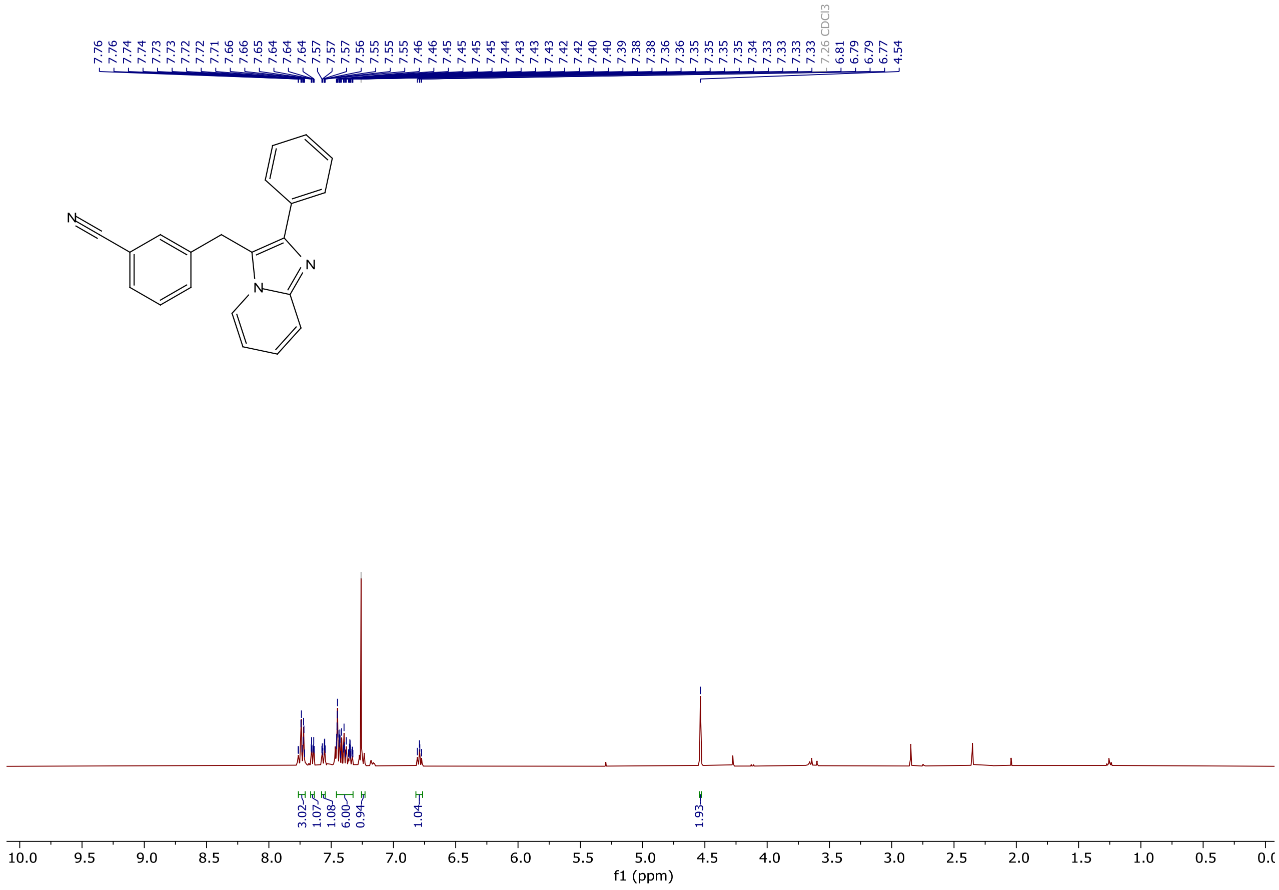

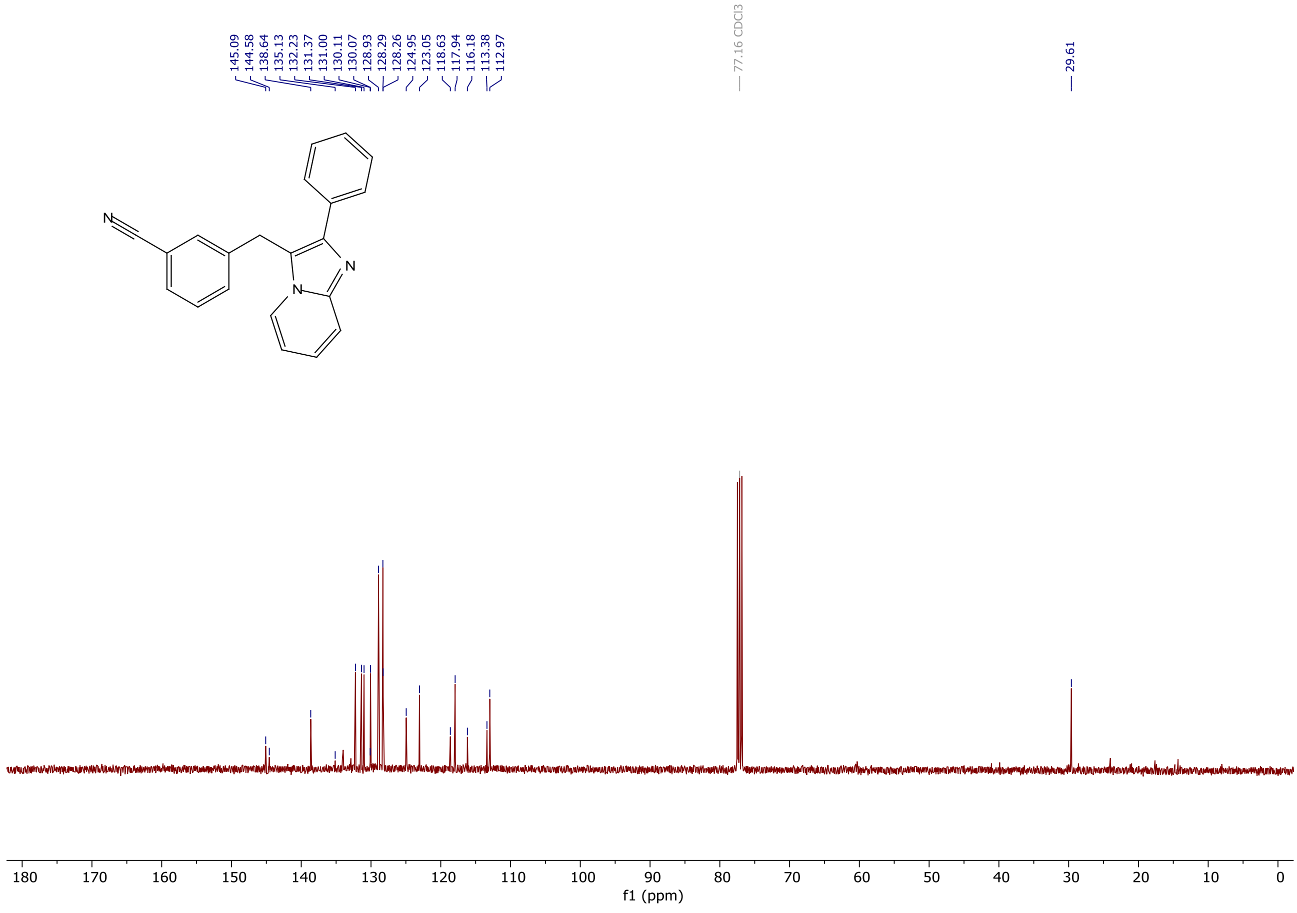

5-(3,5-Difluorobenzyl)-1-methyl-1H-indazole (5ap)

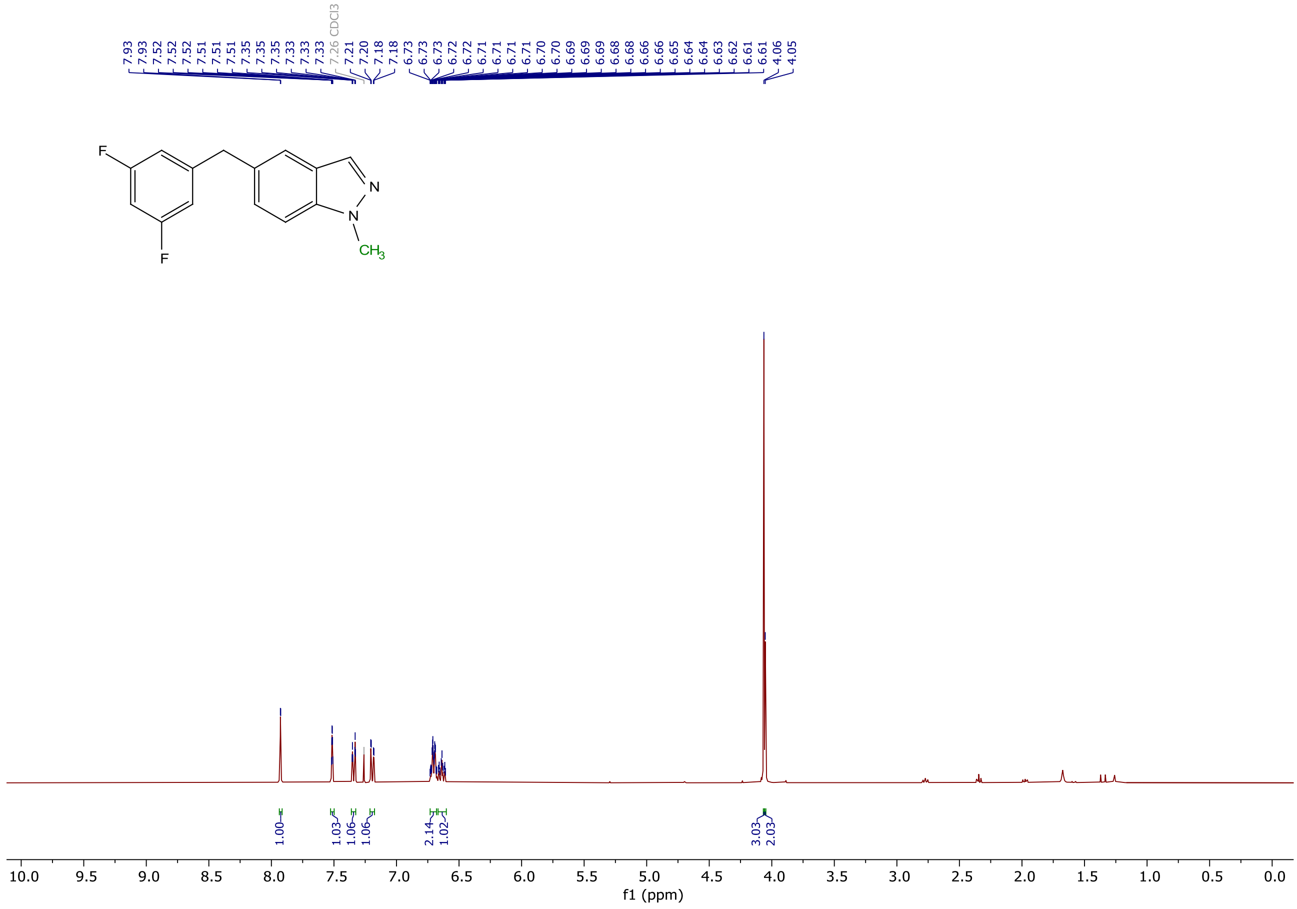

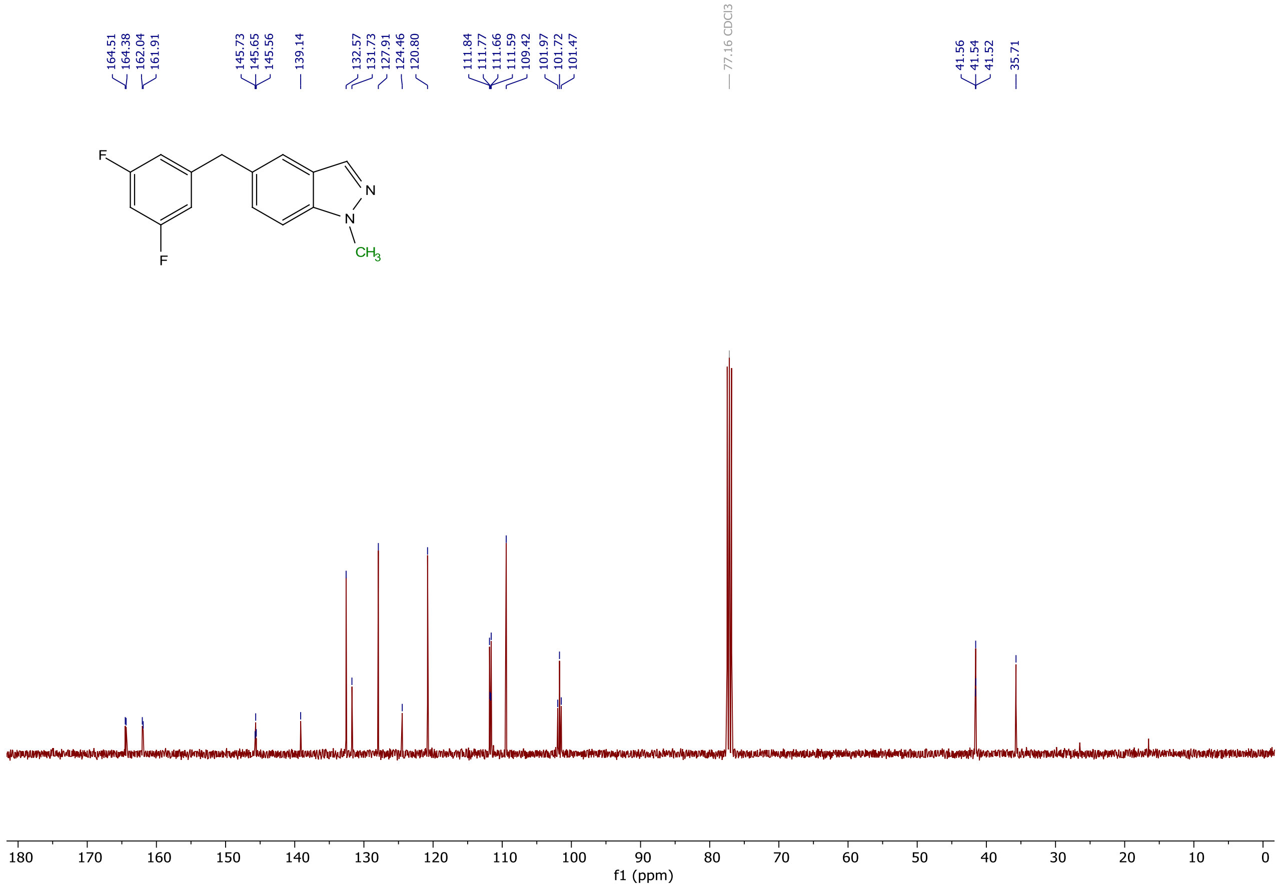

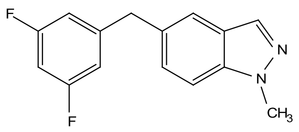

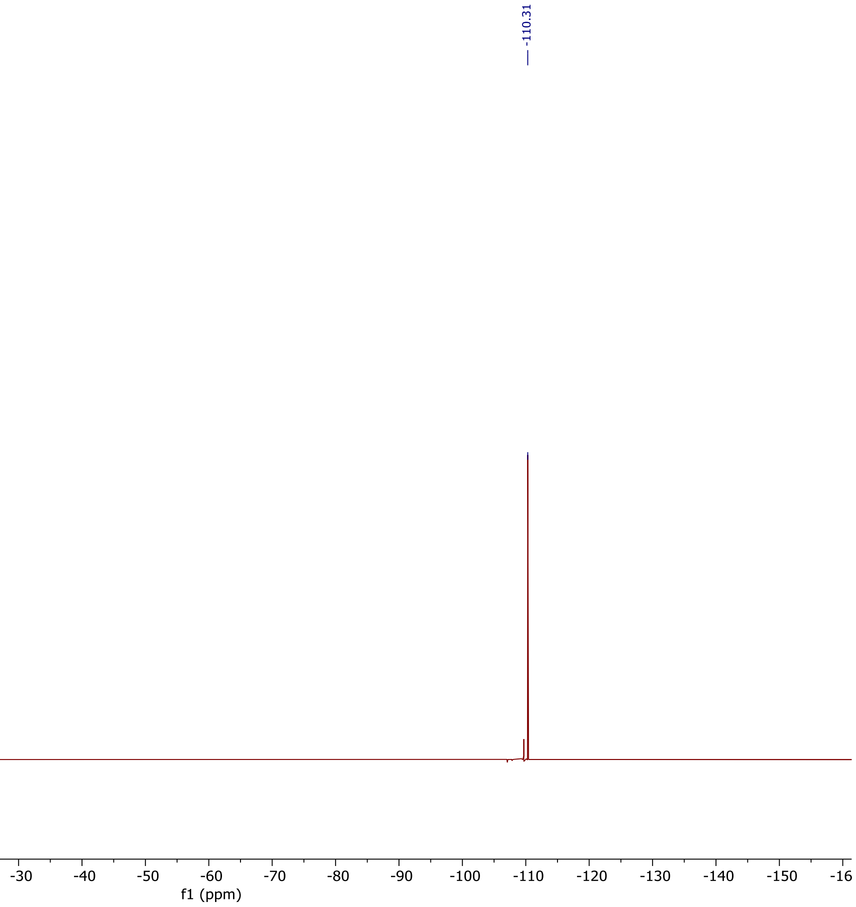
-110.31
40
30
20
10
0
-10
-20
-30
-40
-50
-60
-70
-80
-90
-100
-110
-120
-130
-140
-150
-16
f1 (ppm)

5-(3,5-Difluorobenzyl)-1H-indazole (5aq)

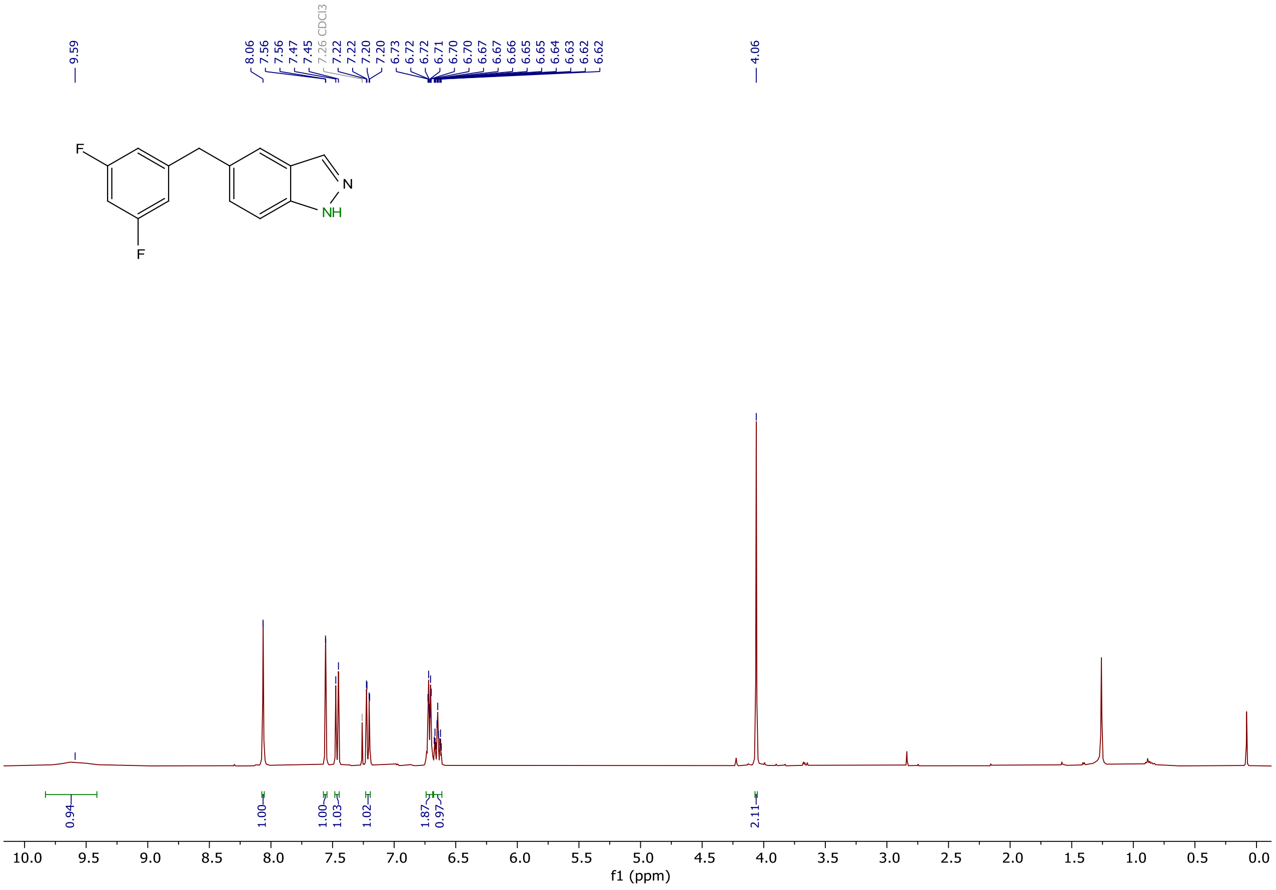

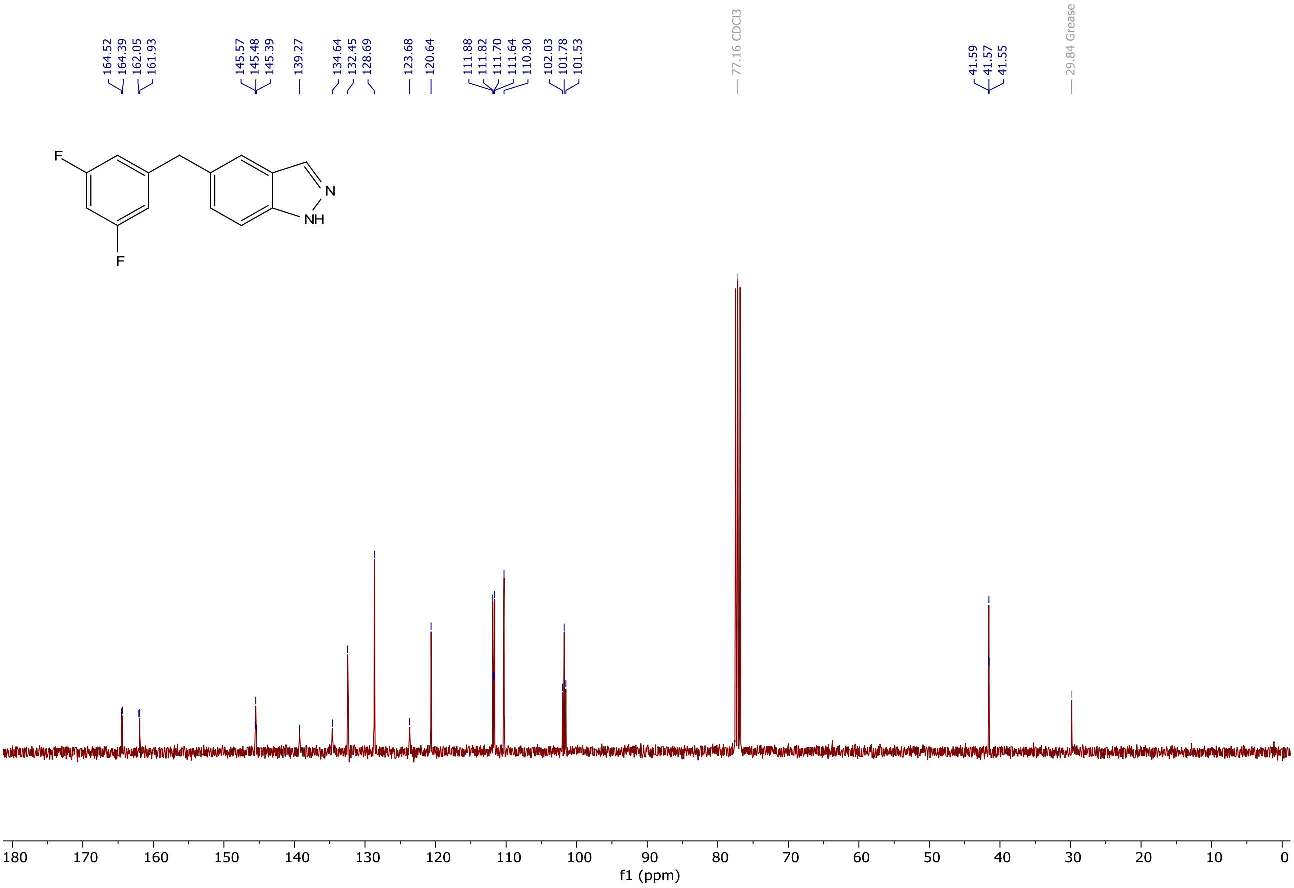

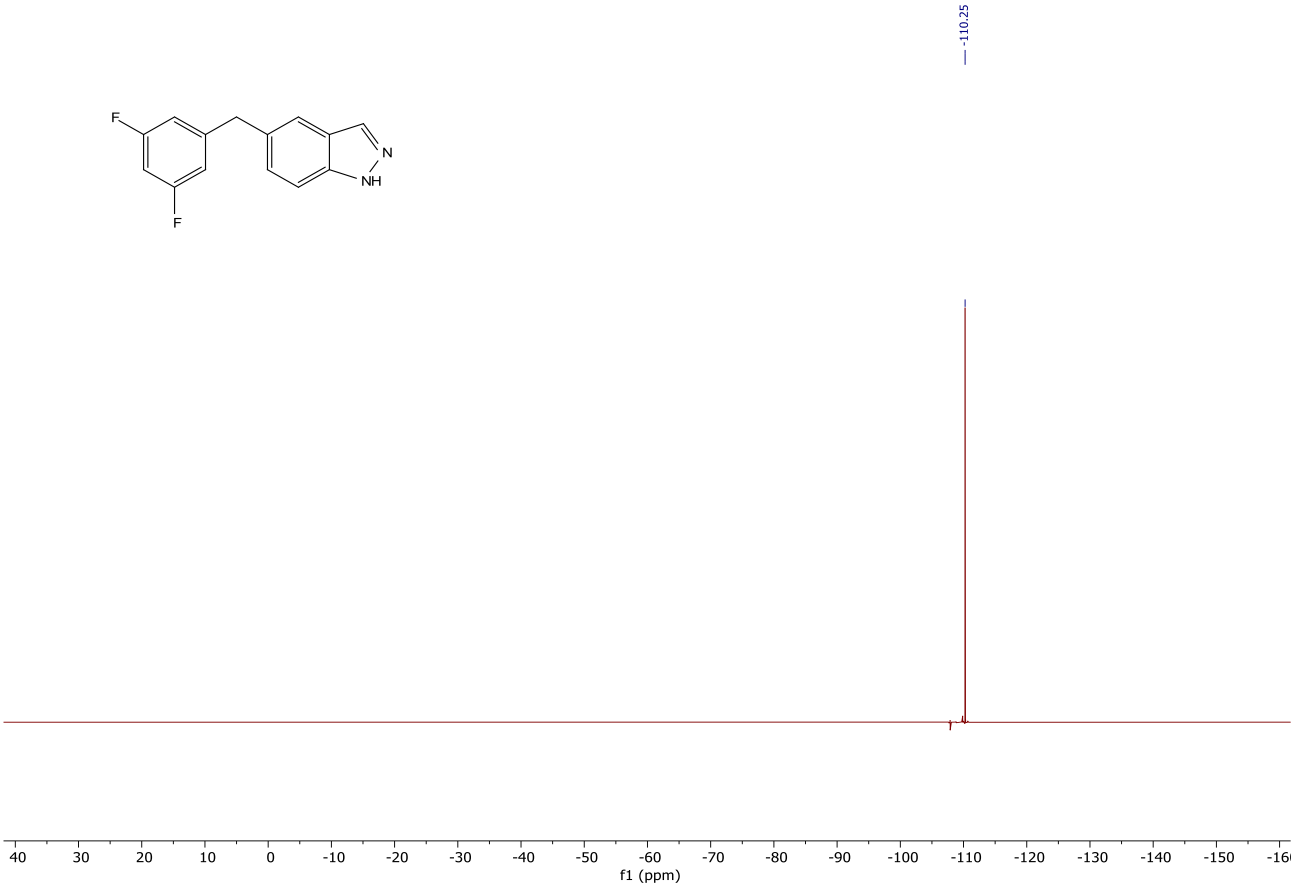
-110.25
F
F
N
NH
40
30
20
10
0
-10
-20
-30
-40
-50
-60
-70
-80
-90
-100
-110
-120
-130
-140
-150
f1 (ppm)

4-(4-(Tert-butyl)benzyl)-1H-indazole (5ar)

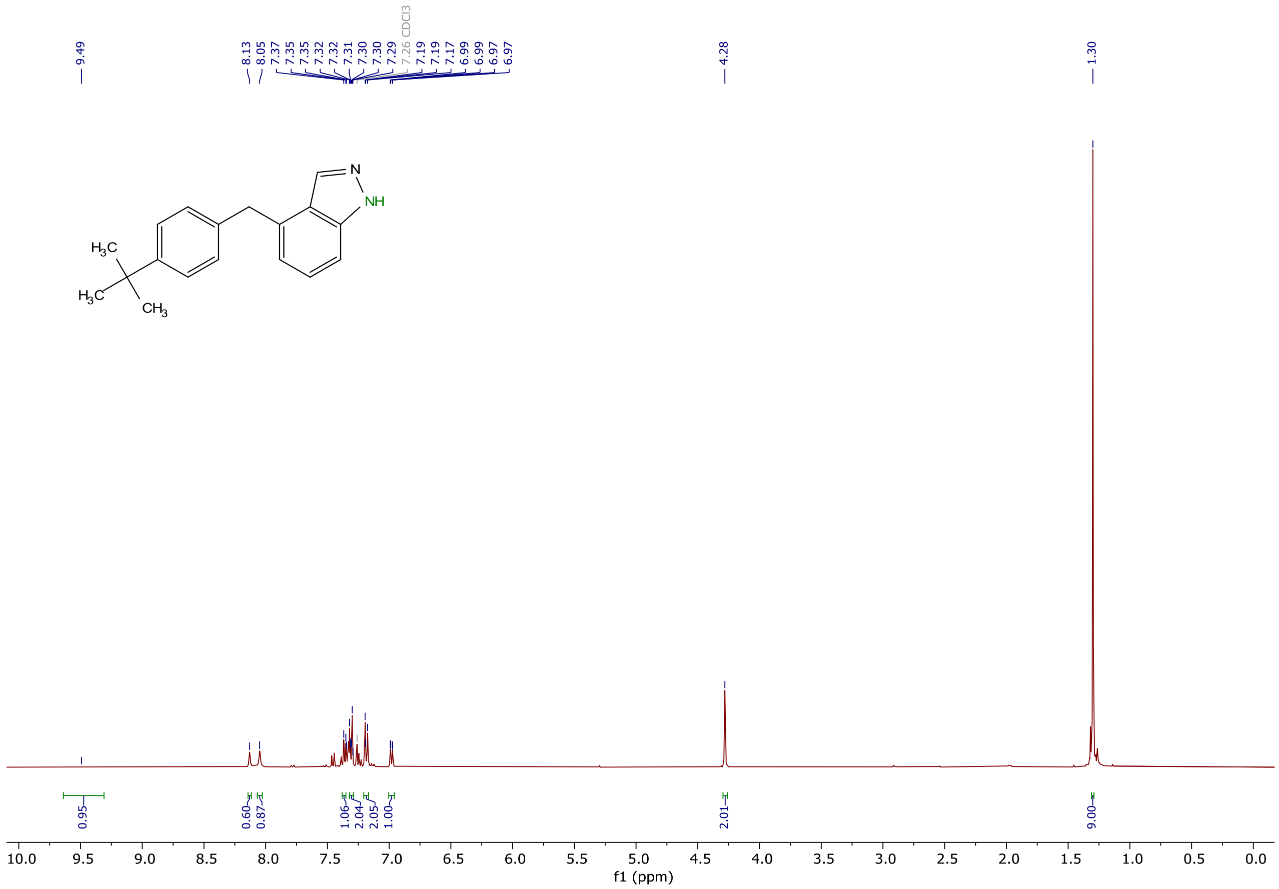

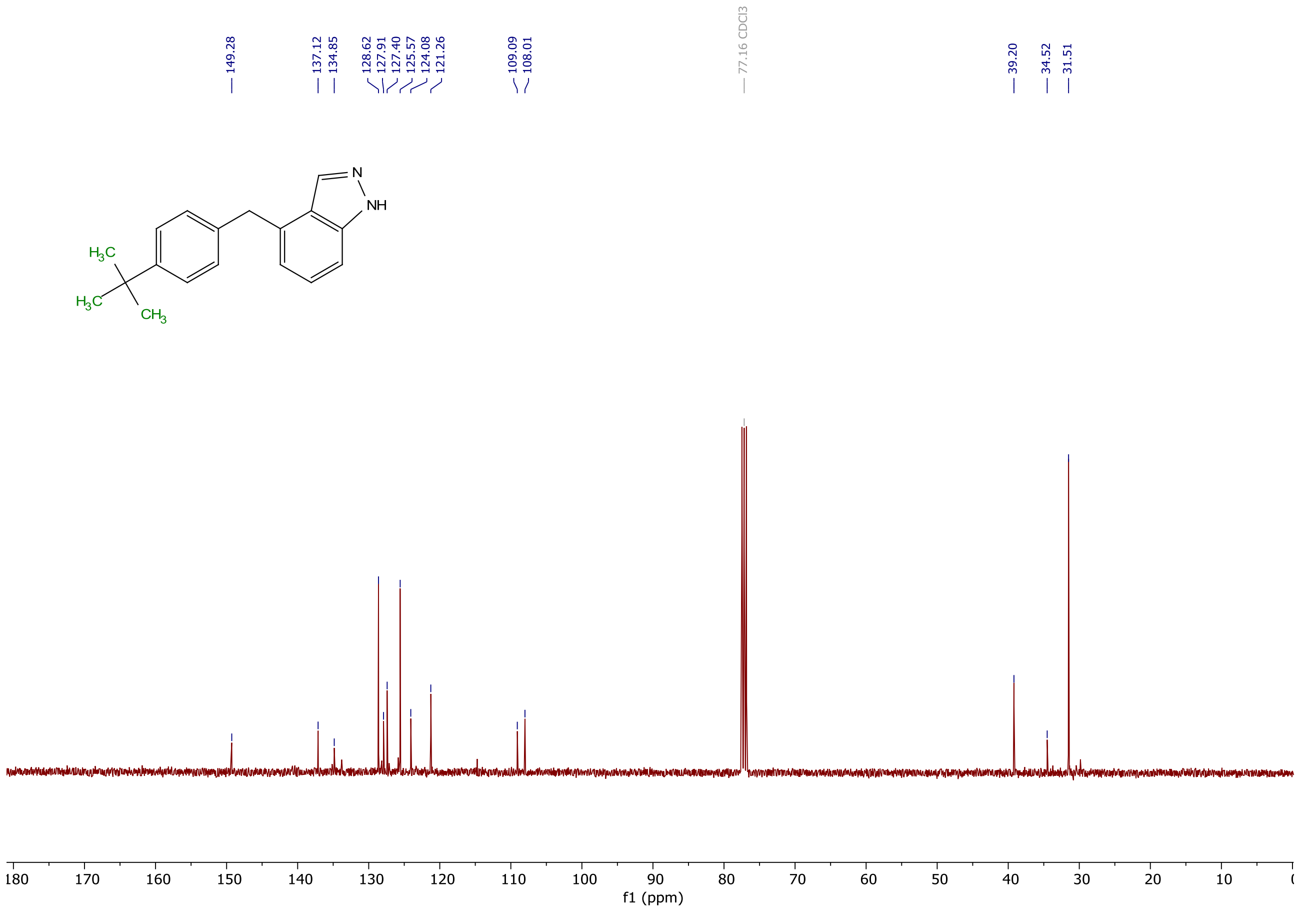

3-(4-Methylbenzyl)benzonitrile (5as)

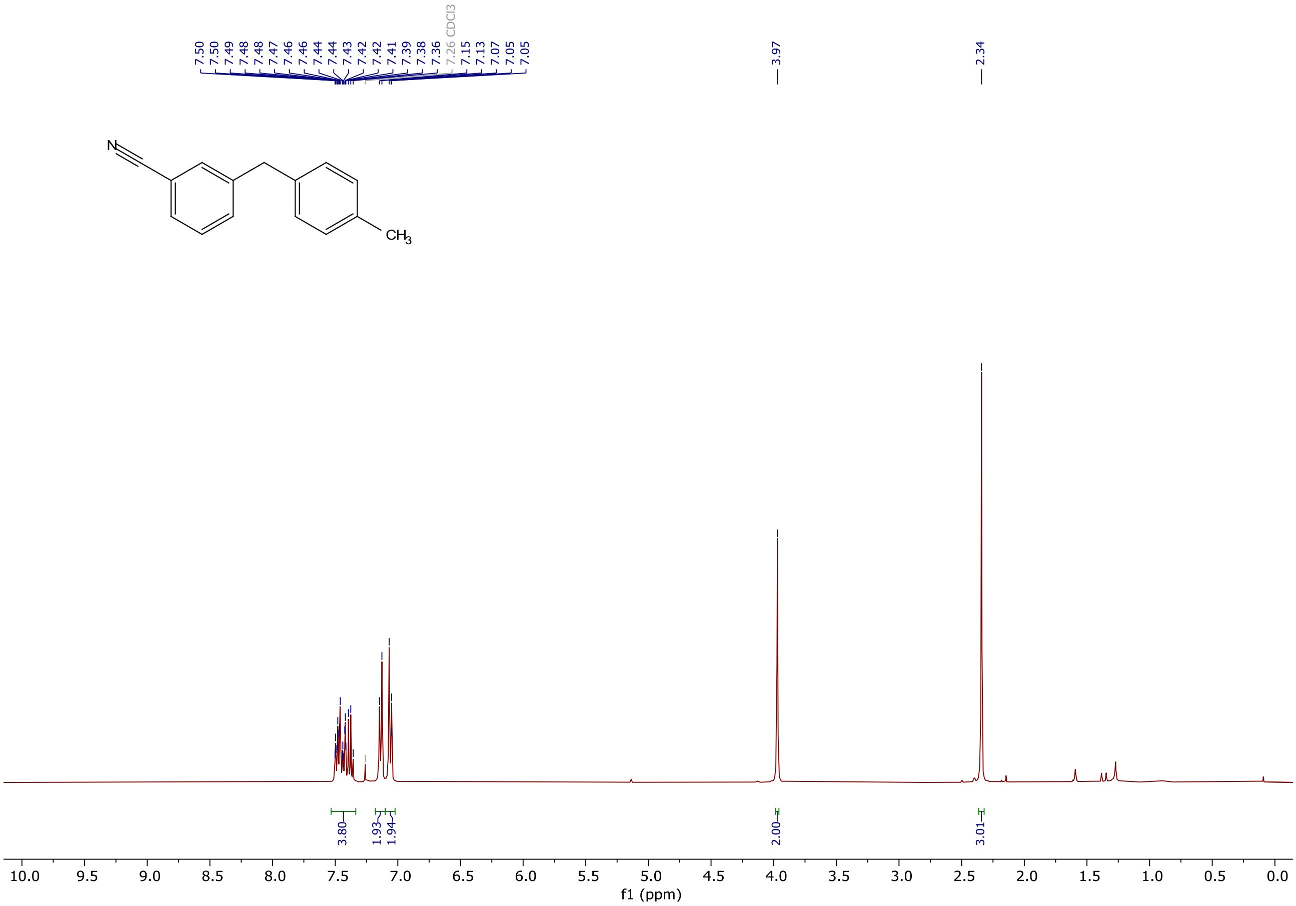

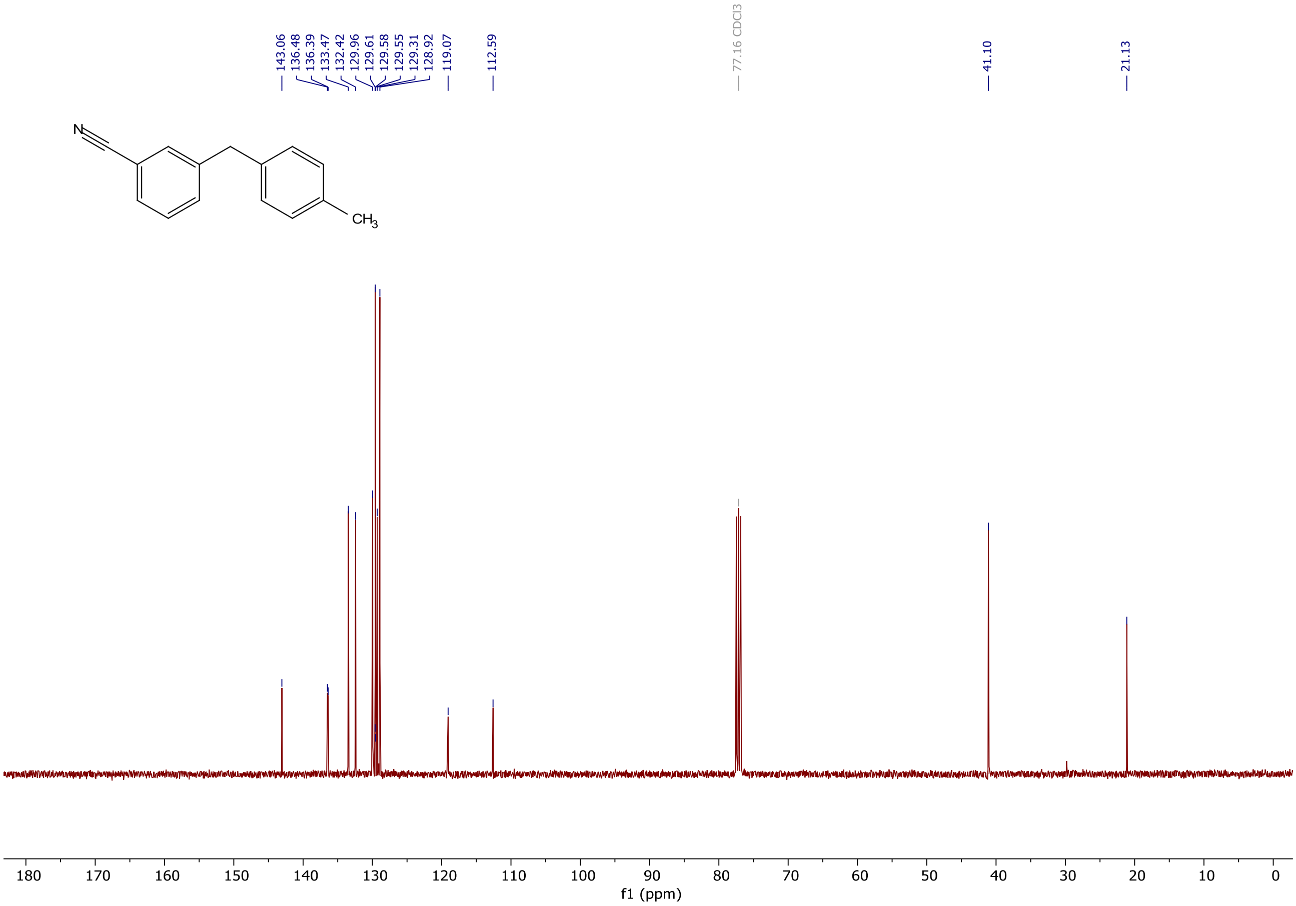

3-((2,3-Dihydrobenzo[b][1,4]dioxin-6-yl)methyl)benzonitrile (5at)

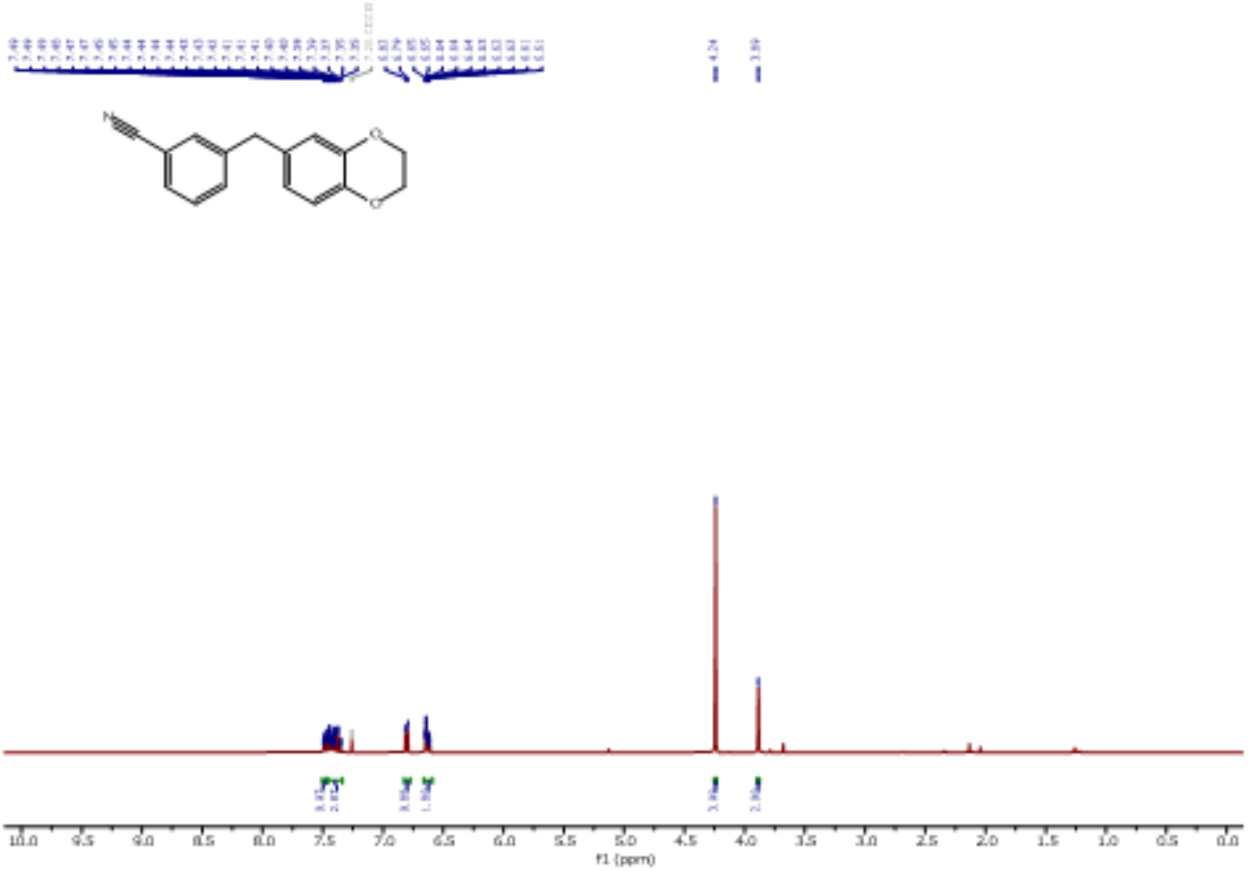

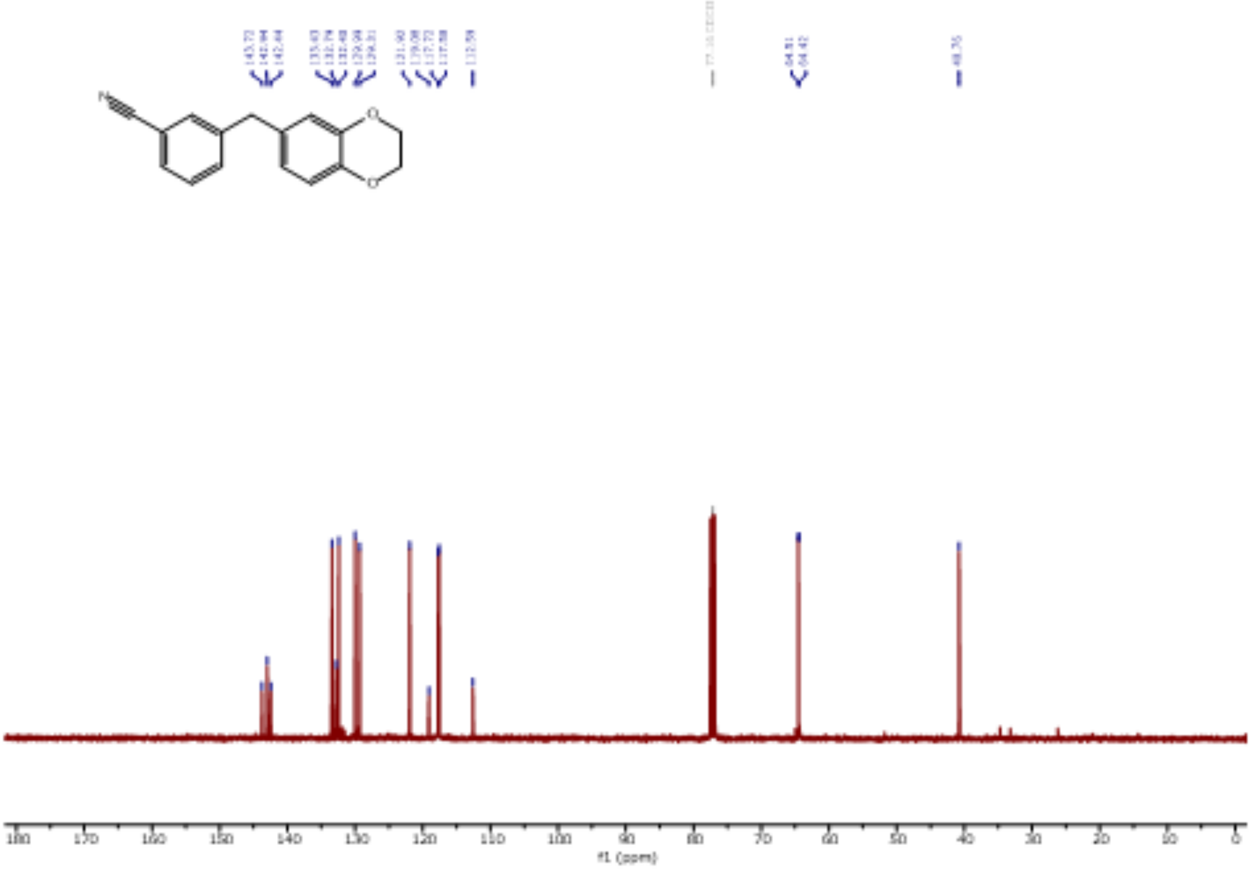

3-(2-Phenylallyl)benzonitrile (5au)

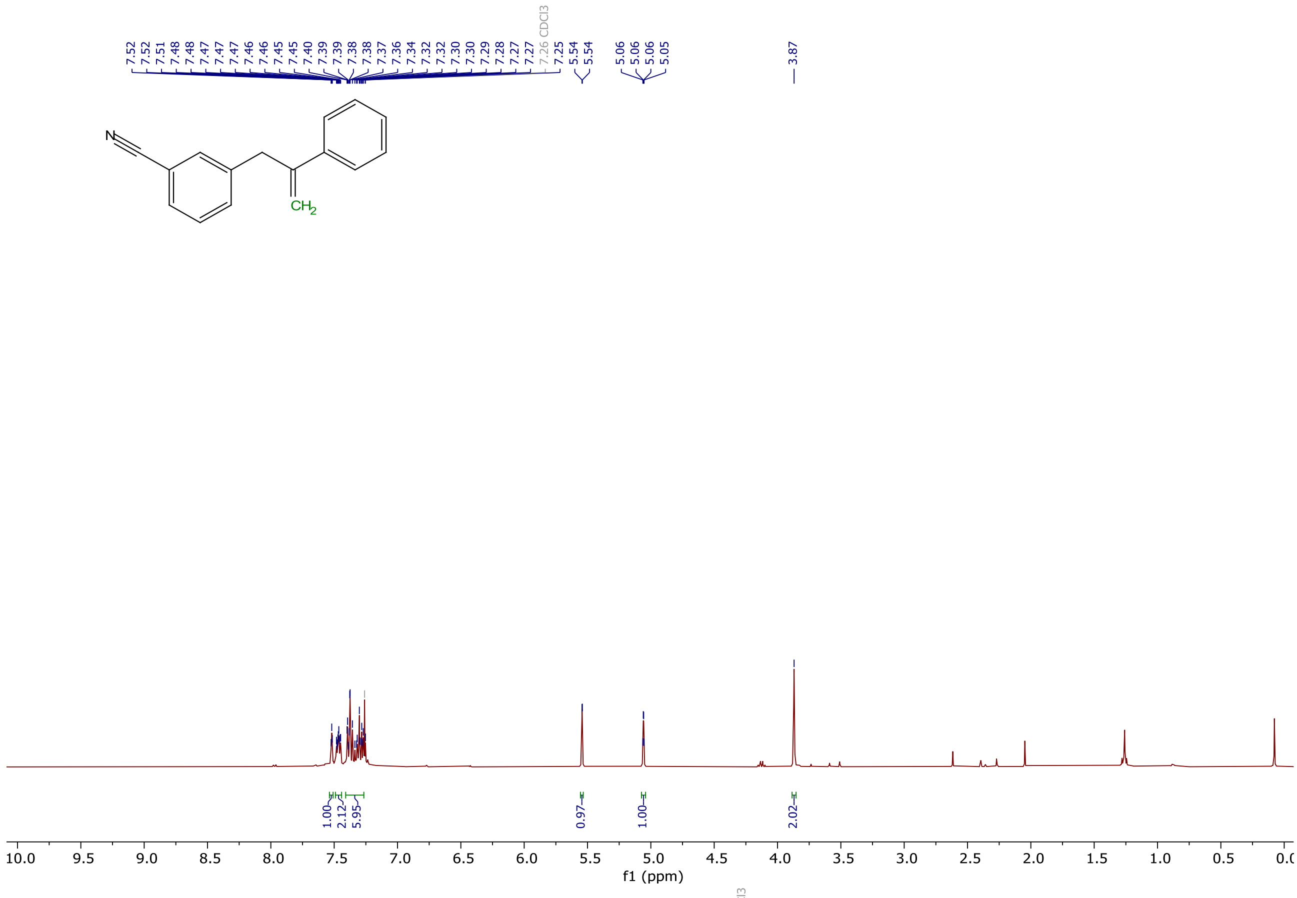

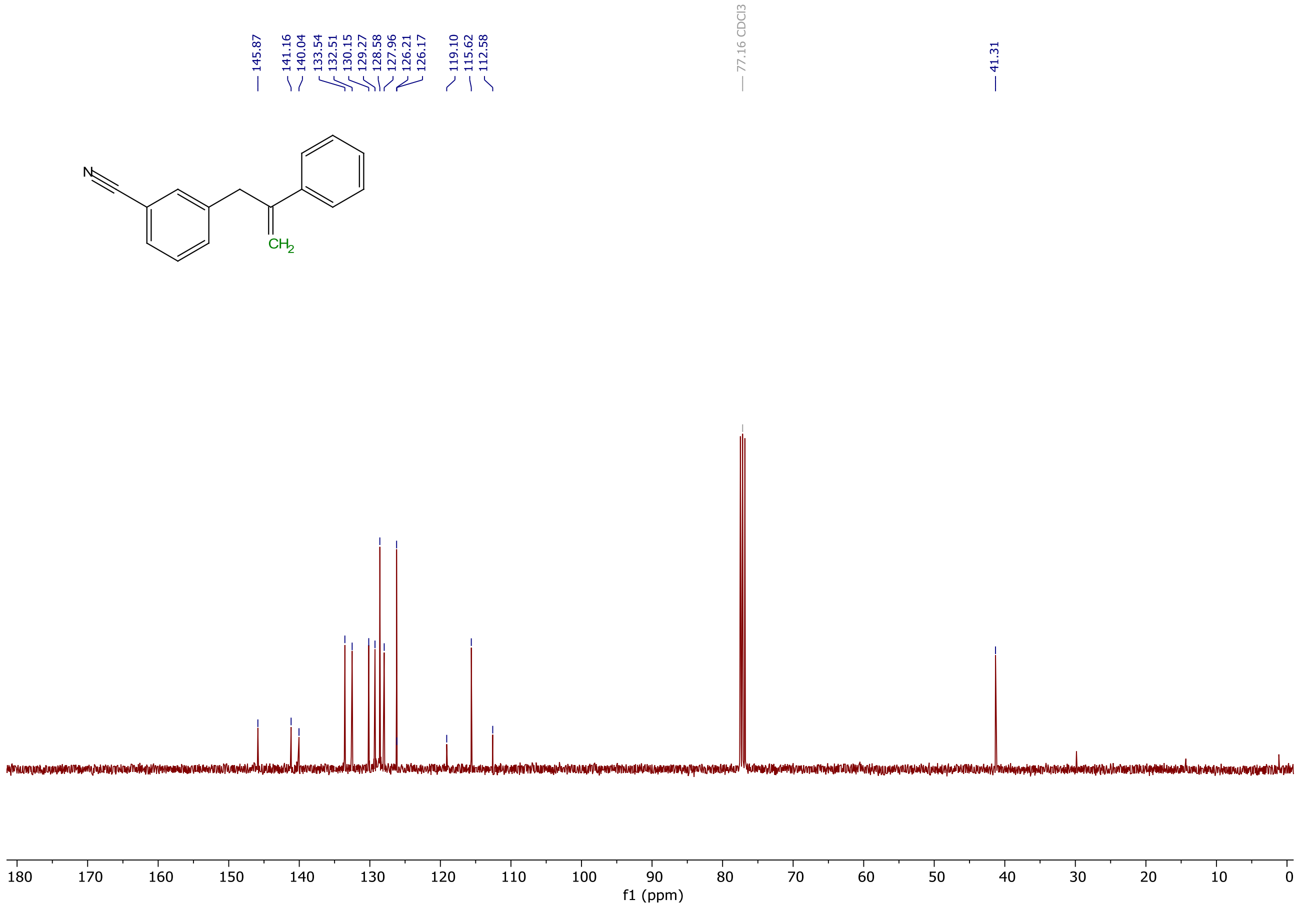

3-(((8R,9S,13S,14S)-13-Methyl-17-oxo-7,8,9,11,12,13,14,15,16,17-decahydro-6H-cyclopenta[a]phenanthren-3-yl)methyl)benzonitrile (5av)

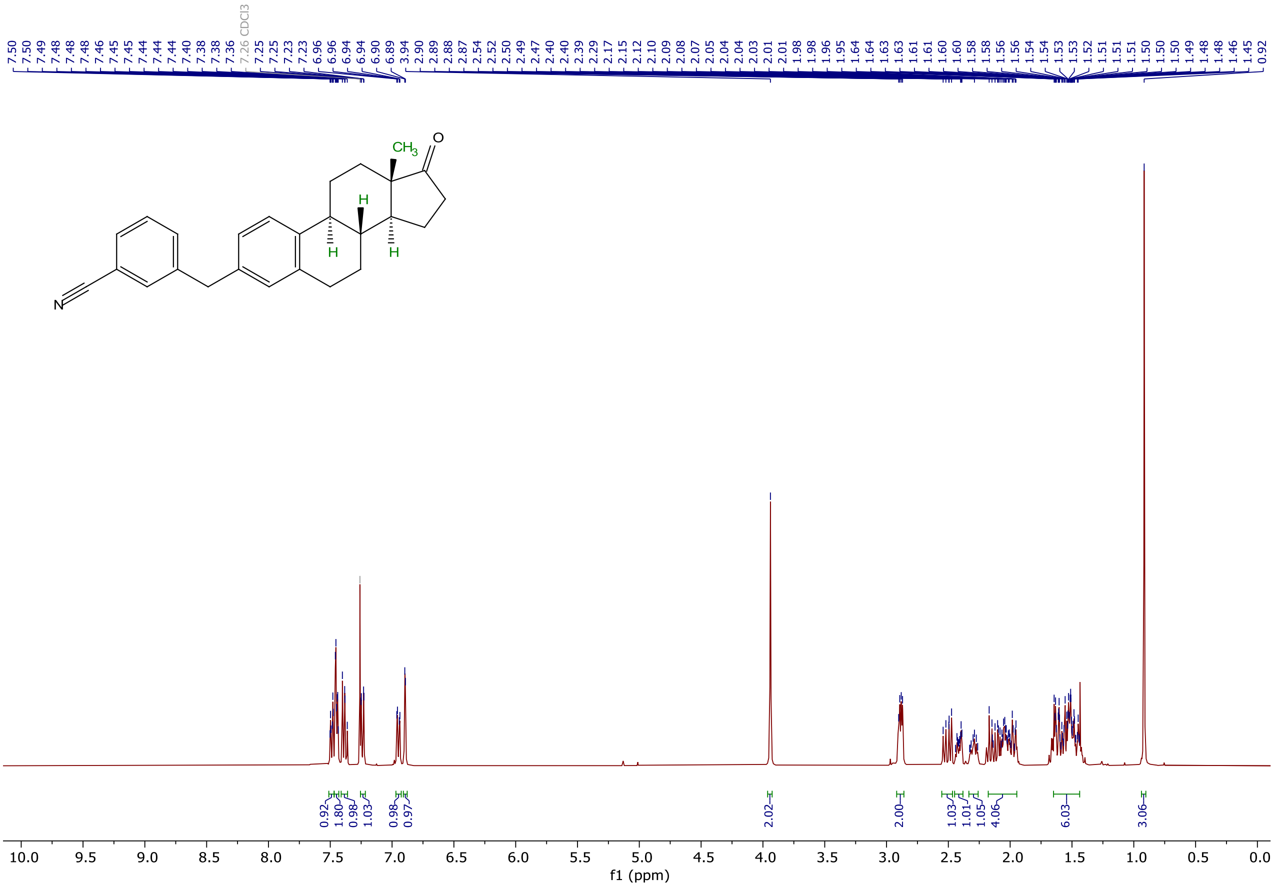

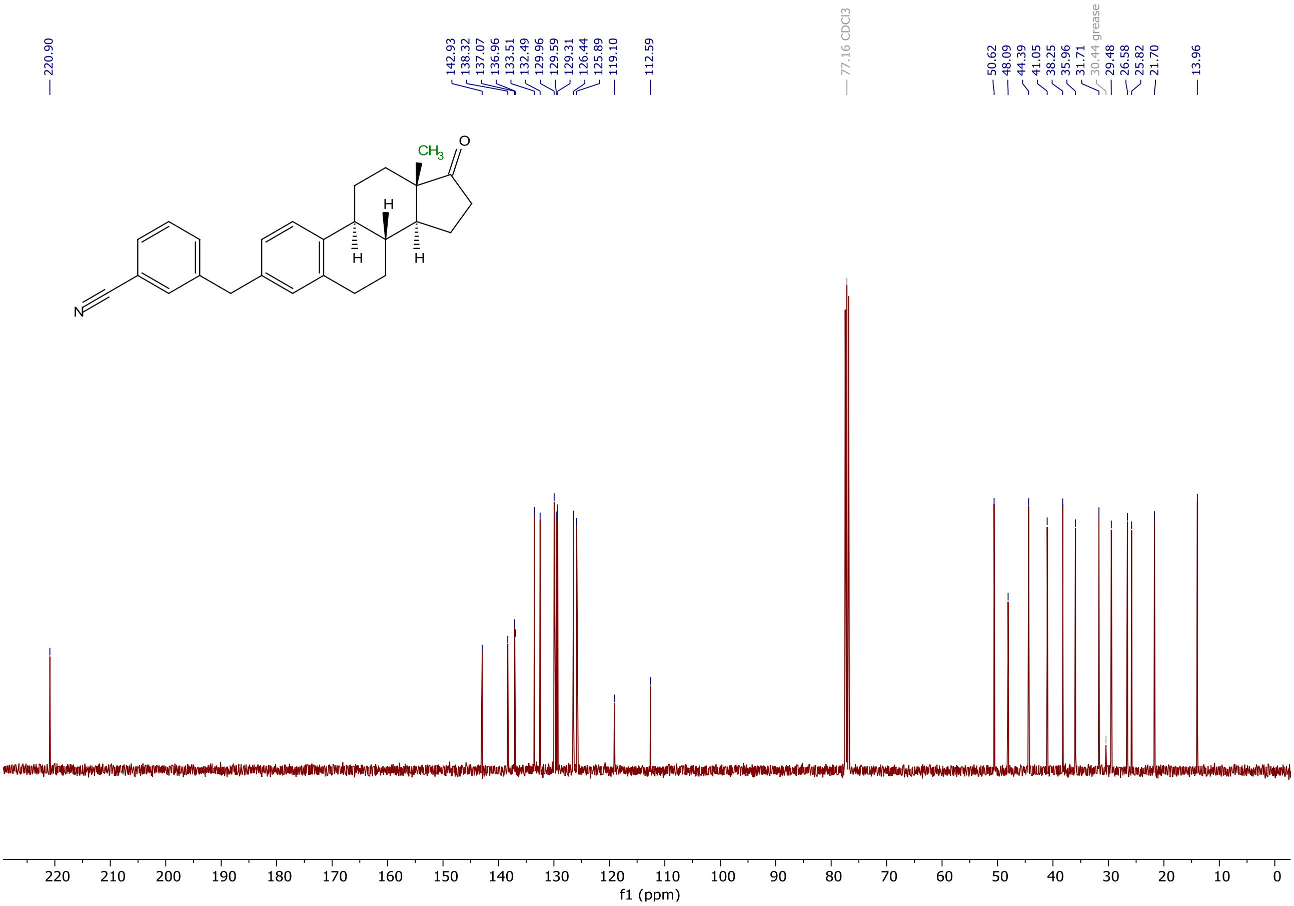

3-(4-(5-(*p*-Tolyl)-3-(trifluoromethyl)-1H-pyrazol-1-yl)benzyl)benzonitrile (5aw)

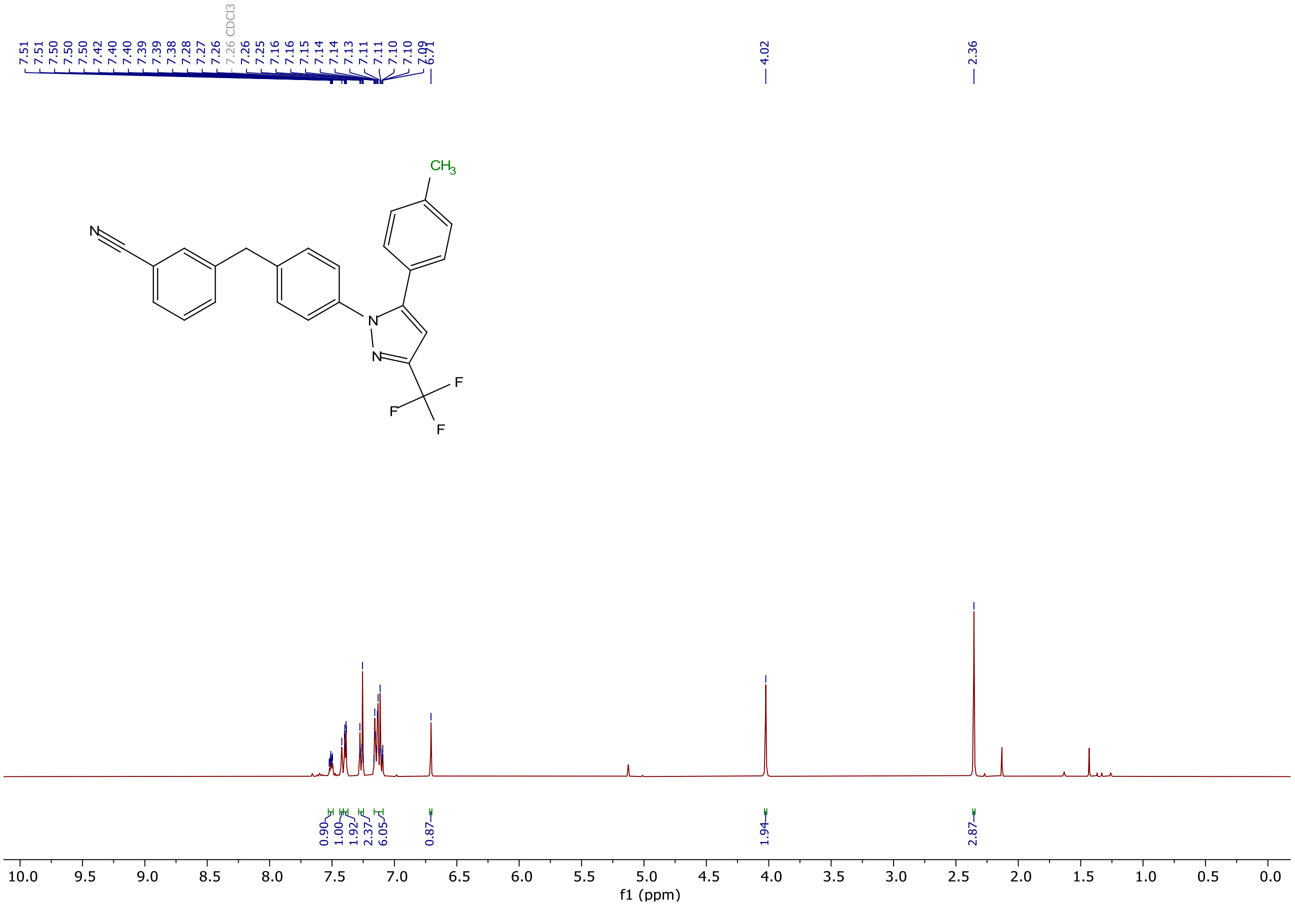

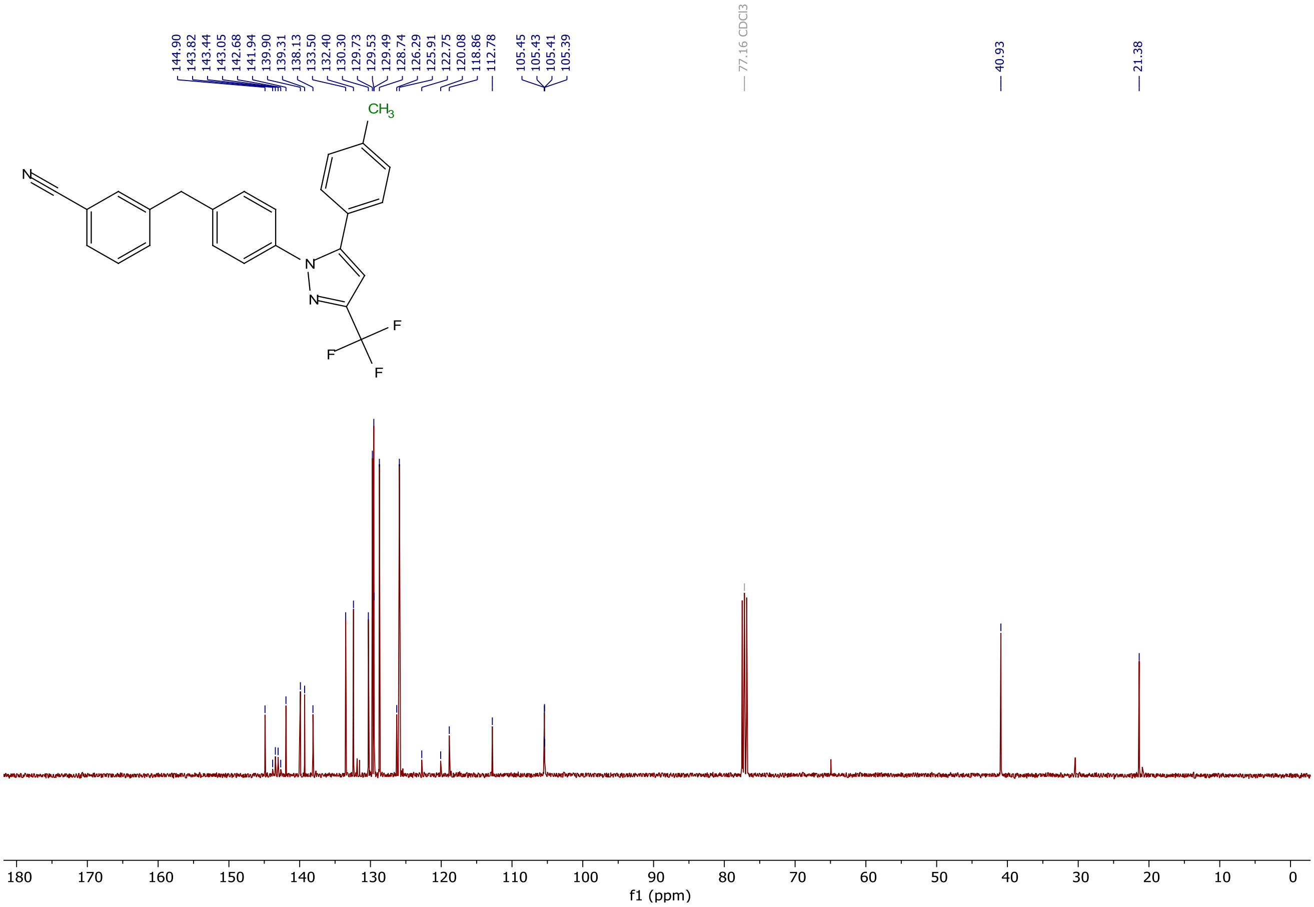

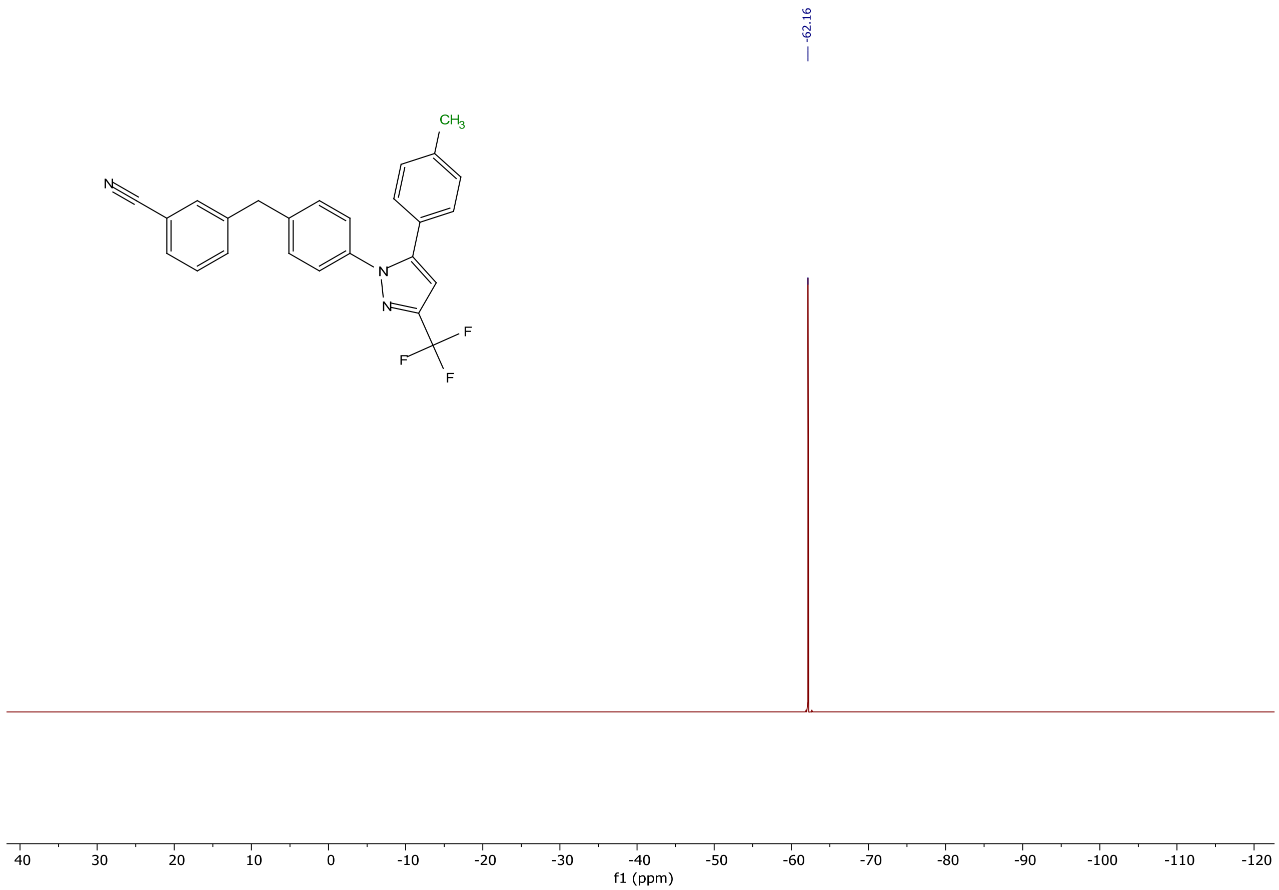

References

[1] A. Shavnya, K. D. Hesp, A. S. Tsai, *Adv. Synth. Catal.* **2018**, *360*, 1768-1774.
[2] [a] C. M. Alder, J. D. Hayler, R. K. Henderson, A. M. Redman, L. Shukla, L. E. Shuster, H. F. Sneddon, *Green Chem.* **2016**, *18*, 3879-3890; [b] F. P. Byrne, S. Jin, G. Paggiola, T. H. M. Petchey, J. H. Clark, T. J. Farmer, A. J. Hunt, C. Robert Mcelroy, J. Sherwood, *Sustain. Chem. Process.* **2016**, *4*.
[3] [a] R. Kuwano, *J. Synth. Org. Chem. Jpn.* **2011**, *69*, 1263-1270; [b] R. Kuwano, J.-Y. Yu, *Heterocycles* **2007**, *74*, 233 - 237; [c] R. Kuwano, M. Yokogi, *Org. Lett.* **2005**, *7*, 945-947; dS. Tabuchi, K. Hirano, M. Miura, *Chem. Eur. J.* **2015**, *21*, 16823-16827; eN. Yoshiaki, S. Ebata, J. Chen, H. Imanaka, T. Hiyama, *Chem. Lett.* **2007**, *36*, 606 - 607; fM. Ohsumi, R. Kuwano, *Chem. Lett.* **2008**, *37*, 796 - 797.
[4] J. K. Kochi, G. S. Hammond, *J. Am. Chem. Soc.* **1953**, *75*, 3443 - 3444.
[5] J. M. Baskin, Z. Wang, *Tetrahedron Lett.* **2002**, *43*, 8479-8483.
[6] W. Li, L. Gao, W. Zhuge, X. Sun, G. Zheng, *Org. Biomol. Chem.* **2017**, *15*, 7819-7823.
[7] Y. Ueno, A. Kojima, M. Okawara, *Chem. Lett.* **1984**, *13*, 2125 - 2128.
[8] M. Haddad, P. Phansavath, V. Ratovelomanana-Vidal, C. Tran, B. Flamme, A. Chagnes, *Synlett* **2018**, *29*, 1622-1626.
[9] F. Kazemi, A. R. Massah, M. Javaherian, *Tetrahedron* **2007**, *63*, 5083-5087.
[10] Q. Wu, S. Han, X. Ren, H. Lu, J. Li, D. Zou, Y. Wu, Y. Wu, *Org. Lett.* **2018**, *20*, 6345-6348.
[11] Z. Tan, H. Jiang, M. Zhang, *Org. Lett.* **2016**, *18*, 3174-3177.
[12] M. Kuriyama, M. Shinozawa, N. Hamaguchi, S. Matsuo, O. Onomura, *J. Org. Chem.* **2014**, *79*, 5921-5928.
[13] M. Ishikura, M. Kamada, I. Oda, T. Ohta, M. Terashima, *J. Heterocycl. Chem.* **1987**, *24*, 377-386.
[14] F. Xie, R. Xie, J.-X. Zhang, H.-F. Jiang, L. Du, M. Zhang, *ACS Catal.* **2017**, *7*, 4780-4785.
[15] X. Chen, L. Zhou, Y. Li, T. Xie, S. Zhou, *J. Org. Chem.* **2014**, *79*, 230-239.
[16] K. Nakajima, S. Nojima, Y. Nishibayashi, *Angew. Chem. Int. Ed.* **2016**, *55*, 14106-14110.
[17] C. Zhao, G.-F. Zha, W.-Y. Fang, K. P. Rakesh, H.-L. Qin, *Eur. J. Org. Chem.* **2019**, *2019*, 1801-1807.
[18] Z.-L. Hou, F. Yang, Z. Zhou, Y.-F. Ao, B. Yao, *Tetrahedron Lett.* **2018**, *59*, 4557 - 4561.
